# Bond fission in monocationic frameworks: diverse fragmentation pathways for phosphinophosphonium cations†
†Electronic supplementary information (ESI) available. See DOI: 10.1039/c5sc03804a


**DOI:** 10.1039/c5sc03804a

**Published:** 2016-01-05

**Authors:** Karlee L. Bamford, Saurabh S. Chitnis, Rhonda L. Stoddard, J. Scott McIndoe, Neil Burford

**Affiliations:** a Department of Chemistry , University of Victoria , P.O. Box 3065, Stn CSC , Victoria , BC V8W 3V6 , Canada . Email: nburford@uvic.ca ; Email: mcindoe@uvic.ca ; Fax: +1 250-721-7147 ; Tel: +1 250-721-7150 ; Tel: +1 250-721-7181

## Abstract

A series of phosphinophosphonium cations ([R_2_PPMe_3_]^+^; R = Me, Et, ^*i*^Pr, ^*t*^Bu, Cy, Ph and N^*i*^Pr_2_) have been prepared and examined by collision-induced dissociation (CID) to determine the fragmentation pathways accessible to these prototypical *catena*-phosphorus cations in the gas-phase. Experimental evidence for fission of P–P and P–E (E = P, C) bonds, and β-hydride elimination has been obtained. Comparison of appearance potentials for the P–P bond dissociation fragments [R_2_P]^+^ (P–P heterolysis) and [PMe_3_]^+^˙ (P–P homolysis) shows that heterolytic P–P cleavage is more sensitive than P–P homolysis towards changes in substitution at the trivalent phosphorus center. The facility of β-hydride elimination increases with the steric bulk of R in [R_2_PPMe_3_]^+^. A density functional theory (DFT) study modelling these observed processes in gas-phase, counterion- and solvent-free conditions, to mimic the mass spectrometric environment, was performed for derivatives of [R_2_PPMe_3_]^+^ (R = Me, Et, ^*i*^Pr, ^*t*^Bu, Ph and N^*i*^Pr_2_), showing good agreement with experimental trends. The unusual observation of both homolytic and heterolytic cleavage pathways for the P–P and P–C bonds reveals new insight into the fundamental aspects of bonding in monocations and undermines the use of simplistic bonding models.

## Abstract 2

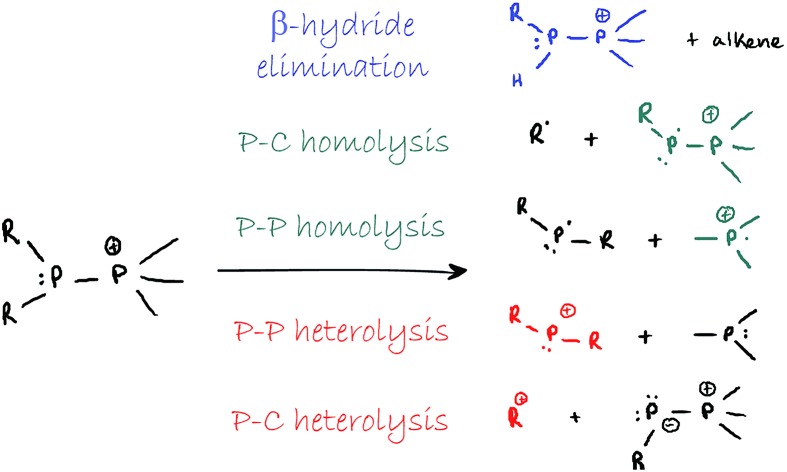
Bond dissociation pathways for phosphinophosphonium cations have been addressed through the concerted application of ESI-CID-MS/MS and DFT modelling.

## Introduction

Bond strength is an essential parameter for discussion of bonding and reactivity. While bond fission can occur by homolysis or heterolysis, for neutral compounds such as alkanes the term “bond strength” generally denotes rupture by the lowest energy homolytic pathway. For example, the C–C bond strength in ethane is listed as 359 kJ mol^–1^, representing homolysis,^1^ which requires one-third of the energy for heterolysis (1297 kJ mol^–1^).^2^

In contrast, heterolytic cleavage of the dative bond is preferred for a neutral donor–acceptor complex with the accommodation of the bond pair by the donor fragment of the complex. For example, the classical coordination complex H_3_NBH_3_, which is isoelectronic with ethane, serves as a source of ammonia and borane through heterolysis as the lowest energy dissociation pathway (130 kJ mol^–1^)^3^ available to the N–B bond. Homolysis is less favoured in this case because the electron affinity of BH_3_ (0.038 eV)^4^ is much less than the ionization energy of NH_3_ (10.35 eV).^5^ Similarly, H_3_NBH_3_ also serves as a source of H_2_ by facile (29 kJ mol^–1^)^6^ heterolytic removal of H^+^ (from N) and H^–^ (from B), which has created interest in the use of this complex as a hydrogen storage medium. Thus, knowledge of energetically preferred bond fission pathways is pertinent to the evolving understanding of chemical bonding within coordination complexes^7^ as well as reactivity and application.

**Scheme 1:**
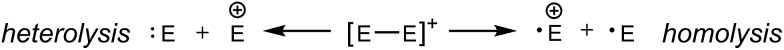
Homolytic and heterolytic fission of homoatomic bonds in monocations.

The preferred dissociation pathways for single bonds in complexes bearing a positive charge are less obvious since potentially unstable open-shell cations (Scheme 1) result from either fission mode of any bond in such species. Phosphinophosphonium cations, [R_2_PPR_3_]^+^, are prototypical examples of monocations featuring a homoatomic bond. Experimental evidence for heterolytic P–P cleavage has been reported in the form of ligand and acceptor exchange studies,^8^ but the evidence required to demonstrate a dissociative mechanism involving free phosphenium ions as intermediates is lacking. Phosphenium ions have only been isolated when π-donating or sterically hindered substituents are employed,^9^ and therefore, P–P heterolysis may not be accessible with small alkyl substituents at phosphorus. There is no evidence for homolytic P–P cleavage in phosphinophosphonium cations, despite the predicted accessibility of this pathway in quantum chemical studies depending upon the electronic and steric properties of the substituents around the P–P bond.^10^,^11^ Experimental evidence for both bond cleavage modes operating within a single phosphinophosphonium has not been reported, nor has the preference for either mode been experimentally assessed under conditions that favour neither homolysis nor heterolysis products.

While quantitative determination of bond strengths is experimentally challenging for molecules of this type, qualitative approaches have been developed to probe the relative thresholds for various bond fission processes in a molecule. Tandem mass spectrometry (MS/MS) provides one such approach through collision-induced dissociation (CID). A highly dynamic technique, CID is capable of probing a wide range of interaction types^12^–^15^ through the inelastic collision of a chosen molecular ion with an inert gas molecule (*e.g.* Ar). Bond energies can be quantified for well-behaved systems (*i.e.* where fragmentation occurs *via* a single pathway) through treatment of the kinetic shift by extraction of threshold energies with programs such as CRUNCH16*a* and LCID.16*b* The appearance potentials of fragments formed from conversion of kinetic energy to potential energy upon collision can be qualitatively compared to determine the kinetically preferred bond fragmentation pathways in the gas phase. Electrospray ionization (ESI) is ideally suited to produce ions of interest for CID-MS/MS experiments because the source simply desolvates solution-phase ions and hence causes minimal fragmentation of the parent ion during its transit into the gas phase.^17^

A collection of alkyl- and aryl-substituted diphosphines (*e.g.* R_2_PPR_2_ where R = Me, Et, and ^*t*^Bu) have been the subject of sporadic CID studies^18^–^22^ utilizing electron impact mass spectrometry, but the use of this ionization method limits the practical relevance of these studies as the electronic structures of radical cation molecular ions differ from those of neutral precursors. Isolable polyphosphorus cations have not been studied by mass spectrometry using ESI-MS methods, despite the similarity of mass spectrometric conditions with reported gas-phase theoretical models.^10^,^23^,^24^

We now report the first experimental evidence for both homolytic and heterolytic P–E (E = P, C) bond dissociation processes in the gas phase within members of a systematically-varied series of isolable phosphinophosphonium cations, [R_2_PPMe_3_]^+^ (R = Me, Et, ^*i*^Pr, ^*t*^Bu, Cy, Ph, and N^*i*^Pr_2_). The relative preference for P–P homolysis and heterolysis has been assessed in each case to clarify the fundamental ambiguity of homoatomic bond dissociation pathways in cationic complexes, and the results are consistent with charge-delocalization over the molecular framework. In addition, a remarkable diversity of hitherto unpredicted unimolecular fragmentation pathways has been discovered for these prototypical *catena*-phosphorus cations. The observed processes have been comprehensively modeled in the gas phase using benchmarked quantum-chemical methods and rationalized as a function of the electronic and steric properties of the substituents at the trivalent phosphorus center. The concerted application of the ESI-CID-MS/MS experiment and computational chemistry defines a state-of-the-art qualitative methodology for experimentally addressing challenging questions regarding the nature of chemical bonding.^7^,^14^,^25^

## Experimental

A series of phosphinophosphonium triflate salts of the generic formula [R_2_PPMe_3_][OTf] (R = Me, Et, ^*i*^Pr, ^*t*^Bu, Cy, Ph, and N^*i*^Pr_2_) were prepared according to published synthetic methods^26^,^27^ and analysed from dilute solutions by ESI-MS/MS. All mass spectra were collected on a Micromass Q-ToF Micro mass spectrometer in positive mode, using electrospray ionization: capillary voltage, 3000 V; sample cone voltage, 15 V; extraction voltage, 0.5 V; source temperature, 70 °C; desolvation temperature, 200 °C; cone gas flow, 100 L h^–1^; desolvation gas flow, 100 L h^–1^; collision voltage 1–50 V for MS/MS experiments; MCP voltage, 2700 V. Data collected in CID experiments are presented in terms of averaged intensities normalized with respect to the total ion count and collision energies normalized with respect to the mass of the fragmenting phosphinophosphonium cation rather than absolute intensity and time, as in the raw data, to allow discussion of relative appearance potentials for fragments irrespective of the identity of the parent phosphinophosphonium cation. Mass normalization was accomplished using the formula *E*_0_ = *E*_lab_ × *m*_Ar_/(*m*_Ar_ + *m*_M_), where *E*_0_ is the mass normalized collision voltage, *E*_lab_ is the collision voltage set in lab, *m*_Ar_ is the mass of the [argon] collision gas, and *m*_M_ is the mass of the molecular ion selected for CID. The appearance potential of a fragment is proportional to the energetic requirement for that fragmentation process and, thus, the appearance potentials of [R_2_P]^+^ and [PMe_3_]^+^˙ for a given substituent R represent relative energy requirements for heterolysis and homolysis, respectively.

## Results and discussion

**Scheme 2:**
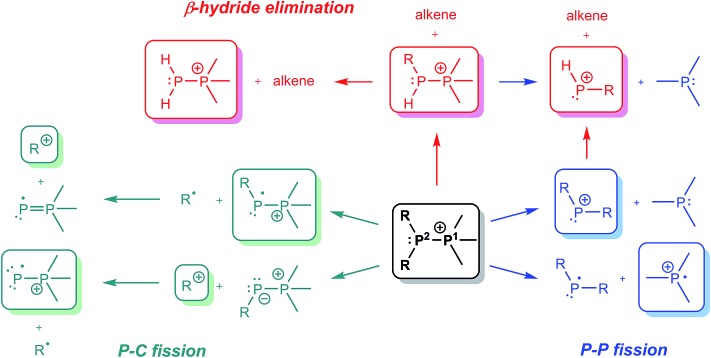
Mass spectrometrically observed dissociation pathways for [R_2_PPMe_3_]^+^ cations, where R = Me, Et, ^*i*^Pr, ^*t*^Bu, Cy, or Ph (only cationic species, in rounded boxes, are detected in CID-MS/MS).

**Fig. 1:**
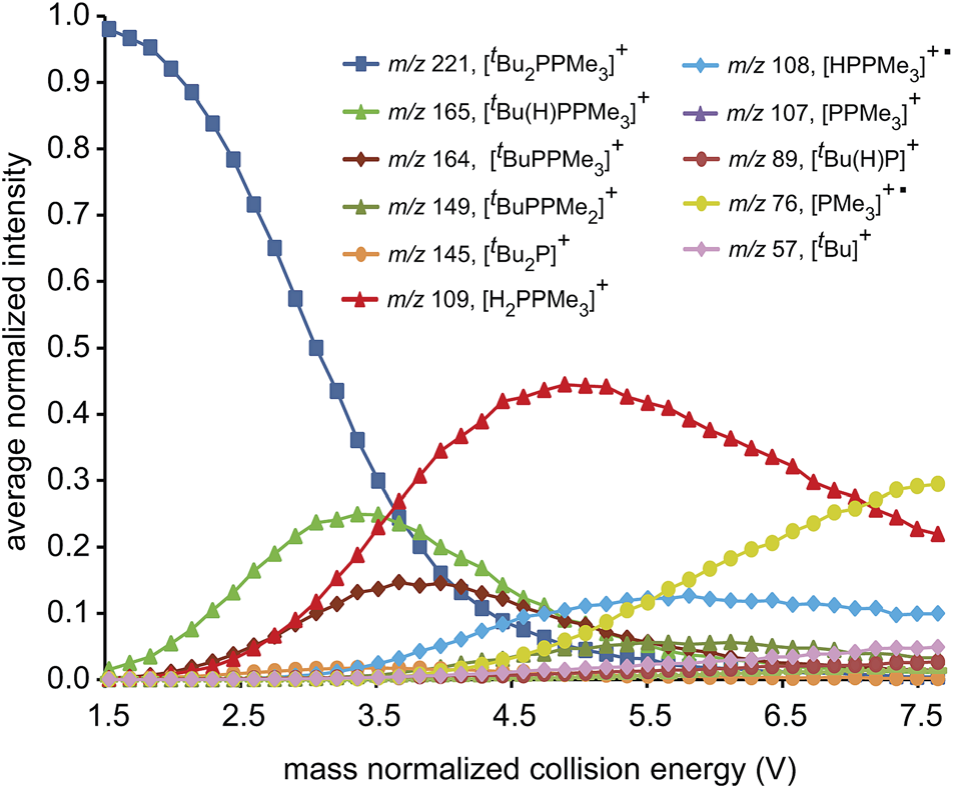
Fragmentation plot for [^*t*^Bu_2_PPMe_3_]^+^. Average normalized intensities of [^*t*^Bu_2_PPMe_3_]^+^ and its array of daughter fragments as a function of mass normalized collision energy in ESI-CID-MS/MS experiments.

ESI-CID-MS/MS experiments of [R_2_PPMe_3_]^+^ molecular ions show that a diverse array of fragmentation processes are accessible to phosphinophosphonium cations, including P–P fission, P–C fission, and β-hydride elimination (Scheme 2). Fig. 1 shows the average intensities of the parent ion [^*t*^Bu_2_PPMe_3_]^+^ and its daughter fragments as a function of increasing collision energy, normalized to the total ion current for each MS/MS experiment (*y* axis), and plotted against the mass normalized collision energy (*x* axis). [^*t*^Bu_2_PPMe_3_]^+^ is an illustrative example of the series [R_2_PPMe_3_]^+^ since all processes in Scheme 2 are observed (see also Fig. S18d†), whereas for other substitutions only some of the processes in Scheme 2 are observed (Table 1).

**Table 1:** Summary of Dissociation Pathways Observed by ESI-CID-MS/MS^*a*^

Process	Observable fragment	Me	Et	^*i*^Pr	^*t*^Bu	Cy	Ph
Heterolytic P_1_–P_2_	[R_2_P]^+^	✓	✓	✓	✓	✓	✓
	[R(H)P]^+^	n.d.	✓	✓	✓	✓	✗
Homolytic P_1_–P_2_	[PMe_3_]^+^˙	✓	✓	✓	✓	✓	✗
Heterolytic P_2_–C	R^+^	n.d.	n.d.	n.d.	✓	✓	✗
Homolytic P_2_–C	[RPPMe_3_]^+^˙	✓	✓	✓	✓	✓	✗
	[HPPMe_3_]^+^˙	—	✓	✓	✓	✓	✗
	[PPMe_3_]^+^	?	✓	✓	✗	✗	✗
Homolytic P_1_–C	[R_2_PPMe_2_]^+^˙	?	✗	✗	✗	✗	✗
	[RPPMe_2_]^+^	?	✓	✓	✓	✗	✗
β-Hydride elimination	[R(H)PPMe_3_]^+^	—	✓	✓	✓	✓	✗
	[H_2_PPMe_3_]^+^	—	✗	✓	✓	✓	✗

^*a*^n.d. indicates processes that were not detected because the indicated fragments were below the detection limit of *m*/*z* 50; ✗ indicates processes that were not observed; ? indicates multiple pathways resulting in the same *m*/*z* fragment observed by MS/MS; processes that are not possible for a particular substitution are denoted with a dash; heterolytic P_1_–C cleavage is not detectable due to the mass of Me^+^ being less than *m*/*z* 50.

Dissociation pathways inferred from these characteristic fragments include primary processes occurring in the parent molecular ion, [R_2_PPMe_3_]^+^, and secondary processes occurring in the products generated by primary processes. The large number of products observed from fragmentation of each phosphinophosphonium cation (see Fig. S18 and S19† for summary fragmentation plots) is largely due to these secondary processes. For example, two sequential losses of the ^*t*^Bu groups are observed following homolytic P–C cleavage in [^*t*^Bu_2_PPMe_3_]^+^, resulting in detection of [^*t*^BuPPMe_3_]^+^ from the primary process and [PPMe_3_]^+^ from the secondary process (Fig. 1).

**Fig. 2:**
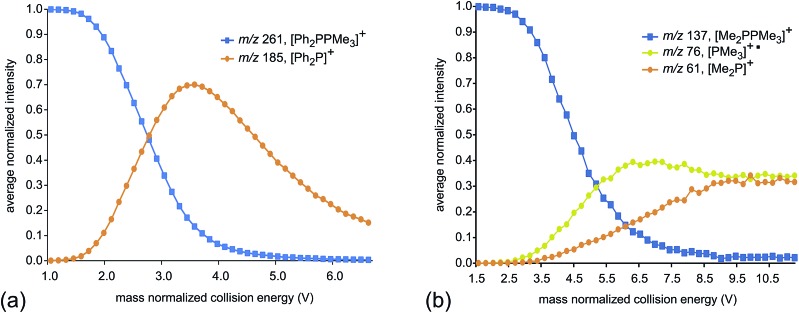
Average normalized intensities of [R_2_PPMe_3_]^+^, [PMe_3_]^+^˙ and [R_2_P]^+^ (R = Ph (a) and Me (b)) as a function of mass normalized collision energy in ESI-CID-MS/MS experiments.

**Scheme 3:**
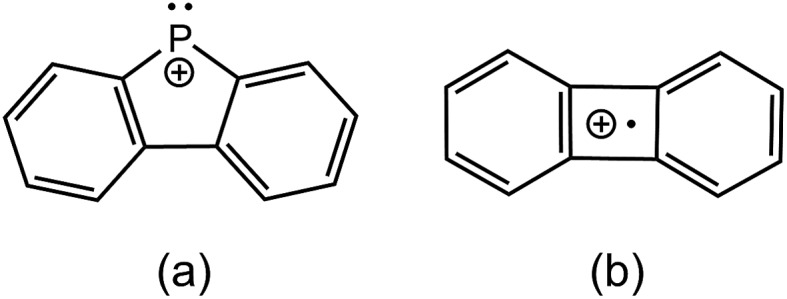
Postulated structures of the *o*-biphenylene phosphenium (a) and [(C_6_H_4_)_2_]^+^˙ (b) fragments.

Fig. 2 presents fragmentation plots for [R_2_PPMe_3_]^+^, R = Ph (a) and Me (b), including traces for the occurrence of P–P heterolysis and homolysis as indicated by the appearance of [R_2_P]^+^ and [PMe_3_]^+^˙, respectively. The fewest dissociation pathways are observed for R = Ph, for which the primary P–P heterolytic cleavage forming [Ph_2_P]^+^ and the secondary loss of H_2_ from this fragment to give the *o*-biphenylene phosphenium ion (Scheme 3a) are the most significant processes (see Fig. S18f and S19f†). The formation of the *o*-biphenylene phosphenium ion and several low intensity fragments (*e.g.* [(C_6_H_4_)_2_]^+^, *m*/*z* 152) (Scheme 3b) have previously been observed in MS studies involving triphenylphosphine.^28^,^29^ The trace for [Ph_2_P]^+^ exhibits typical intermediate behaviour and diminishes concomitantly with the formation of [(C_6_H_4_)_2_]^+^˙, suggesting that formation of the radical cation is a secondary process.

**Fig. 3:**
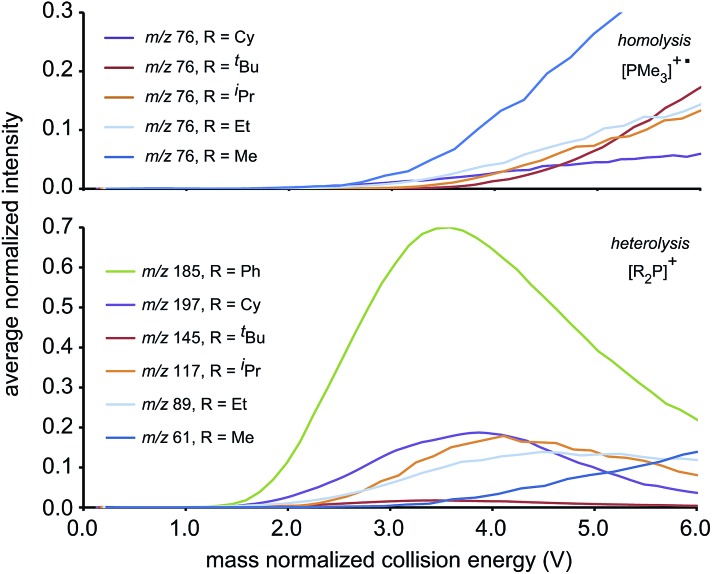
The average normalized intensities of P–P fission products [PMe_3_]^+^˙ (top) and [R_2_P]^+^ (bottom) with increasing mass normalized collision energy.

We ascribe the preference for heterolytic P–P cleavage in R = Ph to resonance stabilization of the phosphenium center in [Ph_2_P]^+^ by π-donation from the phenyl substituents to the vacant p-orbital at the phosphenium (analogous to resonance stabilization of [Ph_3_C]^+^).^18^ While similar behaviour was anticipated for the R = N^*i*^Pr_2_ derivative, the fragment of greatest mass observed for solutions of [(N^*i*^Pr_2_)_2_PPMe_3_][OTf] prior to any collision-induced dissociation was unassignable (see Fig. S13 and S14†). The fragmentation data for [Ph_2_PPMe_3_]^+^ is unique amongst the derivatives of [R_2_PPMe_3_]^+^ studied as it shows no evidence for P–P homolytic dissociation. In all other derivatives, heterolytic and homolytic P–P fission were detected, providing rare experimental evidence of both cleavage modes operating for the same bond within a compound. As predicted, [Me_2_PPMe_3_]^+^ undergoes P–P homolysis preferentially (by 15 kJ mol^–1^)^10^ over heterolysis. However, for all other derivatives, the experimental data indicate that heterolysis is preferred. The curves in Fig. 3 exhibit an increasing trend of R = Me < Et ≈ ^*i*^Pr ≈ Cy < ^*t*^Bu for P–P homolysis, and the trend Ph < Cy < Et ≈ ^*i*^Pr < ^*t*^Bu < Me for P–P heterolysis. Curiously, the decreasing ease of homolytic cleavage in diphosphines, C_6_H_6_ > CH_3_ > C_2_H_5_ > *n*-C_3_H_7_ > *n*-C_4_H_9_, parallels that observed for heterolysis in [R_2_PPMe_3_]^+^ cations. In contrast to previous computational work^10^ showing a general preference for homolytic P–P fission irrespective of molecular charge, these experimental results show that preference for homolysis is sensitive to variations in the substitution pattern. The viability of both fission modes for the P–P bond suggests significant charge delocalization within these complexes, which is further consistent with the observation of both heterolytic and homolytic P–C fission at the trivalent phosphorus for all derivatives except R = Ph, where detectable (see Table 1 and Fig. S19†). Observation of heterolytic P–C fission from the tetravalent phosphorus, giving [Me]^+^, is precluded by the small *m*/*z* of this fragment with respect to detection limits. Interestingly, homolytic P–C fission at the tetravalent phosphorus is only observed as a secondary process following homolytic P–C fission at the trivalent phosphorus, producing the fragment [RPPMe_2_]^+^ for the substitution patterns R = Et, ^*i*^Pr and ^*t*^Bu.

The complexity of fragmentation data for R = Me results from the fact that multiple processes may lead to fragments of differing connectivity or electronic structure, but equivalent *m*/*z*. For example, the peak observed at *m*/*z* 107 could not be assigned unambiguously because the fragments expected from successive P–C homolysis from either or both phosphorus centers have the same empirical formulae (*i.e.* [P^2^P^1^Me_3_]^+^, [MeP^2^P^1^Me_2_]^+^ and [Me_2_P^2^P^1^Me]^+^, using the atom numbering given in Scheme 2). Low mass fragments such as those at *m*/*z* 75, *m*/*z* 61, and *m*/*z* 59 appear simultaneously in the spectra of all phosphinophosphonium cations that exhibited P–P homolysis and are assigned as derivatives of [PMe_3_]^+^˙. Consistently, fragments of the same *m*/*z* were also observed in an electron impact study^13^ of neutral PMe_3_.

Formation of the primary and secondary β-hydride elimination products [R(H)PPMe_3_]^+^ and [H_2_PPMe_3_]^+^ is observed for all phosphinophosphonium cations containing R groups with β-hydrogen atoms (*i.e.* R = Et, ^*i*^Pr, ^*t*^Bu, Cy). The fragmentation data presented in Fig. 1 (and additionally Fig. S19d in the ESI†) indicate that β-hydride elimination, which yields extremely rare examples of H-phosphinophosphonium cations, is in fact the most preferred dissociation pathway for R = ^*t*^Bu in the gas phase as determined from the appearance potential and intensity of the resulting fragments. The observation of [Cy(H)PPMe_3_]^+^ by NMR spectroscopy^30^ and the recent isolation of NHC-stabilized phosphenium cations^31^ of the form [R(H)P]^+^ (R = H, Me, or CPh_3_) provide experimental evidence for the stability of [R(H)PPR′_3_]^+^ cations (R, R′ = alkyl or aryl) and supports the proposed β-hydride elimination pathway. Interestingly, β-hydride elimination is ubiquitous in transition metal coordination chemistry but has been found only rarely in main group complexes.^32^

**Fig. 4:**
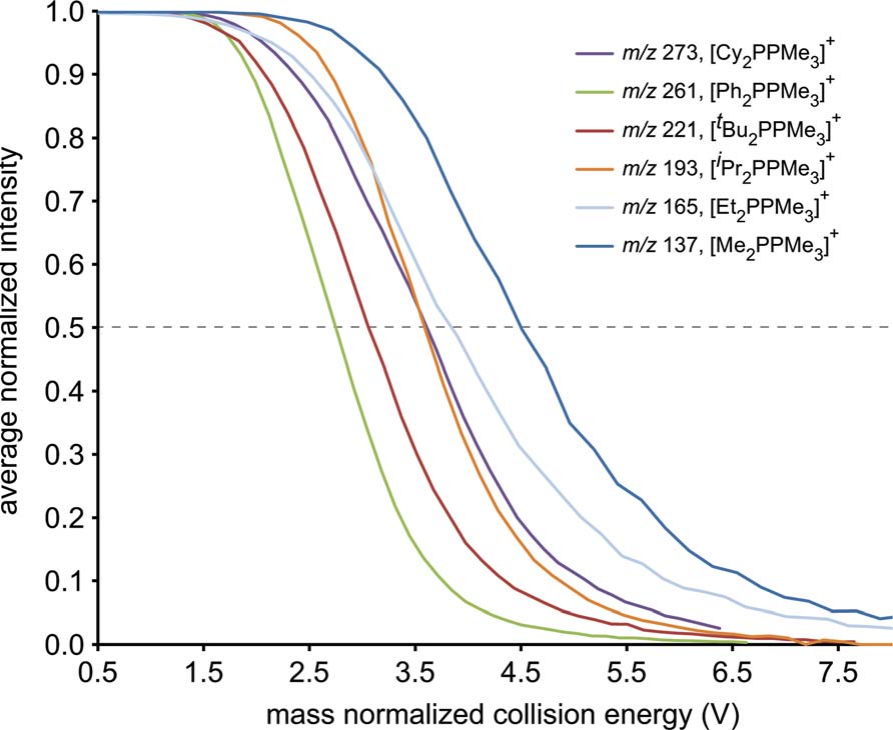
The decay of [R_2_PPMe_3_]^+^ cations (R = Me, Et, ^*i*^Pr, ^*t*^Bu, Cy, Ph) in terms of average normalized intensities with increasing mass normalized collision energy. Dashed line indicates 50% disappearance.

The resistance of the studied phosphinophosphonium cations towards all forms of decomposition is indicated by the order of increasing collision energy required for disappearance of [R_2_PPMe_3_]^+^ molecular ions (Fig. 4). By comparing the mass normalized collision voltage required to fragment a given phosphinophosphonium cation to 50% of its initial intensity,^33^ we surmise that the robustness of [R_2_PPMe_3_]^+^ increases in the order R = Ph < ^*t*^Bu < ^*i*^Pr ≈ Cy < Et < Me. The apparent inverse correlation between robustness and steric bulk (at the carbon bound to the trivalent phosphorus) for the subset of alkyl substituents is supported by the similar mass normalized collision energies of [Cy_2_PPMe_3_]^+^ and [^*i*^Pr_2_PPMe_3_]^+^ at 50% intensity. We therefore conclude that the trend in robustness depends on both the electronic and steric nature of the substituents at the tricoordinate phosphorus centre, and is defined by facile P–P heterolysis for a π-donor (R = Ph), facile β-hydride elimination for bulky alkyl substituents (R = ^*t*^Bu) and relative robustness for small alkyl substituents where P–P heterolysis is disfavoured and β-hydride elimination is not possible (R = Me).

**Fig. 5:**
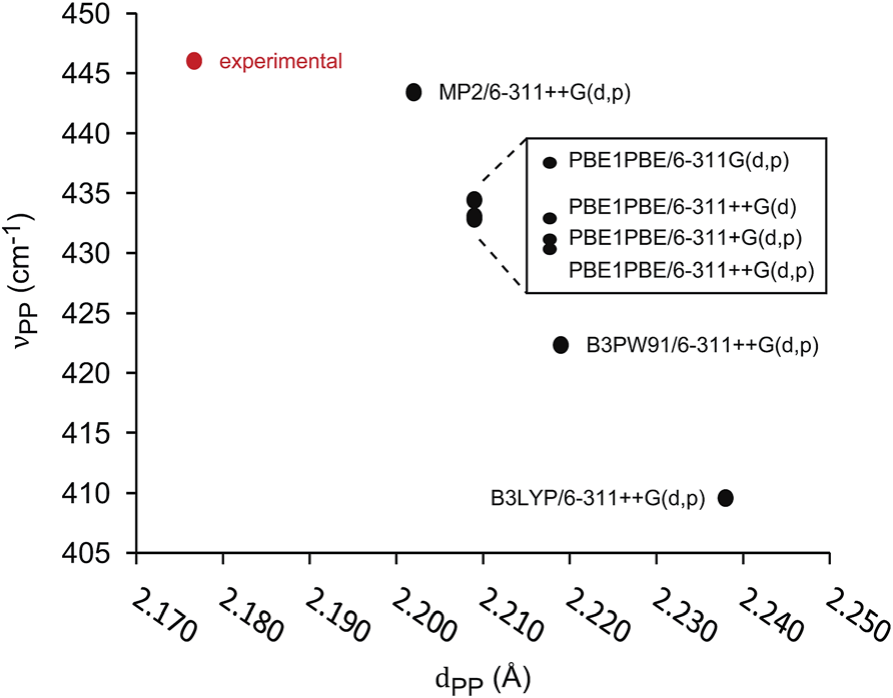
Correlation of calculated P–P stretching frequency (*ν*_PP_) and bond length (*d*_PP_) in benchmarking of functionals and basis sets for [Me_2_PPMe_3_]^+^.

The unimolecular gas-phase conditions inherent in our mass spectrometric experiments are well suited for comparison with predictions from computational chemistry. A benchmarking study of DFT functionals and basis sets was performed using the experimentally known P–P stretching frequency (*ν*_PP_ = 446 cm^–1^) and bond length (*d*_PP_ = 2.1767(6) Å) of [Me_2_PPMe_3_]^+^.^10^ The functionals investigated were selected based on previous use on related systems.^10^,^34^ As shown in Fig. 5 the functional used has a substantial influence over the calculated values of *ν*_PP_ and *d*_PP_ while the choice of basis set alters only the calculated value of *ν*_PP_. The PBE1PBE functional was selected as a compromise between accuracy of theoretical correlates and computational efficiency. Gibbs reaction energies determined from PBE1PBE/6-311++G(d,p) frequency analysis of fragments from the parent cation [Et_2_PPMe_3_]^+^ exhibit a trend that is mirrored by reaction energies calculated using single point energies from MP2/6-311++G(d,p) optimization (see Fig. S22†). In computational studies of diphosphines the inclusion of dispersion correction is reportedly critical to the determination of P–P homolytic dissociation energies.^35^ We have considered dispersion corrections through use of Grimme's DFT-D3 correction^36^ in PBE1PBE/6-311++G(d,p) optimization and frequency analysis of the phosphinophosphoniums [Et_2_PPMe_3_]^+^ and [^*t*^Bu_2_PPMe_3_]^+^. In both cases, Gibbs reaction energies for the modelled processes increased (*Δ* = 12–19 kJ mol^–1^ for R = Et, *Δ* = 27–30 kJ mol^–1^ for R = ^*t*^Bu) upon inclusion of dispersion effects, however these changes did not alter the calculated trends (see Fig. S22 and S23†).

The series of phosphinophosphonium cations [R_2_PPMe_3_]^+^ (R = Me, Et, ^*i*^Pr, ^*t*^Bu, and Ph), and fragments resulting from the mass spectrometrically observed processes were modelled and Gibbs energies of reaction were obtained using Hess's law. Correlation of *ν*_PP_ and *d*_PP_ values for the modelled phosphinophosphonium cations indicates that that there is no obvious relationship between *ν*_PP_ and *d*_PP_ and that the interchangeable use of *ν*_PP_ and *d*_PP_ in descriptions of P–P bond characteristics is unreliable (see Fig. S21†). Comparison of *d*_PP_ and *ν*_PP_ with calculated P–P homolysis and heterolysis energies shows that only *d*_PP_ is correlated with P–P bond energies (see Fig. S26†).

**Fig. 6:**
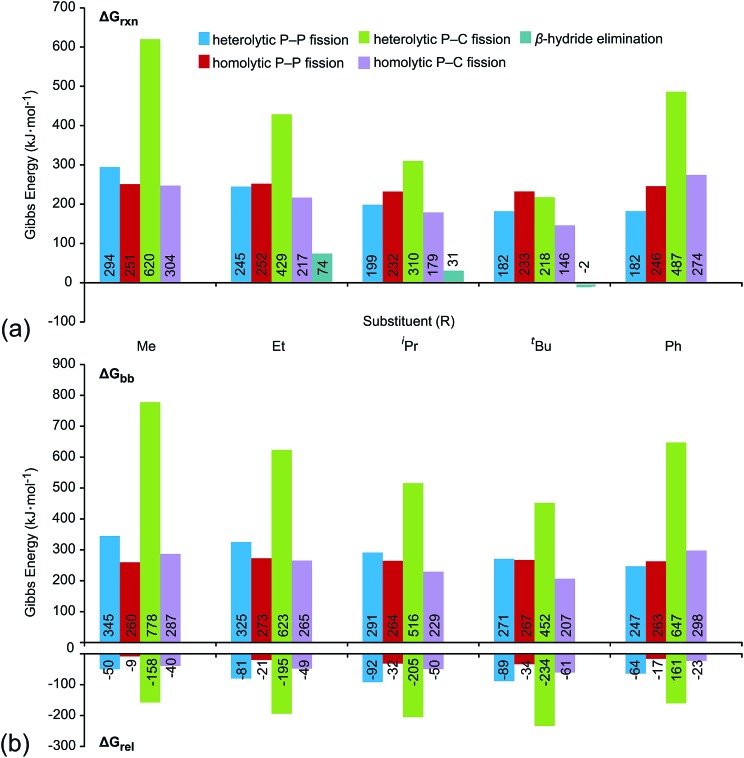
(a) Gibbs energies of reaction (Δ*G*_rxn_) for dissociation processes of [R_2_PPMe_3_]^+^ modelled in the gas-phase (298 K) at the PBE1PBE/6-311++G(d,p) level. See Scheme 2 for process definitions. (b) Decomposition of Δ*G*_rxn_ into bond break (Δ*G*_bb_) and fragment relaxation (Δ*G*_rel_) Gibbs energies. All values given in kJ mol^–1^.

Fig. 6a shows the trends in Gibbs energies of reaction (Δ*G*_rxn_) for P–P fission, P–C fission, and β-hydride elimination for the series of modelled phosphinophosphonium cations. In Fig. 6b, Δ*G*_rxn_ has been decomposed into a bond break process (Δ*G*_bb_, endothermic), corresponding to bond cleavage with retention of the fragment geometry observed in the bound complex, and a relaxation process (Δ*G*_rel_, exothermic), corresponding to the relaxation of the fragments. The overall Δ*G*_rxn_ values for both heterolytic and homolytic P–C fission from the trivalent phosphorus center vary according to the well-established trends in increasing stability for carbocations and carbon radicals, respectively, due to enhanced hyperconjugation with increasingly bulky substituents.^37^ As a result, the energy differences between the P–P and P–C fission processes decreases with increasing steric bulk and both are readily accessible for R = ^*t*^Bu. As is evident in Fig. 6a, the Δ*G*_rxn_ energies of all pathways appear to converge with increasing steric bulk (*cf.* R = Me and ^*t*^Bu). Δ*G*_rxn_ energies of P–C homolysis and heterolysis from the tetravalent phosphorus center are significantly greater than the respective values for the trivalent phosphorus (see Tables S10 and S11 in the ESI†) and have therefore been excluded from Fig. 6.

The calculated Δ*G*_rxn_ and Δ*G*_bb_ energies for P–P heterolysis follow the order R = Ph ≈ ^*t*^Bu < ^*i*^Pr < Et < Me and exhibit a large range (112 kJ mol^–1^ for Δ*G*_rxn_, 98 kJ mol^–1^ for Δ*G*_bb_, Table S6†) whereas the range calculated for P–P homolysis energies vary only slightly (20 kJ mol^–1^ for Δ*G*_rxn_, 10 kJ mol^–1^ for Δ*G*_bb_, Table S8†). Stabilization of phosphenium cations [R_2_P]^+^ for R = ^*t*^Bu and Ph by hyperconjugation and π-donation, respectively, likely accounts for facile heterolytic P–P cleavage in [^*t*^Bu_2_PPMe_3_]^+^ and [Ph_2_PPMe_3_]^+^. Consistent with the proposal that phosphenium stability is the key determinant of P–P heterolysis energies, Δ*G*_rxn_ values for P–P heterolysis show a linear dependence upon the ionization energies of neutral phosphinyl radicals R_2_P˙ (*r*^2^ = 0.99, see Fig. S25†).

Table 2 lists the most favourable dissociation pathway (earliest onset) for each phosphinophosphonium cation according to experimental observations and according to calculated values of Δ*G*_rxn_ and Δ*G*_bb_. While variations in the calculated Δ*G*_rxn_ energies and observed appearance potentials as a function of substitution are in broad agreement for a given process, as described for P–P homolysis, heterolysis and β-hydride elimination, the fragmentation process calculated to be most favourable is not consistently detected experimentally as having the lowest appearance potential. For example, although β-hydride elimination is predicted by the Δ*G*_rxn_ values to be most the accessible process for all substitutions (except R = Me and Ph), a significant preference for P–P and P–C fission is observed experimentally for most derivatives of [R_2_PPMe_3_]^+^. Considering the significance of kinetic barriers in the non-equilibrium conditions of the experiment, the process observed to be most favourable by mass spectrometry is expected to show greater correlation with Δ*G*_bb_ predictions, which represents the kinetic barrier for unimolecular bond dissociation, than with Δ*G*_rxn_, which represents the overall thermodynamic favourability of the process and includes the exothermic relaxation of the dissociated fragments. Consistently, the experimentally observed decomposition preferences are well-represented by Δ*G*_bb_ (Table 2) with the exception of R = ^*t*^Bu, for which a comparison cannot be made since a meaningful Δ*G*_bb_ cannot be calculated for the most favourable process (β-hydride elimination) because a P–H bond is formed concomitantly with a P–C bond cleavage. We therefore resorted to transition state calculations to model this process for the R = Et, ^*i*^Pr and ^*t*^Bu derivatives.

**Table 2:** Experimentally and computationally (Δ*G*_rxn_, Δ*G*_bb_) preferred dissociation pathways for derivatives of [R_2_PPMe_3_]^+^

R	Experiment (lowest appearance potential)	Calculated (lowest Δ*G*_rxn_)	Calculated (lowest Δ*G*_bb_)
Me	Homolytic P–P fission	Homolytic P–P fission	Homolytic P–P fission
Et	Homolytic P–C & heterolytic P–P fission^*a*^	β-Hydride elimination	Homolytic P–C fission
^*i*^Pr	Homolytic P–C & heterolytic P–P fission^*a*^	β-Hydride elimination	Homolytic P–C fission
^*t*^Bu	β-Hydride elimination	β-Hydride elimination	Homolytic P–C fission
Cy	Heterolytic P–P fission	^*b*^	^*b*^
Ph	Heterolytic P–P fission	Heterolytic P–P fission	Heterolytic P–P fission

^*a*^The traces of the two processes are almost identical in terms of intensity with increasing collision energy (see Fig. S18b and c).

^*b*^Not computed.

**Fig. 7:**
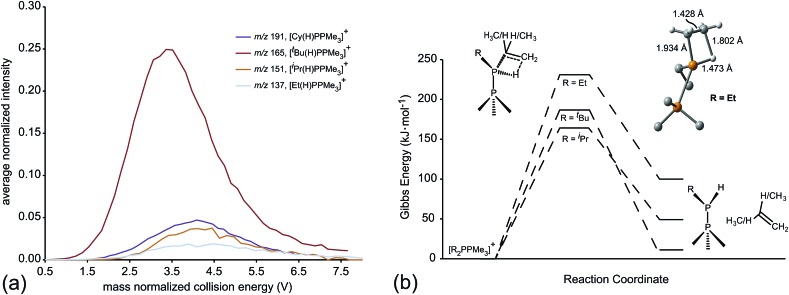
(a) The average normalized intensities [R(H)PPMe_3_]^+^ fragments formed through β-hydride elimination as a function of mass normalized collision energy. (b) Calculated (PBE1PBE/6-311++G(d,p)) reaction coordinate for β-hydride elimination from [R_2_PPMe_3_]^+^ (R = Et, ^*i*^Pr, ^*t*^Bu) and view of the calculated β-hydride transition state for [Et_2_PPMe_3_]^+^.

Of the processes represented in Fig. 6a, β-hydride elimination is calculated to be the most thermodynamically preferred decomposition pathway for R = Et, ^*i*^Pr and ^*t*^Bu in [R_2_PPMe_3_]^+^. Experimentally, β-hydride elimination is not observed for R = Me and Ph, and is observed as the most preferred pathway for R = ^*t*^Bu. The ^*i*^Pr and Cy-substituted phosphinophosphonium cations do not exhibit β-hydride elimination as the most preferred process, but it nevertheless occurs following the dominant P–P heterolytic process in each case (see Fig. S19†). For [Et_2_PPMe_3_]^+^, the experimental onset of β-hydride elimination is detected only after several other fragmentation processes. Therefore the trend in observed extent of β-hydride elimination is ^*t*^Bu > ^*i*^Pr ≈ Cy > Et (see Fig. 7a). We calculated transition states for β-hydride elimination in derivatives of [R_2_PPMe_3_]^+^ (R = Et, ^*i*^Pr, and ^*t*^Bu, Fig. 7b) and found them to resemble the classic four-membered transition state for the analogous process observed in organometallic complexes.^38^ The calculated activation energies were found to be 164 kJ mol^–1^ (R = ^*i*^Pr), 187 kJ mol^–1^ (R = ^*t*^Bu) and 229 kJ mol^–1^ (R = Et), and do not show a simple correlation with the degree of substitution in R for derivatives of [R_2_PPMe_3_]^+^. Interestingly, despite the formation of a strained alkene upon β-hydride elimination, the R = Cy derivative follows this decomposition pathway at an appearance potential comparable to that of R = ^*i*^Pr. Attempts to observe β-hydride elimination in bulk samples of [^*t*^Bu_2_PPMe_3_][OTf] as a solid, in MeCN, or in DMF were unsuccessful. We conclude that the absence of solvent and counterion in the mass spectrometric experiment establish a unique environment that is essential for detecting this process.

## Conclusions

The decomposition pathways of phosphinophosphonium cations [R_2_PPMe_3_]^+^ (R = Me, Et, ^*i*^Pr, ^*t*^Bu, Cy, Ph, N^*i*^Pr_2_) by collision-induced dissociation are diverse in terms of the number and complexity of processes observed. In many cases, the anticipated heterolytic and homolytic P–P cleavage processes were preceded by unexpected processes such as P–C fission and β-hydride elimination. The energy required for P–P homolysis in derivatives of [R_2_PPMe_3_]^+^ shows the trend R = Me < Et ≈ ^*i*^Pr ≈ Cy < ^*t*^Bu and no evidence for homolysis was observed in the case of R = Ph. For R = Me, homolysis is preferred over heterolysis in terms of appearance potentials, as previously predicted in a computational study.^10^ For all other substitution patterns, heterolysis was observed to occur at lower appearance potentials than homolysis. The energy required for P–P heterolysis shows the trend R = Ph < Cy < Et < ^*i*^Pr ≈ ^*t*^Bu < Me, and the variation in appearance potentials for heterolysis is discernibly greater than for homolysis. The simultaneous detection of heterolytic and homolytic P–P fission pathways in a single compound as reported in this work is rare. The relative chemical robustness of these cations is revealed by the disappearance order of parent ions with increasing collision energy to be R = Ph < ^*t*^Bu < ^*i*^Pr ≈ Cy < Et < Me. The behaviour of [(N^*i*^Pr_2_)_2_PPMe_3_]^+^ in ESI-MS and ESI-CID-MS/MS experiments is not yet understood.

Thermochemical data for P–P fission, P–C fission, and β-hydride elimination modelled at the PBE1PBE/6-311++G(d,p) level indicate that Δ*G*_rxn_ values for P–P heterolysis are influenced by the substituents, whereas Δ*G*_rxn_ requirements for homolysis do not vary significantly, as observed experimentally and as paralleled in Δ*G*_bb_ energies. The processes found experimentally to be the most favourable show good correlation with predictions from Δ*G*_bb_ considerations. A significant correlation is evident between calculated Gibbs energies of reaction and *d*(PP), in contrast to Gibbs energies of reaction and *ν*(PP) for derivatives of cations [R_2_PPMe_3_]^+^, where R = Me, Et, ^*i*^Pr, ^*t*^Bu, and Ph. No correlation was found to exist between calculated values of *ν*(PP) and *d*(PP) for this series.

The observation of β-hydride elimination from a phosphorus center represents unique behaviour for phosphinophosphonium cations and a rare mode of reactivity for main group coordination compounds in general. The calculated thermodynamic facility and experimentally observed preference for this process increase with degree of substitution in R for derivatives of [R_2_PPMe_3_]^+^. The existence of H-phosphinophosphonium cations has been recently evidenced by NMR spectroscopy^27^ and X-ray diffraction,^28^ suggesting that β-hydride elimination may be accessible in solution.

**Scheme 4:**
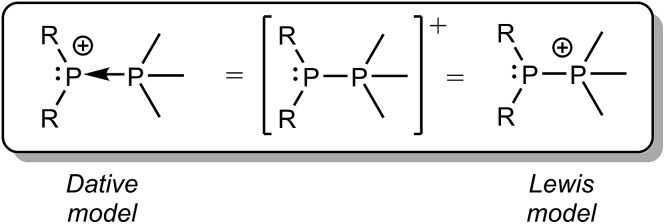
Dative and Lewis model representations of the generic phosphinophosphonium cation [R_2_PPMe_3_]^+^.

We have previously described P–P bonding in phosphinophosphonium cations using both Lewis and dative bonding models^39^ (Scheme 4), which localize the positive charge at the tetravalent and trivalent phosphorus centers, respectively. However, the unprecedented observation in this work of both P–P fragmentation pathways under conditions that are unbiased towards either implies that the exclusive use of either charge localizing model is an over-simplification that discounts the delocalization of the positive charge over the molecular framework. Consequently, the energy difference between Δ*G*_rxn_ for homolytic and heterolytic cleavage of any bond within a monocation (*e.g.* Δ = 24 kJ mol^–1^ for the P–P bond in [Me_2_PPMe_3_]^+^) is predicted to be substantially smaller than that in a corresponding neutral molecule (*e.g.* Δ = 739 kJ mol^–1^ for the P–P bond in Me_2_PPMe_2_),^10^ particularly when the elements in the bond have comparable electronegativities. This realisation will inform synthetic strategies by inspiring new radical coupling routes to E–E monocations.

## Supplementary Material

Supplementary information
