# Supplementary material for: Bond fission in monocationic frameworks: diverse fragmentation pathways for phosphinophosphonium cations
Source: Chem Sci. 2016 Jan 5;7(4):2544–52. doi: 10.1039/c5sc03804a (PMC5477047; doi:10.1039/c5sc03804a)
Supplement: Supplementary file 1 [file SC-007-C5SC03804A-s001.pdf]

Electronic Supplementary Material (ESI) for Chemical Science.
This journal is © The Royal Society of Chemistry 2016

Bond Fission in Monocationic Frameworks: Diverse Fragmentation Pathways for Phosphinophosphonium Cations

Karlee L. Bamford,[a] Saurabh S. Chitnis,[a] Rhonda L. Stoddard,[a] J. Scott McIndoe*[a] and Neil Burford*[a]

[a]*Department of Chemistry, University of Victoria, P.O. Box 3065, Stn CSC, Victoria, BC, V8W 3V6, Canada*

Supporting Information

Contents

Synthesis & Characterization S4
Mass Spectrometry S15
  Sample Calculation for Correction of Intensities Due to M+1 Peaks S32
Quantum Chemical Calculations S33
  Dissociation Trends S34
  Reaction Enthalpies and Gibbs Energies[α] S36
  Benchmarking S40
    Level of Theory S40
    Basis Set S41
  HF Optimizations and Frequency Analysis S42
    $[R_2PPMe_3]^+$ S42
    $[R(H)PPMe_3]^+$ S44
    $[R_2PPMe_2]^{+\cdot}$ S46
    $[R_2PPMe_2]$ S48
    $[RPPMe_3]^{+\cdot}$ S50
    $[RPPMe_3]$ S51
    $[RPPMe_2]^+$ S53
    $[R_2P]^+$ S55
    $[R_2P]^\cdot$ S56
    $[R]^+$ S58
    $[R]^\cdot$ S59

Alkanes S60
Olefins S61
$[PMe_3]$ Derivatives S62
Miscellaneous fragments S63
PBE1PBE Optimizations, Frequency Analysis and MP2 Single Point Calculations S65
$[R_2PPMe_3]^+$ S65
$[R(H)PPMe_3]^+$ S67
$[R_2PPMe_2]^{+\cdot}$ S69
$[R_2PPMe_2]$ S71
$[RPPMe_3]^{+\cdot}$ S73
$[RPPMe_3]$ S75
$[RPPMe_2]^+$ S77
$[R_2P]^+$ S78
$[R_2P]^\cdot$ S80
$[R]^+$ S82
$[R]^\cdot$ S83
Alkanes S84
Olefins S86
$[PMe_3]$ Derivatives S86
Miscellaneous fragments S87
Dispersion Corrected PBE1PBE Optimizations and Frequency Analysis S89
$[R_2PPMe_3]^+$ S89
$[R(H)PPMe_3]^+$ S90
$[RPPMe_3]^{+\cdot}$ S91
$[RPPMe_3]$ S92
$[RPPMe_2]^+$ S92
$[R_2P]^+$ S93
$[R_2P]^\cdot$ S93
$[R]^+$ S94
$[R]^\cdot$ S94
Alkanes S95
Olefins s95

[PMe$_3$] Derivatives S95

PBE1PBE Frequency Analysis of Distorted Geometries S96

[R$_2$PPMe$_2$]$^{+\cdot}$ S96

[R$_2$PPMe$_2$] S98

[RPPMe$_3$]$^{+\cdot}$ S100

[RPPMe$_3$] S102

[PMe$_3$]$^{+\cdot}$ S103

[PMe$_3$] S105

[R$_2$P]$^{+}$ S106

[R$_2$P]$^{\cdot}$ S107

[R]$^{+}$ S109

[R]$^{\cdot}$ S110

[Me]$^{+}$ S111

[Me]$^{\cdot}$ S112

PBE1PBE Optimizations and Frequency Analysis of β-Hydride Elimination Transition States and Products S113

Transition States (TS) S113

[R(H)PPMe$_3$]$^{+}$ and alkene S114

MP2 Optimizations S115

[R$_2$PPMe$_3$]$^{+}$ S115

[R(H)PPMe$_3$]$^{+}$ S116

[RPPMe$_3$]$^{+\cdot}$ S117

[RPPMe$_3$] S117

[RPPMe$_2$]$^{+}$ S118

[R$_2$P]$^{+}$ S120

[R$_2$P]$^{\cdot}$ S121

[R]$^{+}$ S122

[R]$^{\cdot}$ S123

Alkanes S124

Olefins S125

[PMe$_3$] Derivatives S125

Miscellaneous fragments S126

References S127

Synthesis & Characterization

All compounds studied by ESI-MS/MS were prepared in an inert atmosphere of dry (H_2O < 0.5 ppm) and deoxygenated (O_2 < 0.5 ppm) nitrogen, using glovebox techniques. Chlorophosphines R_2PCl, where R = Me, Et, ^{i}Pr, ^{t}Bu, and N^{i}Pr$_2$, were purchased from Sigma Aldrich. Solvents were stored over activated 3 Å molecular sieves following drying procedures. Acetonitrile (MeCN) was obtained from Sigma Aldrich and distilled from CaH_2. Anhydrous N, N-dimethylformamide (DMF) was obtained from Sigma Aldrich and used as received. Ether (Et_2O) and hexanes were obtained from an MBraun Solvent Purification System, and Et_2O was subsequently dried by distillation from NaK alloy. Deuterated solvents (acetone-d6, MeCN-d3) were obtained from Sigma Aldrich. NMR spectra were collected at 298 K on Bruker Avance 300, 360 or 500 MHz spectrometers in 5 mm diameter tubes. ^{1}H and ^{13}C NMR chemical shifts are reported relative to protio-solvent signals, while ^{31}P and ^{19}F NMR chemical shifts are reported relative to H_3PO_4 and $CFCl_3$ external standards, respectively. FT-IR spectra were recorded at 298 K in an inert atmosphere of nitrogen on a Perkin Elmer Frontier instrument equipped with a diamond ATR unit. Band intensities are described as very weak (vw), weak (w), medium (m), strong (s), very strong (vs), or broad (br).

The following general preparative procedure was used, as reported previously[1,2]: R_2PCl (1.00 mmol) and PMe_3 (1.00 mmol) were stirred separately in 1-2 mL of MeCN at room temperature until the solutions appeared homogenous and clear (generally colourless). Slow addition of the PMe_3 solution by pipette caused immediate precipitation of white solids. Reaction mixtures were typically stirred for 15-20 minutes and remained suspensions throughout. The solids were allowed to settle and the supernatant was decanted by pipette. The crude products were then washed with three small volumes (~ 0.5 mL) of diethyl ether and/or hexanes and volatiles were removed in vacuum. Conversion of the chloride salts to triflates was achieved *via* addition of 1 equivalent trimethylsilyl triflate (TMSOTf) in 1-2 mL MeCN to reaction mixtures of the respective phosphinophosphonium chloride salts. Addition of the TMSOTf produced clear and colourless solutions, which were stirred for 15-20 minutes, dried *in vacuo* to give white solids, and washed as described above. NMR spectra of crude reaction mixtures were recorded using acetone-d6 capillaries, while the spectra of solids, isolated following recrystallization at -30 ^{o}C from mixed solvent systems (*e.g.* MeCN/hexanes or MeCN/Et_2O), were recorded in MeCN-d3. Characterization data of previously unknown phosphinophosphoniums are reported herein with accompanying NMR spectra in Figures S1-S5. Tetracoordinate phosphorus centres (*i.e.* PMe_3) are designated P^1 and tricoordinate phosphorus centers (*i.e.* R_2P), P^2.

[Me_2PPMe_3][Cl] : Prepared and isolated according to reported procedure.2

[Me_2PPMe_3][OTf] : Hexanes used in place of MeCN. Me_2PCl (96.4 mg, 1.00 mmol), PMe_3 (77.3 mg, 1.02 mmol), TMSOTf (230.2 mg., 1.04 mmol); white powder; Yield: 138 mg, 80% (powder); ^{1}H NMR (500.3 MHz, 298 K, CD_3CN, δ [ppm]): 1.44 (6H, *doublet of doublets*, $^2J_{HP}$ = 18.7 Hz, $^3J_{HP}$ = 4.4 Hz, P(C*H*$_3$)$_2$), 1.86 (9H, *doublet of doublets*, $^2J_{HP}$ = 13.9 Hz, $^3J_{HP}$ = 3.2 Hz, P(C*H*$_3$)$_3$); ^{31}P NMR (202.5 MHz, CD_3CN, δ [ppm]): -59.6 (*broad doublet*, $^1J_{PP}$ = 275 Hz, P^2), 17.7 (*doublet of decets of multiplets*, $^1J_{PP}$ = 275 Hz, J = 18 Hz, P^1); $^{13}C\{^1H\}$ NMR (125.8 MHz, CD_3CN, δ [ppm]): 5.5 (*doublet of doublets*, $^1J_{CP}$ = 18 Hz, $^2J_{CP}$ = 3 Hz, P(*C*H$_3$)$_2$), 6.2 (*doublet of doublets*, $^1J_{CP}$ = 44 Hz, $^2J_{CP}$ = 7 Hz, P(*C*H$_3$)$_3$); $^{19}F\{^1H\}$ NMR (282.5MHz, CD_3CN, δ [ppm]): -79.1 (*singlet*, CF_3).

[Et_2PPMe_3][OTf] : Et_2PCl (259 mg, 2 mmol), PMe_3 152 mg, 2 mmol), TMSOTf (444 mg, 2 mmol); white powder; Yield:595 mg, 95% (powder); ^{1}H NMR (300.3 MHz, 298 K, CD_3CN, δ [ppm]): 1.23 (6H, *doublet of triplets*, $^3J_{HP}$ = 18.0 Hz, $^3J_{HH}$ = 7.7 Hz, P(CH$_2$C*H*$_3$)$_2$), 1.81-1.96 (4H, (*multiplet overlapping with neighbouring doublet of doublets*, P(C*H*$_2$CH$_3$)$_2$), 1.86 (9H, *doublet of doublets overlapping with neighbouring multiplet*, $^2J_{HP}$ = 13.8 Hz, $^3J_{HP}$ = 2.9 Hz, P(C*H*$_3$)$_3$); ^{31}P NMR (121.6 MHz, CD_3CN, δ [ppm]): -34.7 (*double of multiplets*, $^1J_{PP}$ = 289 Hz, P^2), 15.2 (*doublet of decets*, $^1J_{PP}$ = 288 Hz, $^2J_{PH}$ = 13 Hz, P^1); $^{13}C\{^1H\}$ NMR (75.5 MHz, CD_3CN, δ [ppm]): 7.5 (*doublet of doublets*, $^1J_{CP}$ = 43 Hz, $^2J_{CP}$ = 7 Hz, P(*C*H$_3$)$_3$), 10.4 (*doublet of doublets*, J_{CP} = 18 Hz, J_{CP} = 11 Hz, P(CH$_2$CH$_3$)$_2$), 13.2 (*doublet of doublets*, J_{CP} = 18 Hz, J_{CP} = 3 Hz, P(CH$_2$CH$_3$)$_2$); $^{19}F\{^1H\}$ NMR (282.5MHz, CD_3CN, δ [ppm]): -79.3 (*singlet*, CF_3).

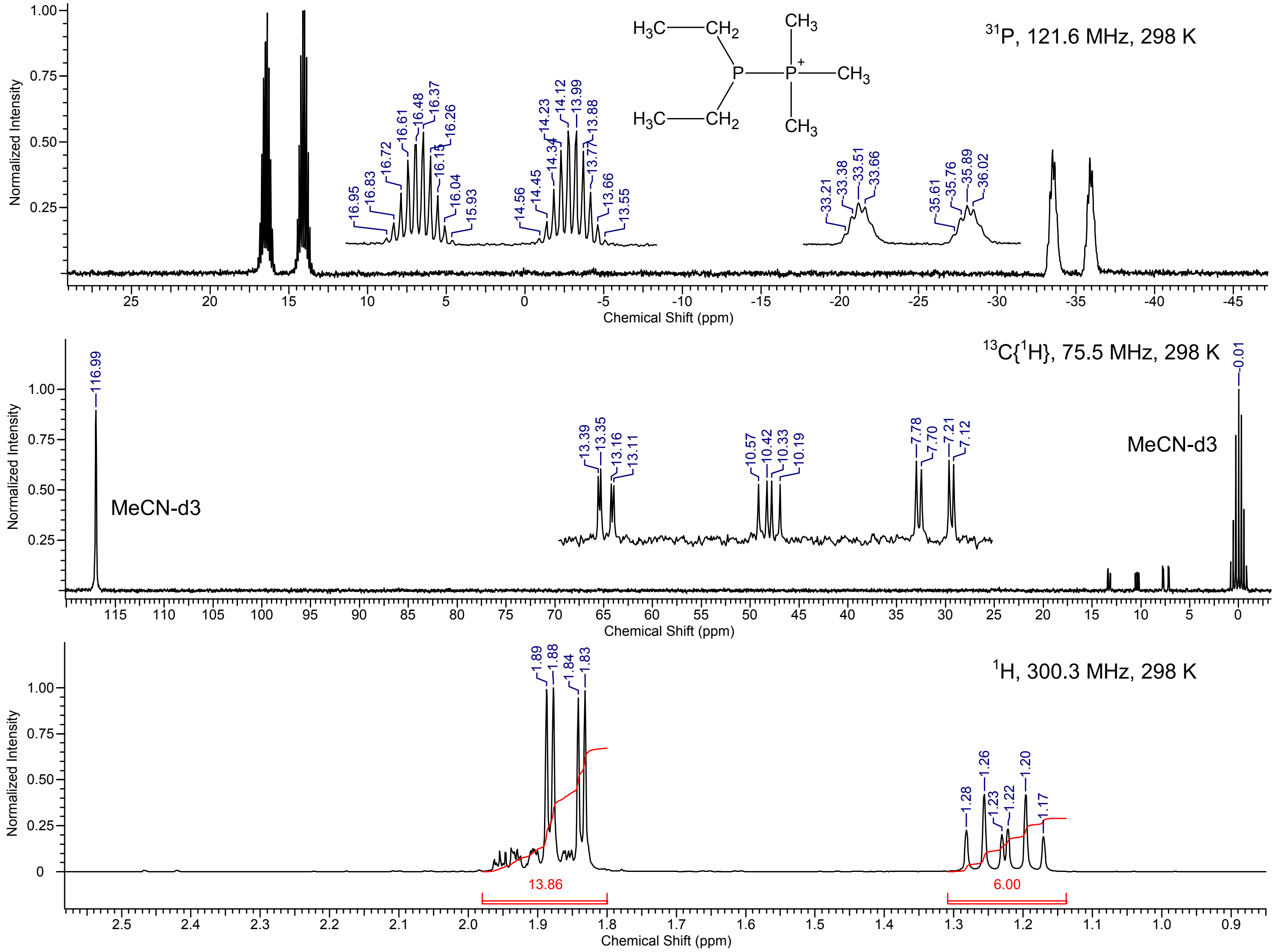

Figure S1. NMR spectra of $[Et_2PPMe_3][OTf]$.

[^{i}Pr$_2$PPMe$_3$][OTf] : Hexanes used in place of MeCN. ^{i}Pr$_2$PCl (152.9 mg, 1.00 mmol), PMe$_3$ (78.0 mg, 1.03 mmol), TMSOTf (225.0 mg, 1.01 mmol); white powder; Yield: 194 mg, 85% (powder); ^{1}H NMR (360.3 MHz, 298 K, CD$_3$CN, δ [ppm]): 1.31 (6H, *doublet of multiplets overlapping with neighbouring doublet of multiplets,* $^3J_{HP}$ = 15 Hz†, $^3J_{HH}$ = 7.0 Hz, CH(C*H*$_3$)(CH$_3$*)), 1.31 (6H, *doublet of multiplets overlapping with neighbouring doublet of multiplets*, $^3J_{HP}$ = 15 Hz†, $^3J_{HH}$ = 7.3 Hz, CH(CH$_3$)(C*H*$_3$*)), 1.96 (9H, *doublet of doublets*, $^2J_{HP}$ = 13.3 Hz, $^3J_{HP}$ = 2.6 Hz, P(C*H*$_3$)$_3$), 2.48 (2H, *apparent nonet of doublets*, J_{HH} = 7.2 Hz, $^2J_{HP}$ = 2.1 Hz, C*H*(CH$_3$)$_2$); ^{31}P NMR (145.8 MHz, CD$_3$CN, δ [ppm]): 1.21 (*broad doublet of apparent nonets*, $^1J_{PP}$ = 323 Hz, $^3J_{PH}$ = 15 Hz, J_{PH} = 2 Hz, P^2), 12.4 (*doublet of decets*, $^1J_{PP}$ = 323 Hz, $^2J_{PH}$ = 13 Hz, P^1); ^{13}C{^{1}H} NMR (90.6 MHz, CD$_3$CN, δ [ppm]); : 10.4 (*doublet of doublets*, , $^1J_{CP}$ = 42 Hz, $^2J_{CP}$ = 7 Hz, P(*C*H$_3$)$_3$), 20.1 (*doublet of doublets*, $^2J_{CP}$ = 12 Hz, $^3J_{CP}$ = 8 Hz, CH(*C*H$_3$)(CH$_3$*)), 21.3 (*doublet of doublets*, $^2J_{CP}$ = 16 Hz, $^3J_{CP}$ = 8 Hz, CH(CH$_3$)(*C*H$_3$*)), 22.0 (*doublet of doublets*, $^1J_{CP}$ = 21 Hz, $^2J_{CP}$ = 4 Hz, *C*H(CH$_3$)(CH$_3$*)); ^{19}F{^{1}H} NMR (282.5 MHz, CD$_3$CN, δ [ppm]) : -79.2 (*singlet*, CF$_3$); FT-IR (ATR, 298 K, [cm^{-1}]): 235 (vw), 244 (vw), 270 (vw), 317 (vw), 346 (vw), 420 (vw), 473 (vw), 515 (m), 572 (w), 607 (w), 635 (vs), 679 (vw), 753 (vw), 762 (vw), 862 (vw), 869 (w), 885 (vw), 958 (m), 1029 (s), 1105 (w), 1144 (s), 1221 (m), 1259 (vs), 1301 (w), 1320 (vw), 1369 (vw), 1385 (vw), 1423 (w), 1456 (vw), 1466 (vw), 2870 (vw), 2914 (vw), 2968 (w), 3000 (vw).

†This coupling constant could not be measured to one decimal place (see ^{1}H NMR data in Figure S2) as for other signals in the spectrum but is consistent with the $^3J_{PH}$ coupling constant observed for the P^2 center by ^{31}P NMR

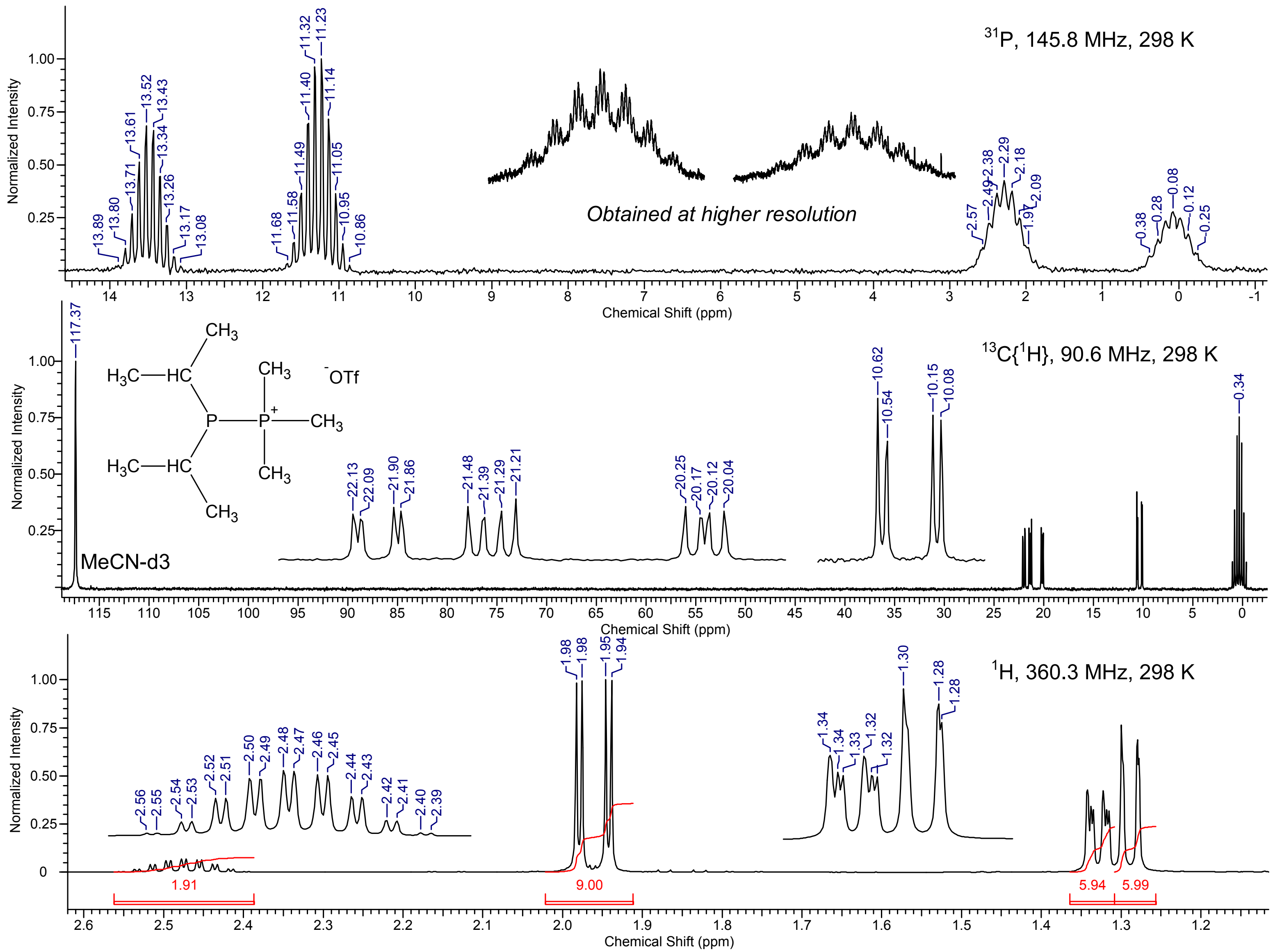

Figure S2. NMR spectra of $[^iPr_2PPMe_3][OTf]$.

[^{t}Bu$_2$PPMe$_3$][OTf] : ^{t}Bu$_2$PCl (181.8 mg, 1.01 mmol), PMe$_3$ (76.8 mg, 1.01 mmol), TMSOTF (230.0 mg, 1.03 mmol); white solid; Yield: 300.1 mg, 84.3% (powder); ^{1}H NMR (500.3 MHz, 298 K, CD$_3$CN, δ [ppm]): 1.46 (18H, *doublet of doublets*, $^3J_{HP}$ = 12.9 Hz, $^4J_{HP}$ = 1.0 Hz, C(C*H*$_3$)$_3$), 2.08 (9H, *doublet of doublets*, $^2J_{HP}$ = 13.1 Hz, $^3J_{HP}$ = 2.4 Hz, P(C*H*$_3$)$_3$); ^{31}P NMR (202.5 MHz, CD$_3$CN, δ [ppm]): 10.9 (*doublet of decets*, $^1J_{PP}$ = 382 Hz, $^2J_{PH}$ = 13 Hz, P^1), 42.1 (*doublet of multiplets*, $^1J_{PP}$ = 381 Hz, P^2); ^{13}C{^{1}H} NMR (125.8 MHz, CD$_3$CN, δ [ppm]): 13.6 (*doublet of doublets*, $^1J_{CP}$ = 42 Hz, $^2J_{CP}$ = 7 Hz, P(*C*H$_3$)$_3$), 31.0 (*doublet of doublets*, $^2J_{CP}$ = 13 Hz, $^3J_{CP}$ = 7 Hz, C(*C*H$_3$)$_3$), 36.2 (*doublet of doublets*, $^1J_{CP}$ = 30 Hz, $^2J_{CP}$ = 5 Hz, *C*(CH$_3$)$_3$); ^{19}F{^{1}H} NMR (282.5 MHz, CD$_3$CN, δ [ppm]) : -79.2 (s*inglet*, CF$_3$); FT-IR (ATR, 298 K, [cm^{-1}]): 236 (vw), 243 (vw), 255 (vw), 260 (vw), 270 (vw), 277 (vw), 284 (vw), 289 (vw), 311 (vw), 325(w), 335 (w), 364 (vw), 398 (w), 433 (vw), 446 (vw), 482 (w), 516 (m), 570 (m), 635 (vs), 768 (w), 754 (w), 805 (w), 859 (vw), 927 (w), 964 (s), 1000 (vw), 1028 (s), 1089 (vw), 1138 (s), 1167 (w), 1177 (vw), 1221 (m), 1252 (s), 1300 (w), 1322 (w), 1349 (vw), 1372 (w), 1421 (w), 1446 (vw), 1465 (w), 1476 (w), 2870 (vw), 2920 (w), 2952 (vw), 3000 (w).

[Cy$_2$PPMe$_3$]OTf: Prepared and isolated according to reported procedure.[3]

[(NMe$_2$)$_2$PPMe$_3$][OTf] : (NMe$_2$)$_2$PCl (155 mg, 1 mmol), PMe$_3$ (76 mg, 1 mmol), TMSOTf (222 mg, 1 mmol); white-yellow solid; Yield: 301 mg, 88 % (powder); ^{1}H NMR (300.3 MHz, 298 K, CD$_3$CN, δ [ppm]): 1.63 (9H, *doublet*, $^2J_{HP}$ = 13.2 Hz, P(C*H*$_3$)$_3$), 2.73 (12H, *doublet*, $^2J_{HP}$ = 10.2 Hz, N(C*H*$_3$)$_2$); ^{31}P NMR (121.6 MHz, CD$_3$CN, δ [ppm]): 0.9 (*broad doublet*, $^1J_{PP}$ = 316 Hz, P^1), 102.0 (*broad doublet*; $^1J_{PP}$ = 316 Hz, P^2); ^{13}C{^{1}H} NMR (75.5 MHz, CD$_3$CN, δ [ppm]); : 8.9 (*doublet*, $^1J_{CP}$ = 36 Hz, P(*C*H$_3$)$_3$), 42.7 (*doublet,* $^2J_{CP}$ = 20 Hz, N(*C*H$_3$)$_2$); ^{19}F{^{1}H} NMR (282.5 MHz, CD$_3$CN, δ [ppm]) : -79.3 (*singlet*, CF$_3$).

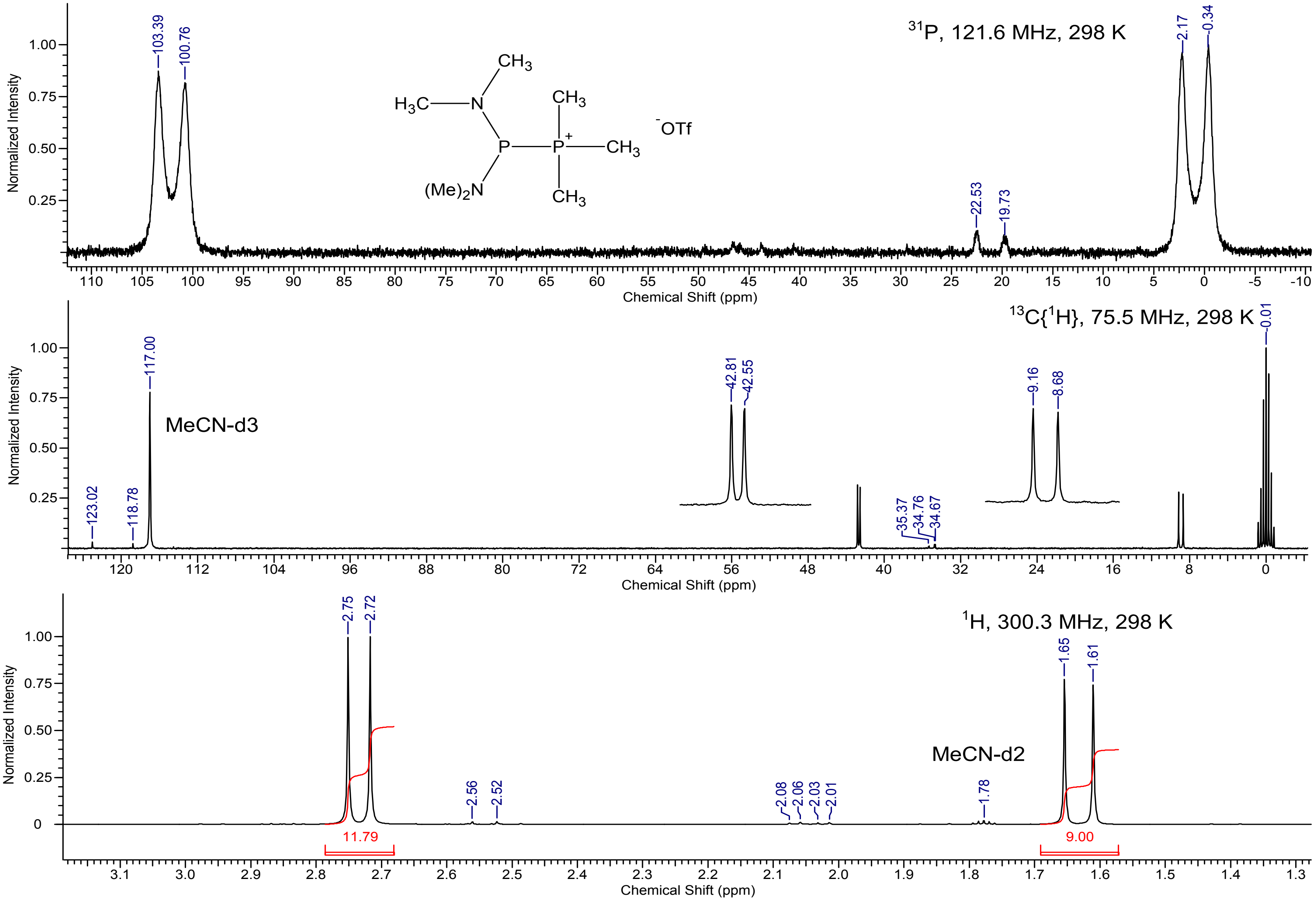

Figure S3. NMR spectra of $[(NMe_2)PPMe_3][OTf]$.

[(NEt$_2$)$_2$PPMe$_3$][OTf] : (NEt$_2$)$_2$PCl (211 mg, 1 mmol), PMe$_3$ (76 mg, 1 mmol), TMSOTf (222 mg, 1 mmol); white-yellow solid; Yield: 388 mg, 97 % (powder); ^{1}H NMR (300.3 MHz, 298 K, CD$_3$CN, δ [ppm]): 1.14 (12H, *triplet*, $^3J_{HH}$ = 7.2 Hz, CH$_2$C*H*$_3$), 1.80 (9H, *doublet*, $^2J_{HP}$ = 13.2 Hz, P(C*H*$_3$)$_3$), 3.24 (8H, *doublet of overlapping quartets*, $^2J_{HP}$ = 11.3 Hz, $^3J_{HH}$ = 7.1 Hz, C*H*$_2$CH$_3$); ^{31}P NMR (121.6 MHz, CD$_3$CN, δ [ppm]): -0.9 (*broad doublet*, $^1J_{PP}$ = 333 Hz, P^1), 93.5 (*broad doublet*; $^1J_{PP}$ = 333 Hz, P^2); ^{13}C{^{1}H} NMR (75.5 MHz, CD$_3$CN, δ [ppm]); : 9.1 (*doublet*, $^1J_{CP}$ = 39 Hz, P(*C*H$_3$)$_3$), 13.7 (*doublet,* $^3J_{CP}$ = 3 Hz, CH$_2$*C*H$_3$), 45.6 (*doublet*, $^2J_{CP}$ = 19 Hz, *C*H$_2$CH$_3$); ^{19}F{^{1}H} NMR (282.5 MHz, CD$_3$CN, δ [ppm]) : -79.3 (*singlet*, CF$_3$).

[(N^{i}Pr$_2$)$_2$PPMe$_3$][OTf] : (N^{i}Pr$_2$)$_2$PCl (266.2 mg, 1.00 mmol), PMe$_3$ (83.2 mg, 1.09 mmol), TMSOTf (333.5 mg, 1.50 mmol); white-yellow solid; Yield: 436.5 mg, 96 % (powder); ^{1}H NMR (360.2 MHz, 298 K, CD$_3$CN, δ [ppm]): 1.28 (26H, *broad apparent doublet*, CH(C*H*$_3$)$_2$), 1.86 (9H, *doublet*, $^2J_{HP}$ = 12.7 Hz, P(C*H*$_3$)$_3$), 3.65 (4H, *septet*, $^3J_{HH}$ = 6.4 Hz, C*H*(CH$_3$)$_2$); ^{31}P NMR (145.9 MHz, CD$_3$CN, δ [ppm]): -2.1 (*broad doublet of multiplets*, $^1J_{PP}$ = 359 Hz, P^1), 60.5 (*broad doublet*; $^1J_{PP}$ = 359 Hz, P^2); ^{13}C{^{1}H} NMR (90.6 MHz, CD$_3$CN, δ [ppm]); : 10.8 (*broad doublet*, $^1J_{CP}$ = 38 Hz, P(*C*H$_3$)$_3$), 23.7 (*broad apparent doublet,* CH(*C*H$_3$)$_2$), 51.0 (*broad singlet*, *C*H(CH$_3$)$_2$); ^{19}F{^{1}H} NMR (282.5 MHz, CD$_3$CN, δ [ppm]) : -79.2 (*singlet*, CF$_3$); FT-IR (ATR, 298 K, [cm^{-1}]): 302 (m), 352 (w), 426 (vw), 491 (m), 516 (s), 570 (m), 634 (vs), 667 (vw), 710 (vw), 753 (m), 789 (w), 845 (m), 870 (m), 953 (s), 975 (m), 1017 (s), 1028 (vs), 1057 (vw), 1113 (s), 1149 (vs), 1172 (m), 1199 (m), 1223 (s), 1253 (vs), 1296 (w), 1337 (vw), 1368 (m), 1393 (w), 1424 (vw), 1460 (w), 2869 (vw), 2936 (w), 2973 (m).

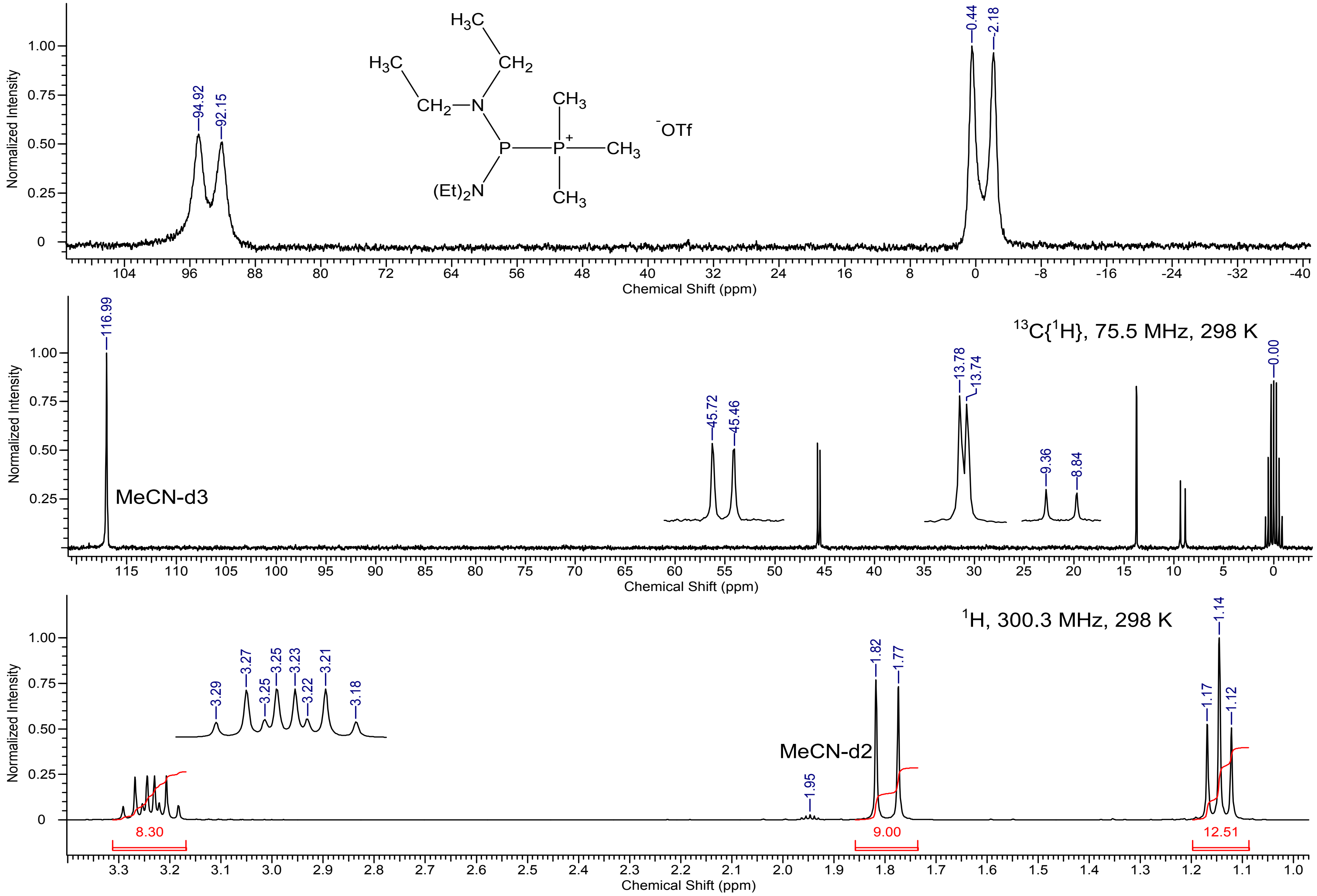

Figure S4. NMR spectra of $[(NEt_2)_2PPMe_3][OTf]$.

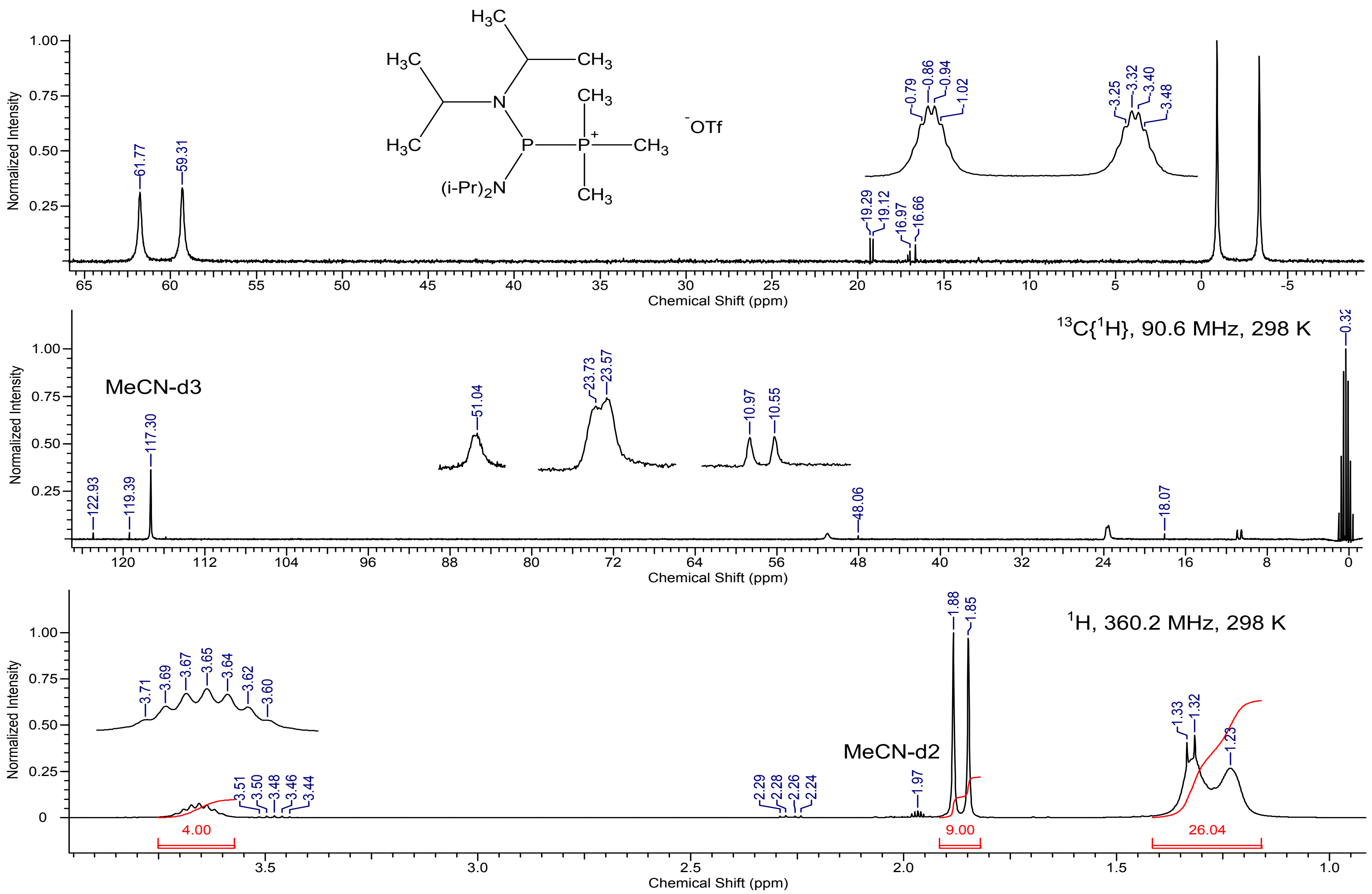

Figure S5. NMR spectra of $[(N^iPr_2)_2PPMe_3][OTf]$.

[Ph_2PPMe_3]OTf : Prepared and isolated according to published procedures.[4] ^1H NMR (360.3 MHz, 298 K, CD_3CN, δ [ppm]): 1.81 (9H, *doublet of doublets*, $^2J_{HP}$ = 13.7 Hz, $^3J_{HP}$ = 3 Hz, P(CH_3)$_3$), 7.67 (10H, *multiplet,* P($C_6$$H_5$)$_2$); ^{31}P NMR (145.8 MHz, CD_3CN, δ [ppm]): -23.7 (*broad doublet*, $^1J_{PP}$ = 297 Hz, P^2), 15.5 (*doublet of decets,* $^1J_{PP}$ = 293 Hz, $^2J_{PH}$ =14 Hz, P^2*)*; $^{13}C\{^1H\}$ NMR (90.6 MHz, CD_3CN, δ [ppm]): 8.49 (*doublet of doublets*, $^1J_{CP}$ = 44 Hz, P(*C*H_3)$_3$), 124.9 (*doublet of doublets*, $^1J_{CP}$ = 15 Hz, $^2J_{CP}$ = 4 Hz, C-ipso), 130.1 (*doublet of doublets*, J_{CP} = 9 Hz, J_{CP} = 2 Hz, C-ortho/meta), 132.0 (*broad doublet,* $^4J_{CP}$ = 5 Hz, C-para), 134.8 (*doublet of doublets*, J_{CP} = 22 Hz, J_{CP} = 7 Hz, C-ortho/meta); $^{19}F\{^1H\}$ NMR (282.5 MHz, CD_3CN, δ [ppm]) : -79.1 (*singlet*, CF_3); FT-IR (ATR, 298 K, [cm^{-1}]): 227 (m), 237 (vw), 245 (vw), 253 (vw), 261 (vw), 296 (w), 317 (vw), 328 (vw), 347 (vw), 373 (vw), 397 (w), 425 (w), 434 (m), 459 (w), 474 (m), 506 (s), 515 (m), 552 (w), 571 (m), 635 (vs), 694 (s),735 (m), 745 (m), 757 (s), 786 (vw), 854 (w), 956 (s), 998 (m), 1027 (vs), 1068 (vw), 1086 (w), 1147 (s), 1223 (s), 1252 (vs), 1299 (w), 1317 (vw), 1416 (w), 1436 (w), 1475 (w), 1581 (vw), 2909 (w), 2988 (w), 3065 (vw).

Mass Spectrometry

Collision-induced dissociation data were collected in the positive ion mode using a Waters Micromass Q-Tof *micro* (quadrupole time-of-flight) mass spectrometer. Stock solutions of phosphinophosphoniums (10 mM) were prepared in acetonitrile (Sigma-Aldrich, anhydrous grade, dried over 3 Å molecular sieves) and diluted to approximately 1 mM for analysis. Sample solutions were introduced to the instrument source *via* pressurized sample infusion using argon gas and a 39 cm length of PEEK (polyether ether ketone) tubing. Electrospray ionisation was achieved using a capillary voltage of 3000 V, a source temperature of 70 °C, and a desolvation temperature of 200 °C. Standard experimental parameters used were: extraction voltage, 0.5 V; sample cone voltage, 15 V; cone gas and desolvation gas flow rates of 100 L/hr. Instrument calibration was completed with a 3.3 mM sodium iodide solution in methanol prior to mass spectrometric analysis. A collision voltage of 2 V was generally used in real time analysis of sample solutions in MS mode for the identification of major species prior to selection in MS/MS mode and subsequent collision-induced dissociation. The high mass and low mass resolutions used in calibration and all experiments were set to 5.0. Interscan and scan times of 0.1 and 1 seconds, respectively, were used per spectrum (examples of instantaneous spectra are provided in Figure S15). The

collision voltage parameter was increased in 1 V increments per 9 scans, using an AutoHotkey[5] script, to a limit of 50 V. An MCP detector voltage of 2700 V were used for optimal intensity. Signal intensities were normalized with respect to a working total ion count (TIC) and intensities of peaks overlapping with M+1 carbon-13 isotope peaks from adjacent peaks (*e.g. m/z* 76 fragment overlaps with the M+1 peak of the *m/z* 75 fragment) have been corrected by consideration of the number of carbon atoms in those fragments and the isotopic abundance of carbon-13 (1.109 %) (see Sample Calculation). Intensities thus corrected were averaged over 9 scans per collision voltage and corrected for the mass of each phosphinophosphonium studied according to the equation:[6]

$$E_0 = \frac{E_{lab} \cdot m_A}{m_A + m_l}$$

E_0 is the mass normalized collision energy, E_{lab} is the voltage specified for the collision cell, m_A is the mass of the collision gas (argon), and m_l is the mass of the selected phosphinophosphonium.

The reproducibility of ESI-CID-MSMS data was assessed through replicate analysis of $[^t Bu_2PPMe_3][OTf]$ over a period of one week, using standard experimental parameters or variations thereof (see Table S1 for parameters used), and averaging of the data obtained (Figure S16). The data collected from analysis of $[Me_2PPMe_3]^+$ as a chloride salt as well as a triflate salt likewise demonstrate low a standard deviation (Figure S17).

Fragmentation plots showing all fragments detected for each substitution of phosphinophosphonium, selected fragments corresponding to key processes (*i.e.* P–P fission, P–C fission, and β-hydride elimination), and fragments representing P–P fission processes are given in Figures S19, S20, and S21, respectively.

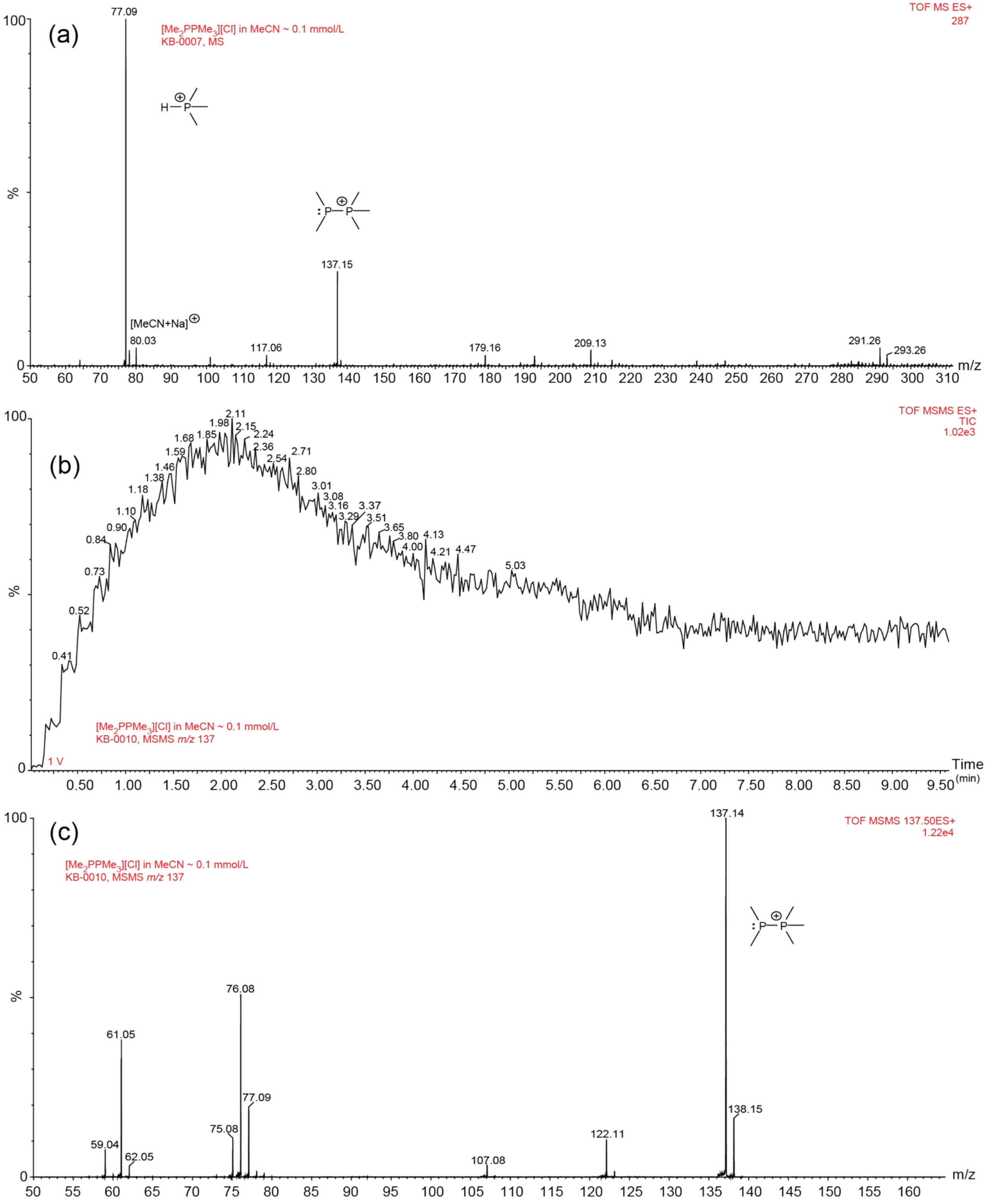

Figure S6. (a) Raw mass spectrum of $[Me_2PPMe_3][Cl]$ in ESI(+)-MS mode, (b) CID chromatogram, and (c) CID mass spectrum of *m/z* 137 molecular ion.

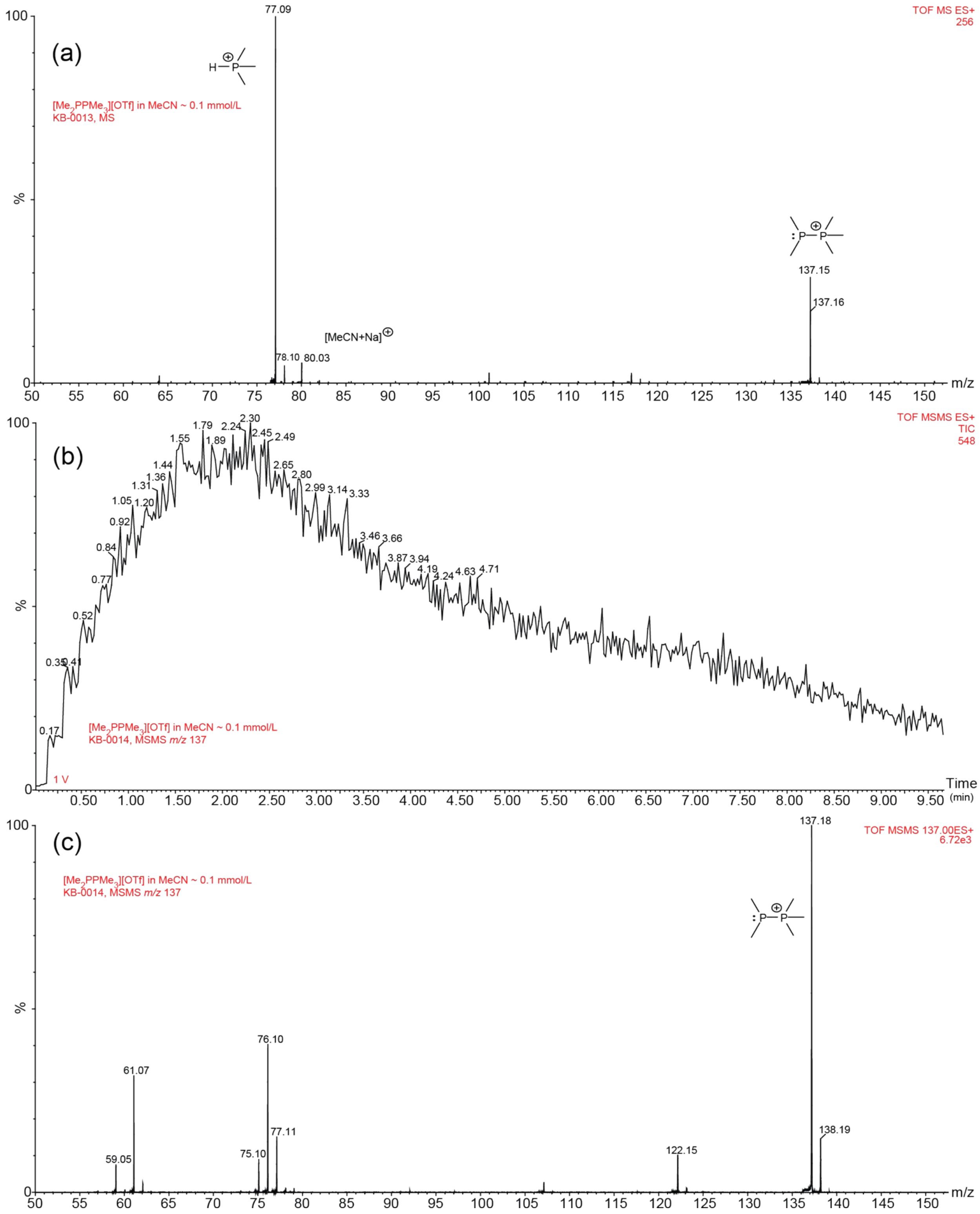

Figure S7. (a) Raw mass spectrum of [Me_2PPMe_3][OTf] in ESI(+)-MS mode, (b) CID chromatogram, and (c) CID mass spectrum of *m/z* 137 molecular ion.

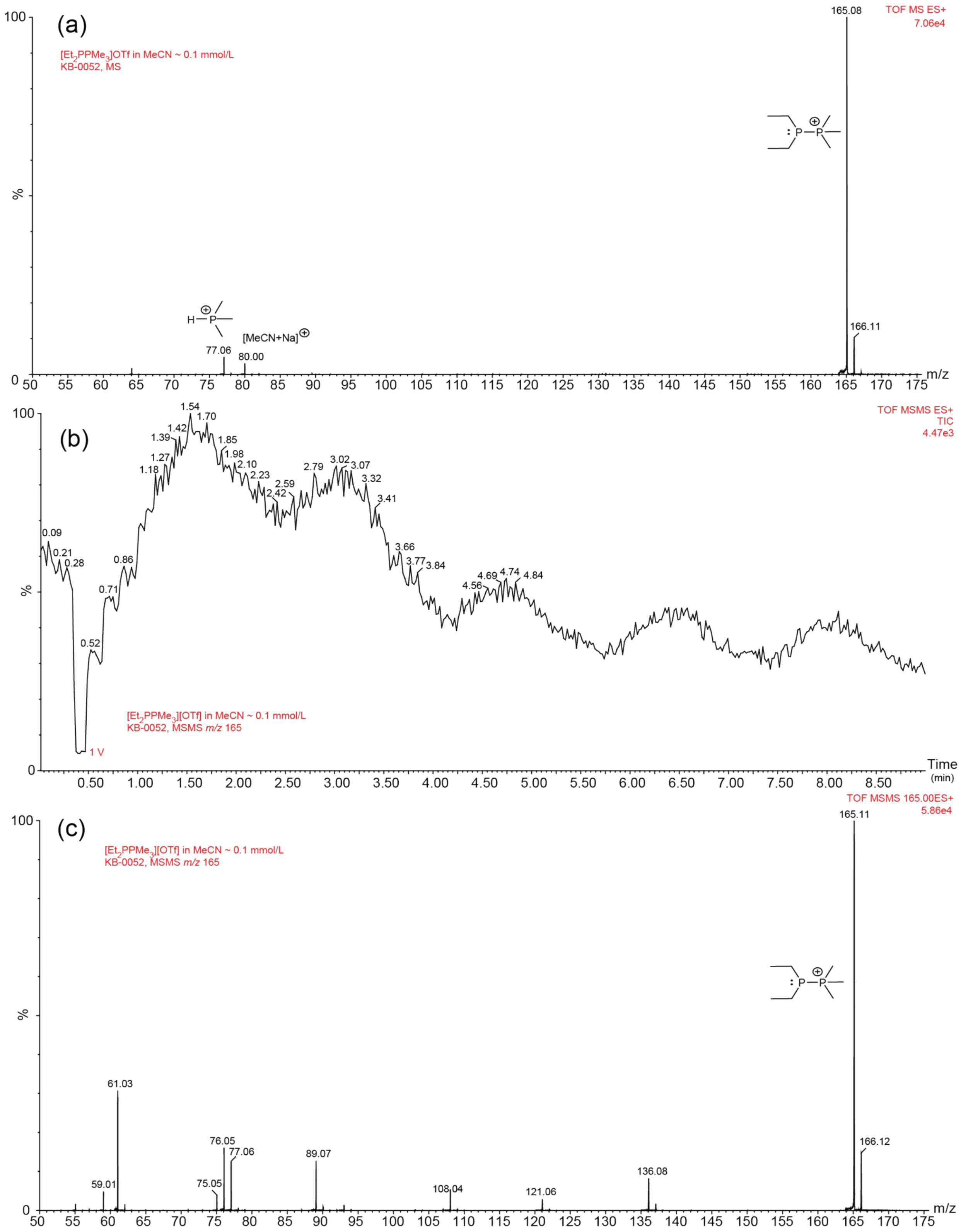

Figure S8. (a) Raw mass spectrum of $[Et_2PPMe_3][OTf]$ in ESI(+)-MS mode, (b) CID chromatogram (periodicity due to malfunctioning regulator), and (c) CID mass spectrum of *m/z* 165 molecular ion.

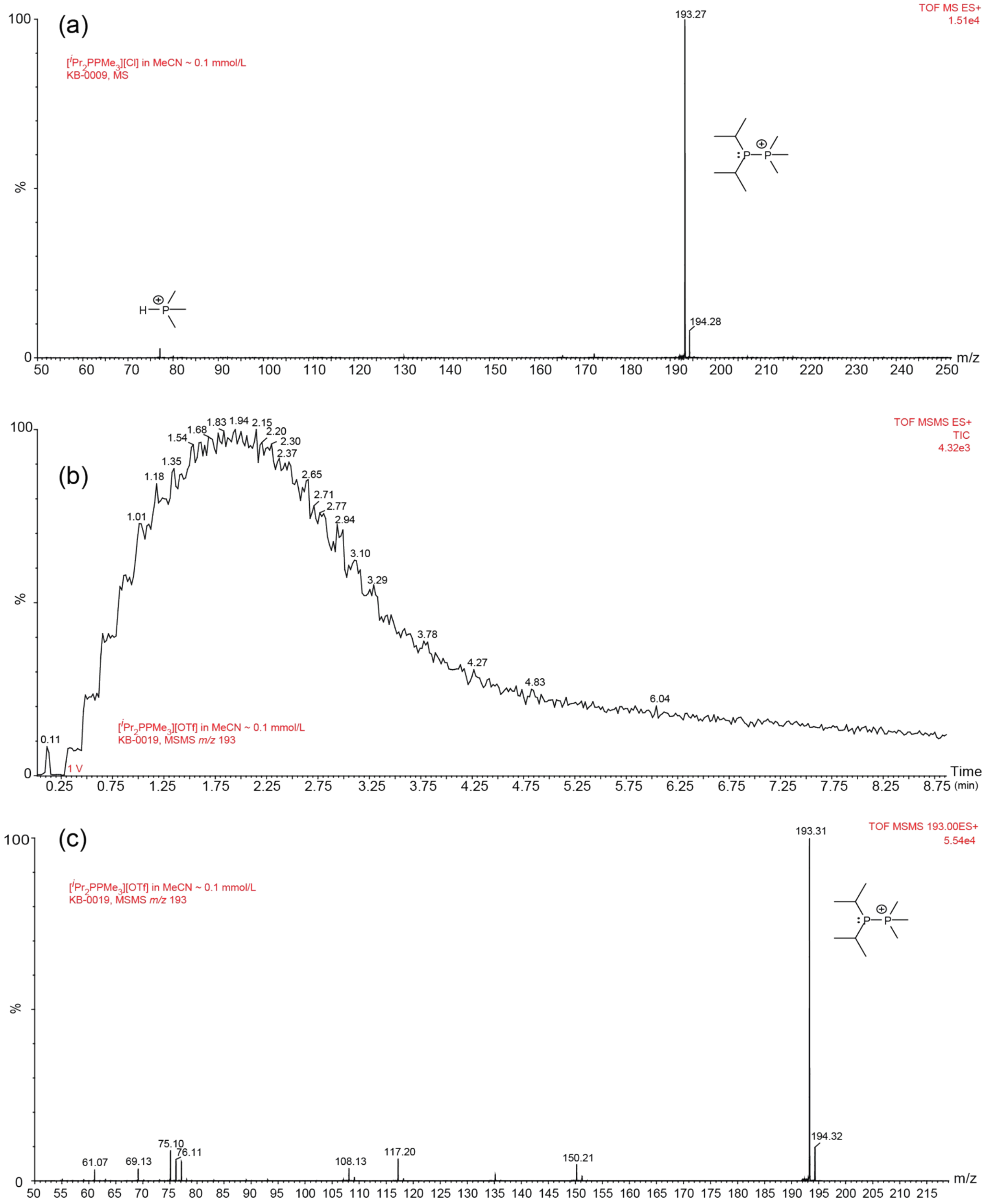

Figure S9. (a) Raw mass spectrum of [^{i}Pr$_2$PPMe$_3$][OTf] in ESI(+)-MS mode, (b) CID chromatogram, and (c) CID mass spectrum of *m/z* 193 molecular ion.

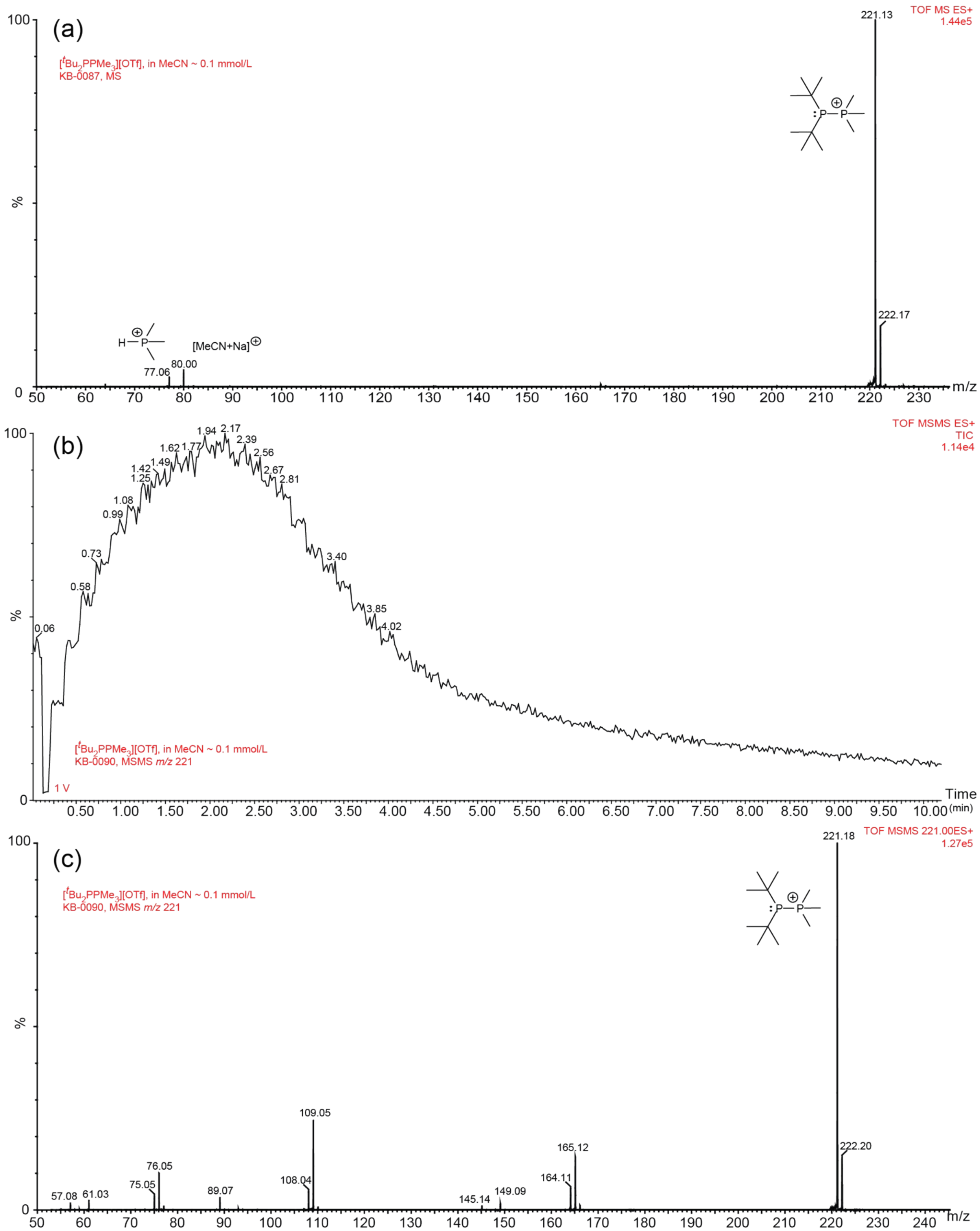

Figure S10. (a) Raw mass spectrum of [^{t}Bu$_2$PPMe$_3$][OTf] in ESI(+)-MS mode, (b) CID chromatogram, and (c) CID mass spectrum of *m/z* 221 molecular ion.

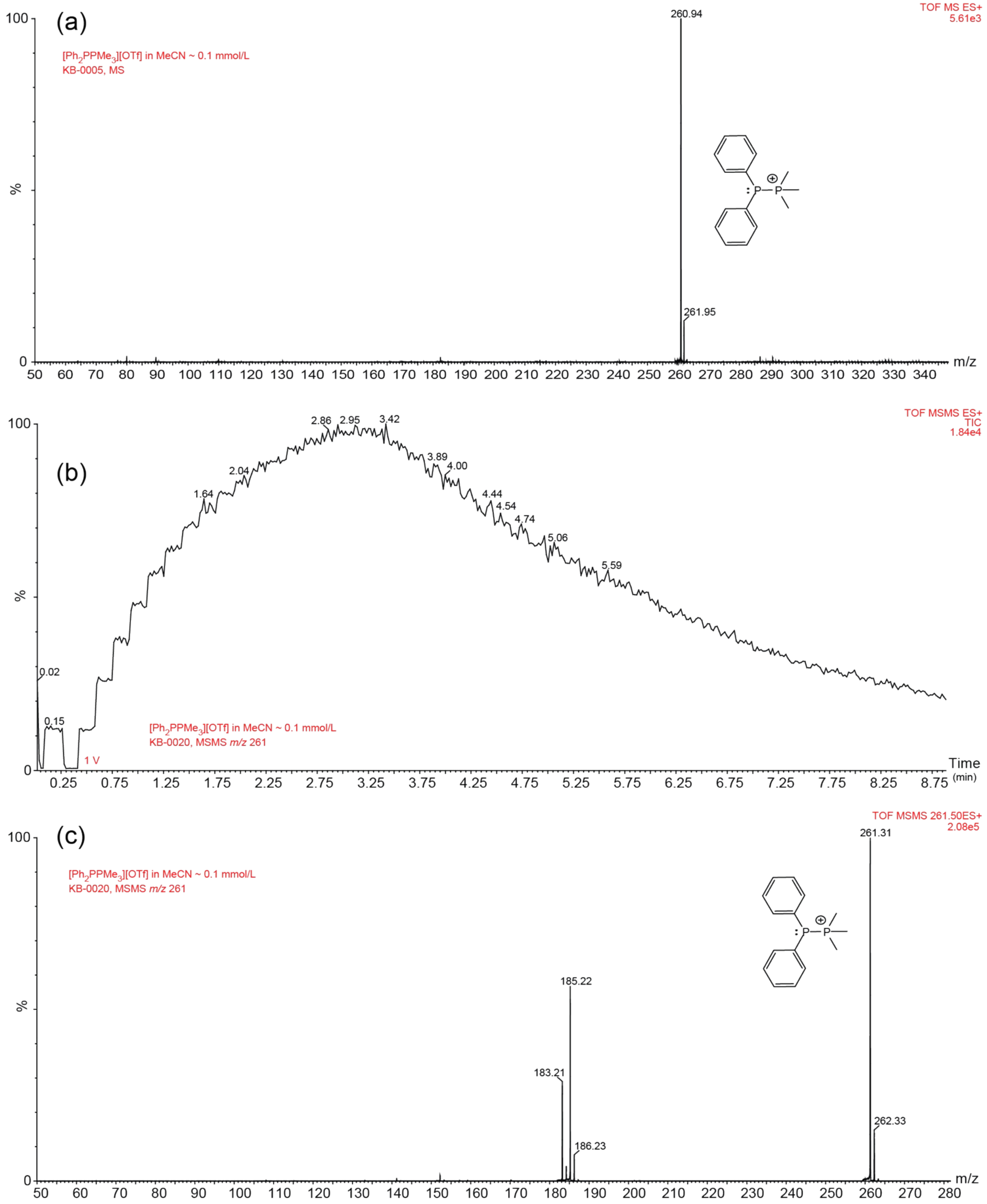

Figure S11. (a) Raw mass spectrum of $[Ph_2PPMe_3][OTf]$ in ESI(+)-MS mode, (b) CID chromatogram and (c) CID mass spectrum of *m/z* 261 molecular ion.

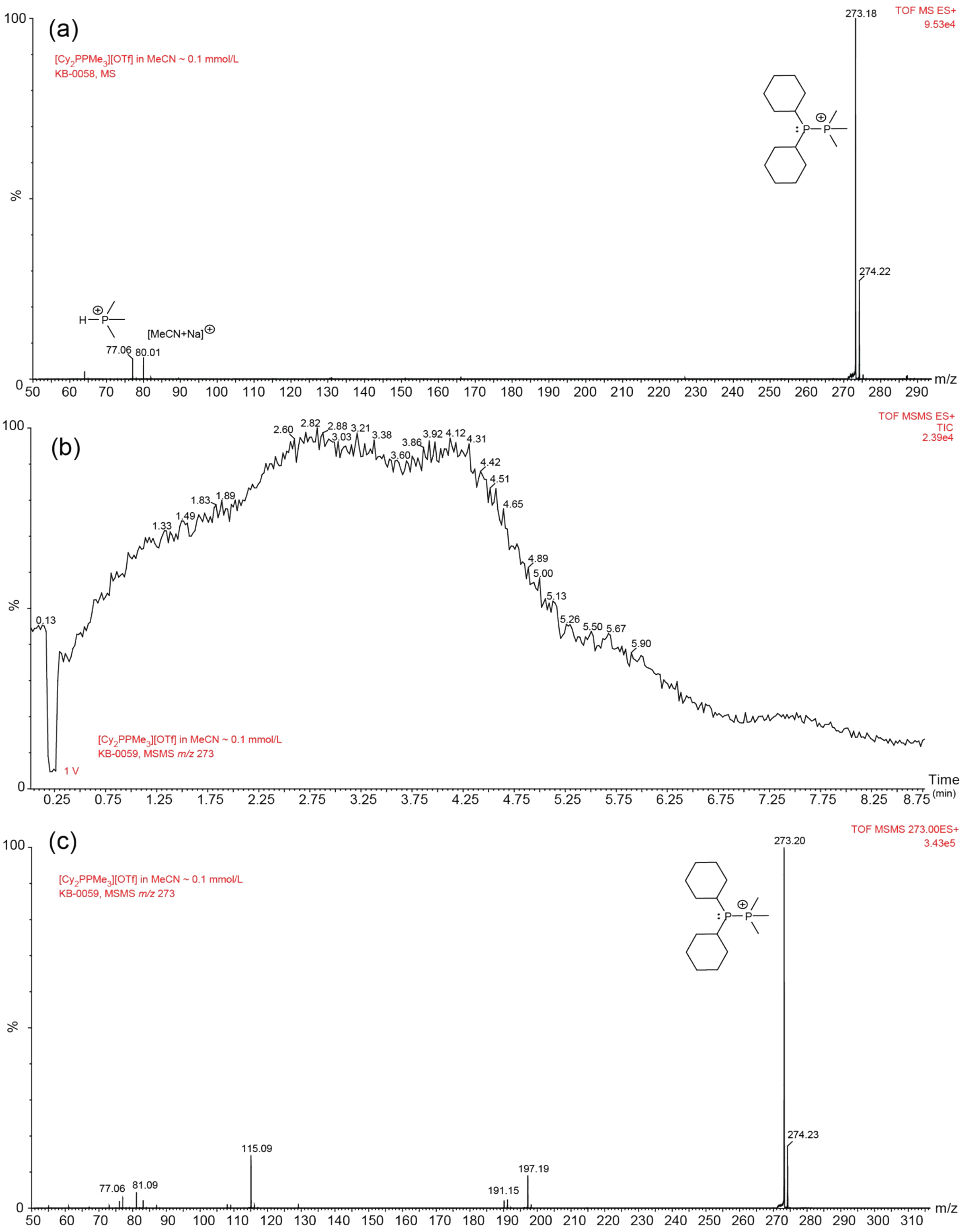

Figure S12. (a) Raw mass spectrum of $[Cy_2PPMe_3][OTf]$ in ESI(+)-MS mode, (b) CID chromatogram, and (c) CID mass spectrum of *m/z* 273 molecular ion.

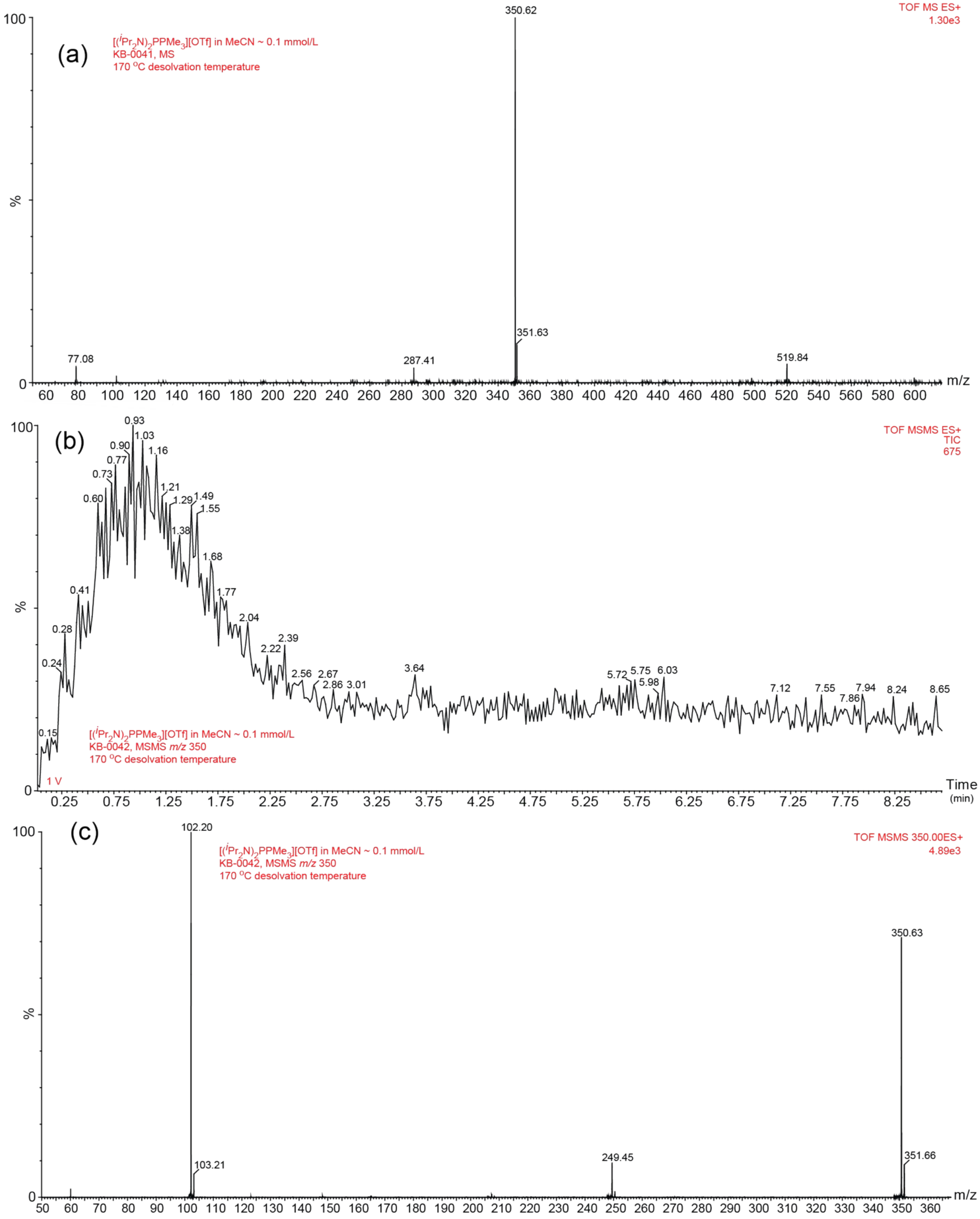

Figure S13. (a) Raw mass spectrum of [(^{i}Pr$_2$N)$_2$PPMe$_3$][OTf] (MeCN solution) in ESI(+)-MS mode, (b) CID chromatogram and (c) CID mass spectrum of *m/z* 350 unidentified molecular ion.

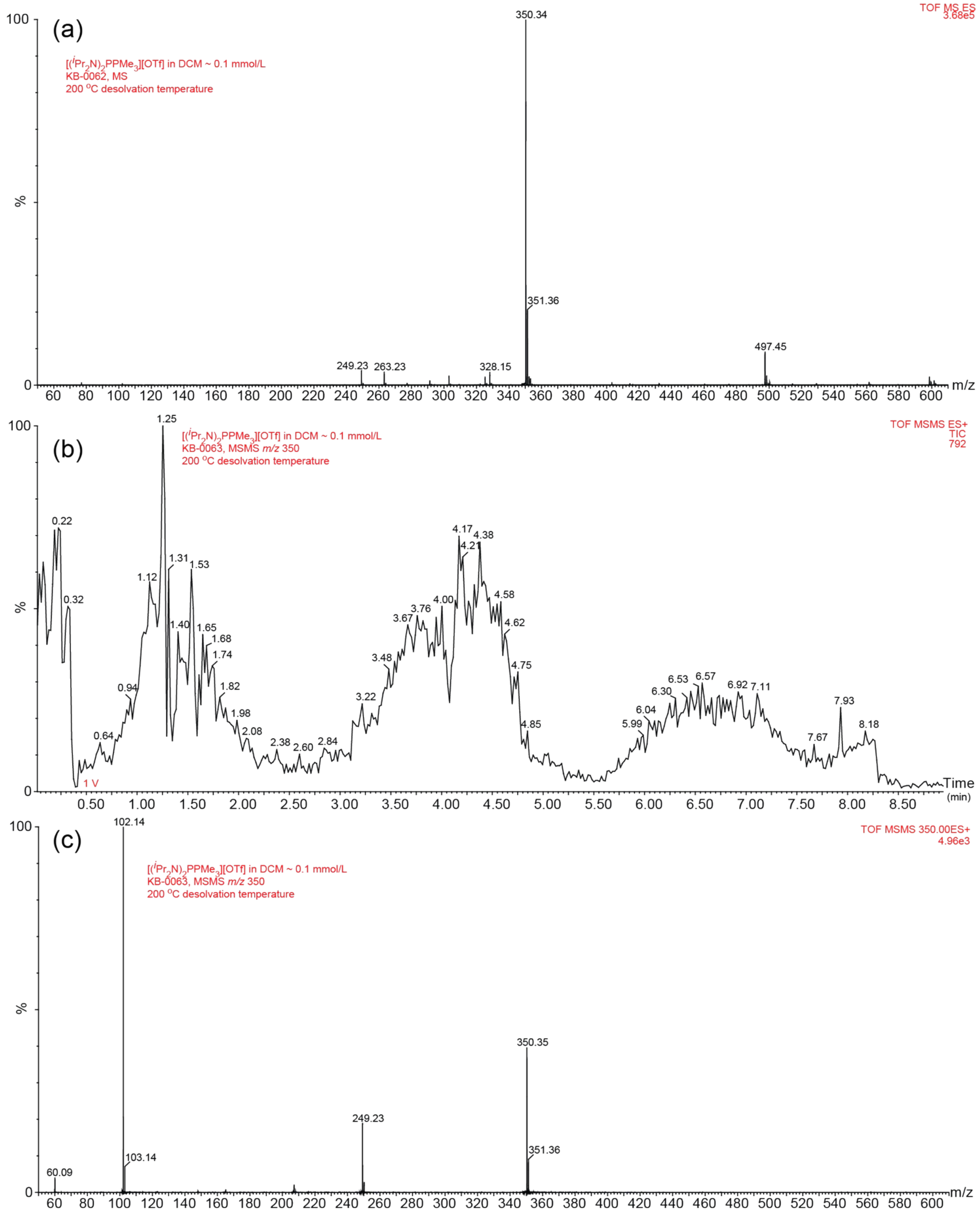

Figure S14. (a) Raw mass spectrum of $[(^i\mathrm{Pr_2N})_2\mathrm{PPMe_3}][\mathrm{OTf}]$ (DCM solution) in ESI(+)-MS mode, (b) CID chromatogram (periodicity due to malfunctioning regulator) and (c) CID mass spectrum of *m/z* 350 unidentified molecular ion.

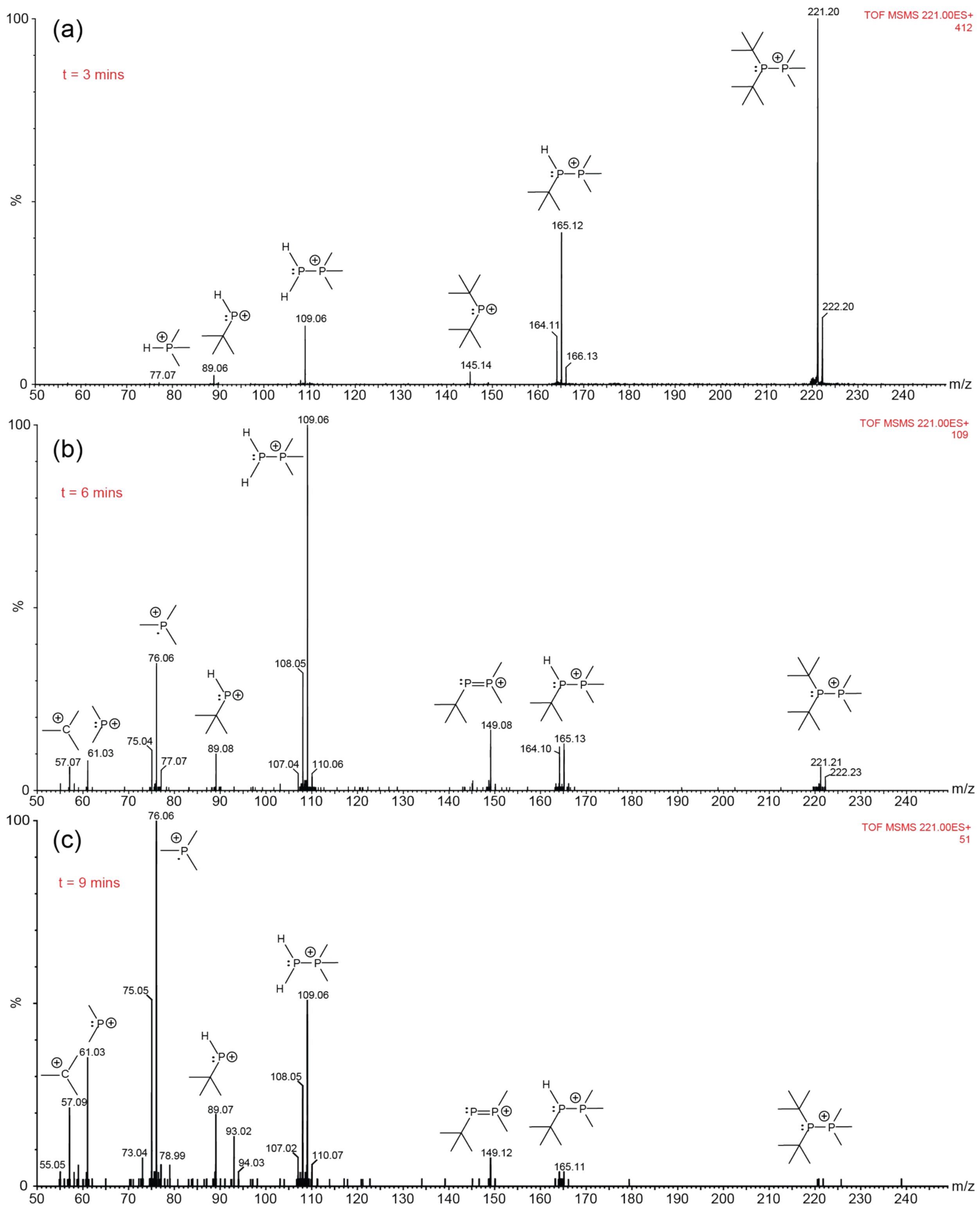

Figure S15. Evolution of instantaneous mass spectra in CID of [^{t}Bu$_2$PPMe$_3$][OTf] over time.

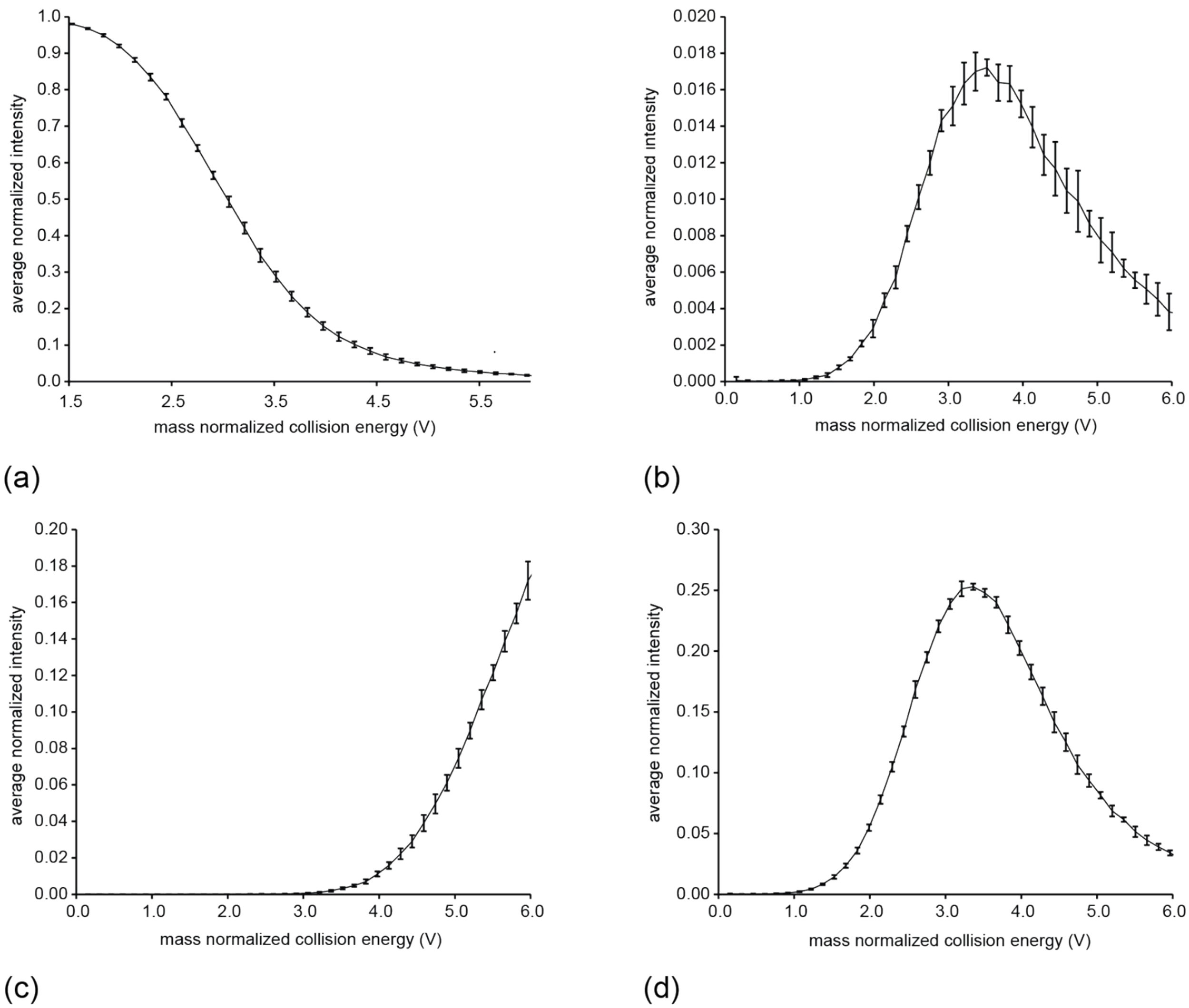

Figure S16. Averaged ESI-CID-MSMS data (n = 9 replicates, see Table S1) for $[^t\text{Bu}_2\text{PPMe}_3][\text{OTf}]$ showing (a) the disappearance of $[^t\text{Bu}_2\text{PPMe}_3]^+$, (b) the appearance of $[^t\text{Bu}_2\text{P}]^+$, (c) the appearance of $[\text{PMe}_3]^{+\bullet}$, and (d) the appearance of $[^t\text{Bu(H)PPMe}_3]^+$ (calculated standard deviation used for error bars).

Table S1. Conditions Used in Replicate Analysis of $[^t\text{Bu}_2\text{PPMe}_3][\text{OTf}]$ by ESI-CID-MSMS

Trial	Desolvation temperature	High and low mass resolution	Time elapsed since Trial 1 data collection
1	200	5.0	-
2	200	5.0	8 minutes
3	200	5.0	20 minutes
4	200	5.0	30 minutes
5	200	5.0	41 minutes
6	200	5.0	50 minutes
7	200	5.0	144 hours
8	170	5.0	144 hours
9	200	1.0	193 hours

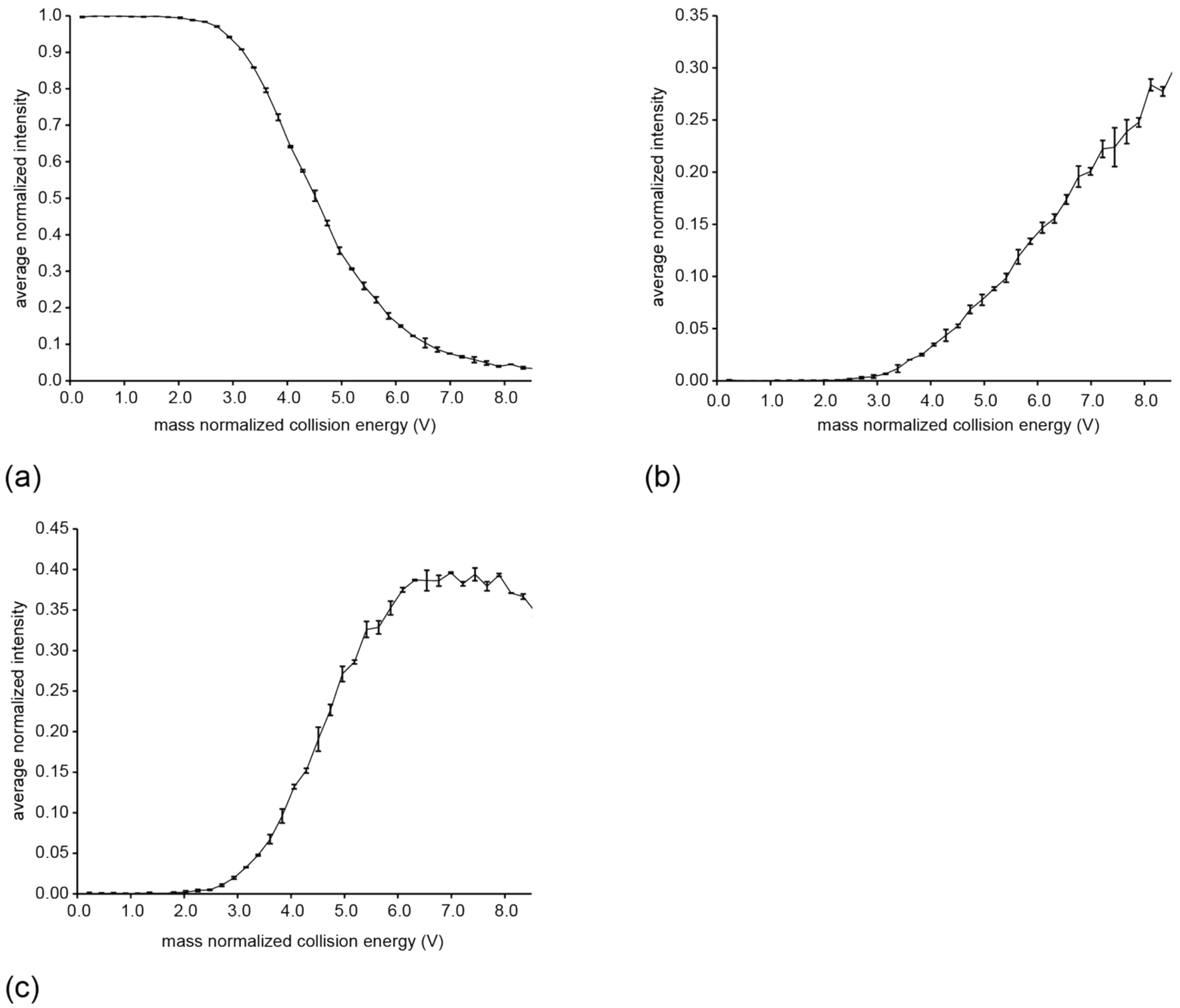

Figure S17. Averaged ESI-CID-MSMS data for $[Me_2PPMe_3][OTf]$ and $[Me_2PPMe_3][Cl]$ showing (a) the disappearance of $[Me_2PPMe_3]^+$, (b) the appearance of $[Me_2P]^+$, and (c) the appearance of $[PMe_3]^{+\bullet}$ (calculated standard deviation used for error bars).

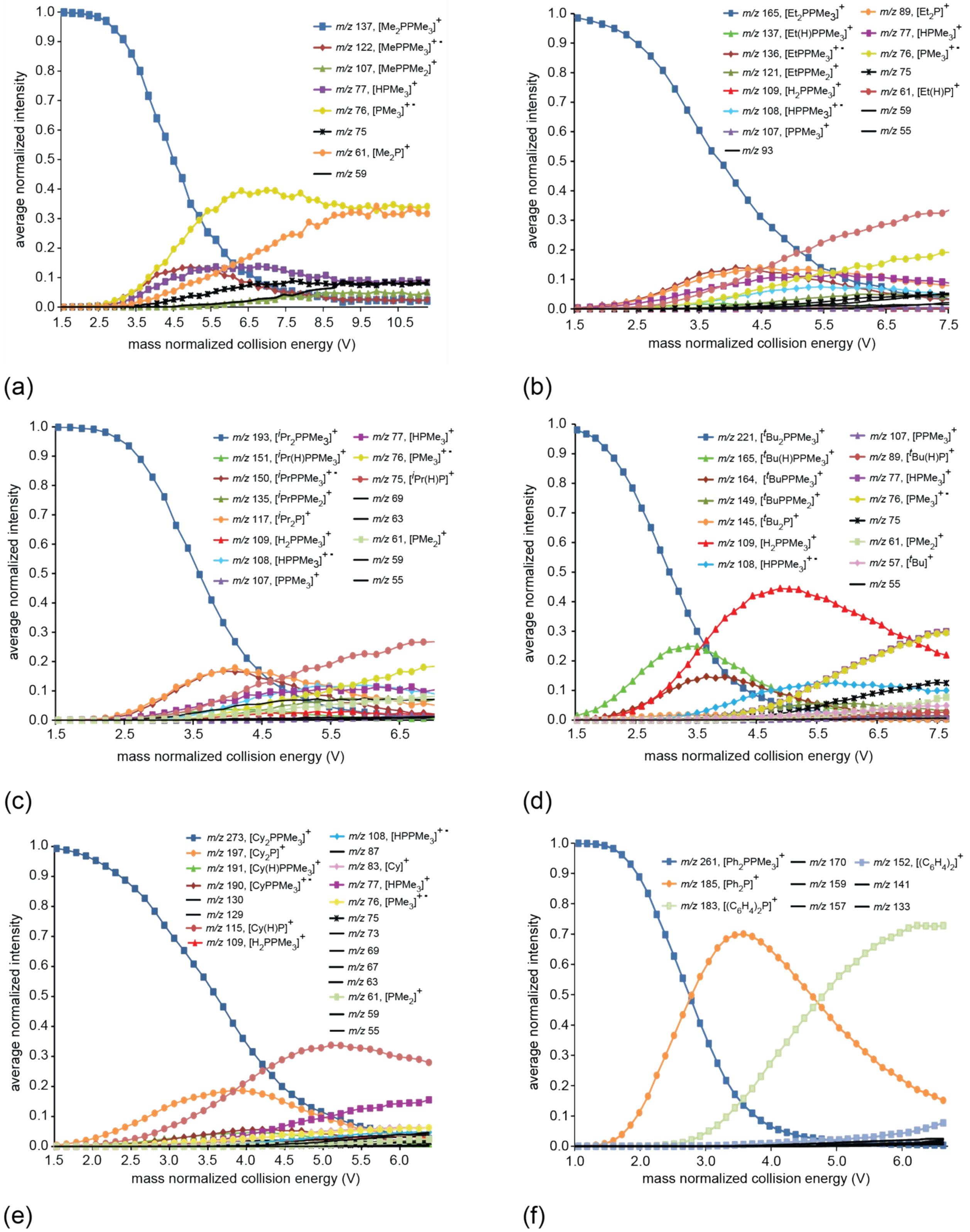

Figure S18. Fragmentation of $[R_2PPMe_3]^+$, where R = Me (a), Et (b), ^iPr (c), ^tBu (d), Cy (e), and Ph (f) (all observed fragments shown).

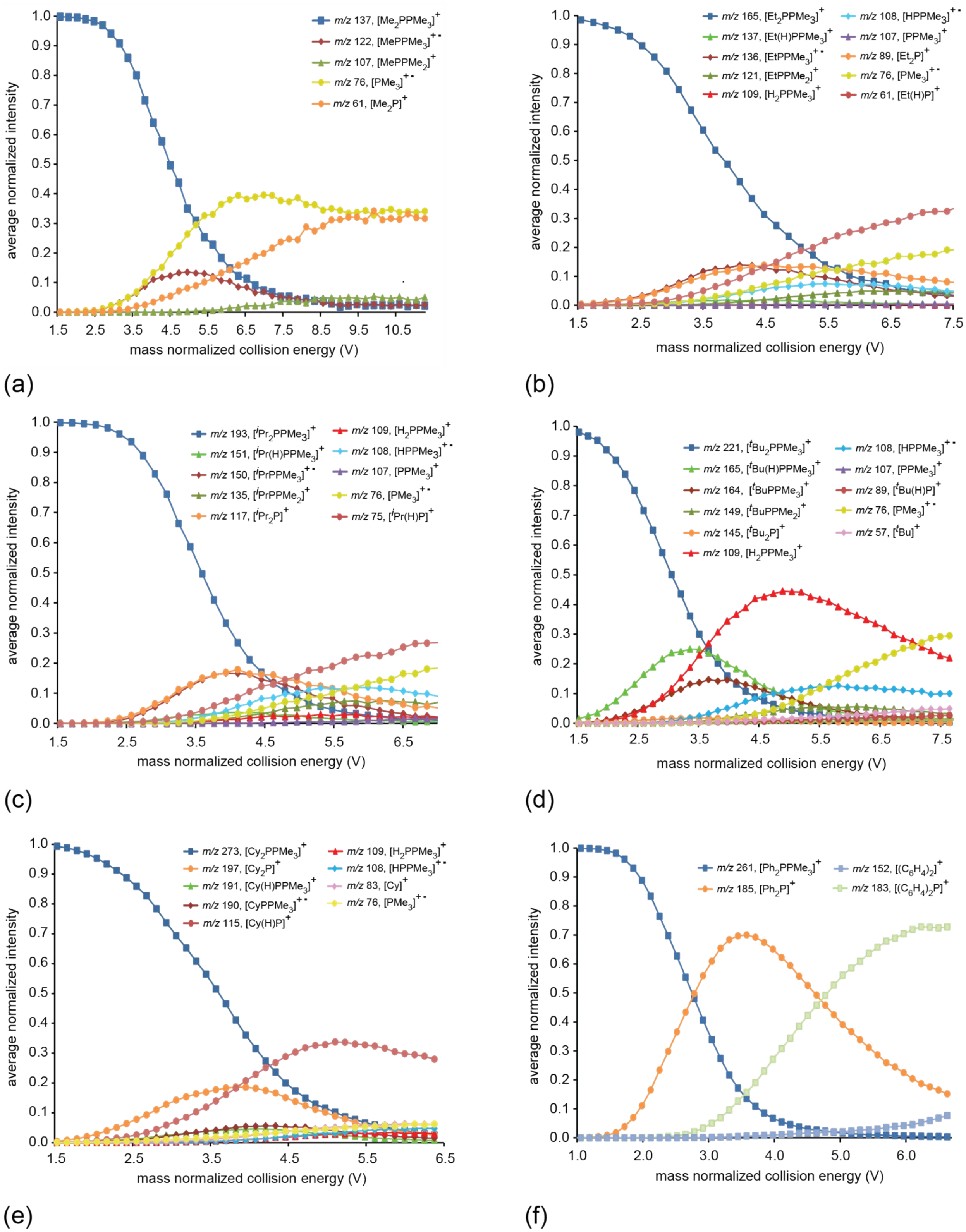

Figure S19. Fragmentation of $[R_2PPMe_3]^+$, where R = Me (a), Et (b), ^{i}Pr (c), ^{t}Bu (d), Cy (e), and Ph (f) (fragments corresponding to discernible processes shown).

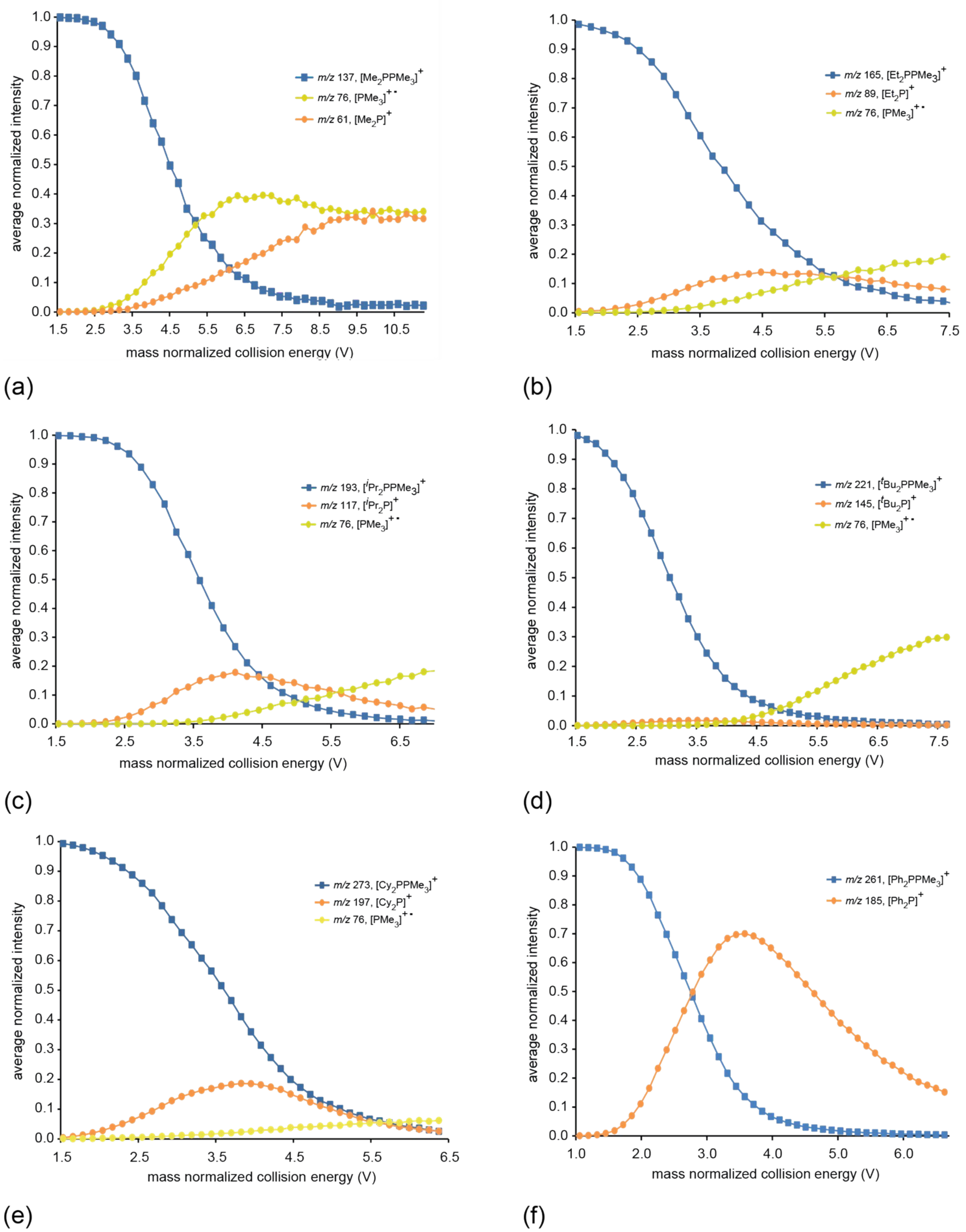

Figure S20. Fragmentation of $[R_2PPMe_3]^+$, where R = Me (a), Et (b), ^{i}Pr (c), ^{t}Bu (d), Cy (e), and Ph (f) (P–P fission products shown).

Sample Calculation for Correction of Intensities Due to M+1 Peaks

(using *m/z* 75 and *m/z* 76 raw intensities from one scan in the analysis of $[Me_2PPMe_3]^+$ as an example)

peak	experiment time	raw intensity	identity	number of carbons
m/z 76	6.038 min	156	$[PMe_3]^{+\bullet}$	3
m/z 75		38	PMe_3 derivative	3

Given that carbon-13 has an isotopic abundance of 1.109 % the percentage of all *m/z* 75 fragments featuring at least one carbon-13 atom is:

M+1 percentage = (100 – 3 · 1.109) % = (100 – 3.327) % = 96.673 %

The relative intensities of the M+ peak (*m/z* 75) and its M+1 peak (*m/z* 76) should therefore theoretically be 3.327:96.673, however a real fragment with mass *m/z* 76 is observed (M*+) and the intensity of this peak is much greater than the theoretical M+1 intensity of the *m/z* 75 M+ peak. The intensity of the *m/z* 76 must therefore be corrected by subtraction, as follows:

Corrected *m/z* 76 M+ intensity $= 156 - 38 \cdot \frac{3.327}{96.673} = 156 - 38 \cdot 0.03441 = 154._{69} = 155$

The correction accounts for small proportion of the observed intensity ($\frac{1}{156}$) but was nonetheless implemented for all instances of M+1/M*+ peak overlap.

Quantum Chemical Calculations

All calculations were performed using Gaussian 09 (see full citation below). Phosphinophosphonium cations $[R_2PPMe_3]^+$ (R = Me, Et, ^{i}Pr, ^{t}Bu, Ph, and N^iPr_2) and fragments corresponding to the variety of processes observed mass spectrometrically ($[R(H)PPMe_3]^+$, $[RPPMe_3]^{+\bullet}$, $[H_2PPMe_3]^+$, $[RPPMe_2]^+$, $[R_2P]^+$, $[R(H)P]^+$, R^+, $R^\bullet$, alkanes R-Me, and olefins) were pre-optimized by the Hartree-Fock (HF) method using the 6-311++G(d,p) basis set in solvent free, gas-phase conditions (298 K) and subsequently modelled at the PBE1PBE/6-311++G(d,p) level of theory. A frequency analysis of the PBE1PBE/6-311++G(d,p) optimized geometries was performed at the same level of theory and as well as single point energy calculations using the MP2/6-311++G(d,p) level of theory. Lastly, the PBE1PBE/6-311++G(d,p) optimized geometries were used as initial guesses in MP2/6-311++G(d,p) optimizations. Optimization criteria typically used for optimizations and frequency calculations were very tight (*i.e.* root-mean-square (RMS) values < $1x10^{-6}$ a.u.) and used in conjunction with an ultra-fine integration grid. Additional keywords (*e.g.* symmetry=none, opt=calcfc) and/or lowered optimization criteria (*e.g.* opt=tight: RMS values < $1x10^{-5}$ a.u.) were necessary for several fragments and are noted below the relevant fragments' coordinates.

Reaction energies have been calculated from enthalpies of formation, Gibbs energies of formation, or single point energies using Hess's Law. Tabulated Gibbs energies of modelled processes are denoted as reaction (ΔG_{rxn}), bond breaking (ΔG_{bb}), and structural relaxation (ΔG_{rel}). The sum of bond breaking and structural relaxation of product fragments is equivalent to the overall reaction Gibbs energy (*i.e.* $\Delta G_{rxn} = \Delta G_{bb} + \Delta G_{rel}$).

Citation for Gaussian 09: Gaussian 09, Revision D.01, M. J. Frisch, G. W. Trucks, H. B. Schlegel, G. E. Scuseria, M. A. Robb, J. R. Cheeseman, G. Scalmani, V. Barone, B. Mennucci, G. A. Petersson, H. Nakatsuji, M. Caricato, X. Li, H. P. Hratchian, A. F. Izmaylov, J. Bloino, G. Zheng, J. L. Sonnenberg, M. Hada, M. Ehara, K. Toyota, R. Fukuda, J. Hasegawa, M. Ishida, T. Nakajima, Y. Honda, O. Kitao, H. Nakai, T. Vreven, J. A. Montgomery, Jr., J. E. Peralta, F. Ogliaro, M. Bearpark, J. J. Heyd, E. Brothers, K. N. Kudin, V. N. Staroverov, T. Keith, R. Kobayashi, J. Normand, K. Raghavachari, A. Rendell, J. C. Burant, S. S. Iyengar, J. Tomasi, M. Cossi, N. Rega, J. M. Millam, M. Klene, J. E. Knox, J. B. Cross, V. Bakken, C. Adamo, J. Jaramillo, R. Gomperts, R. E. Stratmann, O. Yazyev, A. J. Austin, R. Cammi, C. Pomelli, J. W. Ochterski, R. L. Martin, K. Morokuma, V. G. Zakrzewski, G. A. Voth, P. Salvador, J. J. Dannenberg, S. Dapprich, A. D. Daniels, O. Farkas, J. B. Foresman, J. V. Ortiz, J. Cioslowski, and D. J. Fox, Gaussian, Inc., Wallingford CT, 2013.

Table S2. Benchmarking Study for Level of Theory used in Gas Phase (298 K) Modelling of $[Me_2PPMe_3]^+$ with Basis Set 6-311++G(d,p)

Level of theory	CCSD	MP2	B3LYP	B3PW91	PBE1PBE	*experimental*
***d*(PP) /Å**	2.216	2.202	2.238	2.219	2.209	2.1767(6)
**ν(PP) /cm^{-1}**	--	443.4	409.6	422.3	432.8	446

Table S3. Benchmarking Study for Basis Set used in Gas Phase (298 K) Modelling of $[Me_2PPMe_3]^+$ with PBE1PBE Level of Theory

Basis Set	6-311++G(d,p)	6-311+G(d,p)	6-311G(d,p)	6-311++G(d)	*experimental*
***d*(PP)/Å**	2.209	2.209	2.209	2.209	2.1767(6)
**ν(PP) /cm^{-1}**	432.8	432.9	434.4	433.0	446

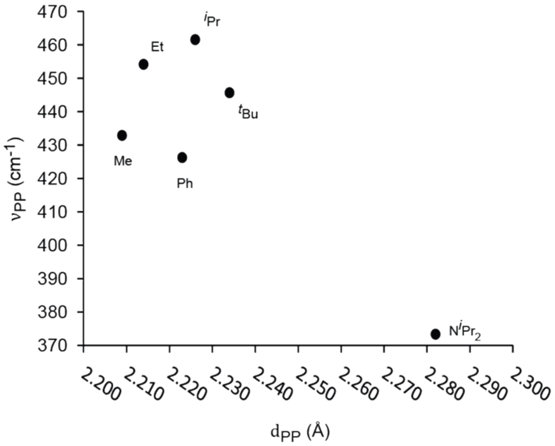

Figure S21. Correlation of calculated P–P stretching frequency and bond length in the series of phosphinophosphonium cations $[R_2PPMe_3]^+$ (R = Me, Et, ^{i}Pr, ^{t}Bu, Ph, N^{i}Pr$_2$) at the PBE1PBE/6-311++G(d,p) level.

Dissociation Trends

The consistency of trends observed for dissociation processes in the phosphinophosphonium $[Et_2PPMe_3]^+$ are established in Figure S22 by comparison of reaction energies (Table S4) obtained through Hess's Law from PBE1PBE/6-311++G(d,p) frequency analysis both with and without dispersion correction, and from MP2/6-311++G(d,p) optimization single point energies. $[Me_2PPMe_3]^+$ was not selected for this analysis because it cannot exhibit β-hydride elimination. Dispersion correction is expected to have a more significant impact towards optimization and frequency analysis in more sterically conjected systems. Comparison of Gibbs energies for the phosphinophosphonium $[^tBu_2PPMe_3]^+$ calculated at PBE1PBE/6-311++G(d,p) with and without the keyword GD3 for Grimme's D3 dispersion correction confirms that the relative energies between the studied dissociation processes are preserved irrespective of dispersion treatment (see Figure S23 and Table S5). Note that P–C cleavage, unless otherwise stated, refers to homolytic or heterolytic processes from the *trivalent* phosphorus (*i.e.* loss of R^+ or R·).

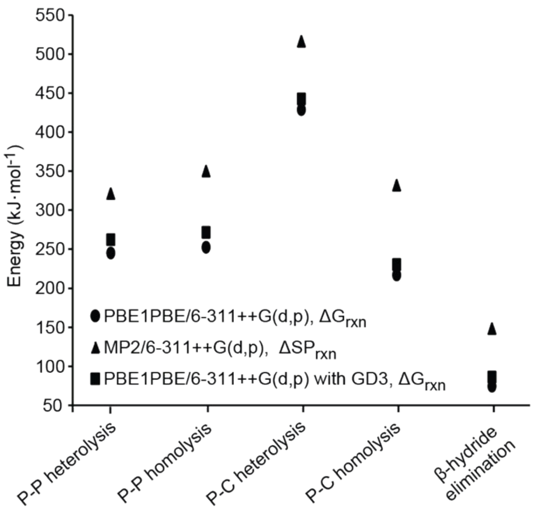

Figure S22. Comparison of calculated PBE1PBE/6-311++G(d,p) Gibbs energies of reaction, dispersion corrected (GD3) PBE1PBE/6-311++G(d,p) Gibbs energies of reaction and MP2/6-311++G(d,p) single point (SP) energies of reaction for dissociation processes in $[Et_2PPMe_3]^+$.

Table S4. Calculated Energies of Reaction for Dissociation Processes in $[Et_2PPMe_3]^+$

Process	PBE1PBE/6-311++G(d,p) ΔG_{rxn} /kJ·mol^{-1}	Dispersion Corrected* PBE1PBE/6-311++G(d,p) ΔG_{rxn} /kJ·mol^{-1}	MP2/6-311++G(d,p) ΔSP_{rxn} /kJ·mol^{-1}
P–P heterolysis	245	262	321
P–P homolysis	252	271	350
P–C heterolysis	429	442	516
P–C homolysis	217	230	332
β-hydride elimination	74	86	148

* Grimme's D3 dispersion correction applied (*i.e.* the keyword Empirical Dispersion=GD3 was used)

Note: SP stands for single point

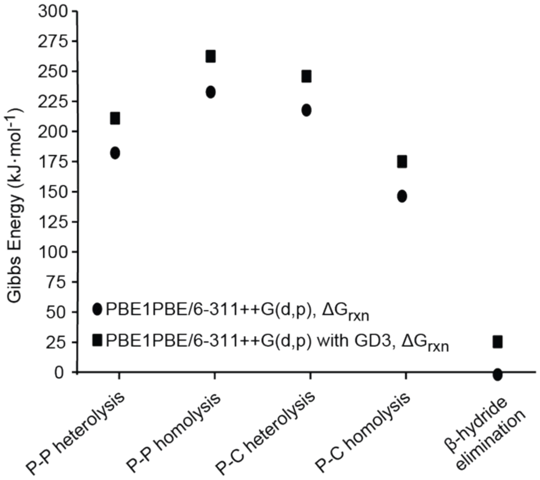

Figure S23. Comparison of calculated PBE1PBE/6-311++G(d,p) Gibbs energies of reaction and dispersion corrected (GD3) PBE1PBE/6-311++G(d,p) Gibbs energies of reaction for dissociation processes in $[^tBu_2PPMe_3]^+$.

Table S5. Calculated Gibbs Energies of Reaction for Dissociation Processes in $[^tBu_2PPMe_3]^+$

Process	PBE1PBE/6-311++G(d,p) ΔG_{rxn} /kJ·mol^{-1}	Dispersion Corrected* PBE1PBE/6-311++G(d,p) ΔG_{rxn} /kJ·mol^{-1}
P–P heterolysis	182	211
P–P homolysis	233	262
P–C heterolysis	218	246
P–C homolysis	146	175
β-hydride elimination	-2	25

Reaction Enthalpies and Gibbs Energies$^{\alpha}$

$^{\alpha}$ calculated from enthalpies and Gibbs energies of formation; species were modelled in the gas phase (298 K) at the PBE1PBE/6-311++G(d,p) level

Table S6. Selected Calculated Bond Lengths (Å) and Stretching Frequencies (cm^{-1}) in $[R_2PPMe_3]^+$, Enthalpies and Gibbs Energies (kJ·mol^{-1}) for Heterolytic P–P Bond Cleavage

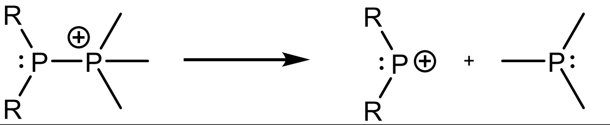

R	*d*(PP) /Å	ν(PP) /cm^{-1}	*d*(PC)$_{avg}$* /Å	ΔH_{rxn} /kJ·mol^{-1}	ΔG_{rxn} /kJ·mol^{-1}	structural relaxation ΔG_{rel} /kJ·mol^{-1}	bond break ΔG_{bb} /kJ·mol^{-1}
Me	2.209	433	1.807	346	294	-50	345
Et	2.214	454	1.810	294	245	-81	325
^{i}Pr	2.226	462	1.814	256	199	-92	291
^{t}Bu	2.234	446	1.816	242	182	-89	271
Ph	2.223	426	1.809	232	182	-64	247

N(^{i}Pr)$_2$	2.282	373	1.819	60	6	-127	133

*P-C bonds on the tetravalent phosphorus (*i.e.* the PMe_3 moiety)

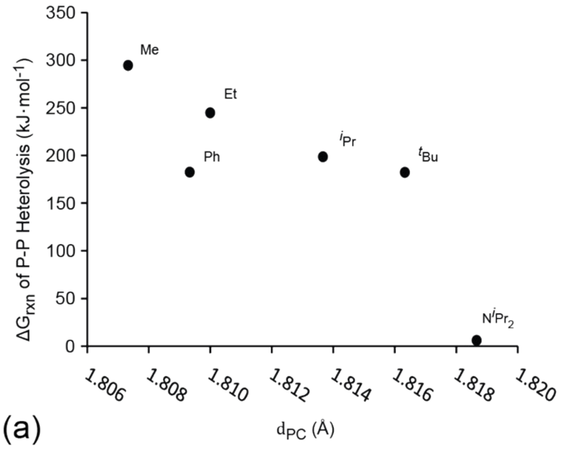

Figure S24. The relation of calculated Gibbs energies of reaction for P–P heterolysis in $[R_2PPMe_3]^+$ and calculated d(PC) in PMe_3 moieties (R = Me, Et, ^{i}Pr, ^{t}Bu, Ph, N^{i}Pr$_2$).

Table S7. Calculated Enthalpies and Gibbs Energies of Reaction (kJ·mol^{-1}) for $[R_2P]^•$ Ionization

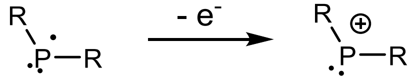

R	ΔH_{rxn} /kJ·mol^{-1}	ΔG_{rxn} /kJ·mol^{-1}
Me	778	779
Et	720	729
^{i}Pr	700	703
^{t}Bu	683	685
Ph	668	673
N(^{i}Pr)$_2$	520	525

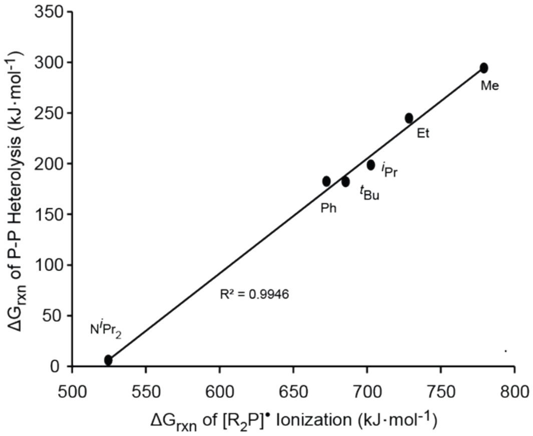

Figure S25. The relation of calculated Gibbs energies reaction for P–P heterolysis in $[R_2PPMe_3]^+$ and $[R_2P]^•$ ionization (R = Me, Et, ^{i}Pr, ^{t}Bu, Ph, N^{i}Pr$_2$).

Table S8. Calculated P–P Bond Length (Å) and Stretching Frequencies (cm^{-1}), Enthalpies and Gibbs Energies (kJ·mol^{-1}) for Homolytic P–P Bond Cleavage

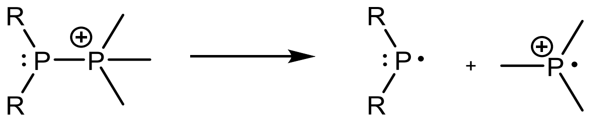

R	*d*(PP) /Å	ν(PP) /cm^{-1}	ΔH$_{rxn}$ /kJ·mol^{-1}	ΔG$_{rxn}$ /kJ·mol^{-1}	**structural relaxation** ΔG$_{rel}$ /kJ·mol^{-1}	**bond break** ΔG$_{bb}$ /kJ·mol^{-1}
Me	2.209	433	309	251	-9	260
Et	2.214	454	315	252	-21	273
^{i}Pr	2.226	462	297	232	-32	264
^{t}Bu	2.234	446	299	233	-34	267
Ph	2.223	426	305	246	-17	263
N(^{i}Pr)$_2$	2.282	373	281	217	-74	292

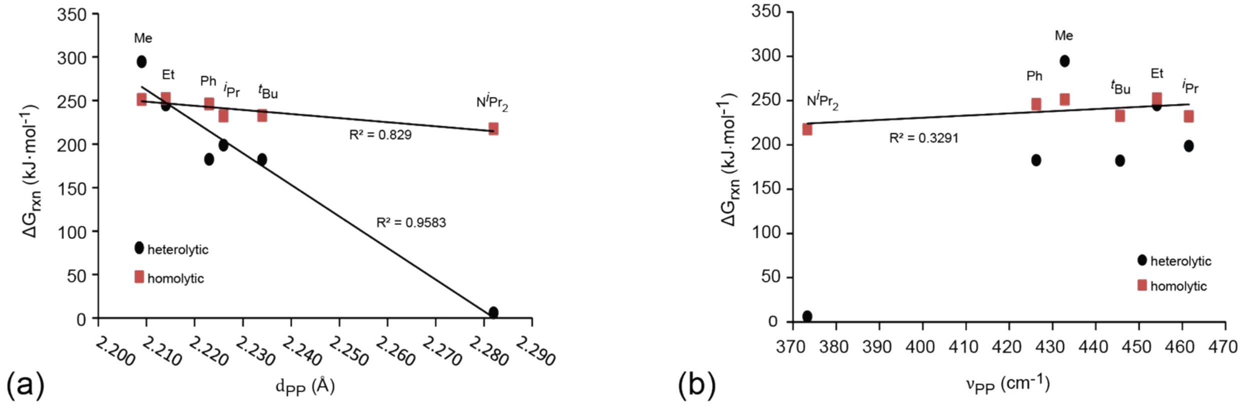

Figure S26. Gibbs energies of reaction (kJ·mol^{-1}) for heterolysis and homolysis with respect to (a) (d$_{PP}$) and (b) (ν$_{PP}$).

Table S9. Enthalpies and Gibbs Energies (kJ·mol-1) for Heterolytic P–C and P–N Bond Cleavage (P-R Cleavage)

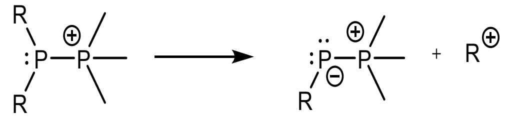

			structural relaxation	bond break
**R**	**ΔH$_{rxn}$ /kJ·mol^{-1}**	**ΔG$_{rxn}$ /kJ·mol^{-1}**	**ΔG$_{rel}$ /kJ·mol^{-1}**	**ΔG$_{bb}$ /kJ·mol^{-1}**
Me	670	620	-158	778
Et	482	429	-195	623
^{i}Pr	370	310	-205	516
^{t}Bu	286	218	-234	452
Ph	542	487	-161	647
N(^{i}Pr)$_2$	453	392	-143	535

Table S10. Enthalpies and Gibbs Energies (kJ·mol-1) for Heterolytic P–C (P-Me Cleavage)

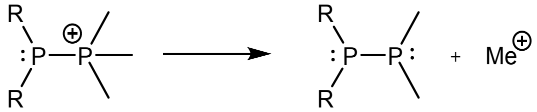

			structural relaxation	bond break
**R**	**ΔH$_{rxn}$ /kJ·mol^{-1}**	**ΔG$_{rxn}$ /kJ·mol^{-1}**	**ΔG$_{rel}$ /kJ·mol^{-1}**	**ΔG$_{bb}$ /kJ·mol^{-1}**
Me	608	561	-163	724
Et	613	562	-162	724
^{i}Pr	612	561	-166	727
^{t}Bu	615	564	-175	740
Ph	617	569	-177	746
N(^{i}Pr)$_2$	617	566	-223	789

Table S11. Enthalpies and Gibbs Energies (kJ·mol-1) for Homolytic P–C and P–N Bond Cleavage (P-R Cleavage)

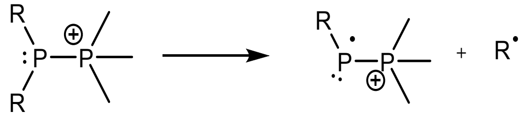

			structural relaxation	bond break
**R**	**ΔH$_{rxn}$ /kJ·mol^{-1}**	**ΔG$_{rxn}$ /kJ·mol^{-1}**	**ΔG$_{rel}$ /kJ·mol^{-1}**	**ΔG$_{bb}$ /kJ·mol^{-1}**
Me	300	247	-40	287
Et	282	217	-49	265
^{i}Pr	247	179	-50	229
^{t}Bu	219	146	-61	207
Ph	332	274	-23	298
N(^{i}Pr)$_2$	227	161	-25	186

Table S12. Enthalpies and Gibbs Energies (kJ·mol-1) for Homolytic P–C Cleavage (P-Me Cleavage)

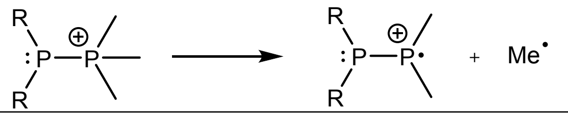

R	ΔH_{rxn} /kJ·mol^{-1}	ΔG_{rxn} /kJ·mol^{-1}	structural relaxation ΔG_{rel} /kJ·mol^{-1}	bond break ΔG_{bb} /kJ·mol^{-1}
Me	334	282	-78	360
Et	331	277	-72	349
^{i}Pr	316	261	-75	335
^{t}Bu	327	268	-63	330
Ph	323	269	-85	354
N(^{i}Pr)$_2$	246	188	-138	326

Table S13. Reaction Enthalpies, Reaction Gibbs Energies, and Activation Energies (kJ·mol^{-1}) for β-Hydride Elimination

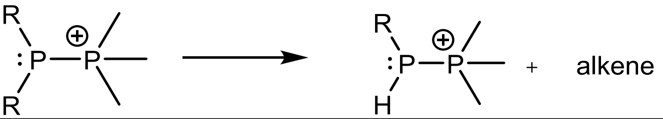

R	R'	alkene or imine	ΔH_{rxn} /kJ·mol^{-1}	ΔG_{rxn} /kJ·mol^{-1}	E_a /kJ·mol^{-1}
Et,	Et	ethene	125	74	229
^{i}Pr	^{i}Pr	propene	87	31	164
^{t}Bu	^{t}Bu	isobutylene	60	-2	187
N(^{i}Pr)$_2$	N(^{i}Pr)$_2$	2-isopropyliminopropane	49	-21	-
Et,	H	ethene	128	83	-
^{i}Pr	H	propene	117	65	-
^{t}Bu	H	isobutylene	100	47	-
N(^{i}Pr)$_2$	H	2-isopropyliminopropane	79	20	-

Benchmarking

The geometry of $[Me_2PPMe_3]^+$ was optimized in the gas phase (298 K) with various basis set/functional combinations, see PBE1PBE Optimization section for data at the chosen PBE1PBE/6-311++G(d,p) level. The starting geometry used in all benchmarking optimizations was that previously obtained at the HF/6-311++G(d,p) level. Values of d(PP) show no basis set dependence (Figure S21). Despite the basis set 6-311G(d,p) yielding the most accurate results with respect to both d(PP) and ν(PP), the basis set 6-311++G(d,p) was selected for this computational study in order to enable future continuation to anionic species, where basis sets with diffuse functions are essential.[7]

Level of Theory

MP2/6-311++G(d,p)

P 0.841293 0.000000 0.013736
P -1.233000 0.000000 -0.724496
C 1.668412 1.461397 -0.651247
C 1.668417 -1.461384 -0.651269

C	-1.874878	1.423436	0.262279
C	-1.874881	-1.423435	0.262281
H	1.228828	2.370560	-0.233980
H	1.562922	1.478046	-1.738980
H	2.730419	1.430283	-0.392105
H	1.228835	-2.370556	-0.234019
H	2.730423	-1.430273	-0.392125
H	1.562929	-1.478016	-1.739003
C	1.044400	-0.000013	1.811847
H	0.584337	0.891335	2.244830
H	2.111941	-0.000011	2.052203
H	0.584343	-0.891371	2.244817
H	-1.440795	2.363694	-0.086950
H	-1.727574	1.319964	1.340437
H	-2.950724	1.479360	0.069719
H	-1.440794	-2.363693	-0.086942
H	-2.950726	-1.479361	0.069715
H	-1.727583	-1.319957	1.340439

(Hartree/Particle)
HF(MP2)=-879.4386487
MP2=-880.453661
Zero-point correction= 0.193337
Thermal correction to Energy= 0.205595
Thermal correction to Enthalpy= 0.206539
Thermal correction to Gibbs Free Energy= 0.155739
Sum of electronic and zero-point Energies= -880.260323
Sum of electronic and thermal Energies= -880.248066
Sum of electronic and thermal Enthalpies= -880.247122
Sum of electronic and thermal Free Energies= -880.297922

B3LYP/6-311++G(d,p)

P	0.862930	0.000000	0.014731
P	-1.256199	0.000000	-0.706336
C	1.682980	1.473040	-0.672769
C	1.682980	-1.473040	-0.672769
C	-1.920046	1.440138	0.266407
C	-1.920046	-1.440138	0.266407
H	1.251202	2.380380	-0.247453
H	1.557434	1.491751	-1.756702
H	2.748508	1.443898	-0.434832
H	1.251202	-2.380380	-0.247453
H	2.748508	-1.443898	-0.434832
H	1.557434	-1.491751	-1.756702
C	1.110640	0.000000	1.822556
H	0.659032	0.889399	2.264364
H	2.180985	0.000000	2.042447
H	0.659032	-0.889399	2.264364
H	-1.485188	2.375516	-0.089664
H	-1.790017	1.353092	1.346511
H	-2.991469	1.485477	0.054092
H	-1.485188	-2.375516	-0.089664
H	-2.991469	-1.485477	0.054092
H	-1.790017	-1.353092	1.346511

(Hartree/Particle)
HF=-882.1907878
Zero-point correction= 0.190531
Thermal correction to Energy= 0.202945
Thermal correction to Enthalpy= 0.203890
Thermal correction to Gibbs Free Energy= 0.153085
Sum of electronic and zero-point Energies= -882.000257
Sum of electronic and thermal Energies= -881.987842
Sum of electronic and thermal Enthalpies= -881.986898
Sum of electronic and thermal Free Energies= -882.037703

B3PW91/6-311++G(d,p)

P	0.856471	0.000000	0.016083
P	-1.243931	0.000000	-0.700780
C	1.668989	1.467161	-0.671310
C	1.668989	-1.467161	-0.671310
C	-1.906293	1.434744	0.263423
C	-1.906293	-1.434744	0.263423
H	1.239932	2.374673	-0.242249
H	1.536205	1.486507	-1.755028
H	2.736547	1.437713	-0.439941
H	1.239932	-2.374673	-0.242249
H	2.736547	-1.437713	-0.439941
H	1.536205	-1.486507	-1.755028
C	1.103055	0.000000	1.815522
H	0.651515	0.890059	2.257512
H	2.173993	0.000000	2.035642
H	0.651515	-0.890059	2.257512
H	-1.473126	2.370588	-0.095317
H	-1.779547	1.351513	1.344794
H	-2.977920	1.479427	0.048392
H	-1.473126	-2.370588	-0.095317
H	-2.977920	-1.479427	0.048392
H	-1.779547	-1.351513	1.344794

(Hartree/Particle)
HF=-882.0210375
Zero-point correction= 0.190771
Thermal correction to Energy= 0.203152
Thermal correction to Enthalpy= 0.204096
Thermal correction to Gibbs Free Energy= 0.153357
Sum of electronic and zero-point Energies= -881.830267
Sum of electronic and thermal Energies= -881.817885
Sum of electronic and thermal Enthalpies= -881.816941
Sum of electronic and thermal Free Energies= -881.867681

CCSD/6-311++G(d,p)

P	0.847246	0.000000	0.013276
P	-1.242317	0.000001	-0.724488
C	1.677501	1.465788	-0.654944
C	1.677517	-1.465757	-0.654993
C	-1.888356	1.428213	0.263722
C	-1.888355	-1.428217	0.263714
H	1.238884	2.376163	-0.235443
H	1.570427	1.483925	-1.743870
H	2.741279	1.433484	-0.397534
H	1.238898	-2.376151	-0.235537
H	2.741292	-1.433458	-0.397567
H	1.570458	-1.483848	-1.743922
C	1.057629	-0.000029	1.816629
H	0.597878	0.892209	2.251572
H	2.127046	-0.000019	2.054227
H	0.597902	-0.892296	2.251540
H	-1.451698	2.369236	-0.085129
H	-1.739509	1.325117	1.343235
H	-2.965604	1.484235	0.071886
H	-1.451683	-2.369235	-0.085131
H	-2.965599	-1.484250	0.071865
H	-1.739521	-1.325118	1.343229

(Hartree/Particle)
HF= -879.4386718

Basis Set

PBE1PBE/6-311+G(d,p)

P	0.850870	0.000000	0.014837
P	-1.236512	0.000000	-0.708113
C	1.664833	1.465179	-0.661299

C	1.664833	-1.465179	-0.661299
C	-1.887920	1.430356	0.260742
C	-1.887920	-1.430356	0.260742
H	1.225142	2.371086	-0.238848
H	1.545809	1.482701	-1.746903
H	2.729545	1.441271	-0.415800
H	1.225142	-2.371086	-0.238848
H	2.729545	-1.441271	-0.415800
H	1.545809	-1.482701	-1.746904
C	1.071750	0.000000	1.811869
H	0.613912	0.890347	2.247644
H	2.139521	0.000000	2.047714
H	0.613912	-0.890347	2.247644
H	-1.451234	2.365907	-0.095547
H	-1.757081	1.341733	1.341667
H	-2.960264	1.484104	0.051234
H	-1.451234	-2.365907	-0.095547
H	-2.960264	-1.484104	0.051234
H	-1.757081	-1.341733	1.341667

(Hartree/Particle)
HF=-881.6357724
Zero-point correction= 0.191355
Thermal correction to Energy= 0.203694
Thermal correction to Enthalpy= 0.204638
Thermal correction to Gibbs Free Energy= 0.153890
Sum of electronic and zero-point Energies= -881.444418
Sum of electronic and thermal Energies= -881.432078
Sum of electronic and thermal Enthalpies= -881.431134
Sum of electronic and thermal Free Energies= -881.481883

PBE1PBE/6-311G(d,p)

P	0.849713	0.000000	0.014627
P	-1.236305	0.000000	-0.710765
C	1.666363	1.465006	-0.658655
C	1.666363	-1.465006	-0.658655
C	-1.884347	1.430310	0.259963
C	-1.884347	-1.430310	0.259963
H	1.221283	2.371138	-0.242761
H	1.556734	1.479292	-1.745184
H	2.728839	1.443685	-0.404244
H	1.221283	-2.371138	-0.242762
H	2.728839	-1.443685	-0.404244
H	1.556734	-1.479292	-1.745184
C	1.063048	0.000000	1.812435
H	0.603457	0.890473	2.245749
H	2.129693	0.000000	2.052280
H	0.603457	-0.890473	2.245749
H	-1.450157	2.365847	-0.099115
H	-1.749326	1.343385	1.340396
H	-2.957476	1.482967	0.054902
H	-1.450157	-2.365847	-0.099115
H	-2.957476	-1.482967	0.054902
H	-1.749326	-1.343385	1.340396

(Hartree/Particle)
HF=-881.63381
Zero-point correction= 0.191509
Thermal correction to Energy= 0.203826
Thermal correction to Enthalpy= 0.204770
Thermal correction to Gibbs Free Energy= 0.154064
Sum of electronic and zero-point Energies= -881.442301
Sum of electronic and thermal Energies= -881.429984
Sum of electronic and thermal Enthalpies= -881.429040
Sum of electronic and thermal Free Energies= -881.479746

PBE1PBE/6-311++G(d)

P	0.849340	0.000000	0.015069
P	-1.236826	0.000000	-0.710183
C	1.665703	1.463698	-0.661194
C	1.665703	-1.463698	-0.661194
C	-1.888762	1.428448	0.260999
C	-1.888762	-1.428448	0.260999
H	1.233284	2.373195	-0.239658
H	1.547091	1.486314	-1.746418
H	2.731220	1.440066	-0.420440
H	1.233284	-2.373195	-0.239658
H	2.731220	-1.440066	-0.420440
H	1.547091	-1.486314	-1.746418
C	1.073351	0.000000	1.812012
H	0.618464	0.888838	2.253000
H	2.140464	0.000000	2.049408
H	0.618464	-0.888838	2.253000
H	-1.455635	2.367209	-0.090269
H	-1.758755	1.340301	1.341816
H	-2.961452	1.484549	0.055760
H	-1.455635	-2.367209	-0.090269
H	-2.961452	-1.484549	0.055760
H	-1.758755	-1.340301	1.341816

(Hartree/Particle)
HF=-881.6150451
Zero-point correction= 0.192423
Thermal correction to Energy= 0.204690
Thermal correction to Enthalpy= 0.205634
Thermal correction to Gibbs Free Energy= 0.155124
Sum of electronic and zero-point Energies= -881.422622
Sum of electronic and thermal Energies= -881.410355
Sum of electronic and thermal Enthalpies= -881.409411
Sum of electronic and thermal Free Energies= -881.459921

HF Optimizations and Frequency Analysis

Cartesian coordinates, enthalpies and Gibbs Energies for structures optimized at the HF/6-311++G(d,p) level in the gas phase (298 K) absence of a counterion or solvent, in the order: $[R_2PPMe_3]^+$, $[R(H)PPMe_3]^{+\cdot}$, $[R_2PPMe_2]^{+\cdot}$, $[R_2PPMe_2]$, $[RPPMe_3]^{+\cdot}$, $[RPPMe_3]$, $[RPPMe_2]^+$, $[R_2P]^+$, $[R_2P]^\cdot$, $[R]^+$, $[R]^\cdot$, alkanes, olefins, $[PMe_3]$ derivatives, miscellaneous fragments. Within each subsection the structures are in the order: R = Me, Et, ^{i}Pr, ^{t}Bu, Ph, N^{i}Pr$_2$.

$[R_2PPMe_3]^+$

$[Me_2PPMe_3]^+$

P	0.861410	0.000000	0.013841
P	-1.243265	0.000000	-0.692430
C	1.679746	1.469415	-0.666673
C	1.679747	-1.469414	-0.666676
C	-1.918833	1.437979	0.253482
C	-1.918833	-1.437979	0.253483
H	1.251612	2.369004	-0.241032
H	1.556400	1.491672	-1.742934
H	2.737242	1.438074	-0.429081
H	1.251612	-2.369004	-0.241038
H	2.737242	-1.438074	-0.429082
H	1.556403	-1.491668	-1.742937
C	1.095808	-0.000001	1.815564
H	0.645960	0.883184	2.252283
H	2.157408	-0.000003	2.038600
H	0.645958	-0.883186	2.252282
H	-1.481098	2.364871	-0.098053

H	-1.797284	1.356017	1.327137
H	-2.980530	1.482530	0.034262
H	-1.481097	-2.364871	-0.098052
H	-2.980529	-1.482531	0.034261
H	-1.797286	-1.356016	1.327137

(Hartree/Particle)
HF=-879.4402326
Zero-point correction= 0.202335
Thermal correction to Energy= 0.214095
Thermal correction to Enthalpy= 0.215039
Thermal correction to Gibbs Free Energy= 0.165650
Sum of electronic and zero-point Energies= -879.237898
Sum of electronic and thermal Energies= -879.226137
Sum of electronic and thermal Enthalpies= -879.225193
Sum of electronic and thermal Free Energies= -879.274583

$[Et_2PPMe_3]^+$

P	-1.272816	-0.318673	0.012315
P	0.818249	0.071672	-0.649094
C	-1.667802	-2.060386	-0.319008
C	-2.407445	0.679134	-0.994148
C	1.690054	-1.307715	0.249566
C	1.283553	1.558477	0.368235
H	-1.119014	-2.709066	0.351686
H	-1.415794	-2.308405	-1.343529
H	-2.730207	-2.217948	-0.169342
H	-2.248030	1.734952	-0.819598
H	-3.430935	0.428143	-0.738281
H	-2.238064	0.467238	-2.043100
C	-1.620834	0.010844	1.765478
H	-0.975161	-0.591146	2.393463
H	-2.654165	-0.239437	1.980479
H	-1.458707	1.057857	1.989483
H	1.280693	-2.243840	-0.114646
H	1.509464	-1.258837	1.319242
C	3.200256	-1.287666	-0.029520
C	0.630677	2.874783	-0.062365
H	2.356842	1.635300	0.223932
H	1.130193	1.371670	1.426412
H	3.405838	-1.332655	-1.093665
H	3.665028	-2.148070	0.437840
H	3.676285	-0.401146	0.373684
H	0.752810	3.051314	-1.125489
H	1.090930	3.700873	0.467104
H	-0.430262	2.903396	0.166584

(Hartree/Particle)
HF=-957.5248173
Zero-point correction= 0.263482
Thermal correction to Energy= 0.277816
Thermal correction to Enthalpy= 0.278760
Thermal correction to Gibbs Free Energy= 0.223025
Sum of electronic and zero-point Energies= -957.261335
Sum of electronic and thermal Energies= -957.247001
Sum of electronic and thermal Enthalpies= -957.246057
Sum of electronic and thermal Free Energies= -957.301792

$[^iPr_2PPMe_3]^+$

P	-1.584576	-0.071283	-0.039330
P	0.583703	0.054583	-0.593729
C	-2.308962	-1.665030	-0.538508
C	-2.463005	1.143261	-1.071624
C	1.484151	-1.360575	0.284867
C	1.171080	1.541504	0.407885
H	-2.074742	-2.447670	0.166429
H	-1.946762	-1.943303	-1.521058
H	-3.385513	-1.542434	-0.585020
H	-2.167074	2.156451	-0.847170
H	-3.528428	1.038115	-0.897956
H	-2.251075	0.937338	-2.114129
C	-2.031676	0.227784	1.698773
H	-1.567893	-0.513732	2.337139
H	-3.109097	0.165828	1.809256
H	-1.702453	1.212239	2.006234
C	0.664411	-2.585833	0.695283
H	1.900893	-0.925903	1.187023
C	2.636965	-1.799416	-0.635771
C	0.438944	2.844290	0.066157
C	2.679186	1.717995	0.157558
H	1.027137	1.320008	1.461736
H	2.254410	-2.297017	-1.521265
H	3.277584	-2.497265	-0.106045
H	3.251854	-0.970458	-0.964838
H	0.429149	3.032985	-1.003109
H	0.949161	3.675387	0.540406
H	-0.581821	2.860862	0.430509
H	-0.095463	-2.354654	1.434444
H	1.330060	-3.313247	1.147302
H	0.198282	-3.068294	-0.157081
H	2.885887	1.903540	-0.892244
H	3.261346	0.862900	0.475969
H	3.031087	2.574931	0.721649

(Hartree/Particle)
HF=-1035.5996826
Zero-point correction= 0.324163
Thermal correction to Energy= 0.340714
Thermal correction to Enthalpy= 0.341658
Thermal correction to Gibbs Free Energy= 0.281835
Sum of electronic and zero-point Energies= -1035.275519
Sum of electronic and thermal Energies= -1035.258969
Sum of electronic and thermal Enthalpies= -1035.258025
Sum of electronic and thermal Free Energies= -1035.317847

$[^tBu_2PPMe_3]^+$

P	-1.765838	-0.001140	-0.013249
P	0.368571	0.002094	-0.735083
C	-2.562060	1.610082	-0.308310
C	1.137116	-1.590379	0.002135
C	1.142908	1.591375	0.002608
C	-2.149242	-0.439607	1.714054
H	-2.420225	1.914635	-1.337279
H	-2.182829	2.375286	0.352755
H	-3.623858	1.485780	-0.124478
H	-1.845410	-1.454712	1.928984
H	-3.221329	-0.357496	1.859861
H	-1.647154	0.233410	2.396047
C	-2.713596	-1.133049	-1.080155
H	-2.507917	-0.901658	-2.118204
H	-3.769690	-0.982937	-0.884140
H	-2.465128	-2.166105	-0.892067
C	0.707297	2.751963	-0.915149
C	2.674823	1.478426	-0.130090
C	0.789659	1.919224	1.461018
C	0.192162	-2.770520	-0.287777
C	1.465716	-1.571877	1.501374
C	2.424847	-1.847205	-0.812335
H	-0.263988	2.141909	1.592493
H	1.333633	2.808398	1.766379
H	1.058435	1.124581	2.144356
H	1.003364	2.576143	-1.943061
H	1.189710	3.665307	-0.580448
H	-0.358943	2.932192	-0.900891
H	2.979067	1.214102	-1.137160
H	3.106541	0.770077	0.563128
H	3.105125	2.449843	0.092093

H	2.217131	-1.910784	-1.875060
H	2.847309	-2.796995	-0.498644
H	3.181885	-1.092201	-0.658760
H	-0.682561	-2.773469	0.353187
H	0.721847	-3.696212	-0.088993
H	-0.125743	-2.797300	-1.324934
H	0.593720	-1.382782	2.117106
H	2.220716	-0.839579	1.752016
H	1.857499	-2.544346	1.786466

(Hartree/Particle)
HF=-1113.6747363
Frequency calculation failure.

$[Ph_2PPMe_3]^+$

P	0.140771	2.066744	-0.210547
P	0.003559	0.202104	1.005542
C	0.395851	1.909342	-2.003410
C	1.543661	3.017056	0.440275
H	-0.497556	1.531424	-2.481932
H	1.225103	1.240951	-2.200513
H	0.623848	2.888304	-2.411818
H	1.430334	3.155275	1.508655
H	1.577438	3.986534	-0.044502
H	2.470086	2.491249	0.244153
C	-1.369489	3.030914	0.075235
H	-2.227324	2.501402	-0.320088
H	-1.283481	3.994225	-0.415385
H	-1.507539	3.184078	1.139060
H	4.711057	-1.030685	1.243218
C	3.798853	-1.200606	0.701034
C	2.644345	-0.530175	1.068177
H	2.672126	0.139740	1.910068
C	1.449102	-0.738679	0.379041
C	1.425856	-1.666416	-0.660430
H	0.514449	-1.887443	-1.179705
C	2.577720	-2.346287	-1.016408
H	2.544225	-3.067451	-1.812647
C	3.766301	-2.108372	-0.343987
H	4.657430	-2.640825	-0.622592
H	-2.232681	-0.693337	2.412858
C	-2.469659	-0.875330	1.379903
C	-1.550395	-0.544370	0.386826
C	-1.888987	-0.789989	-0.946779
H	-1.204435	-0.558213	-1.740710
C	-3.108661	-1.350379	-1.271280
H	-3.354843	-1.535960	-2.300988
C	-4.012242	-1.680272	-0.269199
H	-4.958971	-2.120323	-0.524786
C	-3.692014	-1.444967	1.054135
H	-4.385668	-1.700500	1.833926

(Hartree/Particle)
HF=-1260.5380122
Zero-point correction= 0.316853
Thermal correction to Energy= 0.334242
Thermal correction to Enthalpy= 0.335186
Thermal correction to Gibbs Free Energy= 0.270232
Sum of electronic and zero-point Energies= -1260.221159
Sum of electronic and thermal Energies= -1260.203770
Sum of electronic and thermal Enthalpies= -1260.202826
Sum of electronic and thermal Free Energies= -1260.267780

$[(^iPr_2N)_2PPMe_3]^+$

P	3.662343	-0.336580	0.176550
N	-0.924336	1.228673	-0.309216
C	4.444955	0.457191	1.649343
C	4.350709	-2.045966	0.296561
C	-1.364536	1.652998	1.059477
C	-0.302913	2.185897	-1.303653
C	4.698203	0.359946	-1.184742
H	4.439319	-0.116449	-2.125006
H	4.506157	1.423451	-1.287030
C	-2.184129	2.940828	1.091741
C	0.810757	3.055300	-0.728630
C	-1.355214	2.992954	-2.065221
H	0.169978	1.538423	-2.035403
H	-2.579589	3.059754	2.094981
H	-3.021077	2.911621	0.405656
H	-1.590505	3.818715	0.874601
H	0.447093	3.807352	-0.040839
H	1.284362	3.575654	-1.554370
H	1.563796	2.454168	-0.235645
H	-1.816815	3.759060	-1.457991
H	-2.134227	2.349021	-2.459912
H	-0.873193	3.484522	-2.903888
H	-3.753485	0.763666	0.271001
H	-2.778518	-0.528209	2.516981
C	-4.015399	-0.230003	-0.058606
H	-2.726867	-3.146580	-1.899933
H	-5.030500	-0.421422	0.271621
H	-4.016343	-0.248161	-1.143795
C	-3.121183	-1.430653	2.027591
N	-1.737697	-1.366433	-0.088506
H	0.448031	-2.647403	0.987017
C	-1.671916	-3.301642	-1.700521
C	-3.121538	-1.332659	0.501980
H	-4.133566	-1.623582	2.366624
H	-1.471388	-4.364965	-1.774834
H	-2.497766	-2.250383	2.366336
C	0.194191	-3.030106	0.004363
H	-1.099230	-2.801182	-2.475179
C	-1.282901	-2.803690	-0.310029
H	0.854560	-2.574692	-0.724903
H	0.388203	-4.097293	-0.001711
H	-1.844949	-3.360489	0.425111
P	-0.801746	-0.286724	-0.838935
H	-3.552487	-2.254282	0.134486
H	4.240424	1.523301	1.646508
H	5.521028	0.312403	1.667680
H	4.024355	0.040805	2.559239
H	5.432036	-2.044020	0.396274
H	4.087496	-2.612741	-0.591036
H	3.925869	-2.555158	1.155945
C	-0.194768	1.690791	2.048592
H	0.391150	0.779557	2.001931
H	-0.598225	1.781550	3.052301
H	0.465957	2.529762	1.884235
H	-2.011176	0.864052	1.387787
H	5.759460	0.216922	-1.004478

(Hartree/Particle)
HF=-1379.918741
Zero-point correction= 0.537868
Thermal correction to Energy= 0.565976
Thermal correction to Enthalpy= 0.566920
Thermal correction to Gibbs Free Energy= 0.474407
Sum of electronic and zero-point Energies= -1379.380873
Sum of electronic and thermal Energies= -1379.352765
Sum of electronic and thermal Enthalpies= -1379.351821
Sum of electronic and thermal Free Energies= -1379.444334

$[R(H)PPMe_3]^+$

$[Et(H)PPMe_3]^+$

P	1.094496	-0.046709	-0.008879
P	-0.896336	0.903172	0.134845
C	1.469678	-0.854114	1.571056
C	2.302149	1.278141	-0.282262
C	-2.043195	-0.561911	0.041736
H	0.777716	-1.668756	1.749278
H	1.387894	-0.135509	2.377868
H	2.478761	-1.249978	1.545511
H	2.112333	1.764902	-1.231777
H	3.302871	0.860684	-0.292912
H	2.229603	2.011173	0.512313
C	1.271337	-1.269286	-1.338893
H	0.597313	-2.101139	-1.176337
H	2.290486	-1.640689	-1.352911
H	1.047410	-0.810273	-2.294488
H	-1.911397	-1.135568	0.953706
H	-1.802505	-1.203134	-0.797355
C	-3.497875	-0.081714	-0.060024
H	-3.771877	0.537372	0.787110
H	-4.161503	-0.938185	-0.078746
H	-3.665834	0.487747	-0.968329
H	-0.896236	1.307715	-1.212101

(Hartree/Particle)
HF=-879.4321177
Zero-point correction= 0.202245
Thermal correction to Energy= 0.213732
Thermal correction to Enthalpy= 0.214676
Thermal correction to Gibbs Free Energy= 0.165132
Sum of electronic and zero-point Energies= -879.229872
Sum of electronic and thermal Energies= -879.218386
Sum of electronic and thermal Enthalpies= -879.217441
Sum of electronic and thermal Free Energies= -879.266985

[^{i}Pr(H)PPMe$_3$]$^+$

P	1.313240	0.014968	0.045820
P	-0.610713	-0.859438	-0.620366
C	1.776349	1.393077	-1.039017
C	2.566497	-1.284011	-0.144321
C	-1.920694	0.089060	0.328467
H	1.071053	2.208622	-0.942477
H	1.785312	1.054879	-2.068364
H	2.765204	1.747114	-0.769466
H	2.361373	-2.102442	0.535244
H	3.546278	-0.875632	0.077309
H	2.559884	-1.657012	-1.161764
C	1.347171	0.593076	1.766264
H	0.638620	1.400030	1.906274
H	2.342244	0.953380	2.004630
H	1.095439	-0.221897	2.434450
C	-2.126441	1.490098	-0.255984
H	-1.625046	0.169747	1.368540
C	-3.211215	-0.742549	0.255915
H	-3.545163	-0.868245	-0.769557
H	-3.997743	-0.231856	0.800937
H	-3.087781	-1.725553	0.698506
H	-1.250379	2.122627	-0.143674
H	-2.943065	1.983095	0.260155
H	-2.378435	1.447234	-1.311027
H	-0.465700	-1.969543	0.230532

(Hartree/Particle)
HF=-918.4752468
Zero-point correction= 0.232149
Thermal correction to Energy= 0.244834
Thermal correction to Enthalpy= 0.245779
Thermal correction to Gibbs Free Energy= 0.193847
Sum of electronic and zero-point Energies= -918.243098
Sum of electronic and thermal Energies= -918.230412
Sum of electronic and thermal Enthalpies= -918.229468
Sum of electronic and thermal Free Energies= -918.281400

[^{t}Bu(H)PPMe$_3$]$^+$

P	-1.505275	0.018167	0.001340
P	0.401256	-1.114666	-0.127630
C	-1.667614	1.205671	-1.361010
C	1.865444	0.070278	0.010317
C	-1.865247	0.875473	1.563179
H	-1.447049	0.719499	-2.303802
H	-0.991842	2.039592	-1.220732
H	-2.685642	1.578731	-1.384470
H	-1.782569	0.178539	2.388699
H	-2.878958	1.260550	1.526192
H	-1.179266	1.695587	1.721464
C	-2.786129	-1.250530	-0.214826
H	-2.652201	-1.748940	-1.167028
H	-3.765982	-0.786980	-0.184491
H	-2.719091	-1.985894	0.578738
C	2.154539	0.640788	-1.387702
C	3.029336	-0.848281	0.437331
C	1.703414	1.202434	1.030188
H	0.959097	1.928802	0.717540
H	2.642954	1.738495	1.124887
H	1.438855	0.831324	2.015203
H	2.309172	-0.145974	-2.118187
H	3.057277	1.242427	-1.351329
H	1.354639	1.281750	-1.745910
H	3.162529	-1.681978	-0.245285
H	2.887522	-1.243352	1.438328
H	3.950199	-0.273862	0.436150
H	0.298191	-1.555826	1.203532

(Hartree/Particle)
HF=-957.5153053
Zero-point correction= 0.261715
Thermal correction to Energy= 0.275505
Thermal correction to Enthalpy= 0.276449
Thermal correction to Gibbs Free Energy= 0.222604
Sum of electronic and zero-point Energies= -957.253590
Sum of electronic and thermal Energies= -957.239800
Sum of electronic and thermal Enthalpies= -957.238856
Sum of electronic and thermal Free Energies= -957.292701

[^{i}Pr$_2$N(H)PPMe$_3$]$^+$

P	-2.201014	-0.059812	-0.058159
N	0.968769	-0.160742	0.196390
C	-2.160165	-0.492662	-1.819275
C	-2.535603	1.721294	0.092976
C	1.909220	-1.148687	-0.428219
C	1.297999	1.270393	0.433304
C	-3.590979	-0.937881	0.714569
H	-4.521816	-0.641871	0.243775
H	-3.458448	-2.007302	0.602539
C	2.914257	-1.740880	0.563574
C	1.257350	2.137901	-0.830500
C	2.593036	1.494160	1.214944
H	0.499021	1.629944	1.072410
H	3.429635	-2.574314	0.096484
H	2.414905	-2.119597	1.450202
H	3.662259	-1.023539	0.874562
H	2.133780	2.014068	-1.450599
H	1.210901	3.183642	-0.541504
H	0.386574	1.918718	-1.440496
H	3.471638	1.242364	0.634074
H	2.606898	0.916272	2.132113

H	2.668809	2.544394	1.478170
P	-0.341061	-0.877496	0.929651
H	-2.036613	-1.563809	-1.927984
H	-1.331647	0.008981	-2.303903
H	-3.086643	-0.191389	-2.295217
H	-1.757502	2.296605	-0.390368
H	-2.582603	1.996220	1.140216
H	-3.487486	1.950203	-0.374687
C	2.598367	-0.641505	-1.692062
H	1.886990	-0.248499	-2.409278
H	3.113640	-1.475132	-2.157007
H	3.341724	0.118509	-1.485239
H	1.270855	-1.966757	-0.744403
H	-3.641267	-0.698997	1.770987
H	-0.544747	-0.096710	2.082174

(Hartree/Particle)
HF=-1090.6387263
Zero-point correction= 0.340619
Thermal correction to Energy= 0.358113
Thermal correction to Enthalpy= 0.359058
Thermal correction to Gibbs Free Energy= 0.296334
Sum of electronic and zero-point Energies= -1090.298108
Sum of electronic and thermal Energies= -1090.280613
Sum of electronic and thermal Enthalpies= -1090.279669
Sum of electronic and thermal Free Energies= -1090.342392

$[R_2PPMe_2]^{+\cdot}$

$[Me_2PPMe_2]^{+\cdot}$

P	-0.988178	0.000000	0.462182
P	0.988177	0.000000	-0.462180
C	-1.890640	-1.462923	-0.169629
C	1.890640	-1.462924	0.169627
C	1.890641	1.462924	0.169628
H	-2.016861	-1.419634	-1.244517
H	-2.868058	-1.470073	0.300666
H	-1.376243	-2.374447	0.106483
C	-1.890640	1.462923	-0.169629
H	-1.376244	2.374447	0.106482
H	-2.868058	1.470073	0.300666
H	-2.016861	1.419634	-1.244517
H	1.376244	-2.374447	-0.106485
H	2.016862	-1.419636	1.244515
H	2.868059	-1.470072	-0.300669
H	1.376244	2.374447	-0.106484
H	2.868059	1.470072	-0.300669
H	2.016863	1.419635	1.244515

(Hartree/Particle)
HF=-839.7616604
Zero-point correction= 0.160617
Thermal correction to Energy= 0.171127
Thermal correction to Enthalpy= 0.172071
Thermal correction to Gibbs Free Energy= 0.124092
Sum of electronic and zero-point Energies= -839.601043
Sum of electronic and thermal Energies= -839.590534
Sum of electronic and thermal Enthalpies= -839.589589
Sum of electronic and thermal Free Energies= -839.637568

$[Et_2PPMe_2]^{+\cdot}$

P	-1.690848	-0.052747	-0.488426
P	0.386764	-0.046123	0.186115
C	-2.417879	1.474654	0.235057
C	-2.487255	-1.422929	0.444325
C	1.288965	1.372889	-0.571287
C	1.193443	-1.625798	-0.299610
H	-1.919262	2.355230	-0.150190
H	-2.384887	1.475820	1.317154
H	-3.453537	1.514206	-0.085293
H	-2.057195	-2.375540	0.161993
H	-3.533228	-1.434429	0.156543
H	-2.421378	-1.289100	1.516756
H	0.547571	2.071042	-0.942234
H	1.828332	0.991940	-1.431869
C	2.235575	2.077779	0.411984
H	1.693755	2.493843	1.253907
H	2.735578	2.890848	-0.100442
H	2.996206	1.407283	0.794427
C	2.646569	-1.735265	0.182608
H	3.278562	-0.977067	-0.264892
H	3.039577	-2.702156	-0.106987
H	2.718689	-1.655652	1.261694
H	1.138844	-1.701525	-1.381415
H	0.597137	-2.429690	0.117046

(Hartree/Particle)
HF=-917.8482886
Zero-point correction= 0.222029
Thermal correction to Energy= 0.234980
Thermal correction to Enthalpy= 0.235924
Thermal correction to Gibbs Free Energy= 0.181653
Sum of electronic and zero-point Energies= -917.626260
Sum of electronic and thermal Energies= -917.613309
Sum of electronic and thermal Enthalpies= -917.612365
Sum of electronic and thermal Free Energies= -917.666636

$[^iPr_2PPMe_2]^{+\cdot}$

P	1.567433	-0.683658	-0.042122
P	-0.443392	0.082293	-0.636011
C	2.873259	-0.369567	-1.266896
C	-0.474922	1.662678	0.385683
C	-1.590454	-1.104704	0.271101
H	3.193594	0.665761	-1.214211
H	2.488023	-0.574201	-2.257404
H	3.719158	-1.015617	-1.063376
C	2.204263	-0.465734	1.650882
H	2.475522	0.572040	1.814605
H	3.082006	-1.087689	1.784174
H	1.447106	-0.756239	2.367805
C	0.550219	2.669938	-0.150196
H	-0.236731	1.420237	1.417410
C	-1.886035	2.270819	0.355861
C	-1.086666	-2.553300	0.304813
C	-2.956857	-1.051254	-0.437149
H	-1.703639	-0.747227	1.289946
H	-2.219186	2.460153	-0.660319
H	-1.869925	3.220660	0.879508
H	-2.616366	1.641796	0.848873
H	-0.830159	-2.914852	-0.687283
H	-1.871865	-3.195031	0.688949
H	-0.225997	-2.685414	0.953700
H	1.574385	2.312514	-0.081051
H	0.497182	3.585797	0.428293
H	0.353197	2.919409	-1.187775
H	-2.888555	-1.472936	-1.434762
H	-3.349736	-0.046215	-0.527270
H	-3.675472	-1.635723	0.127580

(Hartree/Particle)
HF=-995.923413
Zero-point correction= 0.281609
Thermal correction to Energy= 0.297026
Thermal correction to Enthalpy= 0.297970

Thermal correction to Gibbs Free Energy= 0.238902
Sum of electronic and zero-point Energies= -995.641804
Sum of electronic and thermal Energies= -995.626387
Sum of electronic and thermal Enthalpies= -995.625443
Sum of electronic and thermal Free Energies= -995.684511

[^{t}Bu$_2$PPMe$_2$]$^{+\cdot}$

P	-1.698991	-1.275990	-0.065905
P	0.134504	-0.009424	-0.362860
C	-2.886508	-0.578821	-1.294340
C	1.647107	-1.096362	-0.035690
C	0.191650	1.848105	0.009800
C	-2.385131	-0.701691	1.551221
H	-2.484670	-0.676067	-2.295960
H	-3.173236	0.446865	-1.111396
H	-3.774051	-1.201641	-1.237965
H	-1.689563	-0.917556	2.353244
H	-3.276030	-1.298023	1.724428
H	-2.664205	0.342569	1.579156
C	-1.186582	2.436481	-0.334619
C	1.245702	2.526977	-0.884324
C	0.507416	2.070475	1.499010
C	1.597019	-2.245716	-1.062027
C	1.550834	-1.657587	1.395993
C	2.962313	-0.324651	-0.211671
H	-0.233162	1.614382	2.147112
H	0.501315	3.138416	1.694914
H	1.485460	1.695224	1.774315
H	-1.442191	2.289166	-1.378283
H	-1.149953	3.505917	-0.156034
H	-1.978772	2.034314	0.282672
H	1.069343	2.333603	-1.937374
H	2.256137	2.234927	-0.641436
H	1.171185	3.599126	-0.732089
H	3.063336	0.093432	-1.206314
H	3.778657	-1.025030	-0.069014
H	3.085073	0.464447	0.519183
H	0.695752	-2.839038	-0.976544
H	2.442164	-2.901058	-0.877385
H	1.680121	-1.879302	-2.080084
H	0.664375	-2.265302	1.537868
H	1.566306	-0.875236	2.147256
H	2.410988	-2.296189	1.571095

(Hartree/Particle)
HF=-1074.0091604
Zero-point correction= 0.341507
Thermal correction to Energy= 0.359098
Thermal correction to Enthalpy= 0.360042
Thermal correction to Gibbs Free Energy= 0.297672
Sum of electronic and zero-point Energies= -1073.667654
Sum of electronic and thermal Energies= -1073.650063
Sum of electronic and thermal Enthalpies= -1073.649118
Sum of electronic and thermal Free Energies= -1073.711489

[Ph$_2$PPMe$_2$]$^{+\cdot}$

P	0.217476	2.578651	0.269398
P	-0.063583	0.463334	-0.409063
C	1.232549	3.247071	-1.121159
C	1.473559	2.339233	1.599235
H	0.659281	3.241116	-2.040254
H	2.171805	2.729011	-1.266773
H	1.446396	4.281831	-0.872433
H	1.032913	1.813284	2.437641
H	1.751740	3.331899	1.940820
H	2.364909	1.820776	1.269807
H	2.136677	-3.436641	1.660920
C	2.217229	-2.640855	0.943658
C	1.149713	-1.767042	0.766723
H	0.257801	-1.892217	1.351528
C	1.255750	-0.715884	-0.163230
C	2.438678	-0.560285	-0.909841
H	2.524717	0.224355	-1.639728
C	3.497293	-1.442786	-0.725757
H	4.394594	-1.325128	-1.304549
C	3.388490	-2.482548	0.199819
H	4.207886	-3.163195	0.339564
C	-1.961291	-1.531562	-0.517876
C	-2.744079	0.652635	0.247425
C	-4.034204	0.144576	0.341625
C	-3.256371	-2.020942	-0.419755
C	-1.684974	-0.187430	-0.167402
C	-4.295175	-1.189015	0.012986
H	-1.176815	-2.174176	-0.870523
H	-5.295372	-1.574042	0.086218
H	-3.457783	-3.041926	-0.686308
H	-4.831109	0.785532	0.670257
H	-2.559021	1.678744	0.500096

(Hartree/Particle)
HF= -1220.8741017
Zero-point correction= 0.271753
Thermal correction to Energy= 0.287866
Thermal correction to Enthalpy= 0.288810
Thermal correction to Gibbs Free Energy= 0.224961
Sum of electronic and zero-point Energies= -1220.602348
Sum of electronic and thermal Energies= -1220.586236
Sum of electronic and thermal Enthalpies= -1220.585292
Sum of electronic and thermal Free Energies= -1220.649141

[(N^{i}Pr$_2$)$_2$PPMe$_2$]$^{+\cdot}$

P	0.420137	2.373814	0.656691
N	-1.354004	-0.299064	-0.030502
C	-1.125825	3.354171	0.462834
C	-1.437045	-1.542739	0.790686
C	-2.535614	0.352109	-0.680605
C	1.535820	3.216693	-0.547148
H	2.467370	2.677154	-0.651465
H	1.080880	3.345853	-1.521416
C	-2.286005	-2.655256	0.178472
C	-3.726145	0.557288	0.253879
C	-2.947076	-0.322483	-1.993010
H	-2.186735	1.340550	-0.956344
H	-2.156335	-3.547192	0.782689
H	-1.970674	-2.889540	-0.830750
H	-3.343609	-2.425285	0.170515
H	-4.221151	-0.372546	0.502070
H	-4.453288	1.184893	-0.250317
H	-3.439520	1.049288	1.175020
H	-3.421979	-1.281049	-1.839170
H	-2.092465	-0.461635	-2.646348
H	-3.657175	0.317875	-2.506760
P	0.084485	0.407009	-0.408041
H	-1.424289	3.496014	-0.568394
H	-0.912915	4.328878	0.891946
H	-1.935754	2.917751	1.030690
C	-1.831976	-1.250248	2.241630
H	-1.237191	-0.444127	2.659072
H	-1.655952	-2.138207	2.840730
H	-2.876909	-0.989925	2.344007
H	-0.427295	-1.913354	0.806226
H	1.762512	4.195347	-0.136069
N	1.398524	-0.528130	-0.079216
C	2.014482	-0.658773	1.279755
C	2.093242	-1.204003	-1.222319
C	1.243052	-2.308726	-1.846736
H	1.815676	-2.827415	-2.608314

H	0.353324	-1.909286	-2.326427
H	0.936061	-3.036506	-1.104500
C	2.604415	-0.219221	-2.274048
H	3.246705	0.531628	-1.828226
H	1.793396	0.282871	-2.794693
H	3.183308	-0.753319	-3.019940
H	2.959161	-1.669805	-0.778216
C	2.200190	-2.115088	1.716368
H	2.959555	-2.626655	1.135978
H	1.284302	-2.688790	1.654973
H	2.526755	-2.127033	2.750809
C	3.336152	0.102595	1.380035
H	3.732250	0.014141	2.386184
H	3.208117	1.156944	1.167945
H	4.081628	-0.295587	0.699447
H	1.315259	-0.201827	1.968558

(Hartree/Particle)
HF= -1340.2790552
Zero-point correction= 0.499009
Thermal correction to Energy= 0.523917
Thermal correction to Enthalpy= 0.524861
Thermal correction to Gibbs Free Energy= 0.445493
Sum of electronic and zero-point Energies= -1339.780046
Sum of electronic and thermal Energies= -1339.755138
Sum of electronic and thermal Enthalpies= -1339.754194
Sum of electronic and thermal Free Energies= -1339.833562

[R_2PPMe_2]

[Me_2PPMe_2]

P	-0.992419	-0.494976	-0.499565
P	0.992419	0.494976	-0.499565
C	-2.096414	0.993602	-0.504734
C	2.096414	-0.993602	-0.504734
C	1.265512	1.113763	1.227672
H	-1.915663	1.664797	0.329367
H	-3.129394	0.662823	-0.461611
H	-1.958219	1.540707	-1.430357
C	-1.265511	-1.113763	1.227672
H	-0.552536	-1.898196	1.458384
H	-2.258353	-1.550809	1.278662
H	-1.192472	-0.337239	1.981402
H	1.958219	-1.540707	-1.430357
H	1.915663	-1.664797	0.329366
H	3.129394	-0.662823	-0.461611
H	0.552536	1.898195	1.458385
H	2.258353	1.550809	1.278662
H	1.192472	0.337239	1.981402

(Hartree/Particle)
HF=-839.996624
Zero-point correction= 0.161051
Thermal correction to Energy= 0.170874
Thermal correction to Enthalpy= 0.171818
Thermal correction to Gibbs Free Energy= 0.126773
Sum of electronic and zero-point Energies= -839.835573
Sum of electronic and thermal Energies= -839.825750
Sum of electronic and thermal Enthalpies= -839.824806
Sum of electronic and thermal Free Energies= -839.869851

[Et_2PPMe_2]

P	-1.612447	0.008263	-0.576588
P	0.357800	-0.003938	0.468894
C	-2.405084	1.448088	0.284763
C	-2.455265	-1.383879	0.314388
C	1.211191	1.343700	-0.507542
C	1.066096	-1.555773	-0.286872
H	-1.944531	2.376630	-0.034617
H	-2.337537	1.376079	1.365511
H	-3.451722	1.488524	-0.001193
H	-2.068652	-2.337016	-0.028308
H	-3.513377	-1.355272	0.072710
H	-2.341135	-1.321753	1.392265
H	0.482218	2.129375	-0.683053
H	1.492243	0.963437	-1.485732
C	2.422444	1.950855	0.208301
H	2.133478	2.383641	1.160843
H	2.866317	2.736620	-0.397117
H	3.191953	1.212040	0.405288
C	2.526982	-1.822807	0.088002
H	3.191272	-1.073529	-0.329695
H	2.843582	-2.787967	-0.297007
H	2.664821	-1.830824	1.165007
H	0.957297	-1.526681	-1.367867
H	0.455298	-2.379279	0.072119

(Hartree/Particle)
HF=-918.0789731
Zero-point correction= 0.221834
Thermal correction to Energy= 0.234358
Thermal correction to Enthalpy= 0.235302
Thermal correction to Gibbs Free Energy= 0.183110
Sum of electronic and zero-point Energies= -917.857139
Sum of electronic and thermal Energies= -917.844615
Sum of electronic and thermal Enthalpies= -917.843671
Sum of electronic and thermal Free Energies= -917.895863

[$^{i}Pr_2PPMe_2$]

P	1.508585	-1.094387	-0.052491
P	-0.319814	0.055384	-0.603107
C	2.764581	-0.393262	-1.220717
C	-0.285367	1.657517	0.390625
C	-1.715017	-0.903696	0.244972
H	2.948724	0.667659	-1.105786
H	2.440179	-0.581613	-2.237825
H	3.698953	-0.924349	-1.065215
C	2.115081	-0.453225	1.579493
H	2.294050	0.615504	1.599619
H	3.045787	-0.961574	1.814068
H	1.401277	-0.703578	2.357504
C	0.785033	2.611242	-0.153872
H	-0.046648	1.420686	1.424105
C	-1.645867	2.368763	0.376269
C	-1.459113	-2.405376	0.419368
C	-3.011202	-0.709849	-0.559666
H	-1.852859	-0.474115	1.234266
H	-1.966427	2.588600	-0.638623
H	-1.566265	3.313365	0.908618
H	-2.423348	1.790390	0.859986
H	-1.201647	-2.880721	-0.522362
H	-2.362857	-2.879984	0.794365
H	-0.659195	-2.613531	1.118520
H	1.784256	2.202615	-0.077717
H	0.771564	3.543197	0.405854
H	0.600388	2.847270	-1.198162
H	-2.933041	-1.202761	-1.524412
H	-3.240925	0.332143	-0.745583
H	-3.852310	-1.146855	-0.026076

(Hartree/Particle)
HF=-996.1541625
Zero-point correction= 0.282104
Thermal correction to Energy= 0.296882
Thermal correction to Enthalpy= 0.297827

Thermal correction to Gibbs Free Energy= 0.241475
Sum of electronic and zero-point Energies= -995.872059
Sum of electronic and thermal Energies= -995.857280
Sum of electronic and thermal Enthalpies= -995.856336
Sum of electronic and thermal Free Energies= -995.912687

[^{t}Bu$_2$PPMe$_2$]

P	-1.514232	-1.306797	-0.058530
P	0.157552	0.009011	-0.724985
C	-2.894495	-0.804356	-1.186804
C	1.651706	-0.957845	-0.023105
C	0.050355	1.768432	0.015416
C	-2.202546	-0.802404	1.590167
H	-2.574283	-0.929544	-2.214598
H	-3.236557	0.213746	-1.048194
H	-3.729822	-1.476067	-1.011932
H	-1.463968	-0.959055	2.367011
H	-3.040618	-1.462153	1.797451
H	-2.556726	0.219469	1.640282
C	-1.351586	2.310115	-0.319758
C	1.055120	2.659199	-0.744997
C	0.295021	1.911960	1.524162
C	1.788981	-2.202775	-0.923324
C	1.529976	-1.424929	1.436868
C	2.942811	-0.136388	-0.176502
H	-0.377311	1.297414	2.109721
H	0.135599	2.947688	1.820181
H	1.311242	1.654997	1.797457
H	-1.579867	2.200624	-1.375396
H	-1.391975	3.370612	-0.082256
H	-2.131304	1.821742	0.249238
H	0.889059	2.612175	-1.816189
H	2.084813	2.389994	-0.553933
H	0.925809	3.693202	-0.430955
H	3.057501	0.253034	-1.183106
H	3.797149	-0.778145	0.026616
H	2.992039	0.691683	0.520313
H	0.906583	-2.830052	-0.885918
H	2.635784	-2.798335	-0.587879
H	1.962358	-1.923290	-1.957423
H	0.704926	-2.116084	1.566647
H	1.399581	-0.601106	2.127735
H	2.438121	-1.951811	1.725114

(Hartree/Particle)
HF=-1074.2278151
Zero-point correction= 0.341654
Thermal correction to Energy= 0.358595
Thermal correction to Enthalpy= 0.359539
Thermal correction to Gibbs Free Energy= 0.300160
Sum of electronic and zero-point Energies= -1073.886161
Sum of electronic and thermal Energies= -1073.869220
Sum of electronic and thermal Enthalpies= -1073.868276
Sum of electronic and thermal Free Energies= -1073.927655

[Ph$_2$PPMe$_2$]

P	0.288137	2.460113	0.091310
P	-0.003439	0.559496	-1.035426
C	1.918207	2.963535	-0.627089
C	0.797094	2.001442	1.810879
H	1.803004	3.162732	-1.686399
H	2.693232	2.217169	-0.487574
H	2.232962	3.883587	-0.144492
H	-0.033343	1.538887	2.332690
H	1.049968	2.917113	2.338205
H	1.649546	1.333187	1.839549
H	1.831107	-2.738681	2.260835
C	1.987637	-2.214786	1.334409
C	1.025692	-1.333914	0.873918
H	0.129450	-1.188882	1.448425
C	1.202466	-0.646690	-0.326992
C	2.369008	-0.875744	-1.051801
H	2.515608	-0.375912	-1.993247
C	3.340860	-1.750106	-0.586562
1	4.234439	-1.911852	-1.162992
C	3.151045	-2.422317	0.607095
H	3.897723	-3.107739	0.967060
C	-1.984351	-1.380801	-0.776799
C	-2.574767	0.704702	0.230093
C	-3.820357	0.186943	0.560518
C	-3.220784	-1.898916	-0.441766
C	-1.632892	-0.073244	-0.434281
C	-4.146880	-1.114659	0.230996
H	-1.284259	-2.002069	-1.307277
H	-5.111143	-1.515044	0.488869
H	-3.465019	-2.910951	-0.712445
H	-4.531217	0.808475	1.075879
H	-2.354396	1.723175	0.488944

(Hartree/Particle)
HF= -1221.0885695
Zero-point correction= 0.275354
Thermal correction to Energy= 0.290845
Thermal correction to Enthalpy= 0.291789
Thermal correction to Gibbs Free Energy= 0.230583
Sum of electronic and zero-point Energies= -1220.813215
Sum of electronic and thermal Energies= -1220.797724
Sum of electronic and thermal Enthalpies= -1220.796780
Sum of electronic and thermal Free Energies= -1220.857986

[(N^{i}Pr$_2$)$_2$PPMe$_2$]

P	0.337506	2.435781	-0.422565
N	-1.303347	-0.383835	-0.039800
6	-0.385847	2.972724	1.202380
6	-1.490900	-0.406739	1.418256
6	-2.259089	-1.014756	-0.979113
6	-0.998929	3.054442	-1.545772
H	-0.818898	2.707572	-2.556599
H	-1.990461	2.744184	-1.235861
C	-1.223509	-1.766419	2.087753
C	-3.485876	-0.152807	-1.306982
C	-2.693314	-2.440276	-0.619957
H	-1.708036	-1.099870	-1.907074
H	-1.035651	-1.615072	3.148343
H	-0.359035	-2.249881	1.654433
H	-2.066171	-2.440902	2.005854
H	-4.164277	-0.049082	-0.468820
H	-4.041511	-0.610070	-2.122464
H	-3.186064	0.837801	-1.627140
H	-3.372573	-2.471169	0.224772
H	-1.841918	-3.073916	-0.402668
H	-3.220471	-2.866728	-1.469109
P	0.103783	0.238872	-0.791172
H	-1.401273	2.631030	1.355310
H	-0.387578	4.059330	1.202737
H	0.228851	2.651333	2.034801
C	-2.840085	0.140718	1.900317
H	-3.072129	1.098395	1.450517
H	-2.808607	0.270904	2.979129
H	-3.655244	-0.540729	1.683576
H	-0.736678	0.266126	1.804048
H	-0.964311	4.139808	-1.551668
N	1.508813	-0.409082	-0.066445
C	2.183013	0.116917	1.131643
C	2.282171	-1.380828	-0.870027
C	1.515013	-2.682787	-1.109867
H	2.146663	-3.400356	-1.627566

H	0.635062	-2.516873	-1.723534
H	1.198257	-3.124406	-0.170978
C	2.816502	-0.811324	-2.189713
H	3.369620	0.106304	-2.023836
H	2.011850	-0.598697	-2.884446
H	3.484532	-1.530201	-2.658690
H	3.145486	-1.639021	-0.270835
C	2.447246	-0.950730	2.201219
H	3.161434	-1.697113	1.867377
H	1.537004	-1.459881	2.486801
H	2.864995	-0.481982	3.088653
C	3.478505	0.878628	0.818776
H	3.861776	1.339889	1.725712
H	3.306104	1.658570	0.088470
H	4.252797	0.216748	0.441756
H	1.499455	0.828008	1.575282

(Hartree/Particle)
HF=-1340.4753555
Zero-point correction= 0.497924
Thermal correction to Energy= 0.522526
Thermal correction to Enthalpy= 0.523470
Thermal correction to Gibbs Free Energy= 0.445465
Sum of electronic and zero-point Energies= -1339.977431
Sum of electronic and thermal Energies= -1339.952829
Sum of electronic and thermal Enthalpies= -1339.951885
Sum of electronic and thermal Free Energies= -1340.029891

$[RPPMe_3]^{+\cdot}$

$[MePPMe_3]^{+\cdot}$

P	-0.629835	-0.031986	0.000000
P	1.360602	0.946425	0.000000
C	-1.865191	1.293779	0.000003
C	-0.882066	-1.061150	1.474628
C	2.450390	-0.550386	0.000000
H	-1.742673	1.911560	-0.881952
H	-1.742672	1.911556	0.881961
H	-2.861627	0.866323	0.000003
H	-0.183768	-1.888975	1.478781
H	-1.893084	-1.453859	1.468357
H	-0.738066	-0.466727	2.369092
C	-0.882068	-1.061145	-1.474631
H	-0.738072	-0.466718	-2.369093
H	-1.893086	-1.453855	-1.468359
H	-0.183769	-1.888968	-1.478789
H	2.307964	-1.160559	0.885257
H	3.472986	-0.192396	0.000001
H	2.307965	-1.160558	-0.885258

(Hartree/Particle)
HF=-839.7872337
Zero-point correction= 0.161432
Thermal correction to Energy= 0.171571
Thermal correction to Enthalpy= 0.172515
Thermal correction to Gibbs Free Energy= 0.125556
Sum of electronic and zero-point Energies= -839.625801
Sum of electronic and thermal Energies= -839.615663
Sum of electronic and thermal Enthalpies= -839.614718
Sum of electronic and thermal Free Energies= -839.661678

$[EtPPMe_3]^{+\cdot}$

P	1.074580	-0.036882	0.000000
P	-0.902940	0.965568	0.000002
C	1.319842	-1.068398	1.474583
C	2.327269	1.273172	0.000012
C	-2.026296	-0.518896	0.000002
H	0.616208	-1.891550	1.478775
H	1.179156	-0.472935	2.368877
H	2.328470	-1.467219	1.468716
H	2.212397	1.892644	-0.881753
H	3.318202	0.833065	0.000010
H	2.212395	1.892631	0.881785
C	1.319847	-1.068377	-1.474597
H	0.616213	-1.891528	-1.478804
H	2.328476	-1.467199	-1.468731
H	1.179167	-0.472900	-2.368883
H	-1.822869	-1.127523	0.876295
H	-1.822866	-1.127527	-0.876287
C	-3.494435	-0.071728	-0.000002
H	-3.728659	0.518761	0.879092
H	-4.139603	-0.942404	-0.000001
H	-3.728656	0.518758	-0.879099

(Hartree/Particle)
HF=-878.831567
Zero-point correction= 0.191874
Thermal correction to Energy= 0.203323
Thermal correction to Enthalpy= 0.204268
Thermal correction to Gibbs Free Energy= 0.153589
Sum of electronic and zero-point Energies= -878.639693
Sum of electronic and thermal Energies= -878.628244
Sum of electronic and thermal Enthalpies= -878.627299
Sum of electronic and thermal Free Energies= -878.677978

$[^{i}Pr\ PPMe_3]^{+\cdot}$

P	1.307702	0.002519	0.053210
P	-0.581054	-0.532965	-0.969933
C	1.388869	1.748289	0.546183
C	2.654762	-0.323096	-1.115162
C	-1.864713	0.040858	0.279284
H	0.617508	1.970949	1.272816
H	1.258143	2.383988	-0.321339
H	2.358872	1.947962	0.988828
H	2.639865	-1.364918	-1.413105
H	3.607417	-0.099238	-0.648152
H	2.535499	0.295679	-1.996749
C	1.572269	-1.022876	1.529133
H	0.790457	-0.845009	2.257277
H	2.530703	-0.774584	1.972095
H	1.569097	-2.070592	1.252983
C	-2.427564	1.409806	-0.135100
H	-1.412952	0.128375	1.262467
C	-2.970446	-1.023874	0.348212
H	-3.437311	-1.171339	-0.621102
H	-3.740588	-0.700959	1.040733
H	-2.592078	-1.981346	0.690077
H	-1.665338	2.181785	-0.163674
H	-3.185691	1.718791	0.577040
H	-2.892376	1.362504	-1.114644

(Hartree/Particle)
HF=-917.8739293
Zero-point correction= 0.221958
Thermal correction to Energy= 0.234579
Thermal correction to Enthalpy= 0.235523
Thermal correction to Gibbs Free Energy= 0.182613
Sum of electronic and zero-point Energies= -917.651972
Sum of electronic and thermal Energies= -917.639351
Sum of electronic and thermal Enthalpies= -917.638406
Sum of electronic and thermal Free Energies= -917.691316

[^{t}BuPPMe$_3$]$^{+\cdot}$

P	-1.484296	0.004178	-0.000878
P	0.409576	-1.170217	-0.021422
C	-1.600203	1.402330	-1.155996
C	1.851397	0.053007	0.000311
C	-1.906787	0.597907	1.664610
H	-1.367539	1.073794	-2.161539
H	-0.921983	2.195156	-0.870806
H	-2.615904	1.783208	-1.135887
H	-1.873193	-0.228183	2.365050
H	-2.910583	1.008808	1.649287
H	-1.213266	1.363678	1.984762
C	-2.752276	-1.201917	-0.483524
H	-2.561023	-1.556226	-1.489515
H	-3.731162	-0.736829	-0.448045
H	-2.734093	-2.047231	0.194189
C	2.067945	0.641518	-1.404364
C	3.059054	-0.832135	0.373610
C	1.695922	1.169967	1.040862
H	0.909328	1.870782	0.778485
H	2.619122	1.738725	1.094790
H	1.498332	0.779127	2.034335
H	2.189414	-0.134937	-2.152567
H	2.974229	1.239914	-1.403378
H	1.253827	1.287652	-1.713902
H	3.195141	-1.648536	-0.328837
H	2.958967	-1.253998	1.368583
H	3.960862	-0.228385	0.356436

(Hartree/Particle)
HF=-956.9143468
Zero-point correction= 0.251646
Thermal correction to Energy= 0.265347
Thermal correction to Enthalpy= 0.266291
Thermal correction to Gibbs Free Energy= 0.212028
Sum of electronic and zero-point Energies= -956.662701
Sum of electronic and thermal Energies= -956.649000
Sum of electronic and thermal Enthalpies= -956.648056
Sum of electronic and thermal Free Energies= -956.702319

[PhPPMe$_3$]$^{+\cdot}$

P	-1.887648	-0.000001	0.216328
P	-0.495956	0.000006	-1.524305
C	-1.671763	1.472116	1.255006
C	-1.671766	-1.472127	1.254993
H	-1.829558	2.367643	0.665691
H	-0.672332	1.486810	1.671032
H	-2.395346	1.447304	2.062753
H	-1.829562	-2.367648	0.665670
H	-2.395348	-1.447320	2.062740
H	-0.672335	-1.486826	1.671020
C	-3.576683	0.000004	-0.443892
H	-3.734312	0.881775	-1.053980
H	-4.290681	0.000001	0.372118
H	-3.734313	-0.881762	-1.053988
H	3.467234	-2.132715	0.502973
C	2.979327	-1.201054	0.282158
C	1.736446	-1.203690	-0.327318
H	1.278455	-2.143218	-0.584118
C	1.098401	0.000002	-0.622094
C	1.736448	1.203692	-0.327312
H	1.278458	2.143222	-0.584108
C	2.979329	1.201052	0.282164
H	3.467236	2.132711	0.502983
C	3.598525	-0.000002	0.590076
H	4.566875	-0.000004	1.056188

(Hartree/Particle)
HF=-1030.3392258
Zero-point correction= 0.218752
Thermal correction to Energy= 0.231627
Thermal correction to Enthalpy= 0.232571
Thermal correction to Gibbs Free Energy= 0.177183
Sum of electronic and zero-point Energies= -1030.120474
Sum of electronic and thermal Energies= -1030.107599
Sum of electronic and thermal Enthalpies= -1030.106655
Sum of electronic and thermal Free Energies= -1030.162043

[^{i}Pr$_2$NPPMe$_3$]$^{+\cdot}$

P	-2.917066	-0.157287	-0.087359
N	1.292221	-0.310562	0.194935
C	-2.320783	1.023595	-1.402753
C	-4.700146	-0.306988	-0.534398
C	2.373387	-1.111378	-0.597631
C	1.657408	1.068846	0.798636
C	-2.920782	-0.397668	1.794652
H	-2.876611	-1.480059	1.956880
H	-2.107353	0.125275	2.290903
C	3.523647	-1.628353	0.265985
C	1.222286	2.272909	-0.046975
C	3.140749	1.239116	1.060964
H	1.158396	1.083109	1.785123
H	4.089629	-2.448890	-0.212090
H	3.141098	-2.031251	1.221472
H	4.258741	-0.841990	0.486925
H	1.596565	2.160295	-1.081077
H	1.618725	3.225743	0.341633
H	0.148027	2.371817	-0.071825
H	3.719217	1.159907	0.122262
H	3.497177	0.495644	1.782511
H	3.357857	2.231112	1.488598
P	-0.093238	-0.840821	0.309840
H	-1.306241	1.292772	-1.267700
H	-2.981213	1.900968	-1.329642
H	-2.501464	0.488237	-2.341895
H	-5.227044	0.637782	-0.320372
H	-5.183282	-1.098296	0.042444
H	-4.807116	-0.543548	-1.607107
C	2.917339	-0.336894	-1.807268
H	2.113173	-0.100811	-2.490478
H	3.631017	-0.982264	-2.359545
H	3.453087	0.588692	-1.550353
H	1.851407	-2.000330	-0.912009
H	-3.893406	-0.027474	2.136336

Convergence failure.

Item	Value	Threshold	Converged?
Maximum Force	0.034352	0.000002	NO
RMS Force	0.004664	0.000001	NO
Maximum Displacement	1.526727	0.000006	NO
RMS Displacement	0.485678	0.000004	NO

Predicted change in Energy=-3.801954D-02

(Hartree/Particle)
HF = -1089.87020783

[RPPMe$_3$]

[MePPMe$_3$]

P	-0.549123	0.001129	0.000000
P	1.353823	0.939295	0.000000
C	-1.817238	1.306406	-0.000001
C	-0.956795	-1.057090	1.435828

C	2.433627	-0.610131	-0.000001
H	-1.696583	1.926135	-0.879998
H	-1.696580	1.926141	0.879991
H	-2.811090	0.871635	0.000002
H	-0.253781	-1.880263	1.493224
H	-1.962414	-1.458865	1.355857
H	-0.868731	-0.467922	2.340282
C	-0.956795	-1.057092	-1.435827
H	-0.868730	-0.467925	-2.340281
H	-1.962414	-1.458866	-1.355855
H	-0.253782	-1.880266	-1.493221
H	2.314301	-1.228720	0.884592
H	3.458195	-0.251285	0.000001
H	2.314302	-1.228718	-0.884595

(Hartree/Particle)
HF=-839.9705354
Zero-point correction= 0.161203
Thermal correction to Energy= 0.171160
Thermal correction to Enthalpy= 0.172104
Thermal correction to Gibbs Free Energy= 0.126670
Sum of electronic and zero-point Energies= -839.809332
Sum of electronic and thermal Energies= -839.799375
Sum of electronic and thermal Enthalpies= -839.798431
Sum of electronic and thermal Free Energies= -839.843866

[EtPPMe$_3$]

P	0.992071	-0.006128	0.000000
P	-0.887982	0.974996	0.000000
C	1.379826	-1.072115	1.435993
C	2.288004	1.271961	-0.000001
C	-2.012948	-0.550515	-0.000001
H	0.664199	-1.884343	1.492041
H	1.299890	-0.482066	2.340624
H	2.379255	-1.489291	1.357009
H	2.180649	1.894167	-0.879944
H	3.272151	0.815566	0.000001
H	2.180648	1.894169	0.879941
C	1.379826	-1.072116	-1.435992
H	0.664199	-1.884345	-1.492039
H	2.379255	-1.489292	-1.357008
H	1.299889	-0.482068	-2.340623
H	-1.841387	-1.169911	0.876601
H	-1.841388	-1.169909	-0.876603
C	-3.476725	-0.099063	0.000001
H	-3.700339	0.500682	0.877490
H	-4.145898	-0.955972	0.000000
H	-3.700340	0.500684	-0.877488

(Hartree/Particle)
HF=-879.0126173
Zero-point correction= 0.191631
Thermal correction to Energy= 0.202881
Thermal correction to Enthalpy= 0.203825
Thermal correction to Gibbs Free Energy= 0.154712
Sum of electronic and zero-point Energies= -878.820987
Sum of electronic and thermal Energies= -878.809737
Sum of electronic and thermal Enthalpies= -878.808793
Sum of electronic and thermal Free Energies= -878.857905

[^{i}PrPPMe$_3$]

P	1.227344	-0.012111	0.026160
P	-0.560700	-0.773996	-0.820836
C	1.520571	1.794694	-0.012087
C	2.610481	-0.723194	-0.920467
C	-1.863677	0.083066	0.274321
H	0.780039	2.300003	0.597166
H	1.424680	2.145214	-1.032338
H	2.509369	2.036095	0.366611
H	2.557278	-1.804028	-0.879488
H	3.559321	-0.393497	-0.510718
H	2.538119	-0.411416	-1.955058
C	1.577192	-0.452877	1.767901
H	0.799391	-0.058358	2.411508
H	2.534663	-0.053383	2.088754
H	1.581212	-1.531921	1.859082
C	-2.357809	1.414668	-0.304250
H	-1.450498	0.274112	1.262956
C	-3.042461	-0.882324	0.457690
H	-3.464183	-1.167463	-0.503696
H	-3.835988	-0.415897	1.038921
H	-2.734041	-1.790213	0.964017
H	-1.552162	2.132607	-0.417330
H	-3.117497	1.859903	0.338419
H	-2.795145	1.265633	-1.287323

(Hartree/Particle)
HF=-918.0541955
Zero-point correction= 0.221444
Thermal correction to Energy= 0.233840
Thermal correction to Enthalpy= 0.234784
Thermal correction to Gibbs Free Energy= 0.183082
Sum of electronic and zero-point Energies= -917.832752
Sum of electronic and thermal Energies= -917.820356
Sum of electronic and thermal Enthalpies= -917.819411
Sum of electronic and thermal Free Energies= -917.871114

[^{t}BuPPMe$_3$]

P	-1.407997	-0.024832	0.000436
P	0.373413	-1.193880	0.015677
C	-1.666414	1.226972	-1.310630
C	1.830884	0.048084	0.003307
C	-1.909057	0.857506	1.527292
H	-1.478819	0.772758	-2.275514
H	-0.982413	2.056474	-1.178072
H	-2.683545	1.605811	-1.278083
H	-1.910292	0.150664	2.348063
H	-2.899962	1.288136	1.415817
H	-1.204816	1.646493	1.756824
C	-2.747199	-1.234459	-0.256470
H	-2.640351	-1.688566	-1.233237
H	-3.714330	-0.747971	-0.185186
H	-2.680069	-2.011174	0.495372
C	2.097920	0.615785	-1.400385
C	3.041876	-0.811063	0.413460
C	1.703192	1.207327	1.001140
H	0.924357	1.908110	0.709907
H	2.633303	1.773619	1.043312
H	1.482584	0.849506	2.001395
H	2.235418	-0.181045	-2.122991
H	2.997957	1.231866	-1.398573
H	1.278520	1.236115	-1.749748
H	3.163759	-1.665526	-0.246554
H	2.935322	-1.185513	1.426477
H	3.954928	-0.219984	0.362830

(Hartree/Particle
HF=-957.0933952
Zero-point correction= 0.251166)
Thermal correction to Energy= 0.264609
Thermal correction to Enthalpy= 0.265553
Thermal correction to Gibbs Free Energy= 0.212547
Sum of electronic and zero-point Energies= -956.842229
Sum of electronic and thermal Energies= -956.828786
Sum of electronic and thermal Enthalpies= -956.827842

Sum of electronic and thermal Free Energies= -956.880848

[PhPPMe$_3$]

P	-1.813003	0.000000	0.173404
P	-0.556701	0.000000	-1.546578
C	-1.689314	1.436596	1.295559
C	-1.689313	-1.436595	1.295560
H	-1.888524	2.340611	0.733159
H	-0.685064	1.494299	1.698299
H	-2.397125	1.352130	2.114685
H	-1.888522	-2.340611	0.733160
H	-2.397125	-1.352131	2.114685
H	-0.685063	-1.494296	1.698302
C	-3.537099	-0.000001	-0.412201
H	-3.712833	0.880073	-1.018688
H	-4.223854	0.000000	0.427467
H	-3.712832	-0.880076	-1.018686
H	3.459838	-2.131589	0.507027
C	2.977262	-1.196287	0.282104
C	1.733671	-1.192842	-0.331510
H	1.267434	-2.127918	-0.585820
C	1.077286	0.000000	-0.640434
C	1.733671	1.192842	-0.331510
H	1.267434	2.127919	-0.585819
C	2.977262	1.196286	0.282105
H	3.459838	2.131589	0.507027
C	3.602067	0.000000	0.594726
H	4.568986	0.000000	1.066421

(Hartree/Particle)
HF=-1030.525199
Zero-point correction= 0.218601
Thermal correction to Energy= 0.231196
Thermal correction to Enthalpy= 0.232140
Thermal correction to Gibbs Free Energy= 0.178886
Sum of electronic and zero-point Energies= -1030.306598
Sum of electronic and thermal Energies= -1030.294003
Sum of electronic and thermal Enthalpies= -1030.293059
Sum of electronic and thermal Free Energies= -1030.346313

[^{i}Pr$_2$NPPMe$_3$]

P	-2.108630	-0.014499	-0.018152
N	0.872538	-0.045169	0.175646
C	-2.110804	-0.836248	-1.649307
C	-3.619494	-0.584285	0.826943
C	1.569133	-1.202163	-0.420742
C	1.533460	1.255445	0.392743
C	-2.440768	1.739858	-0.419893
H	-2.573274	2.290528	0.503696
H	-1.592468	2.157033	-0.948309
C	2.452308	-1.999308	0.553018
C	1.695032	2.107493	-0.876918
C	2.851684	1.210595	1.176209
H	0.837499	1.796795	1.019388
H	2.743438	-2.942594	0.095389
H	1.908812	-2.227587	1.463251
H	3.359894	-1.470155	0.819256
H	2.509518	1.780990	-1.511258
H	1.899628	3.138000	-0.594637
H	0.785700	2.097509	-1.469516
H	3.659371	0.763442	0.606104
H	2.734460	0.654461	2.100197
H	3.157064	2.222966	1.430306
P	-0.436465	-0.455718	1.269951
H	-1.216855	-0.549577	-2.190506
H	-2.984006	-0.555872	-2.230552
H	-2.098268	-1.909586	-1.505053
H	-4.501138	-0.334738	0.246278
H	-3.689588	-0.111029	1.799431
H	-3.572943	-1.657047	0.967418
C	2.342542	-0.896144	-1.706690
H	1.728696	-0.361984	-2.424264
H	2.650850	-1.833796	-2.161768
H	3.241844	-0.317135	-1.528056
H	0.770533	-1.875815	-0.708543
H	-3.328662	1.843176	-1.036923

(Hartree/Particle)
HF=-1090.2103171
Zero-point correction= 0.329277
Thermal correction to Energy= 0.346578
Thermal correction to Enthalpy= 0.347522
Thermal correction to Gibbs Free Energy= 0.285110
Sum of electronic and zero-point Energies= -1089.881040
Sum of electronic and thermal Energies= -1089.863740
Sum of electronic and thermal Enthalpies= -1089.862795
Sum of electronic and thermal Free Energies= -1089.925207

[RPPMe$_2$]$^+$

[MePPMe$_2$]$^+$

P	-0.640386	0.029930	-0.010305
P	1.120055	-0.877130	0.006779
C	-2.130018	-1.006373	0.002120
C	2.372786	0.480669	-0.002339
H	-2.717451	-0.775331	0.882917
H	-1.841557	-2.049263	0.019498
H	-2.715310	-0.805355	-0.887317
C	-1.010061	1.809203	0.007061
H	-1.469896	2.066419	0.954306
H	-1.702536	2.020987	-0.799662
H	-0.109956	2.389793	-0.133118
H	2.843091	0.486193	-0.980172
H	3.126903	0.208084	0.727459
H	1.995418	1.465479	0.227935

(Hartree/Particle)
HF=-800.1075783
Zero-point correction= 0.120922
Thermal correction to Energy= 0.129577
Thermal correction to Enthalpy= 0.130521
Thermal correction to Gibbs Free Energy= 0.086757
Sum of electronic and zero-point Energies= -799.986656
Sum of electronic and thermal Energies= -799.978002
Sum of electronic and thermal Enthalpies= -799.977057
Sum of electronic and thermal Free Energies= -800.020821

[EtPPMe$_2$]$^+$

P	1.090319	0.038401	-0.006313
P	-0.626096	-0.924678	-0.227011
C	1.409152	1.828160	0.012796
C	-1.927174	0.387058	-0.430780
H	1.763336	2.113192	0.996761
H	0.512692	2.380853	-0.226707
H	2.174211	2.047327	-0.723277
C	2.596175	-0.946398	0.234411
H	3.049568	-0.679605	1.181802
H	3.292656	-0.742681	-0.570306
H	2.340202	-1.997798	0.236474
H	-2.259341	0.291665	-1.460714
H	-1.541944	1.388192	-0.300518
C	-3.096368	0.117139	0.529381
H	-3.508711	-0.875728	0.387019
H	-3.883832	0.836466	0.338758

H	-2.792903	0.216525	1.565729

(Hartree/Particle)
HF=-839.1529842
Zero-point correction= 0.151698
Thermal correction to Energy= 0.161448
Thermal correction to Enthalpy= 0.162392
Thermal correction to Gibbs Free Energy= 0.115740
Sum of electronic and zero-point Energies= -839.001286
Sum of electronic and thermal Energies= -838.991536
Sum of electronic and thermal Enthalpies= -838.990592
Sum of electronic and thermal Free Energies= -839.037244

[^{i}PrPPMe$_2$]$^+$

P	-1.379670	0.043342	0.000000
P	0.323584	-0.966486	0.000000
C	-1.651680	1.841530	0.000000
C	-2.933696	-0.896299	0.000000
C	1.692117	0.311207	0.000000
H	-0.711052	2.372091	0.000002
H	-2.218539	2.108027	0.884681
H	-2.218535	2.108028	-0.884683
H	-2.711562	-1.955325	-0.000001
H	-3.506077	-0.644043	-0.884860
H	-3.506077	-0.644044	0.884860
C	2.525856	0.105983	1.275190
H	1.282622	1.312116	0.000000
C	2.525855	0.105983	-1.275190
H	2.940982	-0.896029	-1.322878
H	3.353430	0.807193	-1.270663
H	1.945494	0.278786	-2.175154
H	1.945494	0.278786	2.175154
H	3.353431	0.807193	1.270663
H	2.940982	-0.896029	1.322877

(Hartree/Particle)
HF=-878.1984713
Zero-point correction= 0.181419
Thermal correction to Energy= 0.192536
Thermal correction to Enthalpy= 0.193480
Thermal correction to Gibbs Free Energy= 0.143495
Sum of electronic and zero-point Energies= -878.017052
Sum of electronic and thermal Energies= -878.005936
Sum of electronic and thermal Enthalpies= -878.004992
Sum of electronic and thermal Free Energies= -878.054976

[^{t}BuPPMe$_2$]$^+$

P	-1.541790	-0.022145	0.000000
P	0.125978	-1.092587	0.000000
C	-1.836548	1.773266	0.000000
C	1.640538	0.048850	0.000000
C	-3.101703	-0.954456	0.000000
H	-0.906749	2.319545	-0.000005
H	-2.409446	2.029237	0.884284
H	-2.409454	2.029236	-0.884279
H	-2.886395	-2.014635	0.000001
H	-3.672762	-0.698209	-0.884660
H	-3.672762	-0.698208	0.884660
C	1.690132	0.909040	-1.272860
C	2.826753	-0.938317	0.000001
C	1.690130	0.909042	1.272858
H	0.901785	1.651748	1.306865
H	2.638424	1.436854	1.298480
H	1.626930	0.305854	2.172554
H	1.626933	0.305851	-2.172555
H	2.638425	1.436853	-1.298481
H	0.901786	1.651746	-1.306869
H	2.826249	-1.573658	-0.879975
H	2.826248	-1.573657	0.879979
H	3.752144	-0.372132	0.000002

(Hartree/Particle)
HF=-917.238226
Zero-point correction= 0.211076
Thermal correction to Energy= 0.223247
Thermal correction to Enthalpy= 0.224192
Thermal correction to Gibbs Free Energy= 0.172742
Sum of electronic and zero-point Energies= -917.027150
Sum of electronic and thermal Energies= -917.014979
Sum of electronic and thermal Enthalpies= -917.014034
Sum of electronic and thermal Free Energies= -917.065484

[PhPPMe$_2$]$^+$

P	-2.049970	0.113156	0.138398
P	-0.702172	-1.253311	-0.391110
C	-3.801709	-0.299897	-0.108768
C	-1.778508	1.821490	0.697785
H	-3.883909	-1.331368	-0.426046
H	-4.225865	0.349433	-0.865488
H	-4.335566	-0.164847	0.824345
H	-0.760920	1.933819	1.044041
H	-2.464430	2.017581	1.514068
H	-1.975195	2.514844	-0.111369
H	2.779386	2.231824	-1.013655
C	2.520763	1.282211	-0.582702
C	1.238552	0.790482	-0.723371
H	0.517242	1.357549	-1.283604
C	0.904647	-0.451842	-0.177851
C	1.881672	-1.204530	0.476240
H	1.649200	-2.179185	0.868402
C	3.158833	-0.696591	0.629922
H	3.903764	-1.272707	1.146604
C	3.476592	0.544328	0.102651
H	4.473377	0.931493	0.209946

(Hartree/Particle)
HF=-990.6619548
Zero-point correction= 0.178700
Thermal correction to Energy= 0.189682
Thermal correction to Enthalpy= 0.190626
Thermal correction to Gibbs Free Energy= 0.140847
Sum of electronic and zero-point Energies= -990.483255
Sum of electronic and thermal Energies= -990.472273
Sum of electronic and thermal Enthalpies= -990.471329
Sum of electronic and thermal Free Energies= -990.521108

[^{i}Pr$_2$NPPMe$_2$]$^+$

P	2.336472	-0.021511	0.534119
N	-0.746940	0.247460	-0.066162
C	2.664900	-1.403150	-0.660058
C	3.602242	1.230938	0.027859
C	-0.806358	-1.166938	0.451637
C	-1.997650	1.041558	-0.435237
C	-0.969893	-2.184690	-0.680992
C	-2.690805	1.632634	0.792641
C	-2.948482	0.289012	-1.358836
H	-1.606392	1.875761	-1.003094
H	-0.737100	-3.168883	-0.288092
H	-0.295066	-1.982745	-1.504874
H	-1.978621	-2.217486	-1.065745
H	-3.231490	0.897868	1.370597
H	-3.405793	2.371217	0.446276
H	-1.982910	2.136789	1.441518
H	-3.468932	-0.517937	-0.860513
H	-2.437984	-0.100148	-2.231463
H	-3.696978	0.995081	-1.700831

P	0.596718	1.081350	-0.272612
H	1.990412	-2.230704	-0.492196
H	3.670770	-1.747323	-0.444553
H	2.623570	-1.098864	-1.698749
H	4.565245	0.865024	0.366570
H	3.418558	2.173734	0.530117
H	3.651034	1.394219	-1.042358
C	-1.823477	-1.372417	1.570496
H	-1.681724	-0.663397	2.376541
H	-1.665202	-2.366918	1.973037
H	-2.846914	-1.321928	1.224487
H	0.163392	-1.324851	0.898794

(Hartree/Particle)
HF=-1050.3855611
Zero-point correction= 0.291310
Thermal correction to Energy= 0.306583
Thermal correction to Enthalpy= 0.307527
Thermal correction to Gibbs Free Energy= 0.249712
Sum of electronic and zero-point Energies= -1050.094251
Sum of electronic and thermal Energies= -1050.078978
Sum of electronic and thermal Enthalpies= -1050.078034
Sum of electronic and thermal Free Energies= -1050.135849

$[R_2P]^+$

$[Me_2P]^+$

P	0.000000	-0.645514	0.000000
C	-1.405259	0.464197	0.015355
C	1.405259	0.464197	-0.015355
H	-2.288358	-0.021284	0.411165
H	-1.592081	0.660829	-1.050447
H	-1.213314	1.416631	0.496190
H	2.288357	-0.021283	-0.411166
H	1.592082	0.660827	1.050447
H	1.213314	1.416632	-0.496188

(Hartree/Particle)
HF=-419.6914925
Zero-point correction= 0.076595
Thermal correction to Energy= 0.081691
Thermal correction to Enthalpy= 0.082635
Thermal correction to Gibbs Free Energy= 0.049306
Sum of electronic and zero-point Energies= -419.614897
Sum of electronic and thermal Energies= -419.609802
Sum of electronic and thermal Enthalpies= -419.608857
Sum of electronic and thermal Free Energies= -419.642186

$[Et_2P]^+$

P	0.030561	-0.722219	-0.387274
C	1.555252	-0.367493	0.494155
C	-1.228532	0.140626	0.560618
H	2.234683	-1.205438	0.394110
H	1.392332	-0.140996	1.540540
C	2.159735	0.874265	-0.247241
C	-2.531896	0.459983	-0.182508
H	-0.811977	0.995278	1.085200
H	-1.409273	-0.618146	1.343405
H	2.350284	0.675091	-1.295977
H	3.108660	1.093409	0.225003
H	1.531199	1.753004	-0.157849
H	-2.375682	1.228461	-0.930429
H	-3.265121	0.826224	0.524353
H	-2.940878	-0.417890	-0.669387

(Hartree/Particle)
HF=-497.7844298
Zero-point correction= 0.138171
Thermal correction to Energy= 0.145787
Thermal correction to Enthalpy= 0.146731
Thermal correction to Gibbs Free Energy= 0.106239
Sum of electronic and zero-point Energies= -497.646259
Sum of electronic and thermal Energies= -497.638643
Sum of electronic and thermal Enthalpies= -497.637699
Sum of electronic and thermal Free Energies= -497.678191

$[^iPr_2P]^+$

P	0.000000	0.000004	-0.732935
C	1.428844	0.097586	0.363126
C	-1.428843	-0.097588	0.363126
C	1.812196	-1.422501	0.429470
H	1.134325	0.418516	1.355214
C	2.581064	0.950159	-0.181191
C	-2.581062	-0.950162	-0.181193
C	-1.812198	1.422498	0.429472
H	-1.134324	-0.418519	1.355213
H	2.872892	0.638702	-1.178909
H	3.441672	0.843072	0.468966
H	2.317999	2.001478	-0.213311
H	-2.872889	-0.638705	-1.178911
H	-3.441671	-0.843075	0.468963
H	-2.317996	-2.001480	-0.213313
H	1.028349	-2.047231	0.846698
H	2.670462	-1.497104	1.087493
H	2.108266	-1.817375	-0.537569
H	-2.108277	1.817372	-0.537564
H	-1.028349	2.047231	0.846694
H	-2.670458	1.497099	1.087503

(Hartree/Particle)
HF=-575.8769361
Zero-point correction= 0.198097
Thermal correction to Energy= 0.208535
Thermal correction to Enthalpy= 0.209479
Thermal correction to Gibbs Free Energy= 0.162451
Sum of electronic and zero-point Energies= -575.678839
Sum of electronic and thermal Energies= -575.668401
Sum of electronic and thermal Enthalpies= -575.667457
Sum of electronic and thermal Free Energies= -575.714485

$[^tBu_2P]^+$

P	-0.069235	-0.851211	-0.389506
C	-1.575514	0.075661	-0.003518
C	1.517044	0.055574	-0.031279
C	2.611044	-1.032009	-0.107268
C	1.656878	0.822535	1.289818
C	1.642659	0.999847	-1.254428
C	-2.778150	-0.531809	-0.741550
C	-1.667389	1.599583	0.031241
C	-1.363640	-0.589254	1.393034
H	1.467857	0.489213	-2.201004
H	2.659955	1.374587	-1.291836
H	0.972158	1.847419	-1.190332
H	2.531737	-1.740751	0.711310
H	3.584081	-0.557578	-0.041489
H	2.576034	-1.586712	-1.039477
H	1.580760	0.162184	2.147151
H	0.941684	1.624533	1.402066
H	2.645738	1.268374	1.316569
H	-0.499836	-1.339475	1.406158
H	-2.187220	-1.227687	1.678953
H	-1.089335	0.125508	2.152334
H	-2.826792	-0.160339	-1.758716

H	-3.693040	-0.244928	-0.233270
H	-2.737500	-1.615196	-0.777841
H	-1.692878	1.995980	-0.976934
H	-0.856869	2.070006	0.565663
H	-2.595600	1.882269	0.516977

Hartree/Particle)
HF=-653.9588226
Zero-point correction= 0.257324 (
Thermal correction to Energy= 0.269536
Thermal correction to Enthalpy= 0.270480
Thermal correction to Gibbs Free Energy= 0.220955
Sum of electronic and zero-point Energies= -653.701499
Sum of electronic and thermal Energies= -653.689287
Sum of electronic and thermal Enthalpies= -653.688343
Sum of electronic and thermal Free Energies= -653.737867

$[Ph_2P]^+$

P	0.000000	1.383065	0.000000
H	4.717047	1.002192	0.661809
C	3.837209	0.470622	0.352385
C	2.611203	1.099847	0.344911
H	2.543420	2.132507	0.640027
C	1.443144	0.399638	-0.030905
C	1.566296	-0.936370	-0.479585
H	0.709872	-1.466884	-0.846391
C	2.793842	-1.545107	-0.500061
H	2.890735	-2.552884	-0.858099
C	3.924378	-0.847259	-0.068741
H	4.881939	-1.336149	-0.083845
H	-0.709872	-1.466885	0.846391
C	-1.566296	-0.936370	0.479585
C	-1.443144	0.399638	0.030904
C	-2.611203	1.099847	-0.344911
H	-2.543421	2.132507	-0.640026
C	-3.837209	0.470622	-0.352384
H	-4.717047	1.002192	-0.661808
C	-3.924378	-0.847259	0.068742
H	-4.881939	-1.336149	0.083845
C	-2.793841	-1.545107	0.500061
H	-2.890735	-2.552884	0.858099

(Hartree/Particle)
HF=-800.8352352
Zero-point correction= 0.194008
Thermal correction to Energy= 0.204119
Thermal correction to Enthalpy= 0.205063
Thermal correction to Gibbs Free Energy= 0.156756
Sum of electronic and zero-point Energies= -800.641228
Sum of electronic and thermal Energies= -800.631116
Sum of electronic and thermal Enthalpies= -800.630172
Sum of electronic and thermal Free Energies= -800.678480

$[(^iPr_2N)_2P]^+$

N	1.203794	0.047548	-0.227418
C	1.290305	0.414385	1.233637
C	2.424011	-0.187406	-1.100064
C	1.643830	1.891186	1.436590
C	3.013362	-1.585842	-0.912246
C	3.474803	0.913365	-1.004456
H	2.030542	-0.152788	-2.110108
H	1.415656	2.147632	2.465925
H	1.055829	2.536264	0.795414
H	2.690258	2.102559	1.272442
H	3.527861	-1.703979	0.030391
H	3.733605	-1.757406	-1.705004
H	2.248675	-2.351243	-0.989656
H	4.012016	0.896951	-0.065390
H	3.044444	1.896299	-1.151625
H	4.198154	0.745808	-1.794744
H	-1.327641	2.769773	0.245589
H	-3.150134	1.435554	1.543164
C	-1.743149	2.399109	-0.684919
H	-3.646731	-1.372459	-1.058316
H	-2.455124	3.127411	-1.054526
H	-0.946230	2.353041	-1.426785
C	-3.561808	1.235519	0.558874
N	-1.457557	0.008130	-0.038255
H	-0.497911	-1.783792	1.878064
C	-2.869175	-2.002161	-0.644560
C	-2.450106	1.059179	-0.473113
H	-4.167879	2.086093	0.268110
H	-3.349575	-2.878843	-0.223851
H	-4.222786	0.381485	0.622469
C	-1.024632	-2.237353	1.049645
H	-2.235048	-2.336516	-1.462500
C	-2.059236	-1.292322	0.443637
H	-0.299602	-2.596871	0.323158
H	-1.541795	-3.110239	1.430413
H	-2.721623	-1.005823	1.244922
P	-0.152332	-0.087231	-1.059816
H	-2.897948	0.758881	-1.417497
C	2.198946	-0.504627	2.045246
H	1.961196	-1.550441	1.898861
H	2.049998	-0.272560	3.094409
H	3.247698	-0.349696	1.827186
H	0.282524	0.285441	1.586807

(Hartree/Particle)
HF=-920.2736006
Zero-point correction= 0.417696
Thermal correction to Energy= 0.436823
Thermal correction to Enthalpy= 0.437767
Thermal correction to Gibbs Free Energy= 0.371437
Sum of electronic and zero-point Energies= -919.855904
Sum of electronic and thermal Energies= -919.836778
Sum of electronic and thermal Enthalpies= -919.835834
Sum of electronic and thermal Free Energies= -919.902163

$[R_2P]^{\cdot}$

$[Me_2P]^{\cdot}$

P	0.000000	-0.699465	0.000000
C	-1.410947	0.501383	0.000000
C	1.410947	0.501383	0.000000
H	-2.351371	-0.038201	0.000007
H	-1.380402	1.137941	-0.880452
H	-1.380395	1.137951	0.880445
H	2.351371	-0.038201	0.000006
H	1.380395	1.137951	0.880445
H	1.380402	1.137941	-0.880452

(Hartree/Particle)
HF=-419.9748043
Zero-point correction= 0.077945
Thermal correction to Energy= 0.082891
Thermal correction to Enthalpy= 0.083835
Thermal correction to Gibbs Free Energy= 0.050116
Sum of electronic and zero-point Energies= -419.896859
Sum of electronic and thermal Energies= -419.891913
Sum of electronic and thermal Enthalpies= -419.890969
Sum of electronic and thermal Free Energies= -419.924688

$[Et_2P]^{\cdot}$

P	-0.056120	-0.882026	-0.216827
C	1.584920	-0.327389	0.466024
C	-1.140049	0.540210	0.298169
H	2.232898	-1.199046	0.456255
H	1.454726	-0.047451	1.509236
C	2.258948	0.815246	-0.304492
C	-2.603808	0.336265	-0.100124
H	-0.761464	1.457113	-0.146663
H	-1.059033	0.661011	1.376720
H	2.411994	0.549217	-1.346125
H	3.230052	1.042698	0.126292
H	1.666863	1.724230	-0.276276
H	-2.708610	0.237243	-1.176550
H	-3.210762	1.179329	0.217048
H	-3.014932	-0.559946	0.355010

(Hartree/Particle)
HF=-498.0582066
Zero-point correction= 0.139042
Thermal correction to Energy= 0.146443
Thermal correction to Enthalpy= 0.147387
Thermal correction to Gibbs Free Energy= 0.106291
Sum of electronic and zero-point Energies= -497.919164
Sum of electronic and thermal Energies= -497.911764
Sum of electronic and thermal Enthalpies= -497.910819
Sum of electronic and thermal Free Energies= -497.951916

[$^{i}Pr_2P]^{\cdot}$

P	0.000000	0.000006	-0.893678
C	1.446918	0.128674	0.290455
C	-1.446918	-0.128676	0.290453
C	1.853853	-1.245699	0.841838
H	1.136216	0.752638	1.125114
C	2.631961	0.809719	-0.404407
C	-2.631958	-0.809721	-0.404413
C	-1.853857	1.245692	0.841845
H	-1.136213	-0.752644	1.125108
H	2.958880	0.239883	-1.270675
H	3.476694	0.892696	0.275130
H	2.375790	1.808555	-0.743847
H	-2.958878	-0.239881	-1.270678
H	-3.476691	-0.892705	0.275122
H	-2.375783	-1.808554	-0.743859
H	1.037434	-1.735997	1.362597
H	2.677283	-1.141917	1.544728
H	2.181631	-1.904578	0.042230
H	-2.181638	1.904575	0.042240
H	-1.037440	1.735991	1.362607
H	-2.677287	1.141904	1.544735

(Hartree/Particle)
HF=-576.1425454
Zero-point correction= 0.198767
Thermal correction to Energy= 0.208627
Thermal correction to Enthalpy= 0.209571
Thermal correction to Gibbs Free Energy= 0.163012
Sum of electronic and zero-point Energies= -575.943778
Sum of electronic and thermal Energies= -575.933918
Sum of electronic and thermal Enthalpies= -575.932974
Sum of electronic and thermal Free Energies= -575.979534

[$^{t}Bu_2P]^{\cdot}$

P	0.000000	-1.040329	0.000000
C	-1.569998	0.026366	0.001028
C	1.569998	0.026366	-0.001028
C	2.688912	-0.960014	-0.392275
C	1.876653	0.564762	1.408248
C	1.567306	1.181320	-1.012888
C	-2.688911	-0.960012	0.392282
C	-1.876655	0.564755	-1.408250
C	-1.567305	1.181324	1.012883
H	1.341869	0.835205	-2.017204
H	2.552436	1.643667	-1.039564
H	0.854911	1.955135	-0.753628
H	2.735161	-1.803439	0.290369
H	3.652856	-0.456362	-0.366667
H	2.542660	-1.353003	-1.394039
H	1.900486	-0.235942	2.141735
H	1.150509	1.297920	1.736994
H	2.853851	1.044689	1.412908
H	-1.341864	0.835215	2.017200
H	-2.552435	1.643671	1.039559
H	-0.854911	1.955139	0.753617
H	-2.735160	-1.803441	-0.290357
H	-3.652856	-0.456361	0.366671
H	-2.542658	-1.352995	1.394048
H	-1.900490	-0.235952	-2.141733
H	-1.150512	1.297912	-1.737000
H	-2.853853	1.044682	-1.412910

(Hartree/Particle)
HF=-654.2178846
Zero-point correction= 0.258174
Thermal correction to Energy= 0.270225
Thermal correction to Enthalpy= 0.271169
Thermal correction to Gibbs Free Energy= 0.221126
Sum of electronic and zero-point Energies= -653.959711
Sum of electronic and thermal Energies= -653.947660
Sum of electronic and thermal Enthalpies= -653.946715
Sum of electronic and thermal Free Energies= -653.996759

[$Ph_2P]^{\cdot}$

P	-0.000002	1.700155	-0.000001
H	4.536663	0.243169	1.256968
C	3.680389	0.012965	0.648248
C	2.553244	0.824075	0.706221
H	2.551868	1.680220	1.358399
c	1.429761	0.539879	-0.065025
c	1.462826	-0.570216	-0.910830
H	0.605649	-0.810671	-1.514153
C	2.588432	-1.374759	-0.976533
H	2.599014	-2.226179	-1.633753
C	3.699493	-1.085938	-0.194094
H	4.571426	-1.713689	-0.243438
H	-0.605641	-0.810681	1.514140
C	-1.462822	-0.570223	0.910823
C	-1.429762	0.539878	0.065025
C	-2.553249	0.824079	-0.706212
H	-2.551877	1.680228	-1.358384
C	-3.680393	0.012967	-0.648240
H	-4.536669	0.243174	-1.256955
C	-3.699491	-1.085941	0.194094
H	-4.571423	-1.713695	0.243438
C	-2.588426	-1.374767	0.976526
H	-2.599004	-2.226191	1.633740

Convergence failure.

Item	Value	Threshold	Converged?
Maximum Force	0.008699	0.000002	NO
RMS Force	0.001461	0.000001	NO
Maximum Displacement	0.717748	0.000006	NO
RMS Displacement	0.274511	0.000004	NO

Predicted change in Energy=-1.456984D-03

(Hartree/Particle)

HF = -801.069509859

[(^{i}Pr$_2$N)$_2$P]$^{\cdot}$

N	1.261150	-0.002458	-0.242931
C	1.301409	0.789642	0.994161
C	2.455449	-0.533612	-0.925921
C	1.739459	2.250950	0.801002
C	2.964014	-1.855272	-0.334754
C	3.599640	0.465045	-1.115450
H	2.115378	-0.775988	-1.929525
H	1.464230	2.826407	1.682135
H	1.248796	2.696649	-0.055670
H	2.809560	2.354276	0.669630
H	3.428360	-1.725784	0.635271
H	3.705804	-2.291212	-0.999818
H	2.149866	-2.564132	-0.231254
H	4.097507	0.709704	-0.183926
H	3.248628	1.383394	-1.571923
H	4.344174	0.025620	-1.773523
H	-1.385752	2.730285	0.322033
H	-3.328288	1.401813	1.409450
C	-1.722263	2.388421	-0.652186
H	-3.581826	-1.530753	-1.051406
H	-2.414630	3.124184	-1.050936
H	-0.868860	2.352382	-1.317064
C	-3.637229	1.208765	0.385570
N	-1.479508	-0.015879	-0.069124
H	-0.622941	-1.445016	2.087572
C	-2.847051	-2.100205	-0.491094
C	-2.423882	1.033665	-0.534056
H	-4.210165	2.064766	0.043433
H	-3.371376	-2.903765	0.019966
H	-4.305189	0.356515	0.381778
C	-1.102267	-2.050847	1.327732
H	-2.159606	-2.548308	-1.201976
C	-2.103571	-1.222103	0.524640
H	-0.333274	-2.490131	0.702440
H	-1.626949	-2.862377	1.824534
H	-2.827055	-0.867401	1.244873
P	-0.155969	-0.304450	-1.148166
H	-2.805538	0.775903	-1.524544
C	2.099298	0.142890	2.130970
H	1.786553	-0.879957	2.303975
H	1.932343	0.706285	3.045471
H	3.167612	0.146627	1.942781
H	0.272633	0.821098	1.319408

(Hartree/Particle)
HF=-920.4660313
Zero-point correction= 0.415789
Thermal correction to Energy= 0.434960
Thermal correction to Enthalpy= 0.435904
Thermal correction to Gibbs Free Energy= 0.368983
Sum of electronic and zero-point Energies= -920.050242
Sum of electronic and thermal Energies= -920.031071
Sum of electronic and thermal Enthalpies= -920.030127
Sum of electronic and thermal Free Energies= -920.097048

[R]$^+$

[Me]$^+$

C	0.000000	0.000000	0.000000
H	-0.936863	0.541093	0.000000
H	0.937032	0.540799	0.000000
H	-0.000170	-1.081892	0.000000

(Hartree/Particle)
HF=-39.2438233
Zero-point correction= 0.033071
Thermal correction to Energy= 0.035917
Thermal correction to Enthalpy= 0.036861
Thermal correction to Gibbs Free Energy= 0.014013
Sum of electronic and zero-point Energies= -39.210753
Sum of electronic and thermal Energies= -39.207906
Sum of electronic and thermal Enthalpies= -39.206962
Sum of electronic and thermal Free Energies= -39.229810

[Et]$^+$

C	-0.742583	0.000000	-0.041254
C	0.675834	0.000000	-0.031069
H	-1.304062	-0.922276	0.011162
H	-1.304062	0.922276	0.011162
H	1.155805	-0.915472	-0.343952
H	0.697003	0.000000	1.099522
H	1.155805	0.915473	-0.343952

(Hartree/Particle)
HF=-78.3345915
Zero-point correction= 0.063401
Thermal correction to Energy= 0.066923
Thermal correction to Enthalpy= 0.067867
Thermal correction to Gibbs Free Energy= 0.040616
Sum of electronic and zero-point Energies= -78.271191
Sum of electronic and thermal Energies= -78.267669
Sum of electronic and thermal Enthalpies= -78.266724
Sum of electronic and thermal Free Energies= -78.293976

[^{i}Pr]$^+$

C	0.000000	0.455886	-0.026969
C	1.297265	-0.198642	-0.008326
C	-1.297265	-0.198642	-0.008326
H	0.000000	1.537791	0.005317
H	1.258414	-1.264463	-0.172443
H	1.698653	0.015401	0.992112
H	1.978971	0.304359	-0.691464
H	-1.258414	-1.264464	-0.172438
H	-1.978968	0.304356	-0.691469
H	-1.698657	0.015405	0.992110

(Hartree/Particle)
HF=-117.4134451
Zero-point correction= 0.093380
Thermal correction to Energy= 0.097569
Thermal correction to Enthalpy= 0.098513
Thermal correction to Gibbs Free Energy= 0.067731
Sum of electronic and zero-point Energies= -117.320065
Sum of electronic and thermal Energies= -117.315876
Sum of electronic and thermal Enthalpies= -117.314932
Sum of electronic and thermal Free Energies= -117.345714

[^{t}Bu]$^+$

C	0.000000	0.000000	-0.063097
C	1.178641	0.882301	-0.010587
C	0.174774	-1.461883	-0.010587
C	-1.353415	0.579583	-0.010587
H	-1.397334	1.572803	-0.435346
H	-1.569721	0.672203	1.060422
H	-2.102560	-0.074011	-0.435360
H	2.060754	0.423726	-0.435347
H	1.367006	1.023316	1.060422
H	0.987184	1.857877	-0.435359
H	1.115376	-1.783865	-0.435360
H	-0.663420	-1.996528	-0.435346

H	0.202715	-1.695520	1.060422

(Hartree/Particle)
HF=-156.4829429
Zero-point correction= 0.123362
Thermal correction to Energy= 0.128906
Thermal correction to Enthalpy= 0.129850
Thermal correction to Gibbs Free Energy= 0.094910
Sum of electronic and zero-point Energies= -156.359581
Sum of electronic and thermal Energies= -156.354037
Sum of electronic and thermal Enthalpies= -156.353093
Sum of electronic and thermal Free Energies= -156.388033

[Ph]$^+$

H	-2.160214	1.404015	0.000002
C	-1.269762	0.807371	0.000001
C	0.000000	1.178875	0.000000
C	1.269762	0.807371	-0.000001
H	2.160214	1.404015	0.000000
C	1.212277	-0.604809	0.000001
H	2.148734	-1.132596	0.000002
C	0.000000	-1.281947	-0.000001
H	0.000000	-2.355161	-0.000001
C	-1.212277	-0.604808	-0.000002
H	-2.148734	-1.132596	-0.000003

(Hartree/Particle)
HF=-229.805894
Zero-point correction= 0.090747
Thermal correction to Energy= 0.095165
Thermal correction to Enthalpy= 0.096109
Thermal correction to Gibbs Free Energy= 0.063381
Sum of electronic and zero-point Energies= -229.715147
Sum of electronic and thermal Energies= -229.710729
Sum of electronic and thermal Enthalpies= -229.709785
Sum of electronic and thermal Free Energies= -229.742513

[^{i}Pr$_2$N]$^+$

N	0.000000	0.886810	-0.000003
C	1.156618	0.304777	0.496450
C	-1.156619	0.304775	-0.496452
C	2.018110	0.587624	-0.834972
C	-2.018108	0.587629	0.834971
C	-1.247967	-1.142067	-0.962450
H	-1.567308	0.994395	-1.223671
H	3.004692	0.240322	-0.554765
H	2.055680	1.638452	-1.076541
H	1.660329	0.009080	-1.674509
H	-1.660326	0.009090	1.674510
H	-3.004690	0.240324	0.554767
H	-2.055678	1.638458	1.076533
H	-1.132855	-1.855529	-0.159438
H	-0.522497	-1.353964	-1.739021
H	-2.232672	-1.278639	-1.393636
C	1.247965	-1.142062	0.962455
H	0.522495	-1.353955	1.739027
H	2.232670	-1.278633	1.393643
H	1.132854	-1.855528	0.159447
H	1.567306	0.994400	1.223667

(Hartree/Particle)
HF=-289.5394315
Zero-point correction= 0.202620
Thermal correction to Energy= 0.211228
Thermal correction to Enthalpy= 0.212172
Thermal correction to Gibbs Free Energy= 0.170427
Sum of electronic and zero-point Energies= -289.336812
Sum of electronic and thermal Energies= -289.328203
Sum of electronic and thermal Enthalpies= -289.327259
Sum of electronic and thermal Free Energies= -289.369005

[R]$^·$

[Me]$^·$

C	0.000000	0.000000	0.000000
H	0.000000	1.073482	0.000000
H	0.929663	-0.536741	0.000000
H	-0.929663	-0.536741	0.000000

(Hartree/Particle)
HF=-39.5736882
Zero-point correction= 0.030704
Thermal correction to Energy= 0.033848
Thermal correction to Enthalpy= 0.034793
Thermal correction to Gibbs Free Energy= 0.011909
Sum of electronic and zero-point Energies= -39.542984
Sum of electronic and thermal Energies= -39.539840
Sum of electronic and thermal Enthalpies= -39.538896
Sum of electronic and thermal Free Energies= -39.561779

[Et]$^·$

C	-0.798049	0.000000	-0.033844
C	0.699122	0.000000	0.001963
H	-1.346613	-0.919568	0.071358
H	-1.346613	0.919568	0.071358
H	1.105931	-0.878980	-0.488989
H	1.074925	-0.000001	1.026554
H	1.105931	0.878980	-0.488988

(Hartree/Particle)
HF=-78.6205236
Zero-point correction= 0.062427
Thermal correction to Energy= 0.066281
Thermal correction to Enthalpy= 0.067225
Thermal correction to Gibbs Free Energy= 0.038487
Sum of electronic and zero-point Energies= -78.558097
Sum of electronic and thermal Energies= -78.554242
Sum of electronic and thermal Enthalpies= -78.553298
Sum of electronic and thermal Free Energies= -78.582037

[^{i}Pr]$^·$

C	0.000000	0.540001	-0.076729
C	1.300222	-0.201547	0.007335
C	-1.300222	-0.201547	0.007335
H	0.000000	1.589910	0.168643
H	1.304604	-1.064049	-0.655247
H	1.483699	-0.574839	1.017898
H	2.138773	0.433212	-0.260796
H	-1.304604	-1.064049	-0.655247
H	-2.138773	0.433212	-0.260795
H	-1.483698	-0.574840	1.017898

(Hartree/Particle)
HF=-117.6684659
Zero-point correction= 0.093190
Thermal correction to Energy= 0.098092
Thermal correction to Enthalpy= 0.099036
Thermal correction to Gibbs Free Energy= 0.066386
Sum of electronic and zero-point Energies= -117.575276
Sum of electronic and thermal Energies= -117.570374
Sum of electronic and thermal Enthalpies= -117.569430

Sum of electronic and thermal Free Energies= -117.602080

[^{t}Bu]$^·$

C	-0.065436	-0.032745	-0.187934
C	0.761155	-1.276389	-0.018453
C	-1.397082	0.024233	0.506401
C	0.657859	1.263131	-0.424958
H	1.459398	1.144726	-1.148555
H	1.109438	1.640174	0.497632
H	-0.015480	2.033534	-0.789885
H	0.155621	-2.172928	-0.116550
H	1.229444	-1.310130	0.969891
H	1.559909	-1.326301	-0.753014
H	-1.944407	-0.907385	0.394148
H	-2.014997	0.828051	0.116354
H	-1.277902	0.200876	1.579644

(Hartree/Particle)
HF=-156.7165434
Zero-point correction= 0.123451
Thermal correction to Energy= 0.129528
Thermal correction to Enthalpy= 0.130472
Thermal correction to Gibbs Free Energy= 0.094406
Sum of electronic and zero-point Energies= -156.593093
Sum of electronic and thermal Energies= -156.587015
Sum of electronic and thermal Enthalpies= -156.586071
Sum of electronic and thermal Free Energies= -156.622138

[Ph]$^·$

H	-2.152670	1.316590	0.000000
C	-1.228214	0.768176	0.000000
C	0.000000	1.410091	0.000000
C	1.228214	0.768176	0.000000
H	2.152670	1.316590	0.000001
C	1.216726	-0.633355	0.000000
H	2.144346	-1.177883	0.000001
C	0.000000	-1.325782	0.000000
H	0.000000	-2.401131	0.000000
C	-1.216726	-0.633355	-0.000001
H	-2.144346	-1.177883	-0.000001

(Hartree/Particle)
HF=-230.1156077
Zero-point correction= 0.090708
Thermal correction to Energy= 0.094983
Thermal correction to Enthalpy= 0.095927
Thermal correction to Gibbs Free Energy= 0.062697
Sum of electronic and zero-point Energies= -230.024900
Sum of electronic and thermal Energies= -230.020625
Sum of electronic and thermal Enthalpies= -230.019681
Sum of electronic and thermal Free Energies= -230.052910

[^{i}Pr$_2$N]$^·$

N	-0.000001	0.000005	-1.102448
C	1.239502	0.350169	-0.417595
C	-1.239502	-0.350169	-0.417595
C	2.035920	-0.910048	-0.048339
C	-2.035922	0.910045	-0.048336
C	-1.141136	-1.327152	0.759625
H	-1.819241	-0.856210	-1.184616
H	3.056640	-0.631446	0.196927
H	2.068499	-1.599861	-0.885379
H	1.618378	-1.427782	0.807051
H	-1.618379	1.427780	0.807052
H	-3.056641	0.631440	0.196933
H	-2.068507	1.599859	-0.885376
H	-0.638277	-0.904819	1.621919
H	-0.625170	-2.237089	0.472338
H	-2.145411	-1.604388	1.068161
C	1.141138	1.327150	0.759627
H	0.625174	2.237089	0.472341
H	2.145414	1.604385	1.068163
H	0.638279	0.904817	1.621920
H	1.819244	0.856211	-1.184615

(Hartree/Particle)
HF=-289.8340374
Zero-point correction= 0.203599
Thermal correction to Energy= 0.212523
Thermal correction to Enthalpy= 0.213467
Thermal correction to Gibbs Free Energy= 0.170107
Sum of electronic and zero-point Energies= -289.630439
Sum of electronic and thermal Energies= -289.621515
Sum of electronic and thermal Enthalpies= -289.620571
Sum of electronic and thermal Free Energies= -289.663930

Alkanes

Ethane, [CH_3CH_3]

H	-1.102047	0.026465	1.071200
C	-0.085612	0.203050	0.731003
H	0.131776	1.257774	0.873681
H	0.581321	-0.361750	1.376183
C	0.085612	-0.203050	-0.731003
H	-0.131776	-1.257774	-0.873681
H	-0.581321	0.361750	-1.376183
H	1.102047	-0.026465	-1.071200

(Hartree/Particle)
HF=-79.2519347
Zero-point correction= 0.078578
Thermal correction to Energy= 0.081983
Thermal correction to Enthalpy= 0.082927
Thermal correction to Gibbs Free Energy= 0.055514
Sum of electronic and zero-point Energies= -79.173357
Sum of electronic and thermal Energies= -79.169952
Sum of electronic and thermal Enthalpies= -79.169008
Sum of electronic and thermal Free Energies= -79.196420

Propane, [$CH_3CH_2CH_3$]

H	0.000000	2.161548	0.366302
C	0.000000	1.273535	-0.259445
H	-0.877722	1.318653	-0.899655
H	0.877722	1.318653	-0.899655
C	0.000000	0.000000	0.584285
H	0.870613	0.000000	1.236825
H	-0.870613	0.000000	1.236825
C	0.000000	-1.273535	-0.259445
H	-0.877722	-1.318653	-0.899655
H	0.877722	-1.318653	-0.899655
H	0.000000	-2.161548	0.366302

(Hartree/Particle)
HF=-118.2962386
Zero-point correction= 0.108990
Thermal correction to Energy= 0.113368
Thermal correction to Enthalpy= 0.114313
Thermal correction to Gibbs Free Energy= 0.084092

Sum of electronic and zero-point Energies= -118.187249
Sum of electronic and thermal Energies= -118.182870
Sum of electronic and thermal Enthalpies= -118.181926
Sum of electronic and thermal Free Energies= -118.212147

Isobutane, $[(CH_3)_2CHCH_3]$

H	-1.023179	-1.887804	0.420219
C	-0.283381	-1.415682	-0.220782
H	0.695476	-1.792102	0.069857
H	-0.472697	-1.738927	-1.240956
C	-0.339602	0.109171	-0.102754
C	0.668981	0.772489	-1.043518
H	1.686867	0.485742	-0.786595
H	0.494097	0.482403	-2.076160
H	0.606246	1.856002	-0.987423
C	-0.122196	0.558517	1.343999
H	-0.859552	0.116312	2.008712
H	-0.196921	1.638788	1.436273
H	0.863266	0.263001	1.698764
H	-1.336413	0.429614	-0.404359

(Hartree/Particle)
HF=-157.3410613
Zero-point correction= 0.138717
Thermal correction to Energy= 0.144189
Thermal correction to Enthalpy= 0.145134
Thermal correction to Gibbs Free Energy= 0.111256
Sum of electronic and zero-point Energies= -157.202345
Sum of electronic and thermal Energies= -157.196872
Sum of electronic and thermal Enthalpies= -157.195928
Sum of electronic and thermal Free Energies= -157.229805

Neopentane, $[(CH_3)_4C]$

H	-2.086293	0.303747	-0.545013
C	-1.252486	-0.336848	-0.821425
H	-1.559349	-1.367746	-0.663041
H	-1.070733	-0.204440	-1.885114
C	0.000000	0.000000	0.000000
C	1.151334	-0.924689	-0.419889
H	0.896518	-1.968315	-0.252810
H	1.385134	-0.805010	-1.474884
H	2.053824	-0.708696	0.146556
C	-0.298109	-0.199386	1.492750
H	-1.111253	0.444185	1.819268
H	0.572997	0.032312	2.100607
H	-0.584308	-1.227307	1.701241
C	0.399261	1.460924	-0.251436
H	1.285467	1.728570	0.318656
H	-0.398783	2.140444	0.037317
H	0.616778	1.632256	-1.302784

(Hartree/Particle)
HF=-196.3853836
Zero-point correction= 0.168084
Thermal correction to Energy= 0.174673
Thermal correction to Enthalpy= 0.175617
Thermal correction to Gibbs Free Energy= 0.139207
Sum of electronic and zero-point Energies= -196.217300
Sum of electronic and thermal Energies= -196.210711
Sum of electronic and thermal Enthalpies= -196.209767
Sum of electronic and thermal Free Energies= -196.246177

Toluene, $[C_6H_5CH_3]$

H	-0.732197	-2.124492	0.000000
C	-0.195849	-1.190519	0.000000
C	-0.905453	0.007863	0.000000
C	-0.188259	1.195636	0.000000
H	-0.714216	2.134694	0.000000
C	1.200113	1.191824	0.000000
H	1.735244	2.125159	0.000000
C	1.893157	-0.004568	0.000000
H	2.968546	-0.010038	0.000000
C	1.187083	-1.199217	0.000000
H	1.715270	-2.136519	0.000000
C	-2.416344	0.002109	0.000000
H	-2.812446	1.011066	-0.000003
H	-2.803442	-0.509318	0.876593
H	-2.803442	-0.509323	-0.876590

(Hartree/Particle)
HF=-269.8028429
Zero-point correction= 0.135386
Thermal correction to Energy= 0.141313
Thermal correction to Enthalpy= 0.142257
Thermal correction to Gibbs Free Energy= 0.104112
Sum of electronic and zero-point Energies= -269.667457
Sum of electronic and thermal Energies= -269.661530
Sum of electronic and thermal Enthalpies= -269.660586
Sum of electronic and thermal Free Energies= -269.698731

Diisopropylmethylamine, $[^{2}Pr_2NMe]$

N	-0.019250	0.520232	-0.626354
C	-1.228160	-0.262732	-0.349781
C	1.268323	0.281234	0.038622
C	-1.977217	0.066104	0.955585
C	2.059222	-0.882919	-0.570089
C	1.246022	0.185452	1.571589
H	1.848981	1.167910	-0.194644
H	-2.956617	-0.405600	0.935994
H	-2.134855	1.133386	1.071453
H	-1.458171	-0.294643	1.835780
H	1.668348	-1.853373	-0.290892
H	3.090297	-0.838511	-0.227123
H	2.057394	-0.812569	-1.652367
H	0.791050	-0.736933	1.917277
H	0.709747	1.017199	2.016998
H	2.264163	0.206767	1.951617
C	-1.026172	-1.772228	-0.476352
H	-0.529759	-2.022978	-1.406556
H	-1.996197	-2.261259	-0.467863
H	-0.451943	-2.185432	0.345934
H	-1.903408	0.011053	-1.156949
C	-0.298604	1.932392	-0.791254
H	0.501577	2.404679	-1.351976
H	-0.412999	2.481656	0.147543
H	-1.213334	2.063212	-1.359664

(Hartree/Particle)
HF=-329.4812395
Zero-point correction= 0.247692
Thermal correction to Energy= 0.257705
Thermal correction to Enthalpy= 0.258649
Thermal correction to Gibbs Free Energy= 0.213746
Sum of electronic and zero-point Energies= -329.233547
Sum of electronic and thermal Energies= -329.223535
Sum of electronic and thermal Enthalpies= -329.222591
Sum of electronic and thermal Free Energies= -329.267494

Olefins

Ethene, $[CH_2CH_2]$

H	1.224350	-0.916402	-0.000002
C	0.659206	0.000000	0.000001
H	1.224350	0.916402	-0.000002
C	-0.659206	0.000000	0.000001
H	-1.224350	-0.916402	-0.000002
H	-1.224350	0.916402	-0.000002

(Hartree/Particle)
HF=-78.0561082
Zero-point correction= 0.053978
Thermal correction to Energy= 0.056960
Thermal correction to Enthalpy= 0.057905
Thermal correction to Gibbs Free Energy= 0.031836
Sum of electronic and zero-point Energies= -78.002130
Sum of electronic and thermal Energies= -77.999148
Sum of electronic and thermal Enthalpies= -77.998204
Sum of electronic and thermal Free Energies= -78.024272

Propene, [CH_2CHCH_3]

H	2.224886	0.282835	-0.000002
C	1.274125	-0.221092	0.000000
H	1.295034	-1.298646	-0.000001
C	0.138563	0.451300	0.000000
H	0.174629	1.530654	-0.000001
C	-1.232902	-0.160787	0.000000
H	-1.183378	-1.244258	0.000001
H	-1.794943	0.156448	0.874878
H	-1.794942	0.156446	-0.874879

(Hartree/Particle)
HF=-117.1048536
Zero-point correction= 0.084292
Thermal correction to Energy= 0.088244
Thermal correction to Enthalpy= 0.089188
Thermal correction to Gibbs Free Energy= 0.059364
Sum of electronic and zero-point Energies= -117.020562
Sum of electronic and thermal Energies= -117.016609
Sum of electronic and thermal Enthalpies= -117.015665
Sum of electronic and thermal Free Energies= -117.045490

Isobutene, [CH_2CHCH_3]

H	-0.916782	2.014172	0.000000
C	0.000000	1.449672	0.000000
H	0.916782	2.014172	0.000000
C	0.000000	0.127255	0.000000
C	1.274852	-0.676935	0.000000
H	2.150982	-0.039318	-0.000001
H	1.320008	-1.322012	0.874782
H	1.320007	-1.322014	-0.874781
C	-1.274852	-0.676935	0.000000
H	-2.150982	-0.039318	0.000001
H	-1.320007	-1.322013	0.874781
H	-1.320008	-1.322012	-0.874782

(Hartree/Particle)
HF=-156.1532293
Zero-point correction= 0.114097
Thermal correction to Energy= 0.119187
Thermal correction to Enthalpy= 0.120131
Thermal correction to Gibbs Free Energy= 0.086941
Sum of electronic and zero-point Energies= -156.039132
Sum of electronic and thermal Energies= -156.034043
Sum of electronic and thermal Enthalpies= -156.033098
Sum of electronic and thermal Free Energies= -156.066288

Isopropyliminopropane, [$^{i}PrNC(CH_3)$]

N	0.166898	-0.842915	0.272753
C	-1.259640	-0.538038	0.183201
C	1.112149	-0.062834	0.018243
C	-1.741983	-0.297550	-1.251324
C	2.519777	-0.593185	0.181335
C	1.069923	1.383268	-0.440252
H	-2.828368	-0.281106	-1.274846
H	-1.405117	-1.101552	-1.897914
H	-1.388709	0.638732	-1.668504
H	3.079117	0.018895	0.885626
H	3.051504	-0.557241	-0.767075
H	2.492156	-1.613330	0.536535
H	0.079302	1.761032	-0.625187
H	1.654165	1.486606	-1.350664
H	1.542668	2.009191	0.312554
C	-1.724566	0.539606	1.168792
H	-1.366565	0.311569	2.167823
H	-2.810666	0.562168	1.199632
H	-1.381808	1.534366	0.908026
H	-1.739930	-1.456526	0.504750

(Hartree/Particle)
HF=-289.2817756
Zero-point correction= 0.191769
Thermal correction to Energy= 0.200802
Thermal correction to Enthalpy= 0.201746
Thermal correction to Gibbs Free Energy= 0.157392
Sum of electronic and zero-point Energies= -289.090007
Sum of electronic and thermal Energies= -289.080974
Sum of electronic and thermal Enthalpies= -289.080030
Sum of electronic and thermal Free Energies= -289.124384

[PMe_3] Derivatives

[PMe_3]

P	0.000000	0.000000	-0.584764
C	1.630358	0.169690	0.271147
C	-0.668223	-1.496776	0.271147
H	2.103103	1.099794	-0.028460
H	2.284509	-0.643125	-0.028461
H	1.534604	0.159724	1.353857
H	-1.699217	-1.656880	-0.028462
H	-0.628978	-1.408867	1.353856
H	-0.099102	-2.371238	-0.028460
C	-0.962135	1.327086	0.271147
H	-0.585293	2.300005	-0.028460
H	-0.905628	1.249143	1.353856
H	-2.004002	1.271443	-0.028461

(Hartree/Particle)
HF=-459.6246024
Zero-point correction= 0.118999
Thermal correction to Energy= 0.125391
Thermal correction to Enthalpy= 0.126335
Thermal correction to Gibbs Free Energy= 0.089967
Sum of electronic and zero-point Energies= -459.505604
Sum of electronic and thermal Energies= -459.499211
Sum of electronic and thermal Enthalpies= -459.498267
Sum of electronic and thermal Free Energies= -459.534636

[PMe_3]$^{+\cdot}$

P	0.000000	0.000000	-0.334591
C	0.265485	-1.720509	0.152478
C	-1.622748	0.630337	0.152478
H	1.213819	-2.067394	-0.239310
H	-0.534172	-2.337121	-0.239315

H	0.276320	-1.790748	1.236714
H	-1.756921	1.631167	-0.239315
H	-1.688993	0.656074	1.236715
H	-2.397325	-0.017502	-0.239309
C	1.357262	1.090172	0.152478
H	2.291092	0.705954	-0.239315
H	1.412673	1.134675	1.236714
H	1.183507	2.084895	-0.239310

(Hartree/Particle)
HF=-459.3779685
Zero-point correction= 0.119088
Thermal correction to Energy= 0.125848
Thermal correction to Enthalpy= 0.126792
Thermal correction to Gibbs Free Energy= 0.088695
Sum of electronic and zero-point Energies= -459.258880
Sum of electronic and thermal Energies= -459.252120
Sum of electronic and thermal Enthalpies= -459.251176
Sum of electronic and thermal Free Energies= -459.289273

$[HPMe_3]^+$

P	0.000000	0.000000	0.335953
C	0.367440	1.679635	-0.213327
C	-1.638327	-0.521605	-0.213327
H	-1.850116	-1.516293	0.160927
H	-1.672803	-0.532572	-1.296855
H	-2.386212	0.167538	0.160941
C	1.270886	-1.158030	-0.213327
H	2.238205	-0.844103	0.160932
H	1.297627	-1.182400	-1.296855
H	1.048012	-2.150290	0.160936
H	-0.388089	2.360394	0.160929
H	0.375179	1.714976	-1.296855
H	1.338199	1.982751	0.160939
H	0.000000	-0.000001	1.725553

(Hartree/Particle)
HF=-460.0058663
Zero-point correction= 0.130691
Thermal correction to Energy= 0.137299
Thermal correction to Enthalpy= 0.138243
Thermal correction to Gibbs Free Energy= 0.101325
Sum of electronic and zero-point Energies= -459.875175
Sum of electronic and thermal Energies= -459.868567
Sum of electronic and thermal Enthalpies= -459.867623
Sum of electronic and thermal Free Energies= -459.904542

Miscellaneous fragments

$[H_2PPMe_3]^+$

P	-0.338963	0.000000	-0.003431
P	1.866076	0.000000	0.092634
C	-0.934345	1.475831	0.862792
C	-0.934343	-1.475846	0.862767
H	-0.589326	2.370120	0.356872
H	-0.565338	1.478983	1.881607
H	-2.018592	1.474113	0.876130
H	-0.589322	-2.370127	0.356832
H	-2.018589	-1.474130	0.876106
H	-0.565334	-1.479015	1.881582
C	-0.996844	0.000014	-1.692837
H	-0.657261	0.882736	-2.221320
H	-2.081117	0.000011	-1.658584
H	-0.657258	-0.882698	-2.221336
H	2.014318	1.048315	-0.831127
H	2.014318	-1.048303	-0.831139

(Hartree/Particle)
HF=-801.3361396
Zero-point correction= 0.140733
Thermal correction to Energy= 0.149377
Thermal correction to Enthalpy= 0.150321
Thermal correction to Gibbs Free Energy= 0.108322
Sum of electronic and zero-point Energies= -801.195406
Sum of electronic and thermal Energies= -801.186763
Sum of electronic and thermal Enthalpies= -801.185818
Sum of electronic and thermal Free Energies= -801.227817

$[HPPMe_3]^{+\cdot}$

P	0.304695	0.000743	0.000000
P	-1.903692	0.101576	0.000001
C	0.910913	-0.860800	1.476759
C	0.940086	1.696634	0.000006
H	0.551836	-1.882962	1.483597
H	0.563849	-0.354632	2.369728
H	1.995611	-0.864487	1.468969
H	0.593221	2.221319	-0.882598
H	2.024225	1.678978	0.000007
H	0.593220	2.221314	0.882614
C	0.910912	-0.860788	-1.476766
H	0.551836	-1.882951	-1.483611
H	1.995610	-0.864475	-1.468977
H	0.563847	-0.354613	-2.369731
H	-2.019774	-1.302557	-0.000005

(Hartree/Particle)
HF=-800.7342979
Zero-point correction= 0.130228
Thermal correction to Energy= 0.138747
Thermal correction to Enthalpy= 0.139691
Thermal correction to Gibbs Free Energy= 0.097299
Sum of electronic and zero-point Energies= -800.604070
Sum of electronic and thermal Energies= -800.595551
Sum of electronic and thermal Enthalpies= -800.594606
Sum of electronic and thermal Free Energies= -800.636999

$[P^tBu_3]$

P	0.000000	0.000000	-0.682506
C	-0.301120	1.782141	-0.004140
C	1.693940	-0.630293	-0.004140
C	2.128781	-1.818452	-0.892828
C	1.758388	-1.060945	1.471585
C	2.775739	0.445112	-0.229900
C	-1.773348	2.181305	-0.229900
C	0.510436	2.752803	-0.892828
C	0.039612	2.053280	1.471585
H	2.780219	0.803973	-1.253477
H	3.749216	0.000920	-0.037095
H	2.677509	1.292509	0.433841
H	1.501866	-2.690007	-0.785888
H	3.139542	-2.113764	-0.618852
H	2.135936	-1.538046	-1.940853
H	1.132032	-1.917530	1.682029
H	1.473525	-0.264647	2.147535
H	2.780218	-1.346230	1.716987
H	-0.507573	1.408432	2.147534
H	-0.224238	3.080853	1.716988
H	1.094614	1.939131	1.682029
H	-2.086372	2.005754	-1.253476
H	-1.875405	3.246457	-0.037095
H	-2.458100	1.672537	0.433842
H	0.264020	2.618797	-1.940853
H	1.578682	2.645656	-0.785887
H	0.260804	3.775804	-0.618852

C	-1.392820	-1.151848	-0.004140
C	-1.002392	-2.626416	-0.229900
H	-0.219409	-2.965045	0.433841
H	-0.693849	-2.809727	-1.253476
H	-1.873812	-3.247377	-0.037095
C	-1.797999	-0.992335	1.471585
H	-2.555976	-1.734625	1.716989
H	-2.226648	-0.021603	1.682027
H	-0.965952	-1.143782	2.147534
C	-2.639217	-0.934351	-0.892828
H	-3.400344	-1.662040	-0.618852
H	-2.399956	-1.080750	-1.940853
H	-3.080548	0.044350	-0.785886

(Hartree/Particle)
HF=-810.9596631
Zero-point correction= 0.391061
Thermal correction to Energy= 0.407909
Thermal correction to Enthalpy= 0.408854
Thermal correction to Gibbs Free Energy= 0.350504
Sum of electronic and zero-point Energies= -810.568602
Sum of electronic and thermal Energies= -810.551754
Sum of electronic and thermal Enthalpies= -810.550809
Sum of electronic and thermal Free Energies= -810.609159

[P(H)^{t}Bu$_2$]

P	-0.000327	-0.985148	-0.164719
C	1.581141	0.042014	0.006136
C	-1.584489	0.041837	0.002817
C	-2.713828	-0.965995	-0.292615
C	-1.634033	1.154082	-1.055541
C	-1.829821	0.637443	1.398028
C	2.667551	-0.989247	0.371300
C	1.576615	1.144860	1.072582
C	1.936573	0.650136	-1.362007
H	-1.828306	-0.133749	2.162450
H	-2.804327	1.122779	1.423630
H	-1.090795	1.379078	1.670147
H	-2.634917	-1.370661	-1.296108
H	-3.680972	-0.476786	-0.198278
H	-2.698618	-1.800367	0.404184
H	-1.431740	0.770504	-2.051540
H	-0.922966	1.946102	-0.847103
H	-2.624842	1.603131	-1.070570
H	1.993151	-0.114317	-2.130528
H	2.905384	1.142459	-1.307311
H	1.212456	1.391257	-1.682074
H	2.502037	-1.413951	1.357144
H	3.643223	-0.508671	0.377167
H	2.704230	-1.805092	-0.344592
H	1.303242	0.759959	2.049934
H	0.896873	1.951218	0.822411
H	2.572310	1.577618	1.155087
H	-0.008766	-1.534072	1.132526

(Hartree/Particle)
HF=-654.8182256
Zero-point correction= 0.268514
Thermal correction to Energy= 0.280610
Thermal correction to Enthalpy= 0.281554
Thermal correction to Gibbs Free Energy= 0.232059
Sum of electronic and zero-point Energies= -654.549712
Sum of electronic and thermal Energies= -654.537616
Sum of electronic and thermal Enthalpies= -654.536672
Sum of electronic and thermal Free Energies= -654.586166

[^{t}Bu(H)P]$^+$

P	1.486262	0.236329	-0.000011
C	-0.309143	0.003436	-0.000001
C	-1.013098	1.371795	-0.000021
C	-0.661461	-0.812438	1.277240
C	-0.661483	-0.812489	-1.277201
H	-0.374369	-0.301927	-2.191827
H	-1.740632	-0.922429	-1.298318
H	-0.222212	-1.803406	-1.269375
H	-0.763636	1.955522	0.880070
H	-2.085866	1.216717	-0.000010
H	-0.763650	1.955488	-0.880139
H	-0.374331	-0.301841	2.191841
H	-0.222193	-1.803357	1.269444
H	-1.740610	-0.922375	1.298380
H	1.864679	-1.119150	0.000005

(Hartree/Particle)
HF=-497.7569106
Zero-point correction= 0.136243
Thermal correction to Energy= 0.142893
Thermal correction to Enthalpy= 0.143837
Thermal correction to Gibbs Free Energy= 0.106515
Sum of electronic and zero-point Energies= -497.620667
Sum of electronic and thermal Energies= -497.614018
Sum of electronic and thermal Enthalpies= -497.613073
Sum of electronic and thermal Free Energies= -497.650396

[^{t}Bu(H)P]$^{\cdot}$

P	1.564147	0.132080	0.000000
C	-0.314137	-0.002251	0.000000
C	-0.885972	1.424350	0.000001
C	-0.789263	-0.748987	1.256742
C	-0.789263	-0.748985	-1.256743
H	-0.465103	-0.250123	-2.165575
H	-1.876628	-0.794841	-1.274705
H	-0.413880	-1.767614	-1.280956
H	-0.568900	1.980423	0.877705
H	-1.973222	1.394062	0.000001
H	-0.568900	1.980424	-0.877702
H	-0.465103	-0.250128	2.165575
H	-0.413881	-1.767616	1.280953
H	-1.876628	-0.794844	1.274704
H	1.831838	-1.255696	0.000000

(Hartree/Particle)
HF=-498.0532546
Zero-point correction= 0.136779
Thermal correction to Energy= 0.143767
Thermal correction to Enthalpy= 0.144712
Thermal correction to Gibbs Free Energy= 0.106197
Sum of electronic and zero-point Energies= -497.916475
Sum of electronic and thermal Energies= -497.909487
Sum of electronic and thermal Enthalpies= -497.908543
Sum of electronic and thermal Free Energies= -497.947057

Dihydrogen, [H_2]

H	0.000000	0.000000	0.367698
H	0.000000	0.000000	-0.367698

(Hartree/Particle)
HF=-1.1325074
Zero-point correction= 0.010467
Thermal correction to Energy= 0.012828
Thermal correction to Enthalpy= 0.013772
Thermal correction to Gibbs Free Energy= -0.001001
Sum of electronic and zero-point Energies= -1.122040
Sum of electronic and thermal Energies= -1.119680
Sum of electronic and thermal Enthalpies= -1.118735

Sum of electronic and thermal Free Energies= -1.133508

$[(C_6H_4)_2P]^+$

P	0.000000	1.955990	-0.000001
H	-4.565183	0.051920	0.000001
C	-3.503288	-0.104062	0.000000
C	-2.634797	0.980648	0.000001
H	-3.020588	1.984661	0.000001
C	-1.260944	0.746225	0.000000
C	-0.743875	-0.575489	0.000000
C	-1.600905	-1.635414	0.000000
H	-1.248072	-2.649634	0.000000
C	-2.987742	-1.384469	0.000000
H	-3.662635	-2.221506	0.000000
C	0.743875	-0.575489	0.000000
C	1.260944	0.746225	0.000000
C	2.634797	0.980648	0.000001
H	3.020588	1.984661	0.000001
C	3.503288	-0.104062	0.000000
H	4.565183	0.051920	0.000001
C	2.987742	-1.384469	0.000000
H	3.662635	-2.221506	0.000000
C	1.600905	-1.635414	0.000000
H	1.248072	-2.649634	0.000000

(Hartree/Particle)
HF=-799.6780232
Zero-point correction= 0.170601
Thermal correction to Energy= 0.179474
Thermal correction to Enthalpy= 0.180418
Thermal correction to Gibbs Free Energy= 0.135966
Sum of electronic and zero-point Energies= -799.507422
Sum of electronic and thermal Energies= -799.498550
Sum of electronic and thermal Enthalpies= -799.497605
Sum of electronic and thermal Free Energies= -799.542057

PBE1PBE Optimizations, Frequency Analysis and MP2 Single Point Calculations

Cartesian coordinates, enthalpies and Gibbs energies for post-HF optimized structures at the PBE1PBE/6-311++G(d,p) level in the gas phase (298 K) absence of a counterion or solvent, and single point energies subsequently calculated at the MP2/6-311++G(d,p) level, in the order: $[R_2PPMe_3]^+$, $[R(H)PPMe_3]^{+\cdot}$, $[R_2PPMe_2]^{+\cdot}$, $[R_2PPMe_2]$, $[RPPMe_3]^{+\cdot}$, $[RPPMe_3]$, $[RPPMe_2]^+$, $[R_2P]^+$, $[R_2P]^{\cdot}$, $[R]^+$, $[R]^{\cdot}$, alkanes, olefins, $[PMe_3]$ derivatives, miscellaneous fragments. Within each subsection the structures are in the order: R = Me, Et, ^{i}Pr, ^{t}Bu, Ph, N^{i}Pr$_2$. The quoted HF energies come from the PBE1PBE /6-311++G(d,p) optimization as well as the later MP2 single point calculation. The structures of PMe_3 and $[PMe_3]^{+\bullet}$ failed to converge to the requested thresholds, but exhibited forces of the same order of magnitude as the desired thresholds and no negative frequencies were found for these geometries. Structures were accepted as true minima based on these observations with the consolation that any error introduced by this assumption is systematic across the study and does not alter the *trends* sought. The failure to converge in these cases is attributed to free rotation of methyl groups and was similarly observed for fragments related to the ^{t}Bu and ^{i}Pr substituted cations (*e.g.* $[^t\text{Bu}]^\bullet$, $[^t\text{Bu}]^+$, neopentane, isobutane).

$[R_2PPMe_3]^+$

$[Me_2PPMe_3]^+$

P	0.850968	0.000000	0.015055
P	-1.236335	0.000000	-0.707611
C	1.665684	1.464676	-0.661508
C	1.665684	-1.464676	-0.661509
C	-1.889177	1.430609	0.260180
C	-1.889177	-1.430609	0.260180
H	1.227787	2.371083	-0.238240
H	1.545664	1.482454	-1.746991
H	2.730561	1.438841	-0.416812
H	1.227787	-2.371083	-0.238240
H	2.730561	-1.438841	-0.416812
H	1.545664	-1.482454	-1.746991
C	1.071996	0.000000	1.812506
H	0.614057	0.890460	2.247883
H	2.139877	0.000000	2.047761
H	0.614057	-0.890460	2.247883
H	-1.453102	2.366288	-0.096374
H	-1.758116	1.341613	1.341010
H	-2.961565	1.481954	0.050267
H	-1.453102	-2.366288	-0.096374
H	-2.961565	-1.481954	0.050267
H	-1.758116	-1.341613	1.341010

(Hartree/Particle)
HF(PBE1PBE)=-881.6364829
Zero-point correction= 0.191376
Thermal correction to Energy= 0.203702
Thermal correction to Enthalpy= 0.204646
Thermal correction to Gibbs Free Energy= 0.154012
Sum of electronic and zero-point Energies= -881.445107
Sum of electronic and thermal Energies= -881.432781
Sum of electronic and thermal Enthalpies= -881.431836
Sum of electronic and thermal Free Energies= -881.482471
HF(MP2)= -879.4391675
MP2=-880.4535568

$[Et_2PPMe_3]^+$

P	-1.248106	-0.334135	0.015521
P	0.811335	0.073591	-0.687633
C	-1.609856	-2.081819	-0.282345
C	-2.409362	0.627137	-0.984017
C	1.694004	-1.286124	0.229827
C	1.240327	1.557876	0.347363
H	-1.017041	-2.713031	0.381921
H	-1.381198	-2.335436	-1.320093
H	-2.670896	-2.267491	-0.096411
H	-2.249715	1.696119	-0.834827
H	-3.434296	0.373138	-0.701564
H	-2.254716	0.392842	-2.039473
C	-1.565665	0.017710	1.764180
H	-0.902635	-0.580621	2.392634
H	-2.603053	-0.230280	2.004709
H	-1.395806	1.076275	1.970617
H	1.330693	-2.236682	-0.172571
H	1.468056	-1.263336	1.301491
C	3.202352	-1.185810	0.002014
C	0.519502	2.841340	-0.046396
H	2.315398	1.678775	0.172936

H	1.125706	1.338270	1.414201
H	3.448891	-1.183740	-1.063150
H	3.701259	-2.044688	0.456728
H	3.627952	-0.286959	0.453831
H	0.593939	3.034972	-1.119631
H	0.962249	3.693604	0.474200
H	-0.541031	2.824562	0.222384

(Hartree/Particle)
HF(PBE1PBE)=-960.1841254
Zero-point correction= 0.249192
Thermal correction to Energy= 0.264153
Thermal correction to Enthalpy= 0.265098
Thermal correction to Gibbs Free Energy= 0.208199
Sum of electronic and zero-point Energies= -959.934934
Sum of electronic and thermal Energies= -959.919972
Sum of electronic and thermal Enthalpies= -959.919028
Sum of electronic and thermal Free Energies= -959.975926
HF(MP2)=-957.5232492
MP2=-958.8465484

[^{i}Pr$_2$PPMe$_3$]$^+$

P	-1.564421	-0.088493	-0.037385
P	0.579383	0.056173	-0.618694
C	-2.279652	-1.681577	-0.526549
C	-2.451974	1.114985	-1.063492
C	1.484981	-1.334036	0.288274
C	1.130273	1.539292	0.407824
H	-2.004608	-2.474310	0.166569
H	-1.940340	-1.943720	-1.531132
H	-3.367750	-1.574461	-0.538312
H	-2.138404	2.136285	-0.853850
H	-3.523839	1.018777	-0.870433
H	-2.254301	0.894178	-2.114799
C	-1.994939	0.214615	1.698683
H	-1.528206	-0.538453	2.336427
H	-3.080422	0.166375	1.821741
H	-1.645161	1.202942	2.003117
C	0.675991	-2.563085	0.680676
H	1.877579	-0.873903	1.202236
C	2.653361	-1.742262	-0.614728
C	0.385139	2.825838	0.068054
C	2.631102	1.720284	0.163728
H	0.977290	1.293688	1.465901
H	2.291565	-2.250089	-1.513452
H	3.308199	-2.433610	-0.075681
H	3.260975	-0.893953	-0.937962
H	0.378107	3.019710	-1.009011
H	0.887495	3.671014	0.547243
H	-0.645500	2.831892	0.431608
H	-0.101510	-2.344533	1.418243
H	1.347364	-3.295233	1.139038
H	0.223553	-3.050114	-0.187661
H	2.842807	1.916014	-0.892112
H	3.222321	0.859341	0.481216
H	2.982674	2.582642	0.737438

(Hartree/Particle)
HF(PBE1PBE)=-1038.7261829
Zero-point correction= 0.306105
Thermal correction to Energy= 0.323513
Thermal correction to Enthalpy= 0.324457
Thermal correction to Gibbs Free Energy= 0.262910
Sum of electronic and zero-point Energies= -1038.420078
Sum of electronic and thermal Energies= -1038.402670
Sum of electronic and thermal Enthalpies= -1038.401725
Sum of electronic and thermal Free Energies= -1038.463273
HF(MP2)=-1035.5976196
MP2=-1037.237429

[^{t}Bu$_2$PPMe$_3$]$^+$

P	-1.737088	-0.001378	-0.014802
P	0.369315	0.002013	-0.759052
C	-2.521396	1.610252	-0.303283
C	1.121981	-1.578633	0.006257
C	1.129151	1.578722	0.007462
C	-2.114705	-0.439351	1.709402
H	-2.382239	1.913994	-1.342267
H	-2.124424	2.378216	0.359743
H	-3.591545	1.494298	-0.109277
H	-1.805032	-1.463238	1.921557
H	-3.194665	-0.356863	1.862809
H	-1.601254	0.238885	2.392092
C	-2.693092	-1.122557	-1.074926
H	-2.503994	-0.872152	-2.120851
H	-3.755523	-0.985003	-0.856338
H	-2.424035	-2.163456	-0.904598
C	0.701924	2.735847	-0.906545
C	2.652729	1.447769	-0.120247
C	0.760951	1.885434	1.458339
C	0.168407	-2.742965	-0.280577
C	1.440226	-1.538495	1.498836
C	2.402948	-1.837603	-0.804626
H	-0.303611	2.104626	1.581538
H	1.301596	2.781633	1.782616
H	1.029615	1.078166	2.141208
H	0.992307	2.555493	-1.944727
H	1.202938	3.651062	-0.573550
H	-0.370510	2.932439	-0.884803
H	2.961966	1.194440	-1.138452
H	3.077954	0.718784	0.570633
H	3.096800	2.419711	0.119403
H	2.195199	-1.905662	-1.876025
H	2.825762	-2.796129	-0.485122
H	3.168562	-1.077581	-0.651058
H	-0.715268	-2.733530	0.363875
H	0.692558	-3.681593	-0.073641
H	-0.146868	-2.773647	-1.327960
H	0.557280	-1.346894	2.113368
H	2.197091	-0.793175	1.746890
H	1.841184	-2.512860	1.800208

(Hartree/Particle)
HF(PBE1PBE)=-1117.2697485
Zero-point correction= 0.362239
Thermal correction to Energy= 0.381949
Thermal correction to Enthalpy= 0.382894
Thermal correction to Gibbs Free Energy= 0.318209
Sum of electronic and zero-point Energies= -1116.907510
Sum of electronic and thermal Energies= -1116.887799
Sum of electronic and thermal Enthalpies= -1116.886855
Sum of electronic and thermal Free Energies= -1116.951540
HF(MP2)=-1113.6720398
MP2=-1115.634892

[Ph$_2$PPMe$_3$]$^+$

P	0.147563	2.019606	-0.241311
P	0.004715	0.232693	1.072989
C	0.501911	1.738279	-1.995646
C	1.483761	3.053220	0.405513
H	-0.379257	1.340821	-2.500492
H	1.328927	1.030410	-2.089486
H	0.782493	2.686438	-2.462551

H	1.320115	3.248784	1.467151
H	1.509493	4.000436	-0.139320
H	2.439870	2.541498	0.276198
C	-1.402080	2.937678	-0.091992
H	-2.225834	2.328811	-0.470338
H	-1.338854	3.866174	-0.664972
H	-1.587115	3.173628	0.958308
H	4.794695	-0.754376	0.956338
C	3.829774	-1.040837	0.552042
C	2.686744	-0.356362	0.943498
H	2.767045	0.440708	1.678004
C	1.433702	-0.717695	0.430961
C	1.338987	-1.807899	-0.437273
H	0.374219	-2.141109	-0.800796
C	2.485187	-2.500313	-0.810628
H	2.401620	-3.353720	-1.475080
C	3.730141	-2.111813	-0.329402
H	4.620292	-2.657310	-0.623461
H	-2.313099	-0.535293	2.426304
C	-2.510815	-0.766709	1.383978
C	-1.531934	-0.510269	0.417175
C	-1.803721	-0.815096	-0.923545
H	-1.058455	-0.643446	-1.692430
C	-3.027464	-1.362611	-1.282918
H	-3.228249	-1.598063	-2.322736
C	-3.992323	-1.616470	-0.311698
H	-4.945756	-2.048255	-0.596578
C	-3.732899	-1.321200	1.020069
H	-4.480141	-1.522047	1.779965

(Hartree/Particle)
HF(PBE1PBE)=-1264.7453562
Zero-point correction= 0.299316
Thermal correction to Energy= 0.317739
Thermal correction to Enthalpy= 0.318684
Thermal correction to Gibbs Free Energy= 0.251607
Sum of electronic and zero-point Energies= -1264.446041
Sum of electronic and thermal Energies= -1264.427617
Sum of electronic and thermal Enthalpies= -1264.426673
Sum of electronic and thermal Free Energies= -1264.493750
HF(MP2)=-1260.5355001
MP2=-1262.896514

[(^{i}Pr$_2$N)$_2$PPMe$_3$]$^+$

P	-0.271477	-2.252956	-0.268426
N	1.290059	0.446499	-0.221504
C	0.117456	-3.184617	1.252530
C	-1.927384	-2.808382	-0.759129
C	1.924113	0.239232	1.098266
C	1.959757	1.225633	-1.309047
C	0.855456	-2.887066	-1.543522
H	0.600941	-2.435303	-2.504334
H	1.887954	-2.632733	-1.299770
C	2.520022	1.497583	1.725444
C	3.395295	0.800266	-1.589004
C	1.840101	2.740666	-1.150522
H	1.384424	0.966294	-2.206167
H	2.794700	1.284076	2.762545
H	1.822234	2.334891	1.723262
H	3.431867	1.809420	1.211753
H	4.084633	1.096046	-0.794977
H	3.733490	1.294411	-2.503861
H	3.477929	-0.278301	-1.742105
H	2.485524	3.136068	-0.365111
H	0.810307	3.032263	-0.928019
H	2.130372	3.225738	-2.087226
H	0.014144	1.573425	2.490973
H	-0.714066	-0.860343	2.465159
C	-1.001932	1.904305	2.275671
H	-2.785316	3.104982	0.448278
H	-1.461165	2.178814	3.229867
H	-0.946632	2.800548	1.656460
C	-1.740745	-0.488344	2.402460
N	-1.573325	0.626506	0.161725
H	-2.693577	-0.285725	-2.251663
C	-2.681750	2.748599	-0.578226
C	-1.826864	0.806492	1.611502
H	-2.073181	-0.312051	3.428928
H	-3.525014	3.148576	-1.147915
H	-2.382869	-1.264556	1.976939
C	-2.690319	0.799882	-2.131028
H	-1.761882	3.166288	-0.999293
C	-2.670996	1.226428	-0.671138
H	-1.849709	1.204343	-2.699688
H	-3.610158	1.185556	-2.578806
H	-3.597198	0.846037	-0.220334
P	-0.266324	-0.009810	-0.687604
H	-2.874002	1.123720	1.654216
H	1.052806	-2.841228	1.694533
H	0.214047	-4.242704	0.991595
H	-0.687524	-3.070041	1.978030
H	-1.977669	-3.899289	-0.714796
H	-2.136399	-2.483202	-1.779692
H	-2.680157	-2.385034	-0.092054
C	2.973382	-0.876843	1.108318
H	2.625616	-1.775137	0.594279
H	3.207685	-1.148497	2.142463
H	3.906433	-0.568933	0.635681
H	1.108027	-0.087736	1.750777
H	0.759707	-3.973233	-1.616479

(Hartree/Particle)
HF(PBE1PBE)=-1384.9704206
Zero-point correction= 0.509566
Thermal correction to Energy= 0.537355
Thermal correction to Enthalpy= 0.538299
Thermal correction to Gibbs Free Energy= 0.454644
Sum of electronic and zero-point Energies= -1384.460855
Sum of electronic and thermal Energies= -1384.433066
Sum of electronic and thermal Enthalpies= -1384.432121
Sum of electronic and thermal Free Energies= -1384.515777
HF(MP2)=-1379.9154645
MP2=-1382.86355

[R(H)PPMe$_3$]$^+$

[Et(H)PPMe$_3$]$^+$

P	1.083920	-0.046833	-0.012355
P	-0.890829	0.912778	0.138257
C	1.478038	-0.785734	1.588671
C	2.286300	1.256842	-0.356504
C	-2.023186	-0.565202	0.076996
H	0.775054	-1.589871	1.816981
H	1.415982	-0.024682	2.369548
H	2.490323	-1.196956	1.564166
H	2.083655	1.707714	-1.330470
H	3.294049	0.833476	-0.361880
H	2.224390	2.029008	0.413414
C	1.235276	-1.321381	-1.287491
H	0.548398	-2.144679	-1.082055
H	2.258198	-1.708134	-1.295970
H	1.003949	-0.896115	-2.266424
H	-1.912766	-1.095488	1.028153
H	-1.755327	-1.251100	-0.730074
C	-3.464060	-0.082138	-0.085627

H	-3.758712	0.592887	0.721782
H	-4.142473	-0.938090	-0.067935
H	-3.611866	0.436675	-1.036349
H	-0.883428	1.241857	-1.247691

(Hartree/Particle)
HF(PBE1PBE)=-881.6263057
Zero-point correction= 0.191245
Thermal correction to Energy= 0.203260
Thermal correction to Enthalpy= 0.204204
Thermal correction to Gibbs Free Energy= 0.153468
Sum of electronic and zero-point Energies= -881.435061
Sum of electronic and thermal Energies= -881.423046
Sum of electronic and thermal Enthalpies= -881.422102
Sum of electronic and thermal Free Energies= -881.472838
HF(MP2)=-879.4308833
MP2=-880.443826

[^{i}Pr(H)PPMe$_3$]$^+$

P	1.294327	0.012640	0.045666
P	-0.607461	-0.888022	-0.613014
C	1.749250	1.350372	-1.081274
C	2.558290	-1.273868	-0.084527
C	-1.894924	0.092111	0.335656
H	1.014594	2.156257	-1.034734
H	1.786349	0.965541	-2.102760
H	2.730978	1.743931	-0.805826
H	2.354385	-2.074435	0.629491
H	3.540076	-0.843419	0.129268
H	2.562410	-1.688780	-1.094972
C	1.300640	0.652159	1.738186
H	0.559310	1.446756	1.844312
H	2.289813	1.055944	1.971460
H	1.067069	-0.152255	2.438985
C	-2.062579	1.484901	-0.261367
H	-1.595589	0.167546	1.385658
C	-3.188098	-0.720534	0.248776
H	-3.514345	-0.845467	-0.788267
H	-3.982827	-0.192576	0.783490
H	-3.088588	-1.711313	0.699986
H	-1.175558	2.113260	-0.133128
H	-2.891423	1.999257	0.233030
H	-2.294826	1.438669	-1.329769
H	-0.440290	-1.959036	0.311302

(Hartree/Particle)
HF(PBE1PBE)=-920.9020943
Zero-point correction= 0.219432
Thermal correction to Energy= 0.232705
Thermal correction to Enthalpy= 0.233650
Thermal correction to Gibbs Free Energy= 0.180624
Sum of electronic and zero-point Energies= -920.682662
Sum of electronic and thermal Energies= -920.669389
Sum of electronic and thermal Enthalpies= -920.668445
Sum of electronic and thermal Free Energies= -920.721471
HF(MP2)=-918.4737493
MP2=-919.6437297

[^{t}Bu(H)PPMe$_3$]$^+$

P	-1.484499	0.016426	0.002847
P	0.400586	-1.133393	-0.090746
c	-1.617320	1.150389	-1.399256
c	1.843854	0.077687	0.011907
c	-1.847462	0.931708	1.523997
H	-1.399652	0.618981	-2.328195
H	-0.918198	1.980862	-1.286211
H	-2.635153	1.546784	-1.443500
H	-1.804430	0.251553	2.377425
H	-2.854106	1.353864	1.453859
H	-1.127373	1.736250	1.672744
C	-2.774577	-1.239242	-0.179738
H	-2.637421	-1.774386	-1.121462
H	-3.757434	-0.760788	-0.170996
H	-2.719768	-1.955233	0.643578
C	2.134549	0.572260	-1.407211
C	2.994768	-0.819641	0.490801
C	1.658433	1.248145	0.970891
H	0.901936	1.954938	0.615280
H	2.597797	1.806528	1.045374
H	1.392882	0.919246	1.979664
H	2.290992	-0.256463	-2.103097
H	3.048099	1.175148	-1.398344
H	1.333992	1.204099	-1.802872
H	3.133753	-1.694464	-0.152218
H	2.847918	-1.163427	1.518664
H	3.925246	-0.243450	0.462362
H	0.286148	-1.473371	1.288078

(Hartree/Particle)
HF(PBE1PBE)=-960.1757805
Zero-point correction= 0.247197
Thermal correction to Energy= 0.261713
Thermal correction to Enthalpy= 0.262657
Thermal correction to Gibbs Free Energy= 0.207420
Sum of electronic and zero-point Energies= -959.928584
Sum of electronic and thermal Energies= -959.914068
Sum of electronic and thermal Enthalpies= -959.913124
Sum of electronic and thermal Free Energies= -959.968360
HF(MP2)=-957.5135957
MP2=-958.8429884

[^{i}Pr$_2$N(H)PPMe$_3$]$^+$

P	-2.205599	-0.058023	-0.047381
N	1.015179	-0.166040	0.293800
C	-2.105853	-0.462148	-1.807474
C	-2.620666	1.700252	0.120788
C	1.906022	-1.149959	-0.395633
C	1.343961	1.268477	0.465989
C	-3.575403	-1.000982	0.671627
H	-4.515076	-0.729197	0.183997
H	-3.396443	-2.070216	0.540112
C	3.011933	-1.683481	0.510478
C	1.113758	2.104731	-0.793421
C	2.731384	1.515691	1.045068
H	0.627976	1.620862	1.217915
H	3.502082	-2.531210	0.023314
H	2.603893	-2.036213	1.461537
H	3.778131	-0.936044	0.719967
H	1.896926	1.956734	-1.537580
H	1.108018	3.166658	-0.531029
H	0.156749	1.869938	-1.268409
H	3.524509	1.263230	0.338173
H	2.888604	0.950859	1.966059
H	2.833392	2.579029	1.278283
P	-0.331415	-0.861826	0.987217
H	-1.949437	-1.536650	-1.926356
H	-1.268242	0.069420	-2.264028
H	-3.031307	-0.175599	-2.312985
H	-1.834663	2.320467	-0.312515
H	-2.725667	1.948859	1.179302
H	-3.565255	1.908761	-0.389703
C	2.446464	-0.664240	-1.732646
H	1.651068	-0.312459	-2.394000
H	2.951018	-1.496866	-2.229421

H	3.184175	0.133104	-1.617879
H	1.248224	-1.999692	-0.615935
H	-3.657607	-0.786906	1.740046
H	-0.571706	0.013116	2.088336

(Hartree/Particle)
HF(PBE1PBE)=-1094.0245687
Zero-point correction= 0.321355
Thermal correction to Energy= 0.339894
Thermal correction to Enthalpy= 0.340838
Thermal correction to Gibbs Free Energy= 0.275625
Sum of electronic and zero-point Energies= -1093.703213
Sum of electronic and thermal Energies= -1093.684675
Sum of electronic and thermal Enthalpies= -1093.683731
Sum of electronic and thermal Free Energies= -1093.748944
HF(MP2)=-1090.6361758
MP2=-1092.453736

$[R_2PPMe_2]^{+\cdot}$

$[Me_2PPMe_2]^{+\cdot}$

P	-0.977424	0.000000	0.459906
P	0.977424	0.000000	-0.459905
C	-1.866431	-1.463192	-0.169138
C	1.866431	-1.463192	0.169138
C	1.866431	1.463192	0.169138
H	-1.992469	-1.424944	-1.253728
H	-2.852671	-1.476823	0.303052
H	-1.344805	-2.378235	0.116142
C	-1.866431	1.463192	-0.169138
H	-1.344805	2.378235	0.116142
H	-2.852671	1.476823	0.303052
H	-1.992469	1.424944	-1.253728
H	1.344805	-2.378235	-0.116143
H	1.992469	-1.424945	1.253728
H	2.852671	-1.476823	-0.303052
H	1.344805	2.378235	-0.116142
H	2.852671	1.476823	-0.303052
H	1.992469	1.424945	1.253728

(Hartree/Particle)
HF(PBE1PBE)= -841.7066896
Zero-point correction= 0.152360
Thermal correction to Energy= 0.163047
Thermal correction to Enthalpy= 0.163991
Thermal correction to Gibbs Free Energy= 0.116053
Sum of electronic and zero-point Energies= -841.554330
Sum of electronic and thermal Energies= -841.543642
Sum of electronic and thermal Enthalpies= -841.542698
Sum of electronic and thermal Free Energies= -841.590637
HF(MP2)= -839.7607679
MP2= -840.5992863

$[Et_2PPMe_2]^{+\cdot}$

P	-1.649426	-0.025108	-0.440351
P	0.384152	-0.036670	0.277613
C	-2.433330	1.495940	0.198281
C	-2.514586	-1.420318	0.358227
C	1.263830	1.357153	-0.549262
C	1.142002	-1.617530	-0.283602
H	-1.896969	2.379862	-0.150859
H	-2.481967	1.496838	1.289483
H	-3.448751	1.537068	-0.205073
H	-2.058607	-2.367838	0.066864
H	-3.546310	-1.418179	-0.004041
H	-2.520620	-1.325402	1.446549
H	0.513609	2.100262	-0.835467
H	1.698105	0.965635	-1.475170
C	2.325939	1.993480	0.347281
H	1.886141	2.419394	1.251701
H	2.820406	2.800569	-0.197914
H	3.094256	1.278325	0.648216
C	2.610540	-1.727417	0.122450
H	3.225989	-0.965352	-0.360804
H	2.994713	-2.700912	-0.189604
H	2.743464	-1.649065	1.204100
H	1.030032	-1.684155	-1.371081
H	0.559239	-2.428230	0.163912

(Hartree/Particle)
HF(PBE1PBE)= -920.2554572
Zero-point correction= 0.210108
Thermal correction to Energy= 0.223512
Thermal correction to Enthalpy= 0.224456
Thermal correction to Gibbs Free Energy= 0.169419
Sum of electronic and zero-point Energies= -920.045349
Sum of electronic and thermal Energies= -920.031945
Sum of electronic and thermal Enthalpies= -920.031001
Sum of electronic and thermal Free Energies= -920.086039
HF(MP2)= -917.8468037
MP2= -918.9922795

$[^iPr_2PPMe_2]^{+\cdot}$

P	1.748049	-0.023045	0.466617
P	-0.265377	-0.012688	-0.332948
C	2.777078	-1.139221	-0.554330
C	-1.060770	1.557053	0.302448
C	-1.158686	-1.527805	0.277628
H	2.802661	-0.815557	-1.597025
H	2.416980	-2.165561	-0.492688
H	3.790302	-1.105186	-0.144369
C	2.455896	1.636513	0.173489
H	2.527683	1.874581	-0.890274
H	3.464178	1.627022	0.597944
H	1.881065	2.403806	0.693527
C	-0.877476	2.658766	-0.743836
H	-0.500091	1.815537	1.209869
C	-2.529906	1.392836	0.680823
C	-0.243121	-2.739086	0.412923
C	-2.333827	-1.821427	-0.658564
H	-1.536733	-1.250829	1.268982
H	-3.152833	1.184848	-0.192392
H	-2.879479	2.337918	1.105968
H	-2.700047	0.618378	1.430985
H	0.194103	-3.026784	-0.547601
H	-0.839006	-3.588142	0.759069
H	0.554712	-2.584345	1.144033
H	0.165585	2.810444	-1.029657
H	-1.251847	3.604177	-0.341522
H	-1.445197	2.435162	-1.651124
H	-1.982206	-2.109988	-1.652859
H	-3.016438	-0.977336	-0.767821
H	-2.908603	-2.657924	-0.251569

(Hartree/Particle)
HF(PBE1PBE)= -998.8033017
Zero-point correction= 0.266847
Thermal correction to Energy= 0.282696
Thermal correction to Enthalpy= 0.283640
Thermal correction to Gibbs Free Energy= 0.223877
Sum of electronic and zero-point Energies= -998.536455
Sum of electronic and thermal Energies= -998.520606
Sum of electronic and thermal Enthalpies= -998.519662
Sum of electronic and thermal Free Energies= -998.579424

HF(MP2)= -995.9275479
MP2= -997.3881365

$[^tBu_2PPMe_2]^{+\cdot}$

P	-1.685580	-1.245787	-0.036383
P	0.132535	-0.010549	-0.378396
C	-2.848509	-0.581948	-1.303863
C	1.607239	-1.110736	-0.029823
C	0.226176	1.830662	-0.005619
C	-2.380709	-0.569732	1.535491
H	-2.448959	-0.758081	-2.304530
H	-3.099517	0.471807	-1.182660
H	-3.764134	-1.174049	-1.209513
H	-1.695021	-0.759296	2.363696
H	-3.289342	-1.150729	1.726297
H	-2.650384	0.486503	1.507771
C	-1.135789	2.432235	-0.362229
C	1.300479	2.480768	-0.884542
C	0.533011	2.021883	1.484089
C	1.530047	-2.265094	-1.038908
C	1.458391	-1.647534	1.402309
C	2.934349	-0.371905	-0.190664
H	-0.229421	1.574447	2.126696
H	0.549651	3.097864	1.691549
H	1.509507	1.621151	1.764759
H	-1.380054	2.297360	-1.419183
H	-1.089798	3.509288	-0.172381
H	-1.948156	2.031315	0.245924
H	1.121871	2.306060	-1.948864
H	2.309661	2.154621	-0.637780
H	1.255100	3.562388	-0.718522
H	3.065755	0.034099	-1.196149
H	3.739258	-1.094678	-0.023666
H	3.064486	0.428389	0.540653
H	0.591496	-2.819531	-0.966009
H	2.344393	-2.962899	-0.818329
H	1.659169	-1.917389	-2.067168
H	0.544926	-2.235051	1.528492
H	1.483672	-0.853658	2.153166
H	2.303415	-2.316480	1.599985

(Hartree/Particle)
HF(PBE1PBE)= -1077.3421373
Zero-point correction= 0.322037
Thermal correction to Energy= 0.340661
Thermal correction to Enthalpy= 0.341605
Thermal correction to Gibbs Free Energy= 0.277096
Sum of electronic and zero-point Energies= -1077.020100
Sum of electronic and thermal Energies= -1077.001477
Sum of electronic and thermal Enthalpies= -1077.000533
Sum of electronic and thermal Free Energies= -1077.065041
HF(MP2)= -1074.0066676
MP2= -1075.7820361

$[Ph_2PPMe_2]^{+\cdot}$

P	0.102291	2.354287	0.532230
P	0.020178	0.487692	-0.592916
C	-0.923620	3.507355	-0.460082
C	1.819806	2.946952	0.309801
H	-1.940119	3.120401	-0.553826
H	-0.502922	3.683842	-1.451828
H	-0.970851	4.454730	0.083997
H	2.522193	2.234356	0.745387
H	1.902279	3.886317	0.864116
H	2.070721	3.134980	-0.736176
H	2.234818	-3.300756	1.666011
C	2.343421	-2.468677	0.979047
C	1.250063	-1.665524	0.695131
H	0.289389	-1.867131	1.156375
C	1.399916	-0.582021	-0.182475
C	2.640075	-0.331480	-0.789778
H	2.749456	0.482675	-1.499635
C	3.724489	-1.143926	-0.498715
H	4.682638	-0.955563	-0.970126
C	3.576311	-2.209419	0.385373
H	4.425732	-2.846227	0.608266
C	-1.823759	-1.486525	-0.997476
C	-2.446173	0.113920	0.719322
C	-3.648362	-0.555480	0.893875
C	-3.027098	-2.148499	-0.810288
C	-1.524129	-0.352242	-0.226715
C	-3.940135	-1.682434	0.131094
H	-1.120856	-1.846611	-1.742747
H	-4.881463	-2.201994	0.274000
H	-3.255911	-3.025834	-1.405005
H	-4.354505	-0.204423	1.638373
H	-2.212461	0.969541	1.344427

(Hartree/Particle)
HF(PBE1PBE)= -1224.8199734
Zero-point correction= 0.260294
Thermal correction to Energy= 0.277060
Thermal correction to Enthalpy= 0.278005
Thermal correction to Gibbs Free Energy= 0.213115
Sum of electronic and zero-point Energies= -1224.559679
Sum of electronic and thermal Energies= -1224.542913
Sum of electronic and thermal Enthalpies= -1224.541969
Sum of electronic and thermal Free Energies= -1224.606858
HF(MP2)= -1220.8708385
MP2=-1222.9801716

$[(N^iPr_2)_2PPMe_2]^{+\cdot}$

P	0.422676	2.335188	0.599756
N	-1.361295	-0.289574	-0.038285
C	-1.162664	3.256191	0.525086
C	-1.421013	-1.494834	0.815197
C	-2.537847	0.321051	-0.709425
C	1.438541	3.233419	-0.643116
H	2.357614	2.682154	-0.849770
H	0.902337	3.412399	-1.577267
C	-2.269687	-2.629067	0.257413
C	-3.729119	0.530866	0.214677
C	-2.928945	-0.404836	-1.996747
H	-2.186284	1.314779	-1.018531
H	-2.137714	-3.502931	0.901046
H	-1.959446	-2.908334	-0.751380
H	-3.336355	-2.396669	0.247850
H	-4.203439	-0.411441	0.495444
H	-4.479549	1.125013	-0.312919
H	-3.456940	1.064356	1.127305
H	-3.388249	-1.374424	-1.804250
H	-2.060448	-0.549918	-2.643922
H	-3.655574	0.205087	-2.540488
P	0.102213	0.405976	-0.502199
H	-1.518735	3.428498	-0.493344
H	-0.975427	4.229201	0.990570
H	-1.929127	2.752073	1.114548
C	-1.795876	-1.140902	2.255282
H	-1.192892	-0.310205	2.632471
H	-1.608762	-2.007916	2.895103
H	-2.849603	-0.877226	2.356626
H	-0.389177	-1.851943	0.828211
H	1.717040	4.195877	-0.203977
N	1.397039	-0.536419	-0.068777
C	2.009251	-0.570443	1.290429
C	2.108287	-1.259054	-1.164444
C	1.238143	-2.345108	-1.781452

H	1.806517	-2.908445	-2.526133
H	0.369659	-1.914734	-2.292846
H	0.882202	-3.047943	-1.024182
C	2.670997	-0.311900	-2.216762
H	3.325208	0.437864	-1.765085
H	1.870928	0.204147	-2.759046
H	3.255460	-0.870649	-2.952515
H	2.952579	-1.744616	-0.669710
C	2.175845	-1.992296	1.818457
H	2.916936	-2.561495	1.250938
H	1.238614	-2.553042	1.814266
H	2.530047	-1.948500	2.851691
C	3.330771	0.185675	1.330301
H	3.740893	0.168253	2.343517
H	3.202145	1.231388	1.040398
H	4.074699	-0.267921	0.668724
H	1.301197	-0.050815	1.943351

(Hartree/Particle)
HF(PBE1PBE)= -1345.0738608
Zero-point correction= 0.470054
Thermal correction to Energy= 0.496443
Thermal correction to Enthalpy= 0.497387
Thermal correction to Gibbs Free Energy= 0.414406
Sum of electronic and zero-point Energies= -1344.603806
Sum of electronic and thermal Energies= -1344.577418
Sum of electronic and thermal Enthalpies= -1344.576474
Sum of electronic and thermal Free Energies= -1344.659455
HF(MP2)= -1340.2739587
MP2= -1343.0431688

[R_2PPMe_2]

[Me_2PPMe_2]

P	0.469997	-1.004283	-0.515285
P	-0.469997	1.004283	-0.515285
C	-1.069747	-2.032777	-0.474344
C	1.069747	2.032777	-0.474344
C	-1.069747	1.262713	1.219382
H	-1.717371	-1.811204	0.379633
H	-0.788641	-3.088621	-0.431732
H	-1.633362	-1.871968	-1.396193
C	1.069747	-1.262713	1.219382
H	1.886733	-0.569801	1.437208
H	1.473762	-2.276500	1.296080
H	0.287585	-1.141324	1.974047
H	1.633362	1.871968	-1.396193
H	1.717371	1.811204	0.379633
H	0.788641	3.088621	-0.431732
H	-1.886733	0.569801	1.437208
H	-1.473762	2.276500	1.296080
H	-0.287585	1.141324	1.974047

(Hartree/Particle)
HF(PBE1PBE)= -841.9686727
Zero-point correction= 0.152209
Thermal correction to Energy= 0.162552
Thermal correction to Enthalpy= 0.163496
Thermal correction to Gibbs Free Energy= 0.118010
Sum of electronic and zero-point Energies= -841.816464
Sum of electronic and thermal Energies= -841.806120
Sum of electronic and thermal Enthalpies= -841.805176
Sum of electronic and thermal Free Energies= -841.850663
HF(MP2)= -839.9956301
MP2= -840.8636354

[Et_2PPMe_2]

P	-1.598559	0.010559	-0.595374
P	0.343960	-0.003037	0.498931
C	-2.379575	1.448481	0.278153
C	-2.435795	-1.368640	0.318896
C	1.206614	1.316302	-0.508041
C	1.040815	-1.555248	-0.268296
H	-1.895002	2.379456	-0.026817
H	-2.323547	1.359672	1.366980
H	-3.429016	1.512845	-0.023258
H	-2.035695	-2.330857	-0.009064
H	-3.500682	-1.355094	0.069672
H	-2.323908	-1.284644	1.404308
H	0.473214	2.106808	-0.701680
H	1.480303	0.904660	-1.486146
C	2.417411	1.916644	0.200060
H	2.131628	2.361682	1.157287
H	2.877158	2.699520	-0.411703
H	3.184420	1.165797	0.407629
C	2.511415	-1.785255	0.062124
H	3.149283	-1.018992	-0.386926
H	2.846977	-2.753427	-0.322158
H	2.685617	-1.774898	1.142286
H	0.888638	-1.532288	-1.353892
H	0.444290	-2.386772	0.122759

(Hartree/Particle)
HF(PBE1PBE)= -920.5142732
Zero-point correction= 0.209726
Thermal correction to Energy= 0.222847
Thermal correction to Enthalpy= 0.223792
Thermal correction to Gibbs Free Energy= 0.170387
Sum of electronic and zero-point Energies= -920.304547
Sum of electronic and thermal Energies= -920.291426
Sum of electronic and thermal Enthalpies= -920.290482
Sum of electronic and thermal Free Energies= -920.343886
HF(MP2)= -918.077533
MP2= -919.255144

[$^{i}Pr_2PPMe_2$]

P	1.481165	-1.112401	-0.013917
P	-0.317735	0.046367	-0.625763
C	2.734773	-0.446613	-1.203614
C	-0.255209	1.644045	0.371152
C	-1.705875	-0.887646	0.256639
H	2.921480	0.626277	-1.118516
H	2.399394	-0.662812	-2.220495
H	3.675571	-0.980258	-1.039884
C	2.085126	-0.383225	1.579900
H	2.250528	0.696992	1.547871
H	3.030372	-0.871401	1.835816
H	1.373280	-0.609956	2.377904
C	0.812230	2.571021	-0.203536
H	0.007538	1.393226	1.406395
C	-1.606512	2.355482	0.380541
C	-1.461921	-2.388046	0.384076
C	-3.009724	-0.650429	-0.509643
H	-1.798448	-0.462943	1.264918
H	-1.947214	2.573920	-0.637387
H	-1.515599	3.310593	0.910401
H	-2.382921	1.775512	0.884131
H	-1.248007	-2.841237	-0.589539
H	-2.360250	-2.871001	0.785311
H	-0.622924	-2.625974	1.040533
H	1.814150	2.140280	-0.150930
H	0.830292	3.516799	0.349675
H	0.604053	2.801013	-1.253612
H	-2.956137	-1.117143	-1.498440
H	-3.230479	0.408160	-0.661030

H	-3.853467	-1.097059	0.028987

(Hartree/Particle)
HF(PBE1PBE)= -999.0564417
Zero-point correction= 0.266383
Thermal correction to Energy= 0.281947
Thermal correction to Enthalpy= 0.282891
Thermal correction to Gibbs Free Energy= 0.224993
Sum of electronic and zero-point Energies= -998.790058
Sum of electronic and thermal Energies= -998.774495
Sum of electronic and thermal Enthalpies= -998.773551
Sum of electronic and thermal Free Energies= -998.831449
HF(MP2)= -996.15 21895
MP2= -997.6458168

[^{t}Bu$_2$PPMe$_2$]

P	-1.490896	-1.296009	-0.053704
P	0.159111	0.013491	-0.752418
C	-2.869991	-0.782631	-1.176083
C	1.627309	-0.961976	-0.018882
C	0.064150	1.758453	0.016203
C	-2.171040	-0.753332	1.585540
H	-2.545846	-0.910342	-2.211127
H	-3.202986	0.248004	-1.035434
H	-3.717120	-1.452715	-1.002012
H	-1.427336	-0.915559	2.368287
H	-3.027453	-1.399293	1.804383
H	-2.508041	0.285086	1.621318
C	-1.334022	2.288967	-0.317299
C	1.069820	2.641202	-0.736615
C	0.308945	1.870640	1.519397
C	1.750594	-2.200771	-0.916238
C	1.464592	-1.422885	1.430588
C	2.914540	-0.146725	-0.156807
H	-0.375359	1.245951	2.096585
H	0.159170	2.910915	1.837499
H	1.331581	1.596463	1.789402
H	-1.559555	2.182282	-1.383165
H	-1.384510	3.356507	-0.070811
H	-2.116571	1.782642	0.250629
H	0.901851	2.598668	-1.816611
H	2.106073	2.359494	-0.544661
H	0.945419	3.683246	-0.415416
H	3.046140	0.236519	-1.173371
H	3.772362	-0.792045	0.068488
H	2.951148	0.695208	0.538892
H	0.845620	-2.813481	-0.881176
H	2.590798	-2.816741	-0.572274
H	1.934633	-1.921132	-1.957512
H	0.612321	-2.100909	1.535510
H	1.335064	-0.589734	2.124194
H	2.359978	-1.976907	1.741380

(Hartree/Particle)
HF(PBE1PBE)= -1077.5986797
Zero-point correction= 0.322389
Thermal correction to Energy= 0.340286
Thermal correction to Enthalpy= 0.341230
Thermal correction to Gibbs Free Energy= 0.280086
Sum of electronic and zero-point Energies= -1077.276290
Sum of electronic and thermal Energies= -1077.258394
Sum of electronic and thermal Enthalpies= -1077.257450
Sum of electronic and thermal Free Energies= -1077.318593
HF(MP2)= -1074.2252644
MP2= -1076.0417889

[Ph$_2$PPMe$_2$]

P	0.233073	2.412137	0.143044
P	-0.017124	0.568687	-1.085179
C	1.908245	2.892248	-0.475967
C	0.668460	1.820782	1.838968
H	1.847113	3.144853	-1.537016
H	2.655733	2.105604	-0.335169
H	2.231460	3.785217	0.066172
H	-0.203714	1.346196	2.295270
H	0.930245	2.693387	2.445612
H	1.503977	1.115678	1.844400
H	1.839186	-2.771025	2.170568
C	2.008346	-2.195105	1.265954
C	1.010477	-1.354269	0.789592
H	0.067243	-1.283956	1.321545
c	1.205350	-0.608685	-0.378404
c	2.425694	-0.728684	-1.052394
H	2.581123	-0.175044	-1.974252
C	3.429513	-1.560152	-0.567134
H	4.368881	-1.642041	-1.104773
C	3.221969	-2.297370	0.592339
H	3.999592	-2.954933	0.967404
C	-1.926497	-1.430059	-0.695078
C	-2.598972	0.730722	0.113678
C	-3.841950	0.209955	0.459152
C	-3.164948	-1.948969	-0.343126
C	-1.618523	-0.084226	-0.459122
C	-4.129263	-1.130653	0.236961
H	-1.187356	-2.080000	-1.154361
H	-5.098498	-1.535611	0.509138
H	-3.380030	-2.996495	-0.529789
H	-4.587593	0.859832	0.906539
H	-2.393996	1.782777	0.288213

(Hartree/Particle)
HF(PBE1PBE)= -1225.0736773
Zero-point correction= 0.259965
Thermal correction to Energy= 0.276382
Thermal correction to Enthalpy= 0.277326
Thermal correction to Gibbs Free Energy= 0.214744
Sum of electronic and zero-point Energies= -1224.813712
Sum of electronic and thermal Energies= -1224.797296
Sum of electronic and thermal Enthalpies= -1224.796351
Sum of electronic and thermal Free Energies= -1224.858934
HF(MP2)= -1221.085887
MP2= -1223.3031267

[(N^{i}Pr$_2$)$_2$PPMe$_2$]

P	-0.337720	-2.447603	0.024814
N	1.306470	0.361435	-0.083025
C	0.933987	-2.629387	1.356945
C	1.396943	0.792873	1.316696
C	2.345978	0.661914	-1.082165
C	0.601586	-3.258186	-1.353531
H	0.053444	-3.129141	-2.289301
H	1.610941	-2.855440	-1.480734
C	1.042286	2.268945	1.536911
C	3.568211	-0.258445	-1.017211
C	2.777804	2.126816	-1.149469
H	1.854628	0.441454	-2.038140
H	0.889578	2.460253	2.604926
H	0.126354	2.526617	1.004956
H	1.838329	2.934726	1.198122
H	4.204422	-0.054368	-0.153286
H	4.180615	-0.123317	-1.915262
H	3.262363	-1.306985	-0.974258
H	3.370278	2.425919	-0.280209
H	1.915272	2.792591	-1.227502
H	3.405742	2.277276	-2.033430
P	-0.117937	-0.342510	-0.756259

H	1.885698	-2.173371	1.073297
H	1.090002	-3.697842	1.534053
H	0.585386	-2.184229	2.291779
C	2.726554	0.486021	2.006048
H	3.016282	-0.561008	1.903249
H	2.636001	0.708895	3.073991
H	3.536836	1.108071	1.616370
H	0.630750	0.208397	1.838330
H	0.672535	-4.330166	-1.146282
N	-1.500703	0.395857	-0.062172
C	-2.139259	-0.008974	1.199376
C	-2.321592	1.219041	-0.973872
C	-1.565745	2.442520	-1.481789
H	-2.209873	3.049154	-2.126362
H	-0.694017	2.139497	-2.070973
H	-1.220565	3.067909	-0.654960
C	-2.909116	0.427063	-2.142851
H	-3.469187	-0.441964	-1.790803
H	-2.111592	0.073498	-2.803224
H	-3.583979	1.056810	-2.732276
H	-3.158227	1.584978	-0.368861
C	-2.370852	1.165935	2.149934
H	-3.074468	1.891830	1.729300
H	-1.440318	1.688476	2.374701
H	-2.801376	0.807461	3.090380
C	-3.434605	-0.796104	0.997996
H	-3.817474	-1.139491	1.964423
H	-3.259784	-1.671647	0.369752
H	-4.213714	-0.178296	0.539832
H	-1.429458	-0.686079	1.688350

(Hartree/Particle)
HF(PBE1PBE)= -1345.2979095
Zero-point correction= 0.469209
Thermal correction to Energy= 0.495179
Thermal correction to Enthalpy= 0.496124
Thermal correction to Gibbs Free Energy= 0.415831
Sum of electronic and zero-point Energies= -1344.828701
Sum of electronic and thermal Energies= -1344.802730
Sum of electronic and thermal Enthalpies= -1344.801786
Sum of electronic and thermal Free Energies= -1344.882079
HF(MP2)= -1340.4713892
MP2=-1343.2702158

$[RPPMe_3]^{+\cdot}$

$[MePPMe_3]^{+\cdot}$

P	-0.622317	-0.030647	0.000000
P	1.353833	0.952409	-0.000002
C	-1.857705	1.285547	-0.000030
C	-0.868048	-1.059423	1.469252
C	2.413464	-0.553531	0.000000
H	-1.735375	1.908977	-0.888650
H	-1.735380	1.909011	0.888567
H	-2.861099	0.852400	-0.000025
H	-0.155592	-1.886655	1.474658
H	-1.883317	-1.465664	1.459933
H	-0.730542	-0.458771	2.370919
C	-0.868031	-1.059474	-1.469219
H	-0.730508	-0.458855	-2.370906
H	-1.883303	-1.465709	-1.459901
H	-0.155580	-1.886710	-1.474585
H	2.264455	-1.171070	0.891184
H	3.450961	-0.211036	0.000004
H	2.264461	-1.171067	-0.891187

(Hartree/Particle)
HF(PBE1PBE)=-841.7197568
Zero-point correction= 0.152553
Thermal correction to Energy= 0.163156
Thermal correction to Enthalpy= 0.164100
Thermal correction to Gibbs Free Energy= 0.115942
Sum of electronic and zero-point Energies= -841.567204
Sum of electronic and thermal Energies= -841.556601
Sum of electronic and thermal Enthalpies= -841.555657
Sum of electronic and thermal Free Energies= -841.603815
HF(MP2)=-839.7864093
MP2=-840.6187595

$[EtPPMe_3]^{+\cdot}$

P	1.062803	-0.036089	0.000000
P	-0.896414	0.976429	0.000005
C	1.298054	-1.068269	1.469163
C	2.321772	1.258534	0.000034
C	-1.997703	-0.513593	0.000007
H	0.576397	-1.887357	1.475170
H	1.166860	-0.465777	2.370519
H	2.309002	-1.485084	1.459810
H	2.210114	1.884187	-0.888364
H	3.317396	0.807723	0.000030
H	2.210104	1.884149	0.888457
C	1.298073	-1.068208	-1.469205
H	0.576412	-1.887292	-1.475258
H	2.309019	-1.485026	-1.459854
H	1.166894	-0.465676	-2.370536
H	-1.781343	-1.128825	0.882148
H	-1.781331	-1.128841	-0.882119
C	-3.462394	-0.078136	-0.000006
H	-3.706383	0.514430	0.884986
H	-4.109412	-0.958081	-0.000002
H	-3.706372	0.514412	-0.885013

(Hartree/Particle)
HF(PBE1PBE)=-880.9950088
Zero-point correction= 0.181298
Thermal correction to Energy= 0.193299
Thermal correction to Enthalpy= 0.194243
Thermal correction to Gibbs Free Energy= 0.141641
Sum of electronic and zero-point Energies= -880.813710
Sum of electronic and thermal Energies= -880.801710
Sum of electronic and thermal Enthalpies= -880.800766
Sum of electronic and thermal Free Energies= -880.853368
HF(MP2)=-878.830612
MP2=-879.815711

$[^iPrPPMe_3]^{+\cdot}$

P	1.287886	0.000468	0.050722
P	-0.578546	-0.543073	-0.979476
C	1.337964	1.734660	0.569025
C	2.647033	-0.290102	-1.102797
C	-1.830240	0.040807	0.284560
H	0.534254	1.940302	1.278724
H	1.227273	2.385556	-0.300700
H	2.299104	1.938421	1.049455
H	2.647859	-1.336061	-1.417620
H	3.599781	-0.058729	-0.619717
H	2.526748	0.343387	-1.984364
C	1.550234	-1.035743	1.513210
H	0.743753	-0.884637	2.233613
H	2.502041	-0.768946	1.980903
H	1.575267	-2.087302	1.219889
C	-2.373415	1.408416	-0.143403

H	-1.364823	0.129587	1.273095
C	-2.939636	-1.011322	0.352133
H	-3.410729	-1.156031	-0.625132
H	-3.716666	-0.676460	1.045594
H	-2.573284	-1.980279	0.699556
H	-1.601790	2.182020	-0.176454
H	-3.139331	1.730700	0.568836
H	-2.841199	1.357238	-1.130742

(Hartree/Particle)
HF(PBE1PBE)=-920.2699166
Zero-point correction= 0.209783
Thermal correction to Energy= 0.223004
Thermal correction to Enthalpy= 0.223948
Thermal correction to Gibbs Free Energy= 0.169847
Sum of electronic and zero-point Energies= -920.060133
Sum of electronic and thermal Energies= -920.046913
Sum of electronic and thermal Enthalpies= -920.045969
Sum of electronic and thermal Free Energies= -920.100069
HF(MP2)=-917.872703
MP2=-919.0145539

[^{t}BuPPMe$_3$]$^{+\cdot}$

P	-1.458496	0.001633	-0.000567
P	0.413203	-1.182296	-0.006915
C	-1.567807	1.384774	-1.166952
C	1.825216	0.058825	0.002059
C	-1.876028	0.615180	1.653474
H	-1.347516	1.037288	-2.178236
H	-0.868953	2.176889	-0.895348
H	-2.586287	1.783131	-1.140762
H	-1.858543	-0.212868	2.365362
H	-2.880230	1.047803	1.630764
H	-1.160830	1.374860	1.970860
C	-2.731306	-1.196923	-0.466853
H	-2.542377	-1.565274	-1.477502
H	-3.715679	-0.723020	-0.432504
H	-2.714127	-2.042746	0.224048
C	2.031866	0.600131	-1.417401
C	3.031597	-0.804234	0.404572
C	1.642581	1.196442	1.005115
H	0.840185	1.882004	0.715922
H	2.563728	1.787684	1.046259
H	1.448857	0.829372	2.017283
H	2.165098	-0.202985	-2.147520
H	2.941841	1.210414	-1.432676
H	1.207147	1.235236	-1.749971
H	3.181954	-1.644872	-0.279518
H	2.932709	-1.200421	1.419106
H	3.935706	-0.187721	0.372582

(Hartree/Particle)
HF(PBE1PBE)=-959.5439759
Zero-point correction= 0.237680
Thermal correction to Energy= 0.252115
Thermal correction to Enthalpy= 0.253059
Thermal correction to Gibbs Free Energy= 0.197343
Sum of electronic and zero-point Energies= -959.306296
Sum of electronic and thermal Energies= -959.291861
Sum of electronic and thermal Enthalpies= -959.290917
Sum of electronic and thermal Free Energies= -959.346633
HF(MP2)=-956.912915
MP2=-958.2139936

[PhPPMe$_3$]$^{+\cdot}$

P	-1.959922	-0.118942	0.000001
P	-0.354968	1.386868	-0.000004
C	-2.011747	-1.164112	1.480446
C	-2.011752	-1.164114	-1.480444
H	-2.041882	-0.526314	2.366537
H	-1.132575	-1.806300	1.540032
H	-2.912510	-1.783580	1.450930
H	-2.041885	-0.526315	-2.366535
H	-2.912516	-1.783579	-1.450927
H	-1.132580	-1.806302	-1.540030
C	-3.467636	0.880059	0.000002
H	-3.493329	1.514863	0.888381
H	-4.342437	0.225003	0.000004
H	-3.493331	1.514862	-0.888377
H	2.750349	-2.565282	-0.000003
C	2.621195	-1.488435	-0.000002
C	1.348519	-0.948801	-0.000002
H	0.500925	-1.624143	-0.000002
C	1.163208	0.447927	0.000000
C	2.308019	1.277383	0.000002
H	2.190137	2.357671	0.000003
C	3.578482	0.729296	0.000002
H	4.445706	1.380039	0.000004
C	3.739173	-0.653432	0.000000
H	4.734512	-1.084127	0.000000

(Hartree/Particle)
HF(PBE1PBE)=-1033.2797511
Zero-point correction= 0.206891
Thermal correction to Energy= 0.220361
Thermal correction to Enthalpy= 0.221306
Thermal correction to Gibbs Free Energy= 0.165709
Sum of electronic and zero-point Energies= -1033.072860
Sum of electronic and thermal Energies= -1033.059390
Sum of electronic and thermal Enthalpies= -1033.058446
Sum of electronic and thermal Free Energies= -1033.114042
HF(MP2)=-1030.3464193
MP2=-1031.806394

[^{i}Pr$_2$NPPMe$_3$]$^{+\cdot}$

P	-2.132384	-0.098559	0.053866
N	0.985829	-0.248735	-0.092192
C	-2.563449	1.470821	-0.752591
C	-3.415212	-1.285200	-0.425528
C	2.239577	-1.051507	-0.201818
C	0.962763	1.196885	0.205229
C	-2.262976	0.132589	1.849948
H	-2.082500	-0.822720	2.346628
H	-1.530477	0.860160	2.203218
C	2.785243	-1.468353	1.161099
C	1.147425	2.051232	-1.049315
C	1.898757	1.618125	1.329932
H	-0.051537	1.387532	0.574719
H	3.541966	-2.243908	1.015337
H	1.993946	-1.885738	1.788380
H	3.258818	-0.643382	1.693953
H	2.172420	2.011120	-1.419383
H	0.921590	3.096240	-0.817233
H	0.482536	1.723626	-1.853081
H	2.950106	1.532383	1.051190
H	1.728339	1.038038	2.238919
H	1.711945	2.670595	1.559185
P	-0.327818	-1.153584	-0.644459
H	-1.851791	2.255386	-0.492720
H	-3.564809	1.774877	-0.434812
H	-2.556460	1.329638	-1.835355
H	-4.397611	-0.887124	-0.160269
H	-3.258673	-2.234100	0.091233

H	-3.379241	-1.457896	-1.503487
C	3.296219	-0.432514	-1.105949
H	2.893111	-0.196267	-2.093031
H	4.104901	-1.155622	-1.239374
H	3.739321	0.469425	-0.678712
H	1.913513	-1.979126	-0.694809
H	-3.267274	0.487667	2.097698

(Hartree/Particle)
HF(PBE1PBE)=-1093.40634
Zero-point correction= 0.312212
Thermal correction to Energy= 0.330418
Thermal correction to Enthalpy= 0.331362
Thermal correction to Gibbs Free Energy= 0.266722
Sum of electronic and zero-point Energies= -1093.094128
Sum of electronic and thermal Energies= -1093.075922
Sum of electronic and thermal Enthalpies= -1093.074978
Sum of electronic and thermal Free Energies= -1093.139618
HF(MP2)= -1090.0390013
MP2=-1091.8385253

[RPPMe$_3$]

[MePPMe$_3$]

P	-0.529667	0.007989	0.000000
P	1.342819	0.943526	0.000001
C	-1.801801	1.306376	-0.000006
C	-0.952922	-1.059341	1.429120
C	2.395598	-0.621344	-0.000001
H	-1.678228	1.929769	-0.887212
H	-1.678210	1.929795	0.887179
H	-2.800965	0.864355	0.000011
H	-0.233290	-1.878813	1.492695
H	-1.960155	-1.476879	1.338045
H	-0.875622	-0.462354	2.339522
C	-0.952917	-1.059348	-1.429115
H	-0.875605	-0.462368	-2.339521
H	-1.960153	-1.476878	-1.338046
H	-0.233291	-1.878826	-1.492679
H	2.268705	-1.241662	0.892411
H	3.433088	-0.275262	0.000000
H	2.268704	-1.241659	-0.892414

(Hartree/Particle)
HF(PBE1PBE)=-841.9453981
Zero-point correction= 0.152373
Thermal correction to Energy= 0.162836
Thermal correction to Enthalpy= 0.163780
Thermal correction to Gibbs Free Energy= 0.117188
Sum of electronic and zero-point Energies= -841.793025
Sum of electronic and thermal Energies= -841.782563
Sum of electronic and thermal Enthalpies= -841.781618
Sum of electronic and thermal Free Energies= -841.828211
HF(MP2)=-839.9695423
MP2=-840.8399507

[EtPPMe$_3$]

P	0.968960	0.000654	0.000001
P	-0.878731	0.982686	0.000003
C	1.368106	-1.076162	1.429298
C	2.273903	1.266608	-0.000008
C	-1.984071	-0.555756	-0.000001
H	0.632862	-1.881696	1.491320
H	1.301116	-0.478375	2.339993
H	2.367406	-1.512600	1.339203
H	2.166199	1.892980	-0.887159
H	3.261371	0.798863	0.000017
H	2.166174	1.893019	0.887112
C	1.368100	-1.076170	-1.429294
H	0.632865	-1.881713	-1.491300
H	2.367406	-1.512595	-1.339207
H	1.301092	-0.478390	-2.339992
H	-1.802658	-1.176103	0.884868
H	-1.802656	-1.176100	-0.884872
C	-3.441571	-0.104886	-0.000001
H	-3.665061	0.502039	0.882811
H	-4.121299	-0.963267	-0.000003
H	-3.665059	0.502042	-0.882812

(Hartree/Particle)
HF(PBE1PBE)=-881.2186206
Zero-point correction= 0.181136
Thermal correction to Energy= 0.192951
Thermal correction to Enthalpy= 0.193895
Thermal correction to Gibbs Free Energy= 0.143387
Sum of electronic and zero-point Energies= -881.037484
Sum of electronic and thermal Energies= -881.025670
Sum of electronic and thermal Enthalpies= -881.024725
Sum of electronic and thermal Free Energies= -881.075234
HF(MP2)=-879.011487
MP2=-880.035935

[^{i}PrPPMe$_3$]

P	1.198271	-0.018365	0.021182
P	-0.555504	-0.812820	-0.799585
C	1.506971	1.787720	-0.076291
C	2.593671	-0.759907	-0.879444
C	-1.836561	0.093884	0.278931
H	0.752176	2.316949	0.509801
H	1.417208	2.099216	-1.118539
H	2.499269	2.044381	0.307401
H	2.539205	-1.846368	-0.793165
H	3.543353	-0.406755	-0.470273
H	2.526336	-0.487760	-1.934099
C	1.546063	-0.375942	1.785475
H	0.741574	0.028487	2.404121
H	2.496091	0.062333	2.105904
H	1.568517	-1.458969	1.917568
C	-2.297576	1.412597	-0.336938
H	-1.411959	0.295354	1.271180
C	-3.022345	-0.851013	0.469743
H	-3.444236	-1.143780	-0.498757
H	-3.820895	-0.365146	1.043276
H	-2.725719	-1.764749	0.990190
H	-1.471723	2.116978	-0.465593
H	-3.063475	1.893633	0.286560
H	-2.728579	1.239935	-1.328390

(Hartree/Particle)
HF(PBE1PBE)=-920.4930702
Zero-point correction= 0.209283
Thermal correction to Energy= 0.222309
Thermal correction to Enthalpy= 0.223254
Thermal correction to Gibbs Free Energy= 0.170207
Sum of electronic and zero-point Energies= -920.283787
Sum of electronic and thermal Energies= -920.270761
Sum of electronic and thermal Enthalpies= -920.269817
Sum of electronic and thermal Free Energies= -920.322864
HF(MP2)=-918.0528505
MP2=-919.2351469

[^{t}BuPPMe$_3$]

P	-1.372082	-0.033134	0.000361
P	0.371050	-1.206257	0.022244
C	-1.634166	1.222949	-1.310547
C	1.807986	0.055289	0.004100
C	-1.891557	0.868411	1.515190
H	-1.453307	0.757935	-2.281126
H	-0.930987	2.048345	-1.181679
H	-2.653867	1.617908	-1.270535
H	-1.912206	0.155830	2.341925
H	-2.880366	1.320094	1.387683
H	-1.166666	1.648257	1.752854
C	-2.721746	-1.229278	-0.253696
H	-2.622565	-1.678738	-1.242589
H	-3.691778	-0.733384	-0.166417
H	-2.646132	-2.017123	0.497998
C	2.066024	0.595795	-1.404157
C	3.015896	-0.785121	0.435511
C	1.647229	1.217942	0.981352
H	0.853459	1.905230	0.667698
H	2.574316	1.805807	1.029502
H	1.416684	0.862664	1.989660
H	2.207455	-0.221100	-2.116518
H	2.968777	1.223262	-1.416369
H	1.232570	1.205303	-1.764695
H	3.153881	-1.650990	-0.220548
H	2.897728	-1.153983	1.458396
H	3.930484	-0.180361	0.389164

(Hartree/Particle)
HF(PBE1PBE)=-959.7661244
Zero-point correction= 0.237285
Thermal correction to Energy= 0.251444
Thermal correction to Enthalpy= 0.252388
Thermal correction to Gibbs Free Energy= 0.198018
Sum of electronic and zero-point Energies= -959.528839
Sum of electronic and thermal Energies= -959.514681
Sum of electronic and thermal Enthalpies= -959.513737
Sum of electronic and thermal Free Energies= -959.568106
HF(MP2)=-957.0917901
MP2=-958.4346381

[PhPPMe$_3$]

P	-1.762748	0.000000	0.172237
P	-0.597180	0.000003	-1.576105
C	-1.587786	1.429556	1.299217
C	-1.587789	-1.429562	1.299210
H	-1.835822	2.339464	0.749742
H	-0.547189	1.494092	1.625669
H	-2.237179	1.331237	2.174222
H	-1.835829	-2.339467	0.749730
H	-2.237179	-1.331246	2.174216
H	-0.547191	-1.494104	1.625659
C	-3.513052	0.000003	-0.321841
H	-3.716717	0.887307	-0.923914
H	-4.160297	-0.000003	0.558369
H	-3.716717	-0.887295	-0.923925
H	3.407015	-2.144530	0.508763
C	2.921259	-1.201328	0.276819
C	1.679094	-1.199142	-0.349764
H	1.203302	-2.138962	-0.613482
C	1.025905	0.000001	-0.664167
C	1.679094	1.199144	-0.349760
H	1.203303	2.138964	-0.613475
C	2.921259	1.201326	0.276823
H	3.407016	2.144528	0.508769
C	3.544760	-0.000001	0.596238
H	4.515931	-0.000002	1.081027

(Hartree/Particle)
HF(PBE1PBE)=-1033.5046253
Zero-point correction= 0.206540
Thermal correction to Energy= 0.219829
Thermal correction to Enthalpy= 0.220773
Thermal correction to Gibbs Free Energy= 0.166050
Sum of electronic and zero-point Energies= -1033.298085
Sum of electronic and thermal Energies= -1033.284797
Sum of electronic and thermal Enthalpies= -1033.283852
Sum of electronic and thermal Free Energies= -1033.338576
HF=-1030.523428
MP2=-1032.066978

[^{i}Pr$_2$NPPMe$_3$]

P	-2.073473	-0.018276	-0.014773
N	0.832156	-0.045292	0.148599
C	-2.010564	-0.741305	-1.690008
C	-3.616197	-0.620631	0.741820
C	1.544239	-1.179987	-0.467635
C	1.497644	1.238553	0.430455
C	-2.394305	1.755179	-0.348186
H	-2.572992	2.259768	0.603380
H	-1.504854	2.192158	-0.806965
C	2.381234	-2.016402	0.507120
C	1.669448	2.136952	-0.799649
C	2.806255	1.148064	1.217984
H	0.783968	1.750965	1.083556
H	2.702165	-2.946727	0.025407
H	1.782979	-2.283245	1.383363
H	3.276336	-1.488776	0.844350
H	2.484770	1.816871	-1.451375
H	1.891486	3.160750	-0.479243
H	0.752448	2.159418	-1.395536
H	3.613790	0.703902	0.628733
H	2.674018	0.554526	2.126427
H	3.132568	2.151267	1.512419
P	-0.446901	-0.520724	1.267714
H	-1.051253	-0.467961	-2.136262
H	-2.823895	-0.374670	-2.322882
H	-2.060313	-1.828373	-1.607533
H	-4.482956	-0.320815	0.148083
H	-3.708621	-0.204626	1.747465
H	-3.580773	-1.708736	0.815911
C	2.363843	-0.814950	-1.701991
H	1.772769	-0.247165	-2.425636
H	2.705813	-1.733968	-2.188251
H	3.255083	-0.232219	-1.453515
H	0.739922	-1.840758	-0.811942
H	-3.251524	1.897619	-1.013706

(Hartree/Particle)
HF(PBE1PBE)=-1093.6083356
Zero-point correction= 0.310750
Thermal correction to Energy= 0.328954
Thermal correction to Enthalpy= 0.329898
Thermal correction to Gibbs Free Energy= 0.265631
Sum of electronic and zero-point Energies= -1093.297585
Sum of electronic and thermal Energies= -1093.279381
Sum of electronic and thermal Enthalpies= -1093.278437
Sum of electronic and thermal Free Energies= -1093.342704
HF(MP2)=-1090.2082411
MP2=-1092.039461

[RPPMe$_2$]$^+$

[MePPMe$_2$]$^+$

P	-0.644323	0.030835	-0.002495
P	1.139765	-0.881236	0.001480
C	-2.111733	-1.015341	0.000652
C	2.346358	0.506512	-0.000349
H	-2.711709	-0.801471	0.889176
H	-1.807930	-2.063729	0.009775
H	-2.706680	-0.815724	-0.894522
C	-1.027678	1.795717	0.001642
H	-1.579157	2.038591	0.914375
H	-1.650036	2.022936	-0.868570
H	-0.115512	2.390853	-0.041557
H	2.946553	0.408239	-0.910505
H	3.016278	0.342121	0.849420
H	1.934882	1.512867	0.055970

(Hartree/Particle)
HF(PBE1PBE)=-801.815171
Zero-point correction= 0.114068
Thermal correction to Energy= 0.123064
Thermal correction to Enthalpy= 0.124008
Thermal correction to Gibbs Free Energy= 0.078918
Sum of electronic and zero-point Energies= -801.701103
Sum of electronic and thermal Energies= -801.692107
Sum of electronic and thermal Enthalpies= -801.691163
Sum of electronic and thermal Free Energies= -801.736253
HF(MP2)=-800.106476
MP2=-800.8008929

[EtPPMe$_2$]$^+$

P	1.073559	0.044330	-0.008373
P	-0.630754	-0.981184	-0.244947
C	1.345621	1.830808	-0.030510
C	-1.920812	0.328151	-0.480814
H	1.753564	2.140464	0.935771
H	0.412562	2.361788	-0.218539
H	2.064404	2.069788	-0.819404
C	2.587689	-0.891118	0.280865
H	3.018639	-0.601802	1.243039
H	3.307236	-0.681346	-0.515003
H	2.356461	-1.957742	0.290831
H	-2.350190	0.107396	-1.465557
H	-1.514585	1.339648	-0.523770
C	-2.991111	0.193541	0.605186
H	-3.416585	-0.812721	0.636910
H	-3.805729	0.889492	0.391443
H	-2.596167	0.429549	1.595731

(Hartree/Particle)
HF(PBE1PBE)=-841.0914093
Zero-point correction= 0.143215
Thermal correction to Energy= 0.153354
Thermal correction to Enthalpy= 0.154298
Thermal correction to Gibbs Free Energy= 0.106587
Sum of electronic and zero-point Energies= -840.948195
Sum of electronic and thermal Energies= -840.938055
Sum of electronic and thermal Enthalpies= -840.937111
Sum of electronic and thermal Free Energies= -840.984823
HF(MP2)=-839.1517404
MP2=-839.9993512

[^{i}PrPPMe$_2$]$^+$

P	-1.375170	0.043592	0.000000
P	0.341197	-0.986939	0.000000
C	-1.635061	1.833058	0.000000
C	-2.924222	-0.881681	0.000000
C	1.674263	0.321180	0.000000
H	-0.681463	2.360745	0.000001
H	-2.204415	2.109888	0.891706
H	-2.204413	2.109888	-0.891708
H	-2.705114	-1.950819	0.000000
H	-3.503618	-0.627755	-0.891585
H	-3.503618	-0.627755	0.891586
C	2.501153	0.115196	1.271680
H	1.244592	1.325630	0.000000
C	2.501153	0.115195	-1.271680
H	2.924687	-0.892769	-1.318898
H	3.336772	0.821528	-1.266700
H	1.920504	0.287587	-2.180917
H	1.920504	0.287588	2.180917
H	3.336772	0.821529	1.266700
H	2.924687	-0.892768	1.318899

(Hartree/Particle)
HF(PBE1PBE)=-880.3689129
Zero-point correction= 0.171313
Thermal correction to Energy= 0.182860
Thermal correction to Enthalpy= 0.183804
Thermal correction to Gibbs Free Energy= 0.133028
Sum of electronic and zero-point Energies= -880.197600
Sum of electronic and thermal Energies= -880.186053
Sum of electronic and thermal Enthalpies= -880.185109
Sum of electronic and thermal Free Energies= -880.235885
HF(MP2)=-878.1971146
MP2=-879.2005775

[^{t}BuPPMe$_2$]$^+$

P	-1.530104	-0.023642	0.000000
P	0.149205	-1.118450	0.000000
C	-1.817538	1.763322	0.000000
C	1.626333	0.060757	0.000000
C	-3.083377	-0.944536	0.000000
H	-0.874897	2.307663	-0.000004
H	-2.394081	2.027833	0.891295
H	-2.394088	2.027832	-0.891292
H	-2.869326	-2.014492	0.000001
H	-3.661834	-0.687389	-0.891382
H	-3.661834	-0.687388	0.891382
C	1.651866	0.912275	-1.270575
C	2.818191	-0.910520	0.000002
C	1.651865	0.912277	1.270574
H	0.843744	1.646482	1.304828
H	2.598505	1.462006	1.302061
H	1.598488	0.302163	2.176393
H	1.598491	0.302159	-2.176394
H	2.598506	1.462004	-1.302062
H	0.843746	1.646479	-1.304832
H	2.830334	-1.549694	-0.887591
H	2.830333	-1.549693	0.887596
H	3.743363	-0.326034	0.000002

(Hartree/Particle)
HF(PBE1PBE)=-919.6422868
Zero-point correction= 0.199217
Thermal correction to Energy= 0.211955
Thermal correction to Enthalpy= 0.212899
Thermal correction to Gibbs Free Energy= 0.160465
Sum of electronic and zero-point Energies= -919.443070
Sum of electronic and thermal Energies= -919.430332
Sum of electronic and thermal Enthalpies= -919.429387

Sum of electronic and thermal Free Energies= -919.481821
HF(MP2)=-917.2366756
MP2=-918.4000081

[PhPPMe$_2$]$^+$

P	-2.038268	0.115647	0.144085
P	-0.696186	-1.300587	-0.366458
C	-3.783394	-0.260203	-0.124012
C	-1.735962	1.811747	0.690787
H	-3.883387	-1.304987	-0.423928
H	-4.186987	0.384393	-0.909355
H	-4.339318	-0.093477	0.802086
H	-0.704360	1.900939	1.034949
H	-2.420956	2.030474	1.514703
H	-1.916774	2.516362	-0.125182
H	2.709568	2.277688	-1.000088
C	2.470721	1.309561	-0.573672
C	1.189185	0.798746	-0.701121
H	0.441536	1.358904	-1.253237
C	0.886827	-0.463826	-0.167007
C	1.893061	-1.217945	0.457445
H	1.678531	-2.210788	0.841564
C	3.166251	-0.686296	0.596627
H	3.938024	-1.263218	1.093741
C	3.454172	0.574564	0.084373
H	4.455767	0.979720	0.179824

(Hartree/Particle)
HF(PBE1PBE)=-993.3753974
Zero-point correction= 0.168787
Thermal correction to Energy= 0.180303
Thermal correction to Enthalpy= 0.181247
Thermal correction to Gibbs Free Energy= 0.130445
Sum of electronic and zero-point Energies= -993.206610
Sum of electronic and thermal Energies= -993.195095
Sum of electronic and thermal Enthalpies= -993.194151
Sum of electronic and thermal Free Energies= -993.244953
HF(MP2)=-990.6602909
MP2=-992.0257679

[^{i}Pr$_2$NPPMe$_2$]$^+$

P	2.280988	0.072381	0.508752
N	-0.767363	0.262431	-0.047964
C	2.663680	-1.500359	-0.376829
C	3.598840	1.222283	-0.068996
C	-0.768277	-1.163425	0.374969
C	-2.027594	1.038220	-0.337097
C	-0.773999	-2.096508	-0.835583
C	-2.757188	1.468135	0.930907
C	-2.926109	0.348014	-1.350812
H	-1.654197	1.955057	-0.808892
H	-0.566988	-3.115828	-0.498636
H	-0.013446	-1.816256	-1.567899
H	-1.744121	-2.104760	-1.333434
H	-3.312402	0.657860	1.401332
H	-3.476780	2.244084	0.656536
H	-2.067971	1.898666	1.661368
H	-3.401649	-0.548985	-0.950274
H	-2.385018	0.089095	-2.263411
H	-3.724538	1.042889	-1.622695
P	0.582689	1.154356	-0.268513
H	1.957174	-2.286567	-0.115447
H	3.652918	-1.804280	-0.023722
H	2.699723	-1.367921	-1.459367
H	4.519195	0.952795	0.454889
H	3.353623	2.248952	0.212244
H	3.772932	1.165807	-1.145102
C	-1.850409	-1.522555	1.381605
H	-1.819507	-0.879631	2.262273
H	-1.662034	-2.547011	1.712848
H	-2.853113	-1.501806	0.952610
H	0.188922	-1.283064	0.897976

(Hartree/Particle)
HF(PBE1PBE)=-1053.5138702
Zero-point correction= 0.274761
Thermal correction to Energy= 0.290806
Thermal correction to Enthalpy= 0.291750
Thermal correction to Gibbs Free Energy= 0.232524
Sum of electronic and zero-point Energies= -1053.239109
Sum of electronic and thermal Energies= -1053.223064
Sum of electronic and thermal Enthalpies= -1053.222120
Sum of electronic and thermal Free Energies= -1053.281346
HF(MP2)=-1050.3813726
MP2=-1052.036719

[R$_2$P]$^+$

[Me$_2$P]$^+$

P	0.000000	0.637061	0.000000
C	1.394573	-0.462072	0.023500
C	-1.394573	-0.462072	-0.023500
H	2.271370	-0.003690	0.489253
H	1.643350	-0.518245	-1.062845
H	1.208644	-1.483591	0.362984
H	-2.271370	-0.003690	-0.489254
H	-1.643350	-0.518245	1.062845
H	-1.208644	-1.483591	-0.362984

(Hartree/Particle)
HF(PBE1PBE)=-420.643882
Zero-point correction= 0.071792
Thermal correction to Energy= 0.077031
Thermal correction to Enthalpy= 0.077976
Thermal correction to Gibbs Free Energy= 0.044537
Sum of electronic and zero-point Energies= -420.572090
Sum of electronic and thermal Energies= -420.566851
Sum of electronic and thermal Enthalpies= -420.565906
Sum of electronic and thermal Free Energies= -420.599345
HF(MP2)=-419.6904109
MP2=-420.0971615

[Et$_2$P]$^+$

P	0.237976	-0.594469	-0.549346
C	1.689471	-0.362266	0.501904
C	-1.140253	-0.232428	0.590687
H	2.582671	-0.877585	0.161360
H	1.531155	-0.437972	1.574100
C	1.522192	1.045963	-0.058225
C	-2.348883	0.413383	-0.080522
H	-0.800378	0.312123	1.477263
H	-1.395750	-1.248483	0.941657
H	0.660773	1.089417	-0.880588
H	2.343988	1.372224	-0.696298
H	1.171729	1.781057	0.664428
H	-2.135071	1.437331	-0.397069
H	-3.180263	0.453433	0.625622
H	-2.683653	-0.152422	-0.953359

(Hartree/Particle)
HF(PBE1PBE)=-499.2121044
Zero-point correction= 0.131043
Thermal correction to Energy= 0.138043

Thermal correction to Enthalpy= 0.138987
Thermal correction to Gibbs Free Energy= 0.100380
Sum of electronic and zero-point Energies= -499.081062
Sum of electronic and thermal Energies= -499.074061
Sum of electronic and thermal Enthalpies= -499.073117
Sum of electronic and thermal Free Energies= -499.111724
HF(MP2)=-497.7837522
MP2=-498.5067244

[^{i}Pr$_2$P]$^+$

P	0.000001	0.000003	-0.681624
C	-1.423510	-0.052960	0.431103
C	1.423508	0.052956	0.431110
C	-1.477276	1.491464	0.257560
H	-1.164356	-0.313441	1.457505
C	-2.651993	-0.791480	-0.069464
C	2.651992	0.791481	-0.069447
C	1.477277	-1.491467	0.257556
H	1.164349	0.313429	1.457513
H	-2.869565	-0.556999	-1.115349
H	-3.522543	-0.507794	0.527639
H	-2.523912	-1.872391	0.018428
H	2.869568	0.557008	-1.115332
H	3.522539	0.507791	0.527659
H	2.523910	1.872391	0.018453
H	-1.179932	2.019906	1.162314
H	-2.454533	1.809675	-0.109837
H	-0.782496	1.890074	-0.543801
H	0.782506	-1.890072	-0.543814
H	1.179926	-2.019916	1.162304
H	2.454538	-1.809673	-0.109833

(Hartree/Particle)
HF(PBE1PBE)=-577.7680955
Zero-point correction= 0.186709
Thermal correction to Energy= 0.197090
Thermal correction to Enthalpy= 0.198035
Thermal correction to Gibbs Free Energy= 0.151462
Sum of electronic and zero-point Energies= -577.581387
Sum of electronic and thermal Energies= -577.571005
Sum of electronic and thermal Enthalpies= -577.570061
Sum of electronic and thermal Free Energies= -577.616633
HF(MP2)=-117.4116025
MP2=-576.9061859

[^{t}Bu$_2$P]$^+$

P	-0.065396	-0.874773	-0.348426
C	-1.587586	0.086585	0.018198
C	1.504217	0.066980	-0.030367
C	2.595719	-1.015318	-0.044872
C	1.649276	0.929986	1.216467
C	1.577682	0.896008	-1.330125
C	-2.785737	-0.555526	-0.675836
C	-1.655043	1.601346	-0.004430
C	-1.287636	-0.547976	1.381166
H	1.376083	0.300168	-2.231805
H	2.600826	1.269528	-1.442331
H	0.898436	1.750359	-1.317648
H	2.550280	-1.652476	0.843057
H	3.575732	-0.528702	-0.055140
H	2.537955	-1.656858	-0.929500
H	1.598608	0.332662	2.131191
H	0.921709	1.739250	1.280052
H	2.640468	1.394350	1.196061
H	-0.430810	-1.371345	1.305922
H	-2.070292	-1.218419	1.742544
H	-0.895678	0.145792	2.121777
H	-2.859890	-0.211322	-1.709575
H	-3.704707	-0.267150	-0.155949
H	-2.729714	-1.647841	-0.686007
H	-1.684091	1.962208	-1.034739
H	-0.826515	2.085663	0.509098
H	-2.582814	1.923236	0.478178

Hartree/Particle)
HF(PBE1PBE)=-656.3168322
Zero-point correction= 0.242532
Thermal correction to Energy= 0.255270
Thermal correction to Enthalpy= 0.256215
Thermal correction to Gibbs Free Energy= 0.205626
Sum of electronic and zero-point Energies= -656.074300
Sum of electronic and thermal Energies= -656.061562
Sum of electronic and thermal Enthalpies= -656.060618
Sum of electronic and thermal Free Energies= -656.111207
HF(MP2)=-653.9557722
MP2=-655.3057771

[Ph$_2$P]$^+$

P	0.000000	0.000000	-1.449301
H	-0.245700	4.767002	-0.917572
C	0.000000	3.837285	-0.417478
C	-0.109246	2.635873	-1.089960
H	-0.433378	2.620578	-2.126912
C	0.178476	1.415311	-0.426036
C	0.664135	1.449900	0.904526
H	0.976060	0.535565	1.394986
C	0.803112	2.658972	1.551722
H	1.197267	2.692632	2.561235
C	0.454800	3.846940	0.900097
H	0.565950	4.792645	1.420793
H	-0.976060	-0.535565	1.394986
C	-0.664135	-1.449900	0.904526
C	-0.178476	-1.415311	-0.426036
C	0.109246	-2.635873	-1.089960
H	0.433378	-2.620578	-2.126912
C	0.000000	-3.837285	-0.417478
H	0.245700	-4.767002	-0.917572
C	-0.454800	-3.846940	0.900097
H	-0.565950	-4.792645	1.420793
C	-0.803112	-2.658972	1.551722
H	-1.197267	-2.692632	2.561235

(Hartree/Particle)
HF(PBE1PBE)=-803.7995183
Zero-point correction= 0.183403
Thermal correction to Energy= 0.194150
Thermal correction to Enthalpy= 0.195095
Thermal correction to Gibbs Free Energy= 0.146239
Sum of electronic and zero-point Energies= -803.616115
Sum of electronic and thermal Energies= -803.605368
Sum of electronic and thermal Enthalpies= -803.604424
Sum of electronic and thermal Free Energies= -803.653280
HF(MP2)=-800.8336139
MP2=-802.5756548

[(^{i}Pr$_2$N)$_2$P]$^+$

N	1.243353	0.225227	-0.034767
C	1.442327	-0.919027	0.897694
C	2.390232	1.002561	-0.620474
C	1.581725	-0.432477	2.340215
C	3.026616	0.299305	-1.814567
C	3.401813	1.476388	0.410431
H	1.908311	1.905921	-1.024949
H	1.547534	-1.298153	3.007305

H	0.762968	0.237068	2.615980
H	2.525617	0.083790	2.515420
H	3.642915	-0.551265	-1.523856
H	3.672881	1.011156	-2.334836
H	2.268854	-0.042364	-2.524317
H	3.988525	0.660161	0.835291
H	2.923886	2.029891	1.221350
H	4.103281	2.151739	-0.086083
H	-2.035781	2.934323	-0.066970
H	-2.260794	1.516553	2.045790
C	-2.720888	2.247262	-0.571767
H	-3.711193	-1.615716	0.506599
H	-3.724501	2.667563	-0.469758
H	-2.475803	2.225271	-1.636848
C	-3.025132	0.916686	1.541009
N	-1.501635	0.046319	-0.201626
H	0.233238	-1.744956	-1.332376
C	-3.068338	-1.940709	-0.312134
C	-2.746380	0.853335	0.044406
H	-3.995430	1.387452	1.719497
H	-3.036790	-3.032422	-0.279573
H	-3.042321	-0.073227	2.002453
C	-0.816581	-2.039834	-1.344817
H	-3.524615	-1.659441	-1.265047
C	-1.630352	-1.448201	-0.198390
H	-1.245960	-1.731419	-2.302172
H	-0.853933	-3.130296	-1.293402
H	-1.250708	-1.808974	0.764967
P	-0.163920	0.980887	-0.420128
H	-3.544291	0.305928	-0.455206
C	2.558597	-1.873024	0.498437
H	2.431864	-2.255275	-0.516182
H	2.531023	-2.727356	1.179507
H	3.549508	-1.425144	0.585223
H	0.516657	-1.488509	0.848601

(Hartree/Particle)
HF(PBE1PBE)=-924.0906746
Zero-point correction= 0.394826
Thermal correction to Energy= 0.414666
Thermal correction to Enthalpy= 0.415611
Thermal correction to Gibbs Free Energy= 0.348180
Sum of electronic and zero-point Energies= -923.695848
Sum of electronic and thermal Energies= -923.676008
Sum of electronic and thermal Enthalpies= -923.675064
Sum of electronic and thermal Free Energies= -923.742494
HF(MP2)=-920.2788043
MP2=-922.6168412

$[R_2P]^{\cdot}$

$[Me_2P]^{\cdot}$

P	0.000000	0.704608	0.000000
C	0.000000	-0.503314	1.398129
C	0.000000	-0.503314	-1.398129
H	0.000000	0.030402	2.350936
H	0.885665	-1.147540	1.362701
H	-0.885665	-1.147540	1.362701
H	0.000000	0.030402	-2.350936
H	-0.885665	-1.147540	-1.362701
H	0.885665	-1.147540	-1.362701

(Hartree/Particle)
HF(PBE1PBE)=-420.9417969
Zero-point correction= 0.073652
Thermal correction to Energy= 0.078792
Thermal correction to Enthalpy= 0.079736
Thermal correction to Gibbs Free Energy= 0.045630
Sum of electronic and zero-point Energies= -420.868144
Sum of electronic and thermal Energies= -420.863005
Sum of electronic and thermal Enthalpies= -420.862061
Sum of electronic and thermal Free Energies= -420.896167
HF(MP2)=-419.974468
MP2=-420.3872287

$[Et_2P]^{\cdot}$

P	-0.056432	-0.899929	-0.215847
C	1.581335	-0.333416	0.455404
C	-1.114391	0.537717	0.293684
H	2.249017	-1.200252	0.417323
H	1.456938	-0.079064	1.515710
C	2.211045	0.837530	-0.299070
C	-2.576728	0.348185	-0.092149
H	-0.717010	1.452388	-0.163670
H	-1.014812	0.669033	1.379370
H	2.344834	0.600164	-1.358448
H	3.194838	1.081621	0.114272
H	1.595786	1.738996	-0.235052
H	-2.690351	0.237044	-1.174495
H	-3.181516	1.204914	0.219768
H	-2.998808	-0.546003	0.375717

(Hartree/Particle)
HF(PBE1PBE)=-499.4873705
Zero-point correction= 0.131458
Thermal correction to Energy= 0.139189
Thermal correction to Enthalpy= 0.140133
Thermal correction to Gibbs Free Energy= 0.098164
Sum of electronic and zero-point Energies= -499.355912
Sum of electronic and thermal Energies= -499.348182
Sum of electronic and thermal Enthalpies= -499.347237
Sum of electronic and thermal Free Energies= -499.389207
HF(MP2)=-498.0575528
MP2=-498.7760041

$[^{i}Pr_2P]^{\cdot}$

P	0.000000	-0.000005	-0.907816
C	-1.430321	-0.130612	0.289834
C	1.430321	0.130613	0.289832
C	-1.805551	1.242694	0.850108
H	-1.104142	-0.770164	1.120670
C	-2.622756	-0.788121	-0.400147
C	2.622753	0.788121	-0.400153
C	1.805554	-1.242688	0.850114
H	1.104141	0.770170	1.120665
H	-2.950530	-0.197999	-1.262904
H	-3.471939	-0.869380	0.287412
H	-2.380519	-1.792266	-0.758915
H	2.950528	0.197995	-1.262907
H	3.471937	0.869386	0.287405
H	2.380514	1.792264	-0.758927
H	-0.970169	1.721975	1.368453
H	-2.630991	1.150882	1.565653
H	-2.132079	1.914423	0.049490
H	2.132084	-1.914421	0.049500
H	0.970174	-1.721968	1.368462
H	2.630995	-1.150869	1.565658

(Hartree/Particle)
HF(PBE1PBE)=-578.0358573
Zero-point correction= 0.187846
Thermal correction to Energy= 0.198203

Thermal correction to Enthalpy= 0.199147
Thermal correction to Gibbs Free Energy= 0.151623
Sum of electronic and zero-point Energies= -577.848011
Sum of electronic and thermal Energies= -577.837655
Sum of electronic and thermal Enthalpies= -577.836711
Sum of electronic and thermal Free Energies= -577.884234
HF(MP2)=-576.1415452
MP2=-577.1714482

[^{t}Bu$_2$P]$^·$

P	0.000000	1.053028	0.000000
C	-1.552886	-0.029892	-0.001824
C	1.552886	-0.029892	0.001824
C	2.670012	0.942442	0.406197
C	1.847963	-0.546461	-1.411209
C	1.526941	-1.189533	0.996908
C	-2.670012	0.942442	-0.406196
C	-1.847963	-0.546463	1.411208
C	-1.526941	-1.189532	-0.996909
H	1.308946	-0.845971	2.012687
H	2.511039	-1.675456	1.016925
H	0.792872	-1.952735	0.728666
H	2.720784	1.800547	-0.271516
H	3.639205	0.429780	0.374733
H	2.522064	1.326295	1.420220
H	1.881200	0.272413	-2.136560
H	1.105461	-1.270304	-1.752573
H	2.827794	-1.041616	-1.423873
H	-1.308946	-0.845969	-2.012688
H	-2.511039	-1.675455	-1.016926
H	-0.792872	-1.952734	-0.728668
H	-2.720784	1.800546	0.271518
H	-3.639205	0.429780	-0.374732
H	-2.522065	1.326296	-1.420219
H	-1.881200	0.272411	2.136560
H	-1.105461	-1.270306	1.752572
H	-2.827794	-1.041617	1.423872

(Hartree/Particle)
HF(PBE1PBE)=-656.5783408
Zero-point correction= 0.243705
Thermal correction to Energy= 0.256494
Thermal correction to Enthalpy= 0.257438
Thermal correction to Gibbs Free Energy= 0.206046
Sum of electronic and zero-point Energies= -656.334636
Sum of electronic and thermal Energies= -656.321847
Sum of electronic and thermal Enthalpies= -656.320903
Sum of electronic and thermal Free Energies= -656.372295
HF=-654.2164337
MP2=-655.5649395

[Ph$_2$P]$^·$

P	0.000001	1.609910	0.000003
H	4.702207	0.615869	0.912841
C	3.790905	0.228340	0.468697
C	2.624758	0.978522	0.521868
H	2.632056	1.952066	1.004399
C	1.428186	0.494436	-0.032014
C	1.449907	-0.757253	-0.668748
H	0.546889	-1.140713	-1.131911
C	2.619572	-1.501145	-0.727204
H	2.619070	-2.463966	-1.228609
C	3.792025	-1.015567	-0.154443
H	4.704147	-1.601604	-0.200437
H	-0.546894	-1.140708	1.131921
C	-1.449910	-0.757250	0.668754
C	-1.428186	0.494437	0.032015
C	-2.624756	0.978521	-0.521873
H	-2.632052	1.952063	-1.004407
C	-3.790902	0.228339	-0.468704
H	-4.702202	0.615866	-0.912854
C	-3.792026	-1.015566	0.154440
H	-4.704148	-1.601603	0.200432
C	-2.619575	-1.501142	0.727208
H	-2.619075	-2.463961	1.228616

(Hartree/Particle)
HF(PBE1PBE)=-804.0527149
Zero-point correction= 0.182258
Thermal correction to Energy= 0.193058
Thermal correction to Enthalpy= 0.194002
Thermal correction to Gibbs Free Energy= 0.143278
Sum of electronic and zero-point Energies= -803.870456
Sum of electronic and thermal Energies= -803.859657
Sum of electronic and thermal Enthalpies= -803.858713
Sum of electronic and thermal Free Energies= -803.909437
HF(MP2)=-801.0880999
MP2=-802.7537623

[(^{i}Pr$_2$N)$_2$P]$^·$

N	1.257974	0.009158	-0.250857
C	1.269307	0.756092	1.005815
C	2.458793	-0.495807	-0.928131
C	1.685816	2.223790	0.853788
C	2.960789	-1.825126	-0.358873
C	3.590454	0.517039	-1.067217
H	2.116396	-0.717159	-1.950771
H	1.406569	2.776917	1.757187
H	1.179404	2.686766	0.004418
H	2.762935	2.342184	0.717645
H	3.415531	-1.707948	0.627006
H	3.716566	-2.252967	-1.025914
H	2.137010	-2.538591	-0.277697
H	4.063123	0.740629	-0.106930
H	3.236420	1.451959	-1.507092
H	4.364245	0.103669	-1.721261
H	-1.315525	2.730975	0.243178
H	-3.198642	1.450889	1.455247
C	-1.686443	2.357100	-0.716389
H	-3.629125	-1.459354	-1.003844
H	-2.369938	3.100482	-1.137227
H	-0.839369	2.254843	-1.399596
C	-3.570276	1.243353	0.445970
N	-1.464207	-0.013699	-0.062958
H	-0.556401	-1.478939	2.035680
C	-2.874093	-2.055184	-0.482312
C	-2.412017	1.030999	-0.525195
H	-4.151415	2.109653	0.117745
H	-3.387576	-2.884282	0.015114
H	-4.255717	0.394459	0.494742
C	-1.075493	-2.067141	1.275312
H	-2.196885	-2.475465	-1.233159
C	-2.093928	-1.216333	0.530370
H	-0.329202	-2.486960	0.594364
H	-1.583984	-2.898851	1.772219
H	-2.797121	-0.850076	1.284498
P	-0.159448	-0.330369	-1.171917
H	-2.845974	0.744245	-1.498951
C	2.062523	0.084624	2.124661
H	1.759606	-0.955659	2.263271
H	1.885232	0.619445	3.063255
H	3.140333	0.107905	1.938646
H	0.216270	0.758101	1.306892

(Hartree/Particle)

HF(PBE1PBE)=-924.2862201
Zero-point correction= 0.391833
Thermal correction to Energy= 0.412122
Thermal correction to Enthalpy= 0.413066
Thermal correction to Gibbs Free Energy= 0.343935
Sum of electronic and zero-point Energies= -923.894387
Sum of electronic and thermal Energies= -923.874098
Sum of electronic and thermal Enthalpies= -923.873154
Sum of electronic and thermal Free Energies= -923.942285
HF(MP2)= -920.4630989
MP2=-922.7977383

$[R]^+$

$[Me]^+$

C	0.000000	0.000000	0.000000
H	-0.947843	0.547237	0.000000
H	0.947843	0.547237	0.000000
H	0.000000	-1.094474	0.000000

(Hartree/Particle)
HF(PBE1PBE)=-39.4302748
Zero-point correction= 0.031274
Thermal correction to Energy= 0.034128
Thermal correction to Enthalpy= 0.035072
Thermal correction to Gibbs Free Energy= 0.012182
Sum of electronic and zero-point Energies= -39.399001
Sum of electronic and thermal Energies= -39.396147
Sum of electronic and thermal Enthalpies= -39.395202
Sum of electronic and thermal Free Energies= -39.418092
HF(MP2)=-39.2435024
MP2=-39.3565107

$[Et]^+$

C	-0.064284	0.688736	0.000000
C	-0.064284	-0.688737	0.000000
H	-0.071081	1.244703	0.936248
H	-0.071081	1.244703	-0.936248
H	-0.071082	-1.244700	0.936249
H	1.055737	0.000000	0.000000
H	-0.071082	-1.244700	-0.936249

(Hartree/Particle)
HF(PBE1PBE)=-78.7757686
Zero-point correction= 0.060879
Thermal correction to Energy= 0.064027
Thermal correction to Enthalpy= 0.064971
Thermal correction to Gibbs Free Energy= 0.038385
Sum of electronic and zero-point Energies= -78.714890
Sum of electronic and thermal Energies= -78.711742
Sum of electronic and thermal Enthalpies= -78.710798
Sum of electronic and thermal Free Energies= -78.737384
HF(MP2)=-78.3348612
MP2=-78.6141373

$[^iPr]^+$

C	0.000000	0.439893	-0.000001
C	1.279386	-0.201779	-0.000002
C	-1.279386	-0.201779	0.000001
H	0.000000	1.534097	0.000000
H	1.266867	-1.289167	-0.000014
H	1.860891	0.206544	0.849685
H	1.860919	0.206569	-0.849655
H	-1.266867	-1.289167	0.000002
H	-1.860903	0.206555	-0.849672
H	-1.860907	0.206558	0.849668

(Hartree/Particle)
HF(PBE1PBE)=-118.0841763
Zero-point correction= 0.087782
Thermal correction to Energy= 0.092125
Thermal correction to Enthalpy= 0.093069
Thermal correction to Gibbs Free Energy= 0.061908
Sum of electronic and zero-point Energies= -117.996394
Sum of electronic and thermal Energies= -117.992051
Sum of electronic and thermal Enthalpies= -117.991107
Sum of electronic and thermal Free Energies= -118.022269
HF(MP2)=-117.4116025
MP2=-117.8343049

$[^tBu]^+$

C	0.002973	-0.002046	-0.011298
C	1.373947	-0.488152	-0.014400
C	-1.110788	-0.940156	-0.012293
C	-0.259459	1.427567	0.017553
H	-0.131982	1.737525	-1.040390
H	0.506435	1.987167	0.562266
H	-1.273300	1.695182	0.315172
H	1.500781	-1.370487	-0.648838
H	1.537925	-0.862866	1.016219
H	2.121925	0.280163	-0.208199
H	-0.840588	-1.967158	-0.251183
H	-1.935940	-0.575153	-0.635612
H	-1.525293	-0.907644	1.013192

(Hartree/Particle)
HF(PBE1PBE)=-157.3878878
Zero-point correction= 0.116506
Thermal correction to Energy= 0.122796
Thermal correction to Enthalpy= 0.123740
Thermal correction to Gibbs Free Energy= 0.087382
Sum of electronic and zero-point Energies= -157.271382
Sum of electronic and thermal Energies= -157.265092
Sum of electronic and thermal Enthalpies= -157.264147
Sum of electronic and thermal Free Energies= -157.300506
HF(MP2)=-156.4836504
MP2=-157.0585758

$[Ph]^+$

H	-2.183856	1.396703	-0.000002
C	-1.268962	0.817754	-0.000001
C	0.000000	1.177631	0.000000
C	1.268962	0.817754	0.000002
H	2.183856	1.396703	0.000003
C	1.208525	-0.609134	-0.000001
H	2.169248	-1.118359	-0.000001
C	0.000000	-1.291718	-0.000001
H	0.000000	-2.375611	-0.000001
C	-1.208525	-0.609134	0.000000
H	-2.169248	-1.118359	0.000000

(Hartree/Particle)
HF(PBE1PBE)=-231.0274415
Zero-point correction= 0.085164
Thermal correction to Energy= 0.090010
Thermal correction to Enthalpy= 0.090954
Thermal correction to Gibbs Free Energy= 0.057570
Sum of electronic and zero-point Energies= -230.942278
Sum of electronic and thermal Energies= -230.937431
Sum of electronic and thermal Enthalpies= -230.936487
Sum of electronic and thermal Free Energies= -230.969872

HF(MP2)=-229.8048051
MP2=-230.5974685

[^{i}Pr$_2$N]$^+$

N	-0.000001	0.891274	-0.000004
C	1.128195	0.287243	0.509814
C	-1.128195	0.287241	-0.509817
C	1.952523	0.622588	-0.847340
C	-1.952519	0.622596	0.847338
C	-1.250245	-1.162370	-0.925968
H	-1.578763	0.974290	-1.234429
H	2.942303	0.240869	-0.576416
H	2.003710	1.689217	-1.050355
H	1.567166	0.067524	-1.701187
H	-1.567159	0.067539	1.701189
H	-2.942300	0.240873	0.576419
H	-2.003707	1.689226	1.050345
H	-1.114051	-1.864046	-0.103618
H	-0.538360	-1.400094	-1.720424
H	-2.252336	-1.312049	-1.334446
C	1.250243	-1.162364	0.925975
H	0.538356	-1.400082	1.720432
H	2.252332	-1.312041	1.334456
H	1.114050	-1.864046	0.103630
H	1.578760	0.974298	1.234423

(Hartree/Particle)
HF(PBE1PBE)=-291.1824542
Zero-point correction= 0.191303
Thermal correction to Energy= 0.200262
Thermal correction to Enthalpy= 0.201206
Thermal correction to Gibbs Free Energy= 0.158842
Sum of electronic and zero-point Energies= -290.991152
Sum of electronic and thermal Energies= -290.982192
Sum of electronic and thermal Enthalpies= -290.981248
Sum of electronic and thermal Free Energies= -291.023612
HF(MP2)=-289.5368867
MP2=-290.6143168

[R]$^\cdot$

[Me]$^\cdot$

C	0.000000	0.000000	0.000000
H	0.000000	1.081466	0.000000
H	0.936577	-0.540733	0.000000
H	-0.936577	-0.540733	0.000000

(Hartree/Particle)
HF(PBE1PBE)=-39.5736882
Zero-point correction= 0.029751
Thermal correction to Energy= 0.032806
Thermal correction to Enthalpy= 0.033750
Thermal correction to Gibbs Free Energy= 0.010999
Sum of electronic and zero-point Energies= -39.765776
Sum of electronic and thermal Energies= -39.762721
Sum of electronic and thermal Enthalpies= -39.761777
Sum of electronic and thermal Free Energies= -39.784528
HF(MP2)=-39.5735545
MP2=-39.708653

[Et]$^\cdot$

C	-0.790831	0.000000	-0.019281
C	0.691017	0.000000	-0.001641
H	-1.349265	-0.926984	0.042069
H	-1.349265	0.926984	0.042069
H	1.104618	-0.886321	-0.492589
H	1.088176	0.000000	1.026567
H	1.104618	0.886321	-0.492589

(Hartree/Particle)
HF(PBE1PBE)=-79.0751718
Zero-point correction= 0.059348
Thermal correction to Energy= 0.063317
Thermal correction to Enthalpy= 0.064261
Thermal correction to Gibbs Free Energy= 0.035158
Sum of electronic and zero-point Energies= -79.015824
Sum of electronic and thermal Energies= -79.011855
Sum of electronic and thermal Enthalpies= -79.010911
Sum of electronic and thermal Free Energies= -79.040014
HF(MP2)=-78.6200681
MP2=-78.9046758

[^{i}Pr]$^\cdot$

C	0.000000	0.534368	-0.044888
C	1.290503	-0.197660	0.002828
C	-1.290503	-0.197660	0.002828
H	0.000000	1.610066	0.101025
H	1.301882	-1.037978	-0.703298
H	1.478725	-0.634551	0.998254
H	2.139880	0.450356	-0.227774
H	-1.301881	-1.037978	-0.703298
H	-2.139880	0.450356	-0.227774
H	-1.478725	-0.634551	0.998254

(Hartree/Particle)
HF(PBE1PBE)=-118.3557837
Zero-point correction= 0.088009
Thermal correction to Energy= 0.093204
Thermal correction to Enthalpy= 0.094148
Thermal correction to Gibbs Free Energy= 0.060758
Sum of electronic and zero-point Energies= -118.267774
Sum of electronic and thermal Energies= -118.262580
Sum of electronic and thermal Enthalpies= -118.261636
Sum of electronic and thermal Free Energies= -118.295026
HF(MP2)=-117.6676797
MP2=-118.1035428

[^{t}Bu]$^\cdot$

C	0.000000	0.000002	-0.158814
C	0.909984	1.165252	0.015754
C	0.554147	-1.370694	0.015753
C	-1.464130	0.205442	0.015754
H	-1.797757	1.148264	-0.430089
H	-1.746950	0.245134	1.083268
H	-2.044351	-0.609159	-0.430075
H	1.893308	0.982763	-0.430077
H	1.085754	1.390343	1.083269
H	0.494637	2.075037	-0.430084
H	1.549699	-1.465898	-0.430125
H	-0.095580	-2.131037	-0.430037
H	0.661235	-1.635459	1.083266

Convergence failure.

Item	Value	Threshold	Converged?
Maximum Force	0.000006	0.000002	NO
RMS Force	0.000001	0.000001	NO
Maximum Displacement	0.000023	0.000006	NO
RMS Displacement	0.000008	0.000004	NO

Predicted change in Energy=-1.204910D-10

(Hartree/Particle)
HF(PBE1PBE) = -157.636443099
Zero-point correction= 0.116567
Thermal correction to Energy= 0.122917
Thermal correction to Enthalpy= 0.123861
Thermal correction to Gibbs Free Energy= 0.087195
Sum of electronic and zero-point Energies= -157.519877
Sum of electronic and thermal Energies= -157.513526
Sum of electronic and thermal Enthalpies= -157.512582
Sum of electronic and thermal Free Energies= -157.549248
HF(MP2)=-156.7155558
MP2=-157.304689

[Ph]·

H	-2.156008	1.321412	0.000000
C	-1.222074	0.768746	0.000000
C	0.000000	1.391847	0.000000
C	1.222074	0.768746	0.000001
H	2.156008	1.321412	0.000001
C	1.209397	-0.629497	0.000000
H	2.148174	-1.175498	0.000000
C	0.000000	-1.318418	0.000000
H	0.000000	-2.403386	-0.000001
C	-1.209397	-0.629497	-0.000001
H	-2.148174	-1.175498	-0.000001

(Hartree/Particle)
HF(PBE1PBE)=-231.3347555
Zero-point correction= 0.087620
Thermal correction to Energy= 0.092000
Thermal correction to Enthalpy= 0.092945
Thermal correction to Gibbs Free Energy= 0.059581
Sum of electronic and zero-point Energies= -231.247136
Sum of electronic and thermal Energies= -231.242755
Sum of electronic and thermal Enthalpies= -231.241811
Sum of electronic and thermal Free Energies= -231.275174
HF(MP2)=-230.1147152
MP2=-230.8636887

[^{i}Pr$_2$N]·

N	0.000000	0.000003	-1.118263
C	1.213994	0.367733	-0.419145
C	-1.213994	-0.367733	-0.419145
C	2.028219	-0.885566	-0.061002
C	-2.028220	0.885565	-0.061001
C	-1.097027	-1.316993	0.776435
H	-1.804676	-0.897141	-1.178914
H	3.047053	-0.590129	0.206881
H	2.083697	-1.565666	-0.914624
H	1.597774	-1.424465	0.785480
H	-1.597776	1.424465	0.785481
H	-3.047053	0.590125	0.206883
H	-2.083700	1.565664	-0.914622
H	-0.592134	-0.864690	1.633178
H	-0.565795	-2.232294	0.504154
H	-2.102897	-1.603463	1.100025
C	1.097029	1.316993	0.776436
H	0.565797	2.232294	0.504156
H	2.102899	1.603461	1.100027
H	0.592135	0.864688	1.633179
H	1.804677	0.897142	-1.178913

(Hartree/Particle)
HF(PBE1PBE)=-291.4731151
Zero-point correction= 0.192090
Thermal correction to Energy= 0.201493
Thermal correction to Enthalpy= 0.202437
Thermal correction to Gibbs Free Energy= 0.158176
Sum of electronic and zero-point Energies= -291.281025
Sum of electronic and thermal Energies= -291.271622
Sum of electronic and thermal Enthalpies= -291.270678
Sum of electronic and thermal Free Energies= -291.314939
HF(MP2)=-289.8327894
MP2=-290.9015982

Alkanes

Ethane, [CH_3CH_3]

H	-1.188611	0.004294	0.985326
C	-0.760209	0.022732	-0.021103
H	-1.170478	-0.831301	-0.567980
H	-1.119283	0.931017	-0.513902
C	0.760209	-0.022732	0.021103
H	1.170478	0.831301	0.567980
H	1.119283	-0.931017	0.513902
H	1.188611	-0.004294	-0.985326

(Hartree/Particle)
HF(PBE1PBE)=-79.7432431
Zero-point correction= 0.074717
Thermal correction to Energy= 0.078198
Thermal correction to Enthalpy= 0.079142
Thermal correction to Gibbs Free Energy= 0.051613
Sum of electronic and zero-point Energies= -79.668526
Sum of electronic and thermal Energies= -79.665045
Sum of electronic and thermal Enthalpies= -79.664101
Sum of electronic and thermal Free Energies= -79.691630
HF(MP2)=-79.2516914
MP2=-79.5716326

Propane, [$CH_3CH_2CH_3$]

H	0.000000	2.167498	0.364597
C	0.000000	1.268333	-0.258420
H	-0.882779	1.311889	-0.905194
H	0.882779	1.311889	-0.905194
C	0.000000	0.000000	0.584077
H	0.875619	0.000000	1.244079
H	-0.875619	0.000000	1.244079
C	0.000000	-1.268333	-0.258420
H	-0.882779	-1.311889	-0.905194
H	0.882779	-1.311889	-0.905194
H	0.000000	-2.167498	0.364597

(Hartree/Particle)
HF(PBE1PBE)=-119.0180223
Zero-point correction= 0.103448
Thermal correction to Energy= 0.107977
Thermal correction to Enthalpy= 0.108921
Thermal correction to Gibbs Free Energy= 0.078455
Sum of electronic and zero-point Energies= -118.914574
Sum of electronic and thermal Energies= -118.910046
Sum of electronic and thermal Enthalpies= -118.909101
Sum of electronic and thermal Free Energies= -118.939568
HF(MP2)=-118.2958867
MP2=-118.7673629

Isobutane, [$(CH_3)_2CHCH_3$]

H	-1.385136	-1.677518	-0.262652

C	-0.389118	-1.398452	0.095355
H	-0.403314	-1.449523	1.190491
H	0.319614	-2.151861	-0.262664
C	0.000006	0.000001	-0.373300
C	1.405655	0.362244	0.095357
H	1.456992	0.375449	1.190492
H	2.145340	-0.360789	-0.262677
H	1.703750	1.352739	-0.262638
C	-1.016542	1.036210	0.095353
H	-2.023378	0.799136	-0.262667
H	-0.760214	2.038328	-0.262633
H	-1.053673	1.074024	1.190489
H	0.000006	0.000001	-1.472137

Convergence failure.

Item	Value	Threshold	Converged?
Maximum Force	0.000005	0.000002	NO
RMS Force	0.000002	0.000001	NO
Maximum Displacement	0.000096	0.000006	NO
RMS Displacement	0.000030	0.000004	NO

Predicted change in Energy=-3.056477D-10

(Hartree/Particle)
HF(PBE1PBE) = -158.294112471
Zero-point correction= 0.131490
Thermal correction to Energy= 0.137203
Thermal correction to Enthalpy= 0.138148
Thermal correction to Gibbs Free Energy= 0.103870
Sum of electronic and zero-point Energies= -158.162623
Sum of electronic and thermal Energies= -158.156909
Sum of electronic and thermal Enthalpies= -158.155965
Sum of electronic and thermal Free Energies= -158.190242
HF(MP2)=-157.3405944
MP2=-157.9662506

Neopentane, [$(CH_3)_4C$]

H	1.633788	0.617018	-1.302257
C	0.654977	0.131209	-1.376446
H	0.034556	0.729085	-2.052617
H	0.802582	-0.851133	-1.837748
C	-0.000019	0.000000	0.000015
C	-1.361847	-0.681839	-0.146131
H	-2.025328	-0.101343	-0.796057
H	-1.257265	-1.681543	-0.581143
H	-1.854939	-0.789295	0.825928
C	-0.188657	1.390534	0.609648
H	0.772161	1.903200	0.726195
H	-0.656738	1.327318	1.597818
H	-0.827085	2.015256	-0.024161
C	0.895529	-0.839909	0.912925
H	0.450618	-0.950833	1.907575
H	1.879435	-0.374740	1.035941
H	1.048319	-1.842959	0.500456

Convergence failure.

Item	Value	Threshold	Converged?
Maximum Force	0.000014	0.000002	NO
RMS Force	0.000003	0.000001	NO
Maximum Displacement	0.000065	0.000006	NO
RMS Displacement	0.000020	0.000004	NO

Predicted change in Energy=-1.450373D-09

(Hartree/Particle)
HF(PBE1PBE) = -197.570378985
Zero-point correction= 0.159194
Thermal correction to Energy= 0.166126
Thermal correction to Enthalpy= 0.167070
Thermal correction to Gibbs Free Energy= 0.130087
Sum of electronic and zero-point Energies= -197.411185
Sum of electronic and thermal Energies= -197.404253
Sum of electronic and thermal Enthalpies= -197.403309
Sum of electronic and thermal Free Energies= -197.440292
HF(MP2)=-157.3405944
MP2=-197.1675037

Toluene, [$C_6H_5CH_3$]

H	-0.739856	-2.137558	0.000000
C	-0.197708	-1.195543	0.000000
C	-0.911308	0.005571	0.000000
C	-0.191049	1.199204	0.000000
H	-0.727347	2.144125	0.000000
C	1.200465	1.197810	0.000000
H	1.739938	2.139791	0.000000
C	1.897004	-0.003761	0.000000
H	2.982090	-0.007962	0.000000
C	1.190884	-1.202860	0.000000
H	1.724992	-2.147937	0.000000
C	-2.413401	0.002447	0.000000
H	-2.813186	1.018890	-0.000003
H	-2.807977	-0.513272	0.881296
H	-2.807977	-0.513277	-0.881293

(Hartree/Particle)
HF(PBE1PBE)=-271.298102
Zero-point correction= 0.128039
Thermal correction to Energy= 0.133389
Thermal correction to Enthalpy= 0.134334
Thermal correction to Gibbs Free Energy= 0.099103
Sum of electronic and zero-point Energies= -271.170063
Sum of electronic and thermal Energies= -271.164712
Sum of electronic and thermal Enthalpies= -271.163768
Sum of electronic and thermal Free Energies= -271.198999
HF(MP2)=-269.8021703
MP2=-270.784128

Diisopropylmethylamine, [^{i}Pr$_2$NMe]

N	-0.025122	0.505916	-0.662079
C	-1.213804	-0.294710	-0.351144
C	1.259575	0.298073	0.017313
C	-1.956983	0.054815	0.948494
C	2.054028	-0.886347	-0.534325
C	1.225832	0.268580	1.549546
H	1.838842	1.187744	-0.258395
H	-2.936913	-0.433757	0.951470
H	-2.130020	1.130614	1.040857
H	-1.418561	-0.280130	1.837962
H	1.667592	-1.850066	-0.197635
H	3.094310	-0.818278	-0.199261
H	2.042754	-0.873767	-1.626816
H	0.777681	-0.655361	1.926741
H	0.664783	1.113686	1.958137
H	2.244834	0.321430	1.946595
C	-0.973297	-1.796173	-0.430651
H	-0.458792	-2.066265	-1.355465
H	-1.935164	-2.316964	-0.408084
H	-0.388013	-2.165343	0.416261
H	-1.906820	-0.053078	-1.169756
C	-0.342930	1.910272	-0.804988
H	0.460636	2.419942	-1.345774
H	-0.497406	2.449879	0.147051
H	-1.258416	2.021241	-1.394797

(Hartree/Particle)
HF(PBE1PBE)=-331.3938758
Zero-point correction= 0.233774
Thermal correction to Energy= 0.244303

Thermal correction to Enthalpy= 0.245247
Thermal correction to Gibbs Free Energy= 0.199410
Sum of electronic and zero-point Energies= -331.160102
Sum of electronic and thermal Energies= -331.149572
Sum of electronic and thermal Enthalpies= -331.148628
Sum of electronic and thermal Free Energies= -331.194466
HF(MP2)=-329.4799263
MP2=-330.7502653

Olefins

Ethene, [CH_2CH_2]

H	-0.924221	1.233491	0.000000
C	0.000000	0.663414	0.000000
H	0.924221	1.233491	0.000000
C	0.000000	-0.663414	0.000000
H	-0.924221	-1.233491	0.000000
H	0.924221	-1.233491	0.000000

(Hartree/Particle)
HF(PBE1PBE)=-78.504343
Zero-point correction= 0.051029
Thermal correction to Energy= 0.054072
Thermal correction to Enthalpy= 0.055016
Thermal correction to Gibbs Free Energy= 0.029505
Sum of electronic and zero-point Energies= -78.453314
Sum of electronic and thermal Energies= -78.450271
Sum of electronic and thermal Enthalpies= -78.449327
Sum of electronic and thermal Free Energies= -78.474838
HF(MP2)=-78.0557769
MP2=-78.3463571

Propene, [CH_2CHCH_3]

H	2.235445	0.289332	0.000000
C	1.277404	-0.219975	0.000000
H	1.296674	-1.306976	0.000000
C	0.131223	0.453415	0.000000
H	0.165057	1.542699	0.000000
C	-1.228393	-0.162716	0.000000
H	-1.173996	-1.254290	0.000000
H	-1.802294	0.152448	0.878684
H	-1.802294	0.152448	-0.878684

(Hartree/Particle)
HF(PBE1PBE)=-117.7848443
Zero-point correction= 0.079675
Thermal correction to Energy= 0.083764
Thermal correction to Enthalpy= 0.084709
Thermal correction to Gibbs Free Energy= 0.054671
Sum of electronic and zero-point Energies= -117.705170
Sum of electronic and thermal Energies= -117.701080
Sum of electronic and thermal Enthalpies= -117.700136
Sum of electronic and thermal Free Energies= -117.730173
HF(MP2)=-117.1043661
MP2=-117.5463019

Isobutene, [$CH_2C(CH_3)_2$]

H	0.000000	0.926194	2.021310
C	0.000000	0.000000	1.454572
H	0.000000	-0.926194	2.021310
C	0.000000	0.000000	0.121568
C	0.000000	-1.269424	-0.676048
H	0.000000	-2.152569	-0.033972
H	-0.878943	-1.318174	-1.329736
H	0.878943	-1.318174	-1.329737
C	0.000000	1.269424	-0.676048
H	0.000000	2.152569	-0.033972
H	-0.878943	1.318174	-1.329737
H	0.878943	1.318174	-1.329736

(Hartree/Particle)
HF(PBE1PBE)=-157.0650736
Zero-point correction= 0.107819
Thermal correction to Energy= 0.113142
Thermal correction to Enthalpy= 0.114086
Thermal correction to Gibbs Free Energy= 0.081163
Sum of electronic and zero-point Energies= -156.957254
Sum of electronic and thermal Energies= -156.951932
Sum of electronic and thermal Enthalpies= -156.950987
Sum of electronic and thermal Free Energies= -156.983910
HF(MP2)=-156.1526275
MP2=-156.747778

Isopropyliminopropane, [$^{i}PrNC(CH_3)$]

N	0.163790	-0.880071	0.236055
C	-1.257927	-0.551038	0.155654
C	1.109297	-0.062763	0.014953
C	-1.723628	-0.226348	-1.263534
C	2.515322	-0.584505	0.147725
C	1.042861	1.397106	-0.368493
H	-2.817158	-0.198808	-1.299502
H	-1.382993	-0.999066	-1.958166
H	-1.354605	0.736919	-1.624240
H	3.076145	-0.003476	0.888886
H	3.053934	-0.489316	-0.802189
H	2.496146	-1.631567	0.447859
H	0.035558	1.772406	-0.523813
H	1.620576	1.560502	-1.284662
H	1.521069	1.999801	0.411833
C	-1.708432	0.478006	1.192381
H	-1.350495	0.194181	2.185946
H	-2.801719	0.516317	1.226886
H	-1.347140	1.486908	0.979372
H	-1.760801	-1.487054	0.427288

(Hartree/Particle)
HF(PBE1PBE)=-290.9209976
Zero-point correction= 0.181016
Thermal correction to Energy= 0.190466
Thermal correction to Enthalpy= 0.191410
Thermal correction to Gibbs Free Energy= 0.146288
Sum of electronic and zero-point Energies= -290.739981
Sum of electronic and thermal Energies= -290.730532
Sum of electronic and thermal Enthalpies= -290.729588
Sum of electronic and thermal Free Energies= -290.774710
HF(MP2)=-289.2804573
MP2= -290.3661796

[PMe_3] Derivatives

[PMe_3]

P	-0.000005	-0.000009	-0.599989
C	1.087090	-1.210134	0.277827
C	-1.591584	-0.336350	0.277825
H	2.127338	-1.048637	-0.017303
H	0.815430	-2.227197	-0.017268
H	1.011339	-1.125775	1.367566
H	-2.336535	0.407453	-0.017307
H	-1.480652	-0.312862	1.367565
H	-1.971863	-1.317990	-0.017243

C	0.504494	1.546497	0.277819
H	1.521149	1.819691	-0.017269
H	0.469310	1.438693	1.367554
H	-0.155437	2.366676	-0.017296

Convergence failure.

Item	Value	Threshold	Converged?
Maximum Force	0.000024	0.000002	NO
RMS Force	0.000006	0.000001	NO
Maximum Displacement	0.000182	0.000006	NO
RMS Displacement	0.000066	0.000004	NO

Predicted change in Energy=-5.704932D-10

(Hartree/Particle)
HF(PBE1PBE) = -460.854328177
Zero-point correction= 0.112660
Thermal correction to Energy= 0.119362
Thermal correction to Enthalpy= 0.120306
Thermal correction to Gibbs Free Energy= 0.083357
Sum of electronic and zero-point Energies= -460.741668
Sum of electronic and thermal Energies= -460.734966
Sum of electronic and thermal Enthalpies= -460.734022
Sum of electronic and thermal Free Energies= -460.770971
HF(MP2)=-459.6240633
MP2=-460.2175523

$[PMe_3]^{+\cdot}$

P	0.000030	0.000026	-0.326429
C	1.504828	0.859848	0.147804
C	-0.007771	-1.733164	0.147519
H	1.489984	1.877880	-0.247485
H	2.374337	0.330010	-0.247278
H	1.574046	0.899544	1.241918
H	-0.901384	-2.221188	-0.247640
H	-0.007992	-1.813153	1.241621
H	0.881285	-2.229271	-0.247854
C	-1.497101	0.873285	0.147514
H	-1.472930	1.891238	-0.247516
H	-1.566302	0.913314	1.241616
H	-2.371232	0.351419	-0.247977

Convergence failure.

Item	Value	Threshold	Converged?
Maximum Force	0.000006	0.000002	NO
RMS Force	0.000002	0.000001	NO
Maximum Displacement	0.000058	0.000006	NO
RMS Displacement	0.000019	0.000004	NO

Predicted change in Energy=-4.420962D-10

(Hartree/Particle)
HF(PBE1PBE) = -460.572363123
Zero-point correction= 0.112423
Thermal correction to Energy= 0.119464
Thermal correction to Enthalpy= 0.120408
Thermal correction to Gibbs Free Energy= 0.081712
Sum of electronic and zero-point Energies= -460.459940
Sum of electronic and thermal Energies= -460.452899
Sum of electronic and thermal Enthalpies= -460.451955
Sum of electronic and thermal Free Energies= -460.490651
HF(MP2)=-459.3773002
MP2=-459.9373705

$[HPMe_3]^{+}$

P	-0.000006	0.000003	0.331592
C	0.756162	-1.537894	-0.211193
C	0.953786	1.423798	-0.211195
H	0.496818	2.341210	0.166527
H	0.975706	1.456314	-1.303262
H	1.976007	1.350402	0.166716
C	-1.709937	0.114096	-0.211199
H	-2.275989	-0.740269	0.166671
H	-1.749093	0.116646	-1.303266
H	-2.157441	1.036140	0.166578
H	1.779103	-1.600900	0.166644
H	0.773501	-1.573110	-1.303261
H	0.181421	-2.386466	0.166613
H	-0.000006	-0.000008	1.737686

Convergence failure.

Item	Value	Threshold	Converged?
Maximum Force	0.000004	0.000002	NO
RMS Force	0.000001	0.000001	NO
Maximum Displacement	0.000144	0.000006	NO
RMS Displacement	0.000041	0.000004	NO

Predicted change in Energy=-6.631819D-10

(Hartree/Particle)
HF(PBE1PBE) = -461.226207752
Zero-point correction= 0.123730
Thermal correction to Energy= 0.130601
Thermal correction to Enthalpy= 0.131545
Thermal correction to Gibbs Free Energy= 0.094152
Sum of electronic and zero-point Energies= -461.102478
Sum of electronic and thermal Energies= -461.095607
Sum of electronic and thermal Enthalpies= -461.094663
Sum of electronic and thermal Free Energies= -461.132055
HF(MP2)=-460.0052381
MP2= -460.5896228

Miscellaneous fragments

$[H_2PPMe_3]^{+}$

P	-0.336098	0.000000	-0.004274
P	1.861607	0.000001	0.085638
C	-0.919065	1.469097	0.866980
C	-0.919064	-1.469086	0.866999
H	-0.577734	2.372109	0.355816
H	-0.535105	1.470396	1.889536
H	-2.011846	1.466758	0.893296
H	-0.577731	-2.372105	0.355848
H	-2.011844	-1.466749	0.893315
H	-0.535104	-1.470371	1.889556
C	-1.011199	-0.000012	-1.679794
H	-0.674418	0.889483	-2.216470
H	-2.103961	-0.000012	-1.632848
H	-0.674417	-0.889514	-2.216458
H	1.957748	1.042038	-0.878588
H	1.957748	-1.042048	-0.878574

(Hartree/Particle)
HF(PBE1PBE)=-803.0669051
Zero-point correction= 0.133062
Thermal correction to Energy= 0.142042
Thermal correction to Enthalpy= 0.142986
Thermal correction to Gibbs Free Energy= 0.100371
Sum of electronic and zero-point Energies= -802.933843
Sum of electronic and thermal Energies= -802.924863
Sum of electronic and thermal Enthalpies= -802.923919
Sum of electronic and thermal Free Energies= -802.966534
HF(MP2)=-801.3349771
MP2= -802.0422638

$[HPPMe_3]^{+\cdot}$

P	0.301230	0.001721	0.000000
P	-1.895905	0.099649	0.000000
C	0.907387	-0.856535	1.471616
C	0.931938	1.691852	-0.000014
H	0.550941	-1.888703	1.476721
H	0.554071	-0.349746	2.372187
H	2.001182	-0.853114	1.463629
H	0.581249	2.220450	-0.889357
H	2.024833	1.676156	-0.000008
H	0.581238	2.220470	0.889313
C	0.907386	-0.856560	-1.471602
H	0.550940	-1.888728	-1.476689
H	2.001180	-0.853140	-1.463617
H	0.554069	-0.349787	-2.372181
H	-1.959844	-1.326940	0.000004

(Hartree/Particle)
HF(PBE1PBE)=-802.4324016
Zero-point correction= 0.123188
Thermal correction to Energy= 0.132052
Thermal correction to Enthalpy= 0.132996
Thermal correction to Gibbs Free Energy= 0.089916
Sum of electronic and zero-point Energies= -802.309213
Sum of electronic and thermal Energies= -802.300349
Sum of electronic and thermal Enthalpies= -802.299405
Sum of electronic and thermal Free Energies= -802.342485
HF(MP2)=-800.7334825
MP2= -801.4124235

[P^{t}Bu$_3$]

P	0.000255	-0.000090	-0.706068
C	-1.625483	0.755673	-0.001782
C	1.467581	1.029533	-0.001856
C	2.687339	0.720852	-0.887373
C	1.841949	0.822439	1.467598
C	1.181118	2.520580	-0.221327
C	-2.773353	-0.237709	-0.223520
C	-1.967342	1.967586	-0.885866
C	-1.634725	1.181854	1.468264
H	0.892462	2.729630	-1.255584
H	2.099760	3.083665	-0.016304
H	0.409543	2.911138	0.443476
H	3.056198	-0.297782	-0.778708
H	3.506398	1.397153	-0.612151
H	2.454982	0.884476	-1.943549
H	2.184254	-0.193469	1.672428
H	1.013283	1.042335	2.143316
H	2.667666	1.497760	1.728231
H	-1.410338	0.354051	2.143631
H	-2.633214	1.557571	1.728433
H	-0.927674	1.987382	1.674714
H	-2.807956	-0.592617	-1.257718
H	-3.720681	0.276488	-0.020605
H	-2.727478	-1.101065	0.441584
H	-1.993675	1.685861	-1.942363
H	-1.268892	2.795640	-0.776516
H	-2.962155	2.339186	-0.609724
C	0.158449	-1.785703	-0.001718
C	1.592807	-2.283350	-0.221646
H	2.317305	-1.810551	0.442710
H	1.917913	-2.138042	-1.256022
H	1.621052	-3.360445	-0.016519
C	-0.208114	-2.004724	1.467851
H	-0.037000	-3.057308	1.729754
H	-1.258961	-1.791949	1.671947
H	0.396949	-1.396536	2.142821
C	-0.719397	-2.688154	-0.886162
H	-0.542712	-3.735469	-0.610670
H	-0.462682	-2.569158	-1.942664
H	-1.785924	-2.498540	-0.776425

(Hartree/Particle)
HF(PBE1PBE)=-814.2873629
Zero-point correction= 0.368801
Thermal correction to Energy= 0.386662
Thermal correction to Enthalpy= 0.387606
Thermal correction to Gibbs Free Energy= 0.327457
Sum of electronic and zero-point Energies= -813.918562
Sum of electronic and thermal Energies= -813.900701
Sum of electronic and thermal Enthalpies= -813.899757
Sum of electronic and thermal Free Energies= -813.959906
HF(MP2)=-810.9568497
MP2=-812.9694272

[P(H)^{t}Bu$_2$]

P	-0.000462	-1.003659	-0.140899
C	1.567945	0.046376	0.007884
C	-1.572454	0.045788	0.003759
C	-2.700044	-0.959648	-0.267701
C	-1.594557	1.118796	-1.085260
C	-1.807078	0.676682	1.376338
C	2.645616	-0.964764	0.419844
C	1.540587	1.185219	1.023519
C	1.919217	0.592014	-1.379953
H	-1.822508	-0.082828	2.163890
H	-2.779699	1.186002	1.388242
H	-1.046369	1.414394	1.634564
H	-2.611503	-1.404254	-1.262881
H	-3.671712	-0.454837	-0.201276
H	-2.699400	-1.774172	0.464669
H	-1.393125	0.695312	-2.074557
H	-0.863264	1.909627	-0.896785
H	-2.584571	1.589526	-1.120468
H	1.976786	-0.212529	-2.119117
H	2.895035	1.091732	-1.346603
H	1.186796	1.322028	-1.734349
H	2.474809	-1.346877	1.431096
H	3.630064	-0.481780	0.404765
H	2.681432	-1.818635	-0.264459
H	1.268360	0.832154	2.022348
H	0.842609	1.975720	0.736297
H	2.536648	1.642042	1.091082
H	-0.008850	-1.470511	1.206449

(Hartree/Particle)
HF(PBE1PBE1)=-657.2094406
Zero-point correction= 0.253486
Thermal correction to Energy= 0.266301
Thermal correction to Enthalpy= 0.267245
Thermal correction to Gibbs Free Energy= 0.216487
Sum of electronic and zero-point Energies= -656.955955
Sum of electronic and thermal Energies= -656.943140
Sum of electronic and thermal Enthalpies= -656.942196
Sum of electronic and thermal Free Energies= -656.992954
HF(MP2)=-654.8165617
MP2=-656.1937167

[^{t}Bu(H)P]$^+$

P	0.730168	-0.593552	0.107483
C	-0.793519	0.071763	-0.016992
C	-1.021529	1.539869	0.027572
C	-1.981310	-0.824992	-0.064295
C	2.264718	0.327110	-0.096764
H	2.132342	1.342026	0.281379
H	2.556138	0.352374	-1.148996

H	3.042290	-0.166873	0.490171
H	-1.672108	1.787543	0.873505
H	-1.565267	1.832714	-0.879031
H	-0.112337	2.136928	0.091243
H	-2.644313	-0.606002	0.779581
H	-1.738286	-1.888185	-0.058817
H	-2.547593	-0.602197	-0.976774
H	0.786457	-1.967544	-0.161621

(Hartree/Particle)
HF(PBE1PBE)=-499.2334919
Zero-point correction= 0.128371
Thermal correction to Energy= 0.136555
Thermal correction to Enthalpy= 0.137499
Thermal correction to Gibbs Free Energy= 0.095969
Sum of electronic and zero-point Energies= -499.105121
Sum of electronic and thermal Energies= -499.096937
Sum of electronic and thermal Enthalpies= -499.095993
Sum of electronic and thermal Free Energies= -499.137523
HF(MP2)=-497.8101395
MP2= -498.5283592

$[^tBu(H)P]^{\cdot}$

P	1.556326	0.137798	0.000000
C	-0.317317	-0.001576	0.000000
C	-0.888194	1.418455	-0.000001
C	-0.778090	-0.749578	1.253551
C	-0.778090	-0.749580	-1.253550
H	-0.447940	-0.248207	-2.168512
H	-1.874400	-0.797608	-1.276985
H	-0.397229	-1.775060	-1.272053
H	-0.570015	1.978814	0.884627
H	-1.983947	1.384959	-0.000001
H	-0.570015	1.978812	-0.884630
H	-0.447940	-0.248203	2.168513
H	-0.397230	-1.775058	1.272056
H	-1.874400	-0.797605	1.276986
H	1.788379	-1.274137	0.000000

(Hartree/Particle)
HF(PBE1PBE)=-499.4824133
Zero-point correction= 0.129219
Thermal correction to Energy= 0.136593
Thermal correction to Enthalpy= 0.137538
Thermal correction to Gibbs Free Energy= 0.098318
Sum of electronic and zero-point Energies= -499.353194
Sum of electronic and thermal Energies= -499.345820
Sum of electronic and thermal Enthalpies= -499.344876
Sum of electronic and thermal Free Energies= -499.384096
HF(MP2)=-498.0525035
MP2= -498.7780958

Dihydrogen, $[H_2]$

H	0.000000	0.000000	0.373195
H	0.000000	0.000000	-0.373195

(Hartree/Particle)
HF(PBE1PBE)=-1.1681575
Zero-point correction= 0.010050
Thermal correction to Energy= 0.012410
Thermal correction to Enthalpy= 0.013354
Thermal correction to Gibbs Free Energy= -0.001447
Sum of electronic and zero-point Energies= -1.158108
Sum of electronic and thermal Energies= -1.155748
Sum of electronic and thermal Enthalpies= -1.154803
Sum of electronic and thermal Free Energies= -1.169604
HF(MP2)=-1.1324225
MP2= -1.160256

$[(C_6H_4)_2P]^+$

P	0.000000	1.974547	0.000000
H	0.000000	0.049656	-4.581842
C	0.000000	-0.106842	-3.509597
C	0.000000	0.981718	-2.642132
H	0.000000	1.995542	-3.030220
C	0.000000	0.746279	-1.260411
C	0.000000	-0.575476	-0.737592
C	0.000000	-1.641576	-1.604436
H	0.000000	-2.664827	-1.244432
C	0.000000	-1.395529	-2.993038
H	0.000000	-2.240917	-3.673483
C	0.000000	-0.575476	0.737592
C	0.000000	0.746279	1.260411
C	0.000000	0.981718	2.642132
H	0.000000	1.995542	3.030220
C	0.000000	-0.106842	3.509597
H	0.000000	0.049656	4.581842
C	0.000000	-1.395529	2.993038
H	0.000000	-2.240917	3.673483
C	0.000000	-1.641576	1.604436
H	0.000000	-2.664827	1.244432

(Hartree/Particle)
HF(PBE1PBE)=-802.6109613
Zero-point correction= 0.161497
Thermal correction to Energy= 0.170973
Thermal correction to Enthalpy= 0.171917
Thermal correction to Gibbs Free Energy= 0.126372
Sum of electronic and zero-point Energies= -802.449464
Sum of electronic and thermal Energies= -802.439989
Sum of electronic and thermal Enthalpies= -802.439045
Sum of electronic and thermal Free Energies= -802.484590
HF(MP2)=-799.676595
MP2= -801.3990047

Dispersion Corrected PBE1PBE Optimizations and Frequency Analysis

Cartesian coordinates, enthalpies and Gibbs energies for R = Et and ^tBu substituted structures optimized at the PBE1PBE/6-311++G(d,p) level with Grimme's D3 (GD3) dispersion correction in the gas phase (298 K) absence of a counterion or solvent, listed in the order: $[R_2PPMe_3]^+$, $[R(H)PPMe_3]^+$, $[RPPMe_3]^{+\cdot}$, $[RPPMe_3]$, $[RPPMe_2]^+$, $[R_2P]^+$, $[R_2P]^{\cdot}$, $[R]^+$, $[R]^{\cdot}$, alkanes, olefins, and $[PMe_3]$ derivatives. The starting geometries used were the geometries obtained at the same level of theory but without dispersion correction applied (*e.g.* PBE1PBE/6-311++G(d,p)).

$[R_2PPMe_3]^+$

$[Et_2PPMe_3]^+$

P	-1.235007	-0.346230	0.015531
P	0.807085	0.077698	-0.717187
C	-1.578379	-2.101390	-0.250081
C	-2.427958	0.588841	-0.968844
C	1.701681	-1.268605	0.208836

C	1.219369	1.564491	0.320799
H	-0.958349	-2.712139	0.408418
H	-1.370658	-2.365366	-1.289590
H	-2.631538	-2.299363	-0.034886
H	-2.273878	1.660911	-0.838062
H	-3.442883	0.328263	-0.657985
H	-2.294624	0.340129	-2.023865
C	-1.511913	0.022928	1.765955
H	-0.827558	-0.564523	2.381672
H	-2.540838	-0.229924	2.035201
H	-1.342708	1.084677	1.955047
H	1.372115	-2.223842	-0.211106
H	1.447485	-1.260861	1.274698
C	3.212396	-1.125680	0.024163
C	0.438369	2.824954	-0.031037
H	2.283200	1.726782	0.115508
H	1.144208	1.325142	1.387093
H	3.488590	-1.101181	-1.033593
H	3.721305	-1.977873	0.480290
H	3.600347	-0.222578	0.500930
H	0.465424	3.033396	-1.103910
H	0.867929	3.687397	0.483638
H	-0.610136	2.765714	0.276585

(Hartree/Particle)
HF(PBE1PBE)= -960.1991351
Zero-point correction= 0.249380 (Hartree/Particle)
Thermal correction to Energy= 0.264236
Thermal correction to Enthalpy= 0.265181
Thermal correction to Gibbs Free Energy= 0.208741
Sum of electronic and zero-point Energies= -959.949755
Sum of electronic and thermal Energies= -959.934899
Sum of electronic and thermal Enthalpies= -959.933955
Sum of electronic and thermal Free Energies= -959.990394

[^{t}Bu$_2$PPMe$_3$]$^+$

P	-1.728219	-0.004713	-0.017439
P	0.367877	0.001965	-0.771845
C	-2.516645	1.604028	-0.300395
C	1.120167	-1.571889	0.003187
C	1.119942	1.575590	0.004326
C	-2.082587	-0.439224	1.710414
H	-2.386690	1.905094	-1.341316
H	-2.114320	2.373117	0.357760
H	-3.584419	1.485259	-0.096037
H	-1.785568	-1.468371	1.914195
H	-3.158314	-0.339434	1.881241
H	-1.545504	0.229506	2.383874
C	-2.694041	-1.128889	-1.062806
H	-2.513722	-0.882675	-2.111202
H	-3.753743	-0.987857	-0.833870
H	-2.424613	-2.169053	-0.889752
C	0.697429	2.735882	-0.907720
C	2.644392	1.445258	-0.110453
C	0.739638	1.877250	1.453310
C	0.168509	-2.739512	-0.277855
C	1.430814	-1.519572	1.496737
C	2.405496	-1.834028	-0.799359
H	-0.324664	2.100846	1.567485
H	1.280780	2.769823	1.786044
H	0.998311	1.066321	2.135150
H	0.996906	2.561637	-1.944364
H	1.192266	3.651176	-0.565894
H	-0.375480	2.929533	-0.894098
H	2.962596	1.196606	-1.127066
H	3.062201	0.712131	0.580310
H	3.087458	2.415154	0.138697
H	2.203405	-1.909550	-1.871346
H	2.828799	-2.789358	-0.471164
H	3.169318	-1.072235	-0.646727
H	-0.713234	-2.728911	0.368997
H	0.693794	-3.676729	-0.068139
H	-0.149761	-2.774436	-1.324237
H	0.544726	-1.321898	2.104199
H	2.186425	-0.772390	1.741802
H	1.829500	-2.491447	1.808404

(Hartree/Particle)
Zero-point correction= 0.362846
Thermal correction to Energy= 0.382336
Thermal correction to Enthalpy= 0.383280
Thermal correction to Gibbs Free Energy= 0.319193
Sum of electronic and zero-point Energies= -1116.936759
Sum of electronic and thermal Energies= -1116.917269
Sum of electronic and thermal Enthalpies= -1116.916325
Sum of electronic and thermal Free Energies= -1116.980412
HF(PBE1PBE)= -1117.2996049

[R(H)PPMe$_3$]$^+$

[Et(H)PPMe$_2$]$^+$

P	1.080287	-0.045331	-0.012092
P	-0.885515	0.928187	0.139494
C	1.455778	-0.808663	1.580813
C	2.304526	1.240583	-0.339555
C	-2.009332	-0.557358	0.075754
H	0.737856	-1.603575	1.794465
H	1.402517	-0.055978	2.370314
H	2.461281	-1.235914	1.553894
H	2.111027	1.704066	-1.309425
H	3.304127	0.798403	-0.346968
H	2.253499	2.005125	0.438658
C	1.214887	-1.309427	-1.297921
H	0.517865	-2.125228	-1.097207
H	2.233180	-1.707880	-1.310028
H	0.987145	-0.872131	-2.272326
H	-1.891911	-1.089293	1.025319
H	-1.735870	-1.238707	-0.733483
C	-3.455050	-0.088336	-0.085211
H	-3.756999	0.581351	0.724078
H	-4.124480	-0.951437	-0.070025
H	-3.608013	0.431303	-1.034824
H	-0.877672	1.256256	-1.246745

(Hartree/Particle)
HF(PBE1PBE)= -881.6357461
Zero-point correction= 0.191219
Thermal correction to Energy= 0.203231
Thermal correction to Enthalpy= 0.204175

Thermal correction to Gibbs Free Energy= 0.153416
Sum of electronic and zero-point Energies= -881.444527
Sum of electronic and thermal Energies= -881.432515
Sum of electronic and thermal Enthalpies= -881.431571
Sum of electronic and thermal Free Energies= -881.482330

[^{t}Bu(H)PPMe$_2$]$^+$

P	-1.473957	0.012674	0.002811
P	0.398958	-1.151204	-0.101169
C	-1.568577	1.206791	-1.350375
C	1.829822	0.074159	0.011239
C	-1.839186	0.869219	1.555701
H	-1.332735	0.715184	-2.296532
H	-0.867791	2.026367	-1.183607
H	-2.583541	1.609939	-1.398004
H	-1.816039	0.152203	2.379151
H	-2.838103	1.310044	1.491962
H	-1.106922	1.653844	1.744471
C	-2.784608	-1.207462	-0.248562
H	-2.645162	-1.704003	-1.210852
H	-3.757016	-0.708416	-0.230810
H	-2.753605	-1.958310	0.544332
C	2.111106	0.588486	-1.402636
C	2.993874	-0.810279	0.481085
C	1.629878	1.233046	0.981724
H	0.868620	1.936825	0.630322
H	2.563671	1.799271	1.064918
H	1.363475	0.890990	1.985912
H	2.280240	-0.229689	-2.107976
H	3.014861	1.205812	-1.387165
H	1.300245	1.211875	-1.790242
H	3.146682	-1.675910	-0.171159
H	2.851671	-1.166529	1.505477
H	3.915246	-0.219316	0.459472
H	0.287351	-1.495983	1.276636

(Hartree/Particle)
HF(PBE1PBE)= -960.1917958
Zero-point correction= 0.247457
Thermal correction to Energy= 0.261846
Thermal correction to Enthalpy= 0.262790
Thermal correction to Gibbs Free Energy= 0.208043
Sum of electronic and zero-point Energies= -959.944338
Sum of electronic and thermal Energies= -959.929950
Sum of electronic and thermal Enthalpies= -959.929006
Sum of electronic and thermal Free Energies= -959.983753

[RPPMe$_3$]$^{+\cdot}$

[EtPPMe$_3$]$^{+\cdot}$

P	1.059402	-0.034254	-0.000002
P	-0.890761	0.991757	0.000020
C	1.278130	-1.071602	1.466921
C	2.339305	1.238305	0.000115
C	-1.985333	-0.504733	0.000034
H	0.546051	-1.881349	1.466952
H	1.151163	-0.469385	2.368980
H	2.284106	-1.500107	1.458061
H	2.238301	1.865075	-0.888714
H	3.325920	0.768094	0.000094
H	2.238274	1.864940	0.889036
C	1.278190	-1.071388	-1.467068
H	0.546102	-1.881126	-1.467255
H	2.284161	-1.499907	-1.458223
H	1.151272	-0.469036	-2.369043
H	-1.763433	-1.118452	0.882109
H	-1.763378	-1.118525	-0.881975
C	-3.454595	-0.084235	-0.000028
H	-3.705347	0.505347	0.885210
H	-4.091691	-0.971470	-0.000006
H	-3.705295	0.505267	-0.885334

(Hartree/Particle)
HF(PBE1PBE)= -881.0037561
Zero-point correction= 0.181331
Thermal correction to Energy= 0.193300
Thermal correction to Enthalpy= 0.194244
Thermal correction to Gibbs Free Energy= 0.141734
Sum of electronic and zero-point Energies= -880.822425
Sum of electronic and thermal Energies= -880.810456
Sum of electronic and thermal Enthalpies= -880.809512
Sum of electronic and thermal Free Energies= -880.862022

[^{t}BuPPMe$_3$]$^{+\cdot}$

P	-1.450104	-0.001427	-0.000290
P	0.410216	-1.197289	-0.008381
C	-1.543353	1.382935	-1.165013
C	1.814992	0.054823	0.001719
C	-1.856259	0.618499	1.653114
H	-1.317888	1.034749	-2.174807
H	-0.842433	2.170707	-0.886988
H	-2.560384	1.785038	-1.143703
H	-1.847614	-0.209669	2.364911
H	-2.854924	1.063556	1.630174
H	-1.129989	1.368708	1.967181
C	-2.742329	-1.178309	-0.464774
H	-2.560854	-1.548467	-1.476085
H	-3.717221	-0.685361	-0.428233
H	-2.738892	-2.023988	0.226430
C	2.015225	0.601440	-1.416995
C	3.032483	-0.792844	0.403413
C	1.622670	1.192021	1.004328
H	0.820866	1.875826	0.709967
H	2.541974	1.785608	1.049771
H	1.423982	0.824829	2.015498
H	2.160788	-0.197955	-2.148811
H	2.916385	1.224687	-1.429004
H	1.182346	1.225768	-1.748972
H	3.193108	-1.631960	-0.280260
H	2.940285	-1.188423	1.418891
H	3.928222	-0.164306	0.369368

(Hartree/Particle)
HF(PBE1PBE)= -959.5590309
Zero-point correction= 0.237974
Thermal correction to Energy= 0.252268

Thermal correction to Enthalpy= 0.253212
Thermal correction to Gibbs Free Energy= 0.197983
Sum of electronic and zero-point Energies= -959.321057
Sum of electronic and thermal Energies= -959.306763
Sum of electronic and thermal Enthalpies= -959.305818
Sum of electronic and thermal Free Energies= -959.361048

[RPPMe$_3$]

[EtPPMe$_3$]

P	0.965344	0.004053	0.000000
P	-0.872932	1.000904	0.000000
C	1.344725	-1.080936	1.426951
C	2.294479	1.243116	-0.000001
C	-1.969139	-0.546185	-0.000001
H	0.595190	-1.873455	1.484503
H	1.286465	-0.483601	2.338436
H	2.336669	-1.533308	1.335096
H	2.199946	1.870834	-0.887671
H	3.270808	0.752548	-0.000001
H	2.199946	1.870835	0.887668
C	1.344724	-1.080938	-1.426949
H	0.595191	-1.873459	-1.484498
H	2.336669	-1.533308	-1.335095
H	1.286461	-0.483606	-2.338436
H	-1.781411	-1.165327	0.884744
H	-1.781413	-1.165325	-0.884747
C	-3.431975	-0.112023	0.000001
H	-3.663320	0.491764	0.883097
H	-4.100950	-0.978921	0.000001
H	-3.663322	0.491765	-0.883094

(Hartree/Particle)
HF(PBE1PBE)= -881.2274479
Zero-point correction= 0.181181
Thermal correction to Energy= 0.192958
Thermal correction to Enthalpy= 0.193902
Thermal correction to Gibbs Free Energy= 0.143567
Sum of electronic and zero-point Energies= -881.046267
Sum of electronic and thermal Energies= -881.034490
Sum of electronic and thermal Enthalpies= -881.033546
Sum of electronic and thermal Free Energies= -881.083881

[^{t}BuPPMe$_3$]

P	-1.363444	-0.038125	0.001129
P	0.368659	-1.223719	0.027398
C	-1.595359	1.233831	-1.298290
C	1.796818	0.051300	0.004344
C	-1.880166	0.859283	1.518022
H	-1.401740	0.778717	-2.270963
H	-0.889829	2.053121	-1.146841
H	-2.613635	1.633022	-1.267474
H	-1.917402	0.140177	2.338404
H	-2.861097	1.326154	1.384886
H	-1.144570	1.625548	1.765778
C	-2.733176	-1.205271	-0.273049
H	-2.633106	-1.649793	-1.264056
H	-3.692180	-0.687350	-0.191996
H	-2.682424	-1.998510	0.475050
C	2.052129	0.582111	-1.408470
C	3.014578	-0.768055	0.448462
C	1.620636	1.224013	0.967815
H	0.827634	1.904625	0.638253
H	2.544462	1.816629	1.017754
H	1.381578	0.878989	1.977728
H	2.210149	-0.238933	-2.112554
H	2.944444	1.224327	-1.422382
H	1.210250	1.174057	-1.777971
H	3.166686	-1.639184	-0.197510
H	2.899003	-1.126162	1.475554
H	3.920800	-0.151053	0.397413

(Hartree/Particle)
HF(PBE1PBE)= -959.7813295
Zero-point correction= 0.237587
Thermal correction to Energy= 0.251604
Thermal correction to Enthalpy= 0.252548
Thermal correction to Gibbs Free Energy= 0.198665
Sum of electronic and zero-point Energies= -959.543743
Sum of electronic and thermal Energies= -959.529725
Sum of electronic and thermal Enthalpies= -959.528781
Sum of electronic and thermal Free Energies= -959.582664

[RPPMe$_2$]$^+$

[EtPPMe$_2$]$^+$

P	0.985638	0.058904	-0.025971
P	-0.554915	-1.214549	-0.162780
C	0.960504	1.846081	-0.284843
C	-1.968817	-0.064066	-0.534755
H	1.284826	2.345367	0.632721
H	-0.047346	2.175309	-0.539451
H	1.647579	2.102149	-1.096274
C	2.622561	-0.582037	0.370572
H	2.986009	-0.113088	1.288985
H	3.311309	-0.356492	-0.448034
H	2.562897	-1.662777	0.511447
H	-2.708173	-0.718458	-1.008447
H	-1.688277	0.681922	-1.282591
C	-2.556787	0.575418	0.724385
H	-2.832059	-0.172534	1.472052
H	-3.463237	1.124630	0.458588
H	-1.859138	1.276271	1.190115

(Hartree/Particle)
HF(PBE1PBE)= -841.0971659
Zero-point correction= 0.143426
Thermal correction to Energy= 0.153413
Thermal correction to Enthalpy= 0.154357
Thermal correction to Gibbs Free Energy= 0.107790
Sum of electronic and zero-point Energies= -840.953740
Sum of electronic and thermal Energies= -840.943753
Sum of electronic and thermal Enthalpies= -840.942809
Sum of electronic and thermal Free Energies= -840.989376

[^{t}BuPPMe$_2$]$^+$

P	-1.525550	-0.027063	0.000000
P	0.146853	-1.130646	0.000000
C	-1.790764	1.762629	0.000000
C	1.619934	0.057822	0.000000
C	-3.091697	-0.925140	0.000000
H	-0.841596	2.295145	-0.000004
H	-2.364113	2.033543	0.891426
H	-2.364119	2.033542	-0.891423
H	-2.895066	-1.998462	0.000001
H	-3.665485	-0.657724	-0.891463
H	-3.665485	-0.657723	0.891464
C	1.641206	0.911097	-1.269931
C	2.820698	-0.902613	0.000001
C	1.641204	0.911098	1.269930
H	0.827147	1.638352	1.306832
H	2.582989	1.469283	1.296412
H	1.596765	0.301665	2.176670
H	1.596767	0.301661	-2.176670
H	2.582990	1.469281	-1.296413
H	0.827148	1.638349	-1.306835
H	2.839665	-1.541116	-0.888012
H	2.839664	-1.541115	0.888016
H	3.739702	-0.308404	0.000001

(Hartree/Particle)
HF(PBE1PBE)= -919.6534785
Zero-point correction= 0.199359
Thermal correction to Energy= 0.212047
Thermal correction to Enthalpy= 0.212991
Thermal correction to Gibbs Free Energy= 0.160445
Sum of electronic and zero-point Energies= -919.454119
Sum of electronic and thermal Energies= -919.441431
Sum of electronic and thermal Enthalpies= -919.440487
Sum of electronic and thermal Free Energies= -919.493034

$[R_2P]^+$

$[Et_2P]^+$

P	0.244459	-0.610126	-0.545947
C	1.690995	-0.343607	0.504598
C	-1.138024	-0.248654	0.590250
H	2.594952	-0.842076	0.167084
H	1.533083	-0.417233	1.577110
C	1.493249	1.058130	-0.063166
C	-2.332353	0.423433	-0.081976
H	-0.796375	0.283336	1.483774
H	-1.410296	-1.264324	0.928171
H	0.635515	1.076341	-0.891472
H	2.310180	1.401777	-0.698750
H	1.120239	1.787978	0.653860
H	-2.098405	1.446566	-0.387652
H	-3.166295	0.471972	0.620629
H	-2.672677	-0.128258	-0.961781

(Hartree/Particle)
HF(PBE1PBE)= -499.2166839
Zero-point correction= 0.131116
Thermal correction to Energy= 0.138083
Thermal correction to Enthalpy= 0.139028
Thermal correction to Gibbs Free Energy= 0.100469
Sum of electronic and zero-point Energies= -499.085568
Sum of electronic and thermal Energies= -499.078601
Sum of electronic and thermal Enthalpies= -499.077656
Sum of electronic and thermal Free Energies= -499.116215

$[^t\mathrm{Bu}_2\mathrm{P}]^+$

P	-0.066947	-0.879483	-0.370118
C	-1.580999	0.085072	0.018112
C	1.496973	0.065435	-0.031870
C	2.599377	-1.003551	-0.102268
C	1.638552	0.866187	1.256593
C	1.558806	0.960222	-1.286648
C	-2.789486	-0.530866	-0.680772
C	-1.631592	1.600551	0.022413
C	-1.279381	-0.575757	1.367280
H	1.365424	0.407861	-2.216828
H	2.576367	1.353956	-1.377245
H	0.867873	1.803134	-1.231256
H	2.563452	-1.683755	0.753726
H	3.574141	-0.506545	-0.091383
H	2.545697	-1.601177	-1.017462
H	1.601436	0.222006	2.139618
H	0.899682	1.660137	1.365327
H	2.622635	1.345502	1.253188
H	-0.420986	-1.398448	1.273245
H	-2.060840	-1.253222	1.717977
H	-0.882268	0.101728	2.120227
H	-2.864740	-0.166264	-1.707414
H	-3.701610	-0.241110	-0.149847
H	-2.746501	-1.623434	-0.710964
H	-1.662782	1.978516	-1.001554
H	-0.793948	2.066327	0.537972
H	-2.552332	1.923285	0.517406

(Hartree/Particle)
HF(PBE1PBE)=-656.3322035
Zero-point correction= 0.242993
Thermal correction to Energy= 0.255536
Thermal correction to Enthalpy= 0.256480
Thermal correction to Gibbs Free Energy= 0.206482
Sum of electronic and zero-point Energies= -656.089211
Sum of electronic and thermal Energies= -656.076667
Sum of electronic and thermal Enthalpies= -656.075723
Sum of electronic and thermal Free Energies= -656.125721

$[R_2P]^{\cdot}$

$[Et_2P]^{\cdot}$

P	-0.059223	-0.916728	-0.213224
C	1.584176	-0.348289	0.442151
C	-1.092451	0.557196	0.241842
H	2.262078	-1.204633	0.365852
H	1.472054	-0.132098	1.512704
C	2.187699	0.858011	-0.277605
C	-2.573169	0.348319	-0.055734
H	-0.717752	1.431885	-0.304435
H	-0.937178	0.771425	1.307675
H	2.294973	0.668307	-1.349775
H	3.180869	1.089406	0.120272
H	1.570265	1.752069	-0.156996
H	-2.740852	0.151584	-1.118912
H	-3.157588	1.232906	0.214759
H	-2.976062	-0.501360	0.503289

(Hartree/Particle)
HF(PBE1PBE)= -499.4918385
Zero-point correction= 0.131565
Thermal correction to Energy= 0.139240
Thermal correction to Enthalpy= 0.140184
Thermal correction to Gibbs Free Energy= 0.098490
Sum of electronic and zero-point Energies= -499.360274
Sum of electronic and thermal Energies= -499.352599
Sum of electronic and thermal Enthalpies= -499.351655
Sum of electronic and thermal Free Energies= -499.393349

[^{t}Bu$_2$P]$^\cdot$

P	0.000000	1.062373	0.000000
C	-1.547145	-0.027807	-0.001731
C	1.547145	-0.027807	0.001731
C	2.672110	0.933841	0.410450
C	1.841207	-0.544803	-1.411685
C	1.511489	-1.190293	0.993431
C	-2.672110	0.933841	-0.410450
C	-1.841208	-0.544803	1.411684
C	-1.511489	-1.190292	-0.993431
H	1.294046	-0.848308	2.009841
H	2.492142	-1.682981	1.012827
H	0.772733	-1.947182	0.721312
H	2.732861	1.792694	-0.265609
H	3.636512	0.412061	0.379876
H	2.525943	1.316270	1.425364
H	1.880107	0.274111	-2.136707
H	1.096047	-1.264800	-1.754549
H	2.818333	-1.045296	-1.422384
H	-1.294045	-0.848307	-2.009842
H	-2.492141	-1.682981	-1.012828
H	-0.772732	-1.947182	-0.721312
H	-2.732861	1.792694	0.265609
H	-3.636512	0.412061	-0.379876
H	-2.525942	1.316271	-1.425363
H	-1.880108	0.274110	2.136707
H	-1.096048	-1.264801	1.754548
H	-2.818333	-1.045296	1.422383

(Hartree/Particle)
HF(PBE1PBE)= -656.5936477
Zero-point correction= 0.244180
Thermal correction to Energy= 0.256795
Thermal correction to Enthalpy= 0.257740
Thermal correction to Gibbs Free Energy= 0.206804
Sum of electronic and zero-point Energies= -656.349468
Sum of electronic and thermal Energies= -656.336852
Sum of electronic and thermal Enthalpies= -656.335908
Sum of electronic and thermal Free Energies= -656.386844

[R]$^+$

[Et]$^+$

C	-0.064225	0.688854	0.000000
C	-0.064225	-0.688854	0.000000
H	-0.071392	1.244959	0.936365
H	-0.071392	1.244959	-0.936365
H	-0.071392	-1.244959	0.936365
H	1.056263	0.000000	0.000000
H	-0.071392	-1.244959	-0.936365

(Hartree/Particle)
HF(PBE1PBE)= -78.7763555
Zero-point correction= 0.060835
Thermal correction to Energy= 0.063985
Thermal correction to Enthalpy= 0.064929
Thermal correction to Gibbs Free Energy= 0.038339
Sum of electronic and zero-point Energies= -78.715521
Sum of electronic and thermal Energies= -78.712371
Sum of electronic and thermal Enthalpies= -78.711427
Sum of electronic and thermal Free Energies= -78.738016

[^{t}Bu]$^+$

C	0.003165	-0.002219	-0.011845
C	1.375897	-0.482875	-0.014557
C	-1.107193	-0.944171	-0.012345
C	-0.265012	1.426180	0.017596
H	-0.140452	1.733898	-1.041382
H	0.499352	1.989685	0.560437
H	-1.279735	1.689567	0.316268
H	1.506422	-1.367875	-0.644365
H	1.540176	-0.850433	1.018694
H	2.120810	0.287753	-0.211636
H	-0.834259	-1.970321	-0.252069
H	-1.934890	-0.581255	-0.633517
H	-1.518564	-0.912509	1.014469

(Hartree/Particle)
HF(PBE1PBE)=-157.3915507
Zero-point correction= 0.116545
Thermal correction to Energy= 0.122823
Thermal correction to Enthalpy= 0.123767
Thermal correction to Gibbs Free Energy= 0.087396
Sum of electronic and zero-point Energies= -157.275006
Sum of electronic and thermal Energies= -157.268728
Sum of electronic and thermal Enthalpies= -157.267784
Sum of electronic and thermal Free Energies= -157.304155

[R]$^\cdot$

[Et]$^\cdot$

C	-0.791057	0.000000	-0.019472
C	0.691206	0.000000	-0.001631
H	-1.349336	-0.927135	0.042369
H	-1.349336	0.927135	0.042369
H	1.104968	-0.886448	-0.492497
H	1.087837	0.000000	1.026874
H	1.104968	0.886448	-0.492496

(Hartree/Particle)
HF(PBE1PBE)=-79.0758982
Zero-point correction= 0.059330
Thermal correction to Energy= 0.063298
Thermal correction to Enthalpy= 0.064242
Thermal correction to Gibbs Free Energy= 0.035138
Sum of electronic and zero-point Energies= -79.016568
Sum of electronic and thermal Energies= -79.012600
Sum of electronic and thermal Enthalpies= -79.011656
Sum of electronic and thermal Free Energies= -79.040760

[^{t}Bu]$^\cdot$

C	3.279499	2.102106	2.026807
C	2.427861	3.305338	2.234006
C	4.721382	2.296569	1.711956
C	2.852131	0.813995	2.638443
H	1.772214	0.658572	2.545408
H	3.083258	0.780529	3.718577
H	3.362798	-0.041083	2.183368
H	2.658465	4.095584	1.511949

H	2.578780	3.742079	3.237852
H	1.362012	3.067354	2.154213
H	4.875837	3.120021	1.006897
H	5.170202	1.392309	1.287803
H	5.305361	2.543256	2.617132

(Hartree/Particle)
HF(PBE1PBE)= -157.6400794
Zero-point correction= 0.116641
Thermal correction to Energy= 0.122956
Thermal correction to Enthalpy= 0.123900
Thermal correction to Gibbs Free Energy= 0.087328
Sum of electronic and zero-point Energies= -157.523438
Sum of electronic and thermal Energies= -157.517124
Sum of electronic and thermal Enthalpies= -157.516179
Sum of electronic and thermal Free Energies= -157.552751

Alkanes

Propane, [$CH_3CH_2CH_3$]

H	0.000000	2.167863	0.362200
C	0.000000	1.267286	-0.258804
H	-0.882841	1.308599	-0.905777
H	0.882841	1.308599	-0.905777
C	0.000000	0.000000	0.585603
H	0.875883	0.000000	1.245372
H	-0.875883	0.000000	1.245372
C	0.000000	-1.267286	-0.258804
H	-0.882841	-1.308599	-0.905777
H	0.882841	-1.308599	-0.905777
H	0.000000	-2.167863	0.362200

(Hartree/Particle)
HF(PBE1PBE)= -119.020566
Zero-point correction= 0.103523
Thermal correction to Energy= 0.108027
Thermal correction to Enthalpy= 0.108971
Thermal correction to Gibbs Free Energy= 0.078556
Sum of electronic and zero-point Energies= -118.917043
Sum of electronic and thermal Energies= -118.912539
Sum of electronic and thermal Enthalpies= -118.911595
Sum of electronic and thermal Free Energies= -118.942010

Neopentane, [$(CH_3)_4C$]

H	1.790256	-0.389752	1.212984
C	1.104834	-0.193301	0.381602
H	1.328727	0.804975	-0.009426
H	1.319611	-0.919710	-0.409464
C	-0.352558	-0.291048	0.836281
C	-1.276734	-0.010866	-0.350177
H	-1.102605	0.991298	-0.756294
H	-1.111536	-0.733337	-1.156610
H	-2.328906	-0.074487	-0.052734
C	-0.612653	0.736738	1.939128
H	0.036923	0.559559	2.802981
H	-1.650961	0.688970	2.284410
H	-0.424501	1.754440	1.580698
C	-0.625612	-1.696741	1.374533
H	-1.664221	-1.795417	1.707914
H	0.023630	-1.924565	2.226692
H	-0.446812	-2.454493	0.604191

(Hartree/Particle)
HF(PBE1PBE)= -197.5777792
Zero-point correction= 0.159508
Thermal correction to Energy= 0.166338
Thermal correction to Enthalpy= 0.167282
Thermal correction to Gibbs Free Energy= 0.130509
Sum of electronic and zero-point Energies= -197.418271
Sum of electronic and thermal Energies= -197.411441
Sum of electronic and thermal Enthalpies= -197.410497
Sum of electronic and thermal Free Energies= -197.447271

Olefins

Ethene, [CH_2CH_2]

H	-0.924299	1.233684	0.000000
C	0.000000	0.663506	0.000000
H	0.924299	1.233684	0.000000
c	0.000000	-0.663506	0.000000
H	-0.924299	-1.233684	0.000000
H	0.924299	-1.233684	0.000000

(Hartree/Particle)
HF(PBE1PBE)= -78.5048291
Zero-point correction= 0.051026
Thermal correction to Energy= 0.054068
Thermal correction to Enthalpy= 0.055012
Thermal correction to Gibbs Free Energy= 0.029502
Sum of electronic and zero-point Energies= -78.453803
Sum of electronic and thermal Energies= -78.450761
Sum of electronic and thermal Enthalpies= -78.449817
Sum of electronic and thermal Free Energies= -78.475327

Isobutene, [$CH_2C(CH_3)_2$]

H	0.000000	0.926227	2.022561
C	0.000000	0.000000	1.455810
H	0.000000	-0.926227	2.022561
C	0.000000	0.000000	0.122867
C	0.000000	-1.268260	-0.676665
H	0.000000	-2.153722	-0.037635
H	-0.879028	-1.314318	-1.330484
H	0.879028	-1.314318	-1.330484
C	0.000000	1.268260	-0.676665
H	0.000000	2.153722	-0.037635
H	-0.879028	1.314318	-1.330484
H	0.879028	1.314318	-1.330484

(Hartree/Particle)
HF(PBE1PBE)= -157.0682898
Zero-point correction= 0.107867 (Hartree/Particle)
Thermal correction to Energy= 0.113170
Thermal correction to Enthalpy= 0.114115
Thermal correction to Gibbs Free Energy= 0.081228
Sum of electronic and zero-point Energies= -156.960423
Sum of electronic and thermal Energies= -156.955119
Sum of electronic and thermal Enthalpies= -156.954175
Sum of electronic and thermal Free Energies= -156.987062

[PMe_3] Derivatives

[PMe_3]

P	-0.000116	-0.000792	-0.606479
C	-0.634908	-1.494855	0.277098
C	-0.977088	1.295952	0.276602
H	-0.048030	-2.370221	-0.013646
H	-1.672237	-1.680268	-0.014088
H	-0.587398	-1.382241	1.365981
H	-0.618876	2.287053	-0.014294

H	-0.904074	1.198405	1.365532
H	-2.028420	1.225331	-0.014835
C	1.611217	0.197013	0.276848
H	2.290543	-0.608638	-0.014123
H	1.490314	0.182451	1.365787
H	2.075621	1.142875	-0.014538

(Hartree/Particle)
HF(PBE1PBE)= -460.8577506
Zero-point correction= 0.112665
Thermal correction to Energy= 0.119350
Thermal correction to Enthalpy= 0.120294
Thermal correction to Gibbs Free Energy= 0.083388
Sum of electronic and zero-point Energies= -460.745085
Sum of electronic and thermal Energies= -460.738401
Sum of electronic and thermal Enthalpies= -460.737457
Sum of electronic and thermal Free Energies= -460.774363

$[PMe_3]^{+\cdot}$

P	-0.000058	0.000022	-0.329952
C	-1.718590	0.210010	0.149142
C	1.041220	1.383298	0.148907
H	-2.316417	-0.615313	-0.243653
H	-2.100055	1.155071	-0.243529
H	-1.791753	0.218882	1.243783
H	2.050396	1.240891	-0.243688
H	1.085448	1.442431	1.243540
H	0.625553	2.313686	-0.244084
C	0.677442	-1.593338	0.148846
H	0.049521	-2.396184	-0.243579
H	0.706802	-1.661148	1.243478
H	1.690947	-1.698467	-0.244348

(Hartree/Particle)
HF(PBE1PBE)= -460.5752676
Zero-point correction= 0.112344
Thermal correction to Energy= 0.119398
Thermal correction to Enthalpy= 0.120342
Thermal correction to Gibbs Free Energy= 0.081616
Sum of electronic and zero-point Energies= -460.462924
Sum of electronic and thermal Energies= -460.455870
Sum of electronic and thermal Enthalpies= -460.454925
Sum of electronic and thermal Free Energies= -460.493652

PBE1PBE Frequency Analysis of Distorted Geometries

Cartesian coordinates, enthalpies and Gibbs energies for fragments produced by deletion of atoms from PBE1PBE/6-311++G(d,p) optimized $[R_2PPMe_3]^+$ cations in the gas phase (298 K) absence of a counterion or solvent, and single point energies subsequently calculated at the MP2/6-311++G(d,p) level. Fragments are listed in the order: $[R_2PPMe_2]^{+\cdot}$, $[R_2PPMe_2]$, $[RPPMe_3]^{+\cdot}$, $[RPPMe_3]$, $[RPPMe_2]^+$, $[PMe_3]^{+\cdot}$, $[PMe_3]$, $[R_2P]^+$, $[R_2P]^{\cdot}$, $[R]^+$, $[R]^{\cdot}$, $[Me]^+$, and $[Me]^{\cdot}$. Within each subsection the structures are in the order: R = Me, Et, ^{i}Pr, ^{t}Bu, Ph, N^{i}Pr$_2$. The quoted HF energies come from the PBE1PBE /6-311++G(d,p) frequency analysis as well as the later MP2 single point calculation. Structures of $[PMe_3]^{+\bullet}$, PMe_3, [Me]+, and $[Me]^{\cdot}$ are listed by the parent phosphinophosphonium from which they are derived.

$[R_2PPMe_2]^{+\cdot}$

$[Me_2PPMe_2]^{+\cdot}$

P	0.85097	0.00000	0.01506
P	-1.23634	0.00000	-0.70761
C	1.66568	1.46468	-0.66151
C	-1.88918	1.43061	0.26018
C	-1.88918	-1.43061	0.26018
H	1.22779	2.37108	-0.23824
H	1.54566	1.48245	-1.74699
H	2.73056	1.43884	-0.41681
C	1.07200	0.00000	1.81251
H	0.61406	0.89046	2.24788
H	2.13988	0.00000	2.04776
H	0.61406	-0.89046	2.24788
H	-1.45310	2.36629	-0.09637
H	-1.75812	1.34161	1.34101
H	-2.96156	1.48195	0.05027
H	-1.45310	-2.36629	-0.09637
H	-2.96156	-1.48195	0.05027
H	-1.75812	-1.34161	1.34101

(Hartree/Particle)
HF(PBE1PBE) = -841.6880497
Zero-point correction= 0.151397
Thermal correction to Energy= 0.161510
Thermal correction to Enthalpy= 0.162454
Thermal correction to Gibbs Free Energy= 0.115736
Sum of electronic and zero-point Energies= -841.536652
Sum of electronic and thermal Energies= -841.526540
Sum of electronic and thermal Enthalpies= -841.525595
Sum of electronic and thermal Free Energies= -841.572314

$[Et_2PPMe_2]^{+\cdot}$

P	-1.24811	-0.33414	0.01552
P	0.81134	0.07359	-0.68763
C	-2.40936	0.62714	-0.98402
C	1.69400	-1.28612	0.22983
C	1.24033	1.55788	0.34736
H	-2.24972	1.69612	-0.83483
H	-3.43430	0.37314	-0.70156
H	-2.25472	0.39284	-2.03947
C	-1.56567	0.01771	1.76418
H	-0.90263	-0.58062	2.39263
H	-2.60305	-0.23028	2.00471
H	-1.39581	1.07628	1.97062
H	1.33069	-2.23668	-0.17257
H	1.46806	-1.26334	1.30149
C	3.20235	-1.18581	0.00201
C	0.51950	2.84134	-0.04640
H	2.31540	1.67877	0.17294
H	1.12571	1.33827	1.41420
H	3.44889	-1.18374	-1.06315
H	3.70126	-2.04469	0.45673
H	3.62795	-0.28696	0.45383
H	0.59394	3.03497	-1.11963
H	0.96225	3.69360	0.47420
H	-0.54103	2.82456	0.22238

(Hartree/Particle)
HF(PBE1PBE) = -920.2386641
Zero-point correction= 0.208805
Thermal correction to Energy= 0.221714
Thermal correction to Enthalpy= 0.222659
Thermal correction to Gibbs Free Energy= 0.168529
Sum of electronic and zero-point Energies= -920.029860

Sum of electronic and thermal Energies= -920.016950
Sum of electronic and thermal Enthalpies= -920.016005
Sum of electronic and thermal Free Energies= -920.070135

[^{i}Pr$_2$PPMe$_2$]$^{+\cdot}$

P	-1.56442	-0.08849	-0.03739
P	0.57938	0.05617	-0.61869
C	-2.45197	1.11498	-1.06349
C	1.48498	-1.33404	0.28827
C	1.13027	1.53929	0.40782
H	-2.13840	2.13629	-0.85385
H	-3.52384	1.01878	-0.87043
H	-2.25430	0.89418	-2.11480
C	-1.99494	0.21462	1.69868
H	-1.52821	-0.53845	2.33643
H	-3.08042	0.16638	1.82174
H	-1.64516	1.20294	2.00312
C	0.67599	-2.56309	0.68068
H	1.87758	-0.87390	1.20224
C	2.65336	-1.74226	-0.61473
C	0.38514	2.82584	0.06805
C	2.63110	1.72028	0.16373
H	0.97729	1.29369	1.46590
H	2.29156	-2.25009	-1.51345
H	3.30820	-2.43361	-0.07568
H	3.26098	-0.89395	-0.93796
H	0.37811	3.01971	-1.00901
H	0.88750	3.67101	0.54724
H	-0.64550	2.83189	0.43161
H	-0.10151	-2.34453	1.41824
H	1.34736	-3.29523	1.13904
H	0.22355	-3.05011	-0.18766
H	2.84281	1.91601	-0.89211
H	3.22232	0.85934	0.48122
H	2.98267	2.58264	0.73744

(Hartree/Particle)
HF(PBE1PBE) = -998.7838198
Zero-point correction= 0.265289
Thermal correction to Energy= 0.280946
Thermal correction to Enthalpy= 0.281891
Thermal correction to Gibbs Free Energy= 0.221300
Sum of electronic and zero-point Energies= -998.518530
Sum of electronic and thermal Energies= -998.502873
Sum of electronic and thermal Enthalpies= -998.501929
Sum of electronic and thermal Free Energies= -998.562520

[^{t}Bu$_2$PPMe$_2$]$^{+\cdot}$

P	-1.73709	-0.00138	-0.01480
P	0.36932	0.00201	-0.75905
C	-2.52140	1.61025	-0.30328
C	1.12198	-1.57863	0.00626
C	1.12915	1.57872	0.00746
C	-2.11470	-0.43935	1.70940
H	-2.38224	1.91399	-1.34227
H	-2.12442	2.37822	0.35974
H	-3.59155	1.49430	-0.10928
H	-1.80503	-1.46324	1.92156
H	-3.19467	-0.35686	1.86281
H	-1.60125	0.23888	2.39209
C	0.70192	2.73585	-0.90655
C	2.65273	1.44777	-0.12025
C	0.76095	1.88543	1.45834
C	0.16841	-2.74296	-0.28058
C	1.44023	-1.53849	1.49884
C	2.40295	-1.83760	-0.80463
H	-0.30361	2.10463	1.58154
H	1.30160	2.78163	1.78262
H	1.02961	1.07817	2.14121
H	0.99231	2.55549	-1.94473
H	1.20294	3.65106	-0.57355
H	-0.37051	2.93244	-0.88480
H	2.96197	1.19444	-1.13845
H	3.07795	0.71878	0.57063
H	3.09680	2.41971	0.11940
H	2.19520	-1.90566	-1.87603
H	2.82576	-2.79613	-0.48512
H	3.16856	-1.07758	-0.65106
H	-0.71527	-2.73353	0.36388
H	0.69256	-3.68159	-0.07364
H	-0.14687	-2.77365	-1.32796
H	0.55728	-1.34689	2.11337
H	2.19709	-0.79317	1.74689
H	1.84118	-2.51286	1.80021

(Hartree/Particle)
HF(PBE1PBE) = -1077.330017
Zero-point correction= 0.321791
Thermal correction to Energy= 0.340373
Thermal correction to Enthalpy= 0.341317
Thermal correction to Gibbs Free Energy= 0.277449
Sum of electronic and zero-point Energies= -1077.008226
Sum of electronic and thermal Energies= -1076.989644
Sum of electronic and thermal Enthalpies= -1076.988700
Sum of electronic and thermal Free Energies= -1077.052568

[Ph$_2$PPMe$_2$]$^{+\cdot}$

P	0.14756	2.01961	-0.24131
P	0.00471	0.23269	1.07299
C	0.50191	1.73828	-1.99565
C	1.48376	3.05322	0.40551
H	-0.37926	1.34082	-2.50049
H	1.32893	1.03041	-2.08949
H	0.78249	2.68644	-2.46255
H	1.32011	3.24878	1.46715
H	1.50949	4.00044	-0.13932
H	2.43987	2.54150	0.27620
H	4.79469	-0.75438	0.95634
C	3.82977	-1.04084	0.55204
C	2.68674	-0.35636	0.94350
H	2.76705	0.44071	1.67800
C	1.43370	-0.71769	0.43096
C	1.33899	-1.80790	-0.43727
H	0.37422	-2.14111	-0.80080
C	2.48519	-2.50031	-0.81063
H	2.40162	-3.35372	-1.47508
C	3.73014	-2.11181	-0.32940
H	4.62029	-2.65731	-0.62346
H	-2.31310	-0.53529	2.42630
C	-2.51082	-0.76671	1.38398
C	-1.53193	-0.51027	0.41718
C	-1.80372	-0.81510	-0.92354
H	-1.05845	-0.64345	-1.69243
C	-3.02746	-1.36261	-1.28292
H	-3.22825	-1.59806	-2.32274
C	-3.99232	-1.61647	-0.31170
H	-4.94576	-2.04826	-0.59658
C	-3.73290	-1.32120	1.02007
H	-4.48014	-1.52205	1.77997

(Hartree/Particle)
HF(PBE1PBE) = -1224.801318
Zero-point correction= 0.259342
Thermal correction to Energy= 0.274567
Thermal correction to Enthalpy= 0.275512
Thermal correction to Gibbs Free Energy= 0.215782
Sum of electronic and zero-point Energies= -1224.541975

Sum of electronic and thermal Energies= -1224.526750
Sum of electronic and thermal Enthalpies= -1224.525806
Sum of electronic and thermal Free Energies= -1224.585536

[(N^{i}Pr$_2$)$_2$PPMe$_2$]$^{+\cdot}$

P	-0.27148	-2.25296	-0.26843
N	1.29006	0.44650	-0.22150
C	0.11745	-3.18462	1.25253
C	-1.92739	-2.80838	-0.75913
C	1.92411	0.23923	1.09827
C	1.95976	1.22563	-1.30905
C	2.52002	1.49758	1.72544
C	3.39530	0.80026	-1.58901
C	1.84010	2.74066	-1.15052
H	1.38442	0.96629	-2.20617
H	2.79470	1.28407	2.76254
H	1.82224	2.33489	1.72326
H	3.43187	1.80942	1.21175
H	4.08463	1.09604	-0.79498
H	3.73349	1.29441	-2.50386
H	3.47793	-0.27830	-1.74211
H	2.48553	3.13606	-0.36511
H	0.81031	3.03226	-0.92802
H	2.13037	3.22574	-2.08723
H	0.01415	1.57342	2.49097
H	-0.71407	-0.86034	2.46516
C	-1.00193	1.90431	2.27567
H	-2.78531	3.10499	0.44828
H	-1.46116	2.17881	3.22987
H	-0.94663	2.80055	1.65646
C	-1.74075	-0.48834	2.40246
N	-1.57332	0.62651	0.16173
H	-2.69358	-0.28572	-2.25166
C	-2.68174	2.74860	-0.57823
C	-1.82686	0.80649	1.61150
H	-2.07318	-0.31205	3.42893
H	-3.52501	3.14858	-1.14792
H	-2.38287	-1.26455	1.97694
C	-2.69032	0.79988	-2.13103
H	-1.76188	3.16629	-0.99930
C	-2.67099	1.22643	-0.67114
H	-1.84971	1.20434	-2.69969
H	-3.61016	1.18556	-2.57881
H	-3.59720	0.84604	-0.22033
P	-0.26632	-0.00981	-0.68760
H	-2.87400	1.12372	1.65422
H	1.05280	-2.84123	1.69453
H	0.21404	-4.24270	0.99160
H	-0.68753	-3.07004	1.97803
H	-1.97767	-3.89929	-0.71479
H	-2.13640	-2.48320	-1.77969
H	-2.68016	-2.38503	-0.09205
C	2.97338	-0.87685	1.10832
H	2.62561	-1.77514	0.59428
H	3.20769	-1.14850	2.14246
H	3.90643	-0.56894	0.63568
H	1.10803	-0.08774	1.75078

(Hartree/Particle)
HF(PBE1PBE) = -1345.0332885
Zero-point correction= 0.469425
Thermal correction to Energy= 0.495114
Thermal correction to Enthalpy= 0.496058
Thermal correction to Gibbs Free Energy= 0.414900
Sum of electronic and zero-point Energies= -1344.563864
Sum of electronic and thermal Energies= -1344.538174
Sum of electronic and thermal Enthalpies= -1344.537230
Sum of electronic and thermal Free Energies= -1344.618389

[R$_2$PPMe$_2$]

[Me$_2$PPMe$_2$]

P	0.85097	0.00000	0.01506
P	-1.23634	0.00000	-0.70761
C	1.66568	1.46468	-0.66151
C	-1.88918	1.43061	0.26018
C	-1.88918	-1.43061	0.26018
H	1.22779	2.37108	-0.23824
H	1.54566	1.48245	-1.74699
H	2.73056	1.43884	-0.41681
C	1.07200	0.00000	1.81251
H	0.61406	0.89046	2.24788
H	2.13988	0.00000	2.04776
H	0.61406	-0.89046	2.24788
H	-1.45310	2.36629	-0.09637
H	-1.75812	1.34161	1.34101
H	-2.96156	1.48195	0.05027
H	-1.45310	-2.36629	-0.09637
H	-2.96156	-1.48195	0.05027
H	-1.75812	-1.34161	1.34101

(Hartree/Particle)
HF(PBE1PBE) = -841.9521241
Zero-point correction= 0.151751
Thermal correction to Energy= 0.161786
Thermal correction to Enthalpy= 0.162730
Thermal correction to Gibbs Free Energy= 0.116772
Sum of electronic and zero-point Energies= -841.800373
Sum of electronic and thermal Energies= -841.790338
Sum of electronic and thermal Enthalpies= -841.789394
Sum of electronic and thermal Free Energies= -841.835352

[Et$_2$PPMe$_2$]

P	-1.24811	-0.33414	0.01552
P	0.81134	0.07359	-0.68763
C	-2.40936	0.62714	-0.98402
C	1.69400	-1.28612	0.22983
C	1.24033	1.55788	0.34736
H	-2.24972	1.69612	-0.83483
H	-3.43430	0.37314	-0.70156
H	-2.25472	0.39284	-2.03947
C	-1.56567	0.01771	1.76418
H	-0.90263	-0.58062	2.39263
H	-2.60305	-0.23028	2.00471
H	-1.39581	1.07628	1.97062
H	1.33069	-2.23668	-0.17257
H	1.46806	-1.26334	1.30149
C	3.20235	-1.18581	0.00201
C	0.51950	2.84134	-0.04640
H	2.31540	1.67877	0.17294
H	1.12571	1.33827	1.41420
H	3.44889	-1.18374	-1.06315
H	3.70126	-2.04469	0.45673
H	3.62795	-0.28696	0.45383
H	0.59394	3.03497	-1.11963
H	0.96225	3.69360	0.47420
H	-0.54103	2.82456	0.22238

(Hartree/Particle)
HF(PBE1PBE) = -920.4991501
Zero-point correction= 0.209382
Thermal correction to Energy= 0.222117
Thermal correction to Enthalpy= 0.223061
Thermal correction to Gibbs Free Energy= 0.170341
Sum of electronic and zero-point Energies= -920.289768
Sum of electronic and thermal Energies= -920.277033

Sum of electronic and thermal Enthalpies= -920.276089
Sum of electronic and thermal Free Energies= -920.328809

[^{i}Pr$_2$PPMe$_2$]

P	-1.56442	-0.08849	-0.03739
P	0.57938	0.05617	-0.61869
C	-2.45197	1.11498	-1.06349
C	1.48498	-1.33404	0.28827
C	1.13027	1.53929	0.40782
H	-2.13840	2.13629	-0.85385
H	-3.52384	1.01878	-0.87043
H	-2.25430	0.89418	-2.11480
C	-1.99494	0.21462	1.69868
H	-1.52821	-0.53845	2.33643
H	-3.08042	0.16638	1.82174
H	-1.64516	1.20294	2.00312
C	0.67599	-2.56309	0.68068
H	1.87758	-0.87390	1.20224
C	2.65336	-1.74226	-0.61473
C	0.38514	2.82584	0.06805
C	2.63110	1.72028	0.16373
H	0.97729	1.29369	1.46590
H	2.29156	-2.25009	-1.51345
H	3.30820	-2.43361	-0.07568
H	3.26098	-0.89395	-0.93796
H	0.37811	3.01971	-1.00901
H	0.88750	3.67101	0.54724
H	-0.64550	2.83189	0.43161
H	-0.10151	-2.34453	1.41824
H	1.34736	-3.29523	1.13904
H	0.22355	-3.05011	-0.18766
H	2.84281	1.91601	-0.89211
H	3.22232	0.85934	0.48122
H	2.98267	2.58264	0.73744

(Hartree/Particle)
HF(PBE1PBE) = -999.0408019
Zero-point correction= 0.265722
Thermal correction to Energy= 0.280223
Thermal correction to Enthalpy= 0.281167
Thermal correction to Gibbs Free Energy= 0.225582
Sum of electronic and zero-point Energies= -998.775080
Sum of electronic and thermal Energies= -998.760579
Sum of electronic and thermal Enthalpies= -998.759635
Sum of electronic and thermal Free Energies= -998.815220

[^{t}Bu$_2$PPMe$_2$]

P	-1.73709	-0.00138	-0.01480
P	0.36932	0.00201	-0.75905
C	-2.52140	1.61025	-0.30328
C	1.12198	-1.57863	0.00626
C	1.12915	1.57872	0.00746
C	-2.11470	-0.43935	1.70940
H	-2.38224	1.91399	-1.34227
H	-2.12442	2.37822	0.35974
H	-3.59155	1.49430	-0.10928
H	-1.80503	-1.46324	1.92156
H	-3.19467	-0.35686	1.86281
H	-1.60125	0.23888	2.39209
C	0.70192	2.73585	-0.90655
C	2.65273	1.44777	-0.12025
C	0.76095	1.88543	1.45834
C	0.16841	-2.74296	-0.28058
C	1.44023	-1.53849	1.49884
C	2.40295	-1.83760	-0.80463
H	-0.30361	2.10463	1.58154
H	1.30160	2.78163	1.78262
H	1.02961	1.07817	2.14121
H	0.99231	2.55549	-1.94473
H	1.20294	3.65106	-0.57355
H	-0.37051	2.93244	-0.88480
H	2.96197	1.19444	-1.13845
H	3.07795	0.71878	0.57063
H	3.09680	2.41971	0.11940
H	2.19520	-1.90566	-1.87603
H	2.82576	-2.79613	-0.48512
H	3.16856	-1.07758	-0.65106
H	-0.71527	-2.73353	0.36388
H	0.69256	-3.68159	-0.07364
H	-0.14687	-2.77365	-1.32796
H	0.55728	-1.34689	2.11337
H	2.19709	-0.79317	1.74689
H	1.84118	-2.51286	1.80021

(Hartree/Particle)
HF(PBE1PBE) = -1077.577603
Zero-point correction= 0.321862
Thermal correction to Energy= 0.339499
Thermal correction to Enthalpy= 0.340443
Thermal correction to Gibbs Free Energy= 0.279588
Sum of electronic and zero-point Energies= -1077.255741
Sum of electronic and thermal Energies= -1077.238104
Sum of electronic and thermal Enthalpies= -1077.237160
Sum of electronic and thermal Free Energies= -1077.298015

[Ph$_2$PPMe$_2$]

P	0.14756	2.01961	-0.24131
P	0.00471	0.23269	1.07299
C	0.50191	1.73828	-1.99565
C	1.48376	3.05322	0.40551
H	-0.37926	1.34082	-2.50049
H	1.32893	1.03041	-2.08949
H	0.78249	2.68644	-2.46255
H	1.32011	3.24878	1.46715
H	1.50949	4.00044	-0.13932
H	2.43987	2.54150	0.27620
H	4.79469	-0.75438	0.95634
C	3.82977	-1.04084	0.55204
C	2.68674	-0.35636	0.94350
H	2.76705	0.44071	1.67800
C	1.43370	-0.71769	0.43096
C	1.33899	-1.80790	-0.43727
H	0.37422	-2.14111	-0.80080
C	2.48519	-2.50031	-0.81063
H	2.40162	-3.35372	-1.47508
C	3.73014	-2.11181	-0.32940
H	4.62029	-2.65731	-0.62346
H	-2.31310	-0.53529	2.42630
C	-2.51082	-0.76671	1.38398
C	-1.53193	-0.51027	0.41718
C	-1.80372	-0.81510	-0.92354
H	-1.05845	-0.64345	-1.69243
C	-3.02746	-1.36261	-1.28292
H	-3.22825	-1.59806	-2.32274
C	-3.99232	-1.61647	-0.31170
H	-4.94576	-2.04826	-0.59658
C	-3.73290	-1.32120	1.02007
H	-4.48014	-1.52205	1.77997

(Hartree/Particle)
HF(PBE1PBE) = -1225.0540164
Zero-point correction= 0.259614
Thermal correction to Energy= 0.274686
Thermal correction to Enthalpy= 0.275630
Thermal correction to Gibbs Free Energy= 0.216684
Sum of electronic and zero-point Energies= -1224.794402
Sum of electronic and thermal Energies= -1224.779330

Sum of electronic and thermal Enthalpies= -1224.778386
Sum of electronic and thermal Free Energies= -1224.837333

[(N^{i}Pr$_2$)$_2$PPMe$_2$]

P	-0.27148	-2.25296	-0.26843
N	1.29006	0.44650	-0.22150
C	0.11745	-3.18462	1.25253
C	-1.92739	-2.80838	-0.75913
C	1.92411	0.23923	1.09827
C	1.95976	1.22563	-1.30905
C	2.52002	1.49758	1.72544
C	3.39530	0.80026	-1.58901
C	1.84010	2.74066	-1.15052
H	1.38442	0.96629	-2.20617
H	2.79470	1.28407	2.76254
H	1.82224	2.33489	1.72326
H	3.43187	1.80942	1.21175
H	4.08463	1.09604	-0.79498
H	3.73349	1.29441	-2.50386
H	3.47793	-0.27830	-1.74211
H	2.48553	3.13606	-0.36511
H	0.81031	3.03226	-0.92802
H	2.13037	3.22574	-2.08723
H	0.01415	1.57342	2.49097
H	-0.71407	-0.86034	2.46516
C	-1.00193	1.90431	2.27567
H	-2.78531	3.10499	0.44828
H	-1.46116	2.17881	3.22987
H	-0.94663	2.80055	1.65646
C	-1.74075	-0.48834	2.40246
N	-1.57332	0.62651	0.16173
H	-2.69358	-0.28572	-2.25166
C	-2.68174	2.74860	-0.57823
C	-1.82686	0.80649	1.61150
H	-2.07318	-0.31205	3.42893
H	-3.52501	3.14858	-1.14792
H	-2.38287	-1.26455	1.97694
C	-2.69032	0.79988	-2.13103
H	-1.76188	3.16629	-0.99930
C	-2.67099	1.22643	-0.67114
H	-1.84971	1.20434	-2.69969
H	-3.61016	1.18556	-2.57881
H	-3.59720	0.84604	-0.22033
P	-0.26632	-0.00981	-0.68760
H	-2.87400	1.12372	1.65422
H	1.05280	-2.84123	1.69453
H	0.21404	-4.24270	0.99160
H	-0.68753	-3.07004	1.97803
H	-1.97767	-3.89929	-0.71479
H	-2.13640	-2.48320	-1.77969
H	-2.68016	-2.38503	-0.09205
C	2.97338	-0.87685	1.10832
H	2.62561	-1.77514	0.59428
H	3.20769	-1.14850	2.14246
H	3.90643	-0.56894	0.63568
H	1.10803	-0.08774	1.75078

(Hartree/Particle)
HF(PBE1PBE) = -1345.2608808
Zero-point correction= 0.469103
Thermal correction to Energy= 0.493886
Thermal correction to Enthalpy= 0.494830
Thermal correction to Gibbs Free Energy= 0.417072
Sum of electronic and zero-point Energies= -1344.791778
Sum of electronic and thermal Energies= -1344.766995
Sum of electronic and thermal Enthalpies= -1344.766050
Sum of electronic and thermal Free Energies= -1344.843809

[RPPMe$_3$]$^{+\cdot}$

[MePPMe$_3$]$^{+\cdot}$

P	0.85097	0.00000	0.01506
P	-1.23634	0.00000	-0.70761
C	1.66568	1.46468	-0.66151
C	1.66568	-1.46468	-0.66151
C	-1.88918	-1.43061	0.26018
H	1.22779	2.37108	-0.23824
H	1.54566	1.48245	-1.74699
H	2.73056	1.43884	-0.41681
H	1.22779	-2.37108	-0.23824
H	2.73056	-1.43884	-0.41681
H	1.54566	-1.48245	-1.74699
C	1.07200	0.00000	1.81251
H	0.61406	0.89046	2.24788
H	2.13988	0.00000	2.04776
H	0.61406	-0.89046	2.24788
H	-1.45310	-2.36629	-0.09637
H	-2.96156	-1.48195	0.05027
H	-1.75812	-1.34161	1.34101

(Hartree/Particle)
HF(PBE1PBE) = -841.7190088
Zero-point correction= 0.152407
Thermal correction to Energy= 0.162222
Thermal correction to Enthalpy= 0.163166
Thermal correction to Gibbs Free Energy= 0.117489
Sum of electronic and zero-point Energies= -841.566601
Sum of electronic and thermal Energies= -841.556787
Sum of electronic and thermal Enthalpies= -841.555843
Sum of electronic and thermal Free Energies= -841.601519

[EtPPMe$_3$]$^{+\cdot}$

P	-1.24811	-0.33414	0.01552
P	0.81134	0.07359	-0.68763
C	-1.60986	-2.08182	-0.28235
C	-2.40936	0.62714	-0.98402
C	1.24033	1.55788	0.34736
H	-1.01704	-2.71303	0.38192
H	-1.38120	-2.33544	-1.32009
H	-2.67090	-2.26749	-0.09641
H	-2.24972	1.69612	-0.83483
H	-3.43430	0.37314	-0.70156
H	-2.25472	0.39284	-2.03947
C	-1.56567	0.01771	1.76418
H	-0.90263	-0.58062	2.39263
H	-2.60305	-0.23028	2.00471
H	-1.39581	1.07628	1.97062
C	0.51950	2.84134	-0.04640
H	2.31540	1.67877	0.17294
H	1.12571	1.33827	1.41420
H	0.59394	3.03497	-1.11963
H	0.96225	3.69360	0.47420
H	-0.54103	2.82456	0.22238

(Hartree/Particle)
HF(PBE1PBE) = -880.993183
Zero-point correction= 0.181756
Thermal correction to Energy= 0.193567
Thermal correction to Enthalpy= 0.194511
Thermal correction to Gibbs Free Energy= 0.143750
Sum of electronic and zero-point Energies= -880.811427
Sum of electronic and thermal Energies= -880.799616
Sum of electronic and thermal Enthalpies= -880.798672
Sum of electronic and thermal Free Energies= -880.849433

[^{i}PrPPMe$_3$]$^{+\cdot}$

P	-1.56442	-0.08849	-0.03739
P	0.57938	0.05617	-0.61869
C	-2.27965	-1.68158	-0.52655
C	-2.45197	1.11498	-1.06349
C	1.13027	1.53929	0.40782
H	-2.00461	-2.47431	0.16657
H	-1.94034	-1.94372	-1.53113
H	-3.36775	-1.57446	-0.53831
H	-2.13840	2.13629	-0.85385
H	-3.52384	1.01878	-0.87043
H	-2.25430	0.89418	-2.11480
C	-1.99494	0.21462	1.69868
H	-1.52821	-0.53845	2.33643
H	-3.08042	0.16638	1.82174
H	-1.64516	1.20294	2.00312
C	0.38514	2.82584	0.06805
C	2.63110	1.72028	0.16373
H	0.97729	1.29369	1.46590
H	0.37811	3.01971	-1.00901
H	0.88750	3.67101	0.54724
H	-0.64550	2.83189	0.43161
H	2.84281	1.91601	-0.89211
H	3.22232	0.85934	0.48122
H	2.98267	2.58264	0.73744

(Hartree/Particle)
HF(PBE1PBE) = -920.2662708
Zero-point correction= 0.209865
Thermal correction to Energy= 0.223111
Thermal correction to Enthalpy= 0.224056
Thermal correction to Gibbs Free Energy= 0.170197
Sum of electronic and zero-point Energies= -920.056406
Sum of electronic and thermal Energies= -920.043159
Sum of electronic and thermal Enthalpies= -920.042215
Sum of electronic and thermal Free Energies= -920.096074

[^{t}BuPPMe$_3$]$^{+\cdot}$

P	-1.73709	-0.00138	-0.01480
P	0.36932	0.00201	-0.75905
C	-2.52140	1.61025	-0.30328
C	1.12198	-1.57863	0.00626
C	-2.11470	-0.43935	1.70940
H	-2.38224	1.91399	-1.34227
H	-2.12442	2.37822	0.35974
H	-3.59155	1.49430	-0.10928
H	-1.80503	-1.46324	1.92156
H	-3.19467	-0.35686	1.86281
H	-1.60125	0.23888	2.39209
C	-2.69309	-1.12256	-1.07493
H	-2.50399	-0.87215	-2.12085
H	-3.75552	-0.98500	-0.85634
H	-2.42403	-2.16346	-0.90460
C	0.16841	-2.74296	-0.28058
C	1.44023	-1.53849	1.49884
C	2.40295	-1.83760	-0.80463
H	2.19520	-1.90566	-1.87603
H	2.82576	-2.79613	-0.48512
H	3.16856	-1.07758	-0.65106
H	-0.71527	-2.73353	0.36388
H	0.69256	-3.68159	-0.07364
H	-0.14687	-2.77365	-1.32796
H	0.55728	-1.34689	2.11337
H	2.19709	-0.79317	1.74689
H	1.84118	-2.51286	1.80021

(Hartree/Particle)
HF(PBE1PBE) = -959.5383353
Zero-point correction= 0.237943
Thermal correction to Energy= 0.252371
Thermal correction to Enthalpy= 0.253315
Thermal correction to Gibbs Free Energy= 0.197728
Sum of electronic and zero-point Energies= -959.300393
Sum of electronic and thermal Energies= -959.285965
Sum of electronic and thermal Enthalpies= -959.285021
Sum of electronic and thermal Free Energies= -959.340607

[PhPPMe$_3$]$^{+\cdot}$

P	0.14756	2.01961	-0.24131
P	0.00471	0.23269	1.07299
C	0.50191	1.73828	-1.99565
C	1.48376	3.05322	0.40551
H	-0.37926	1.34082	-2.50049
H	1.32893	1.03041	-2.08949
H	0.78249	2.68644	-2.46255
H	1.32011	3.24878	1.46715
H	1.50949	4.00044	-0.13932
H	2.43987	2.54150	0.27620
C	-1.40208	2.93768	-0.09199
H	-2.22583	2.32881	-0.47034
H	-1.33885	3.86617	-0.66497
H	-1.58712	3.17363	0.95831
H	-2.31310	-0.53529	2.42630
C	-2.51082	-0.76671	1.38398
C	-1.53193	-0.51027	0.41718
C	-1.80372	-0.81510	-0.92354
H	-1.05845	-0.64345	-1.69243
C	-3.02746	-1.36261	-1.28292
H	-3.22825	-1.59806	-2.32274
C	-3.99232	-1.61647	-0.31170
H	-4.94576	-2.04826	-0.59658
C	-3.73290	-1.32120	1.02007
H	-4.48014	-1.52205	1.77997

(Hartree/Particle)
HF(PBE1PBE) = -1033.2762645
Zero-point correction= 0.206852
Thermal correction to Energy= 0.219461
Thermal correction to Enthalpy= 0.220406
Thermal correction to Gibbs Free Energy= 0.167244
Sum of electronic and zero-point Energies= -1033.069413
Sum of electronic and thermal Energies= -1033.056803
Sum of electronic and thermal Enthalpies= -1033.055859
Sum of electronic and thermal Free Energies= -1033.109020

[^{i}Pr$_2$NPPMe$_3$]$^{+\cdot}$

P	-0.27148	-2.25296	-0.26843
C	0.11746	-3.18462	1.25253
C	-1.92738	-2.80838	-0.75913
C	0.85546	-2.88707	-1.54352
H	0.60094	-2.43530	-2.50433
H	1.88795	-2.63273	-1.29977
H	0.01414	1.57343	2.49097
H	-0.71407	-0.86034	2.46516
C	-1.00193	1.90430	2.27567
H	-2.78532	3.10498	0.44828
H	-1.46117	2.17881	3.22987
H	-0.94663	2.80055	1.65646
C	-1.74075	-0.48834	2.40246
N	-1.57333	0.62651	0.16173
H	-2.69358	-0.28573	-2.25166
C	-2.68175	2.74860	-0.57823
C	-1.82686	0.80649	1.61150
H	-2.07318	-0.31205	3.42893
H	-3.52501	3.14858	-1.14792

H	-2.38287	-1.26456	1.97694
C	-2.69032	0.79988	-2.13103
H	-1.76188	3.16629	-0.99929
C	-2.67100	1.22643	-0.67114
H	-1.84971	1.20434	-2.69969
H	-3.61016	1.18556	-2.57881
H	-3.59720	0.84604	-0.22033
P	-0.26632	-0.00981	-0.68760
H	-2.87400	1.12372	1.65422
H	1.05281	-2.84123	1.69453
H	0.21405	-4.24270	0.99160
H	-0.68752	-3.07004	1.97803
H	-1.97767	-3.89929	-0.71480
H	-2.13640	-2.48320	-1.77969
H	-2.68016	-2.38503	-0.09205
H	0.75971	-3.97323	-1.61648

(Hartree/Particle)
HF(PBE1PBE) = -1093.3924475
Zero-point correction= 0.311484
Thermal correction to Energy= 0.329079
Thermal correction to Enthalpy= 0.330023
Thermal correction to Gibbs Free Energy= 0.266877
Sum of electronic and zero-point Energies= -1093.080963
Sum of electronic and thermal Energies= -1093.063368
Sum of electronic and thermal Enthalpies= -1093.062424
Sum of electronic and thermal Free Energies= -1093.125571

[RPPMe$_3$]

[MePPMe$_3$]

P	0.85097	0.00000	0.01506
P	-1.23634	0.00000	-0.70761
C	1.66568	1.46468	-0.66151
C	1.66568	-1.46468	-0.66151
C	-1.88918	-1.43061	0.26018
H	1.22779	2.37108	-0.23824
H	1.54566	1.48245	-1.74699
H	2.73056	1.43884	-0.41681
H	1.22779	-2.37108	-0.23824
H	2.73056	-1.43884	-0.41681
H	1.54566	-1.48245	-1.74699
C	1.07200	0.00000	1.81251
H	0.61406	0.89046	2.24788
H	2.13988	0.00000	2.04776
H	0.61406	-0.89046	2.24788
H	-1.45310	-2.36629	-0.09637
H	-2.96156	-1.48195	0.05027
H	-1.75812	-1.34161	1.34101

(Hartree/Particle)
HF(PBE1PBE) = -841.9356583
Zero-point correction= 0.152019
Thermal correction to Energy= 0.161950
Thermal correction to Enthalpy= 0.162894
Thermal correction to Gibbs Free Energy= 0.117460
Sum of electronic and zero-point Energies= -841.783640
Sum of electronic and thermal Energies= -841.773708
Sum of electronic and thermal Enthalpies= -841.772764
Sum of electronic and thermal Free Energies= -841.818198

[EtPPMe$_3$]

P	-1.24811	-0.33414	0.01552
P	0.81134	0.07359	-0.68763
C	-1.60986	-2.08182	-0.28235
C	-2.40936	0.62714	-0.98402
C	1.24033	1.55788	0.34736
H	-1.01704	-2.71303	0.38192
H	-1.38120	-2.33544	-1.32009
H	-2.67090	-2.26749	-0.09641
H	-2.24972	1.69612	-0.83483
H	-3.43430	0.37314	-0.70156
H	-2.25472	0.39284	-2.03947
C	-1.56567	0.01771	1.76418
H	-0.90263	-0.58062	2.39263
H	-2.60305	-0.23028	2.00471
H	-1.39581	1.07628	1.97062
C	0.51950	2.84134	-0.04640
H	2.31540	1.67877	0.17294
H	1.12571	1.33827	1.41420
H	0.59394	3.03497	-1.11963
H	0.96225	3.69360	0.47420
H	-0.54103	2.82456	0.22238

(Hartree/Particle)
Hf(PBE1PBE) = -881.2093913
Zero-point correction= 0.180922
Thermal correction to Energy= 0.191949
Thermal correction to Enthalpy= 0.192893
Thermal correction to Gibbs Free Energy= 0.145131
Sum of electronic and zero-point Energies= -881.028469
Sum of electronic and thermal Energies= -881.017442
Sum of electronic and thermal Enthalpies= -881.016498
Sum of electronic and thermal Free Energies= -881.064260

[^{i}PrPPMe$_3$]

P	-1.56442	-0.08849	-0.03739
P	0.57938	0.05617	-0.61869
C	-2.27965	-1.68158	-0.52655
C	-2.45197	1.11498	-1.06349
C	1.13027	1.53929	0.40782
H	-2.00461	-2.47431	0.16657
H	-1.94034	-1.94372	-1.53113
H	-3.36775	-1.57446	-0.53831
H	-2.13840	2.13629	-0.85385
H	-3.52384	1.01878	-0.87043
H	-2.25430	0.89418	-2.11480
C	-1.99494	0.21462	1.69868
H	-1.52821	-0.53845	2.33643
H	-3.08042	0.16638	1.82174
H	-1.64516	1.20294	2.00312
C	0.38514	2.82584	0.06805
C	2.63110	1.72028	0.16373
H	0.97729	1.29369	1.46590
H	0.37811	3.01971	-1.00901
H	0.88750	3.67101	0.54724
H	-0.64550	2.83189	0.43161
H	2.84281	1.91601	-0.89211
H	3.22232	0.85934	0.48122
H	2.98267	2.58264	0.73744

(Hartree/Particle)
HF(PBE1PBE) = -920.4811133
Zero-point correction= 0.209064
Thermal correction to Energy= 0.222436
Thermal correction to Enthalpy= 0.223380
Thermal correction to Gibbs Free Energy= 0.169735
Sum of electronic and zero-point Energies= -920.272049
Sum of electronic and thermal Energies= -920.258678
Sum of electronic and thermal Enthalpies= -920.257734
Sum of electronic and thermal Free Energies= -920.311378

[^{t}BuPPMe$_3$]

P	-1.73709	-0.00138	-0.01480

P	0.36932	0.00201	-0.75905
C	-2.52140	1.61025	-0.30328
C	1.12198	-1.57863	0.00626
C	-2.11470	-0.43935	1.70940
H	-2.38224	1.91399	-1.34227
H	-2.12442	2.37822	0.35974
H	-3.59155	1.49430	-0.10928
H	-1.80503	-1.46324	1.92156
H	-3.19467	-0.35686	1.86281
H	-1.60125	0.23888	2.39209
C	-2.69309	-1.12256	-1.07493
H	-2.50399	-0.87215	-2.12085
H	-3.75552	-0.98500	-0.85634
H	-2.42403	-2.16346	-0.90460
C	0.16841	-2.74296	-0.28058
C	1.44023	-1.53849	1.49884
C	2.40295	-1.83760	-0.80463
H	2.19520	-1.90566	-1.87603
H	2.82576	-2.79613	-0.48512
H	3.16856	-1.07758	-0.65106
H	-0.71527	-2.73353	0.36388
H	0.69256	-3.68159	-0.07364
H	-0.14687	-2.77365	-1.32796
H	0.55728	-1.34689	2.11337
H	2.19709	-0.79317	1.74689
H	1.84118	-2.51286	1.80021

(Hartree/Particle)
HF(PBE1PBE) = -959.752804
Zero-point correction= 0.237011
Thermal correction to Energy= 0.251584
Thermal correction to Enthalpy= 0.252528
Thermal correction to Gibbs Free Energy= 0.197256
Sum of electronic and zero-point Energies= -959.515793
Sum of electronic and thermal Energies= -959.501220
Sum of electronic and thermal Enthalpies= -959.500276
Sum of electronic and thermal Free Energies= -959.555548

[PhPPMe$_3$]

P	0.14756	2.01961	-0.24131
P	0.00471	0.23269	1.07299
C	0.50191	1.73828	-1.99565
C	1.48376	3.05322	0.40551
H	-0.37926	1.34082	-2.50049
H	1.32893	1.03041	-2.08949
H	0.78249	2.68644	-2.46255
H	1.32011	3.24878	1.46715
H	1.50949	4.00044	-0.13932
H	2.43987	2.54150	0.27620
C	-1.40208	2.93768	-0.09199
H	-2.22583	2.32881	-0.47034
H	-1.33885	3.86617	-0.66497
H	-1.58712	3.17363	0.95831
H	-2.31310	-0.53529	2.42630
C	-2.51082	-0.76671	1.38398
C	-1.53193	-0.51027	0.41718
C	-1.80372	-0.81510	-0.92354
H	-1.05845	-0.64345	-1.69243
C	-3.02746	-1.36261	-1.28292
H	-3.22825	-1.59806	-2.32274
C	-3.99232	-1.61647	-0.31170
H	-4.94576	-2.04826	-0.59658
C	-3.73290	-1.32120	1.02007
H	-4.48014	-1.52205	1.77997

(Hartree/Particle)
HF(PBE1PBE) = -1033.4943553
Zero-point correction= 0.206128
Thermal correction to Energy= 0.218815
Thermal correction to Enthalpy= 0.219759
Thermal correction to Gibbs Free Energy= 0.166943
Sum of electronic and zero-point Energies= -1033.288228
Sum of electronic and thermal Energies= -1033.275541
Sum of electronic and thermal Enthalpies= -1033.274597
Sum of electronic and thermal Free Energies= -1033.327412

[$^{i}Pr_2$NPPMe$_3$]

P	-0.27148	-2.25296	-0.26843
C	0.11746	-3.18462	1.25253
C	-1.92738	-2.80838	-0.75913
C	0.85546	-2.88707	-1.54352
H	0.60094	-2.43530	-2.50433
H	1.88795	-2.63273	-1.29977
H	0.01414	1.57343	2.49097
H	-0.71407	-0.86034	2.46516
C	-1.00193	1.90430	2.27567
H	-2.78532	3.10498	0.44828
H	-1.46117	2.17881	3.22987
H	-0.94663	2.80055	1.65646
C	-1.74075	-0.48834	2.40246
N	-1.57333	0.62651	0.16173
H	-2.69358	-0.28573	-2.25166
C	-2.68175	2.74860	-0.57823
C	-1.82686	0.80649	1.61150
H	-2.07318	-0.31205	3.42893
H	-3.52501	3.14858	-1.14792
H	-2.38287	-1.26456	1.97694
C	-2.69032	0.79988	-2.13103
H	-1.76188	3.16629	-0.99929
C	-2.67100	1.22643	-0.67114
H	-1.84971	1.20434	-2.69969
H	-3.61016	1.18556	-2.57881
H	-3.59720	0.84604	-0.22033
P	-0.26632	-0.00981	-0.68760
H	-2.87400	1.12372	1.65422
H	1.05281	-2.84123	1.69453
H	0.21405	-4.24270	0.99160
H	-0.68752	-3.07004	1.97803
H	-1.97767	-3.89929	-0.71480
H	-2.13640	-2.48320	-1.77969
H	-2.68016	-2.38503	-0.09205
H	0.75971	-3.97323	-1.61648

(Hartree/Particle)
HF(PBE1PBE) = -1093.5874348
Zero-point correction= 0.309480
Thermal correction to Energy= 0.326895
Thermal correction to Enthalpy= 0.327839
Thermal correction to Gibbs Free Energy= 0.265089
Sum of electronic and zero-point Energies= -1093.277955
Sum of electronic and thermal Energies= -1093.260540
Sum of electronic and thermal Enthalpies= -1093.259596
Sum of electronic and thermal Free Energies= -1093.322346

[PMe$_3$]$^{+\cdot}$

[Me$_2$PPMe$_3$]$^+$ parent phosphinophosphonium

P	0.85097	0.00000	0.01506
C	1.66568	1.46468	-0.66151
C	1.66568	-1.46468	-0.66151
H	1.22779	2.37108	-0.23824
H	1.54566	1.48245	-1.74699
H	2.73056	1.43884	-0.41681
H	1.22779	-2.37108	-0.23824

H	2.73056	-1.43884	-0.41681
H	1.54566	-1.48245	-1.74699
C	1.07200	0.00000	1.81251
H	0.61406	0.89046	2.24788
H	2.13988	0.00000	2.04776
H	0.61406	-0.89046	2.24788

(Hartree/Particle)
HF(PBE1PBE) = -460.5687625
Zero-point correction= 0.112076
Thermal correction to Energy= 0.119422
Thermal correction to Enthalpy= 0.120367
Thermal correction to Gibbs Free Energy= 0.079778
Sum of electronic and zero-point Energies= -460.456687
Sum of electronic and thermal Energies= -460.449340
Sum of electronic and thermal Enthalpies= -460.448396
Sum of electronic and thermal Free Energies= -460.488984

$[Et_2PPMe_3]^+$ parent phosphinophosphonium

C	-1.60986	-2.08182	-0.28235
C	-2.40936	0.62714	-0.98402
H	-1.01704	-2.71303	0.38192
H	-1.38120	-2.33544	-1.32009
H	-2.67090	-2.26749	-0.09641
H	-2.24972	1.69612	-0.83483
H	-3.43430	0.37314	-0.70156
H	-2.25472	0.39284	-2.03947
C	-1.56567	0.01771	1.76418
H	-0.90263	-0.58062	2.39263
H	-2.60305	-0.23028	2.00471
H	-1.39581	1.07628	1.97062

(Hartree/Particle)
HF(PBE1PBE) = -460.568071
Zero-point correction= 0.112015
Thermal correction to Energy= 0.118478
Thermal correction to Enthalpy= 0.119423
Thermal correction to Gibbs Free Energy= 0.081735
Sum of electronic and zero-point Energies= -460.456056
Sum of electronic and thermal Energies= -460.449593
Sum of electronic and thermal Enthalpies= -460.448648
Sum of electronic and thermal Free Energies= -460.486336

$[^iPr_2PPMe_3]^+$ parent phosphinophosphonium

P	-1.56442	-0.08849	-0.03739
C	-2.27965	-1.68158	-0.52655
C	-2.45197	1.11498	-1.06349
H	-2.00461	-2.47431	0.16657
H	-1.94034	-1.94372	-1.53113
H	-3.36775	-1.57446	-0.53831
H	-2.13840	2.13629	-0.85385
H	-3.52384	1.01878	-0.87043
H	-2.25430	0.89418	-2.11480
C	-1.99494	0.21462	1.69868
H	-1.52821	-0.53845	2.33643
H	-3.08042	0.16638	1.82174
H	-1.64516	1.20294	2.00312

(Hartree/Particle)
HF(PBE1PBE) = -460.5659019
Zero-point correction= 0.112003
Thermal correction to Energy= 0.118517
Thermal correction to Enthalpy= 0.119461
Thermal correction to Gibbs Free Energy= 0.081512
Sum of electronic and zero-point Energies= -460.453899
Sum of electronic and thermal Energies= -460.447385
Sum of electronic and thermal Enthalpies= -460.446441
Sum of electronic and thermal Free Energies= -460.484390

$[^tBu_2PPMe_3]^+$ parent phosphinophosphonium

P	-1.73709	-0.00138	-0.01480
C	-2.52140	1.61025	-0.30328
C	-2.11470	-0.43935	1.70940
H	-2.38224	1.91399	-1.34227
H	-2.12442	2.37822	0.35974
H	-3.59155	1.49430	-0.10928
H	-1.80503	-1.46324	1.92156
H	-3.19467	-0.35686	1.86281
H	-1.60125	0.23888	2.39209
C	-2.69309	-1.12256	-1.07493
H	-2.50399	-0.87215	-2.12085
H	-3.75552	-0.98500	-0.85634
H	-2.42403	-2.16346	-0.90460

(Hartree/Particle)
HF(PBE1PBE) = -460.563973
Zero-point correction= 0.112050
Thermal correction to Energy= 0.118532
Thermal correction to Enthalpy= 0.119476
Thermal correction to Gibbs Free Energy= 0.081646
Sum of electronic and zero-point Energies= -460.451923
Sum of electronic and thermal Energies= -460.445441
Sum of electronic and thermal Enthalpies= -460.444497
Sum of electronic and thermal Free Energies= -460.482327

$[Ph_2PPMe_3]^+$ parent phosphinophosphonium

P	0.14756	2.01961	-0.24131
C	0.50191	1.73828	-1.99565
C	1.48376	3.05322	0.40551
H	-0.37926	1.34082	-2.50049
H	1.32893	1.03041	-2.08949
H	0.78249	2.68644	-2.46255
H	1.32011	3.24878	1.46715
H	1.50949	4.00044	-0.13932
H	2.43987	2.54150	0.27620
C	-1.40208	2.93768	-0.09199
H	-2.22583	2.32881	-0.47034
H	-1.33885	3.86617	-0.66497
H	-1.58712	3.17363	0.95831

(Hartree/Particle)
HF(PBE1PBE) = -460.567987
Zero-point correction= 0.112184
Thermal correction to Energy= 0.119446
Thermal correction to Enthalpy= 0.120390
Thermal correction to Gibbs Free Energy= 0.080621
Sum of electronic and zero-point Energies= -460.455803
Sum of electronic and thermal Energies= -460.448541
Sum of electronic and thermal Enthalpies= -460.447597
Sum of electronic and thermal Free Energies= -460.487366

$[(^iPr_2N)_2PPMe_3]^+$ parent phosphinophosphonium

P	-0.27148	-2.25296	-0.26843
C	0.11746	-3.18462	1.25253
C	-1.92738	-2.80838	-0.75913
C	0.85546	-2.88707	-1.54352
H	0.60094	-2.43530	-2.50433
H	1.88795	-2.63273	-1.29977
H	1.05281	-2.84123	1.69453
H	0.21405	-4.24270	0.99160
H	-0.68752	-3.07004	1.97803
H	-1.97767	-3.89929	-0.71480
H	-2.13640	-2.48320	-1.77969
H	-2.68016	-2.38503	-0.09205
H	0.75971	-3.97323	-1.61648

(Hartree/Particle)
HF(PBE1PBE) = -460.5638311
Zero-point correction= 0.111881
Thermal correction to Energy= 0.118461
Thermal correction to Enthalpy= 0.119405
Thermal correction to Gibbs Free Energy= 0.081024
Sum of electronic and zero-point Energies= -460.451950
Sum of electronic and thermal Energies= -460.445370
Sum of electronic and thermal Enthalpies= -460.444426
Sum of electronic and thermal Free Energies= -460.482807

[PMe_3]

[Me_2PPMe_3]$^+$ parent phosphinophosphonium

P	0.85097	0.00000	0.01506
C	1.66568	1.46468	-0.66151
C	1.66568	-1.46468	-0.66151
H	1.22779	2.37108	-0.23824
H	1.54566	1.48245	-1.74699
H	2.73056	1.43884	-0.41681
H	1.22779	-2.37108	-0.23824
H	2.73056	-1.43884	-0.41681
H	1.54566	-1.48245	-1.74699
C	1.07200	0.00000	1.81251
H	0.61406	0.89046	2.24788
H	2.13988	0.00000	2.04776
H	0.61406	-0.89046	2.24788

(Hartree/Particle)
HF(PBE1PBE) = -460.8403133
Zero-point correction= 0.112609
Thermal correction to Energy= 0.119678
Thermal correction to Enthalpy= 0.120622
Thermal correction to Gibbs Free Energy= 0.082510
Sum of electronic and zero-point Energies= -460.727704
Sum of electronic and thermal Energies= -460.720635
Sum of electronic and thermal Enthalpies= -460.719691
Sum of electronic and thermal Free Energies= -460.757803

[Et_2PPMe_3]$^+$ parent phosphinophosphonium

C	-1.60986	-2.08182	-0.28235
C	-2.40936	0.62714	-0.98402
H	-1.01704	-2.71303	0.38192
H	-1.38120	-2.33544	-1.32009
H	-2.67090	-2.26749	-0.09641
H	-2.24972	1.69612	-0.83483
H	-3.43430	0.37314	-0.70156
H	-2.25472	0.39284	-2.03947
C	-1.56567	0.01771	1.76418
H	-0.90263	-0.58062	2.39263
H	-2.60305	-0.23028	2.00471
H	-1.39581	1.07628	1.97062

(Hartree/Particle)
HF(PBE1PBE) = -460.8414494
Zero-point correction= 0.112613
Thermal correction to Energy= 0.119675
Thermal correction to Enthalpy= 0.120619
Thermal correction to Gibbs Free Energy= 0.082536
Sum of electronic and zero-point Energies= -460.728837
Sum of electronic and thermal Energies= -460.721774
Sum of electronic and thermal Enthalpies= -460.720830
Sum of electronic and thermal Free Energies= -460.758914

[$^iPr_2PPMe_3$]$^+$ parent phosphinophosphonium

P	-1.56442	-0.08849	-0.03739
C	-2.27965	-1.68158	-0.52655
C	-2.45197	1.11498	-1.06349
H	-2.00461	-2.47431	0.16657
H	-1.94034	-1.94372	-1.53113
H	-3.36775	-1.57446	-0.53831
H	-2.13840	2.13629	-0.85385
H	-3.52384	1.01878	-0.87043
H	-2.25430	0.89418	-2.11480
C	-1.99494	0.21462	1.69868
H	-1.52821	-0.53845	2.33643
H	-3.08042	0.16638	1.82174
H	-1.64516	1.20294	2.00312

(Hartree/Particle)
HF(PBE1PBE) = -460.8432478
Zero-point correction= 0.112534
Thermal correction to Energy= 0.119675
Thermal correction to Enthalpy= 0.120620
Thermal correction to Gibbs Free Energy= 0.082238
Sum of electronic and zero-point Energies= -460.730714
Sum of electronic and thermal Energies= -460.723572
Sum of electronic and thermal Enthalpies= -460.722628
Sum of electronic and thermal Free Energies= -460.761010

[$^tBu_2PPMe_3$]$^+$ parent phosphinophosphonium

P	-1.73709	-0.00138	-0.01480
C	-2.52140	1.61025	-0.30328
C	-2.11470	-0.43935	1.70940
H	-2.38224	1.91399	-1.34227
H	-2.12442	2.37822	0.35974
H	-3.59155	1.49430	-0.10928
H	-1.80503	-1.46324	1.92156
H	-3.19467	-0.35686	1.86281
H	-1.60125	0.23888	2.39209
C	-2.69309	-1.12256	-1.07493
H	-2.50399	-0.87215	-2.12085
H	-3.75552	-0.98500	-0.85634
H	-2.42403	-2.16346	-0.90460

(Hartree/Particle)
HF(PBE1PBE) = -460.8455927
Zero-point correction= 0.112572
Thermal correction to Energy= 0.119670
Thermal correction to Enthalpy= 0.120614
Thermal correction to Gibbs Free Energy= 0.082415
Sum of electronic and zero-point Energies= -460.733020
Sum of electronic and thermal Energies= -460.725923
Sum of electronic and thermal Enthalpies= -460.724979
Sum of electronic and thermal Free Energies= -460.763177

[Ph_2PPMe_3]$^+$ parent phosphinophosphonium

P	0.14756	2.01961	-0.24131
C	0.50191	1.73828	-1.99565
C	1.48376	3.05322	0.40551
H	-0.37926	1.34082	-2.50049
H	1.32893	1.03041	-2.08949
H	0.78249	2.68644	-2.46255
H	1.32011	3.24878	1.46715
H	1.50949	4.00044	-0.13932
H	2.43987	2.54150	0.27620
C	-1.40208	2.93768	-0.09199
H	-2.22583	2.32881	-0.47034
H	-1.33885	3.86617	-0.66497
H	-1.58712	3.17363	0.95831

(Hartree/Particle)
HF(PBE1PBE) = -460.8408213
Zero-point correction= 0.112656
Thermal correction to Energy= 0.119694
Thermal correction to Enthalpy= 0.120638
Thermal correction to Gibbs Free Energy= 0.082627
Sum of electronic and zero-point Energies= -460.728166
Sum of electronic and thermal Energies= -460.721127
Sum of electronic and thermal Enthalpies= -460.720183
Sum of electronic and thermal Free Energies= -460.758194

[(^{i}Pr$_2$N)$_2$PPMe$_3$]$^+$ parent phosphinophosphonium

P	-0.27148	-2.25296	-0.26843
C	0.11746	-3.18462	1.25253
C	-1.92738	-2.80838	-0.75913
C	0.85546	-2.88707	-1.54352
H	0.60094	-2.43530	-2.50433
H	1.88795	-2.63273	-1.29977
H	1.05281	-2.84123	1.69453
H	0.21405	-4.24270	0.99160
H	-0.68752	-3.07004	1.97803
H	-1.97767	-3.89929	-0.71480
H	-2.13640	-2.48320	-1.77969
H	-2.68016	-2.38503	-0.09205
H	0.75971	-3.97323	-1.61648

(Hartree/Particle)
HF(PBE1PBE) = -460.8461978
Zero-point correction= 0.112540
Thermal correction to Energy= 0.119654
Thermal correction to Enthalpy= 0.120598
Thermal correction to Gibbs Free Energy= 0.082315
Sum of electronic and zero-point Energies= -460.733657
Sum of electronic and thermal Energies= -460.726544
Sum of electronic and thermal Enthalpies= -460.725600
Sum of electronic and thermal Free Energies= -460.763882

[R$_2$P]$^+$

[Me$_2$P]$^+$

P	-1.23634	0.00000	-0.70761
C	-1.88918	1.43061	0.26018
C	-1.88918	-1.43061	0.26018
H	-1.45310	2.36629	-0.09637
H	-1.75812	1.34161	1.34101
H	-2.96156	1.48195	0.05027
H	-1.45310	-2.36629	-0.09637
H	-2.96156	-1.48195	0.05027
H	-1.75812	-1.34161	1.34101

(Hartree/Particle)
HF(PBE1PBE) = -420.6384018
Zero-point correction= 0.071262
Thermal correction to Energy= 0.075336
Thermal correction to Enthalpy= 0.076280
Thermal correction to Gibbs Free Energy= 0.045114
Sum of electronic and zero-point Energies= -420.567140
Sum of electronic and thermal Energies= -420.563066
Sum of electronic and thermal Enthalpies= -420.562122
Sum of electronic and thermal Free Energies= -420.593288

[Et$_2$P]$^+$

P	0.81134	0.07359	-0.68763
C	1.69400	-1.28612	0.22983
C	1.24033	1.55788	0.34736
H	1.33069	-2.23668	-0.17257
H	1.46806	-1.26334	1.30149
C	3.20235	-1.18581	0.00201
C	0.51950	2.84134	-0.04640
H	2.31540	1.67877	0.17294
H	1.12571	1.33827	1.41420
H	3.44889	-1.18374	-1.06315
H	3.70126	-2.04469	0.45673
H	3.62795	-0.28696	0.45383
H	0.59394	3.03497	-1.11963
H	0.96225	3.69360	0.47420
H	-0.54103	2.82456	0.22238

(Hartree/Particle)
HF(PBE1PBE) = -499.1920648
Zero-point correction= 0.129514
Thermal correction to Energy= 0.136150
Thermal correction to Enthalpy= 0.137094
Thermal correction to Gibbs Free Energy= 0.099004
Sum of electronic and zero-point Energies= -499.062551
Sum of electronic and thermal Energies= -499.055915
Sum of electronic and thermal Enthalpies= -499.054971
Sum of electronic and thermal Free Energies= -499.093060

[^{i}Pr$_2$P]$^+$

P	0.57938	0.05617	-0.61869
C	1.48498	-1.33404	0.28827
C	1.13027	1.53929	0.40782
C	0.67599	-2.56309	0.68068
H	1.87758	-0.87390	1.20224
C	2.65336	-1.74226	-0.61473
C	0.38514	2.82584	0.06805
C	2.63110	1.72028	0.16373
H	0.97729	1.29369	1.46590
H	2.29156	-2.25009	-1.51345
H	3.30820	-2.43361	-0.07568
H	3.26098	-0.89395	-0.93796
H	0.37811	3.01971	-1.00901
H	0.88750	3.67101	0.54724
H	-0.64550	2.83189	0.43161
H	-0.10151	-2.34453	1.41824
H	1.34736	-3.29523	1.13904
H	0.22355	-3.05011	-0.18766
H	2.84281	1.91601	-0.89211
H	3.22232	0.85934	0.48122
H	2.98267	2.58264	0.73744

(Hartree/Particle)
HF(PBE1PBE) = -577.74 33639
Zero-point correction= 0.186887
Thermal correction to Energy= 0.197021
Thermal correction to Enthalpy= 0.197966
Thermal correction to Gibbs Free Energy= 0.151943
Sum of electronic and zero-point Energies= -577.556477
Sum of electronic and thermal Energies= -577.546343
Sum of electronic and thermal Enthalpies= -577.545398
Sum of electronic and thermal Free Energies= -577.591421

[^{t}Bu$_2$P]$^+$

P	0.36932	0.00201	-0.75905
C	1.12198	-1.57863	0.00626
C	1.12915	1.57872	0.00746
C	0.70192	2.73585	-0.90655
C	2.65273	1.44777	-0.12025
C	0.76095	1.88543	1.45834
C	0.16841	-2.74296	-0.28058
C	1.44023	-1.53849	1.49884
C	2.40295	-1.83760	-0.80463

H	-0.30361	2.10463	1.58154
H	1.30160	2.78163	1.78262
H	1.02961	1.07817	2.14121
H	0.99231	2.55549	-1.94473
H	1.20294	3.65106	-0.57355
H	-0.37051	2.93244	-0.88480
H	2.96197	1.19444	-1.13845
H	3.07795	0.71878	0.57063
H	3.09680	2.41971	0.11940
H	2.19520	-1.90566	-1.87603
H	2.82576	-2.79613	-0.48512
H	3.16856	-1.07758	-0.65106
H	-0.71527	-2.73353	0.36388
H	0.69256	-3.68159	-0.07364
H	-0.14687	-2.77365	-1.32796
H	0.55728	-1.34689	2.11337
H	2.19709	-0.79317	1.74689
H	1.84118	-2.51286	1.80021

(Hartree/Particle)
HF(PBE1PBE) = -656.2900572
Zero-point correction= 0.242493
Thermal correction to Energy= 0.255355
Thermal correction to Enthalpy= 0.256299
Thermal correction to Gibbs Free Energy= 0.204901
Sum of electronic and zero-point Energies= -656.047564
Sum of electronic and thermal Energies= -656.034702
Sum of electronic and thermal Enthalpies= -656.033758
Sum of electronic and thermal Free Energies= -656.085157

$[Ph_2P]^+$

P	0.00471	0.23269	1.07299
H	4.79469	-0.75438	0.95634
C	3.82977	-1.04084	0.55204
C	2.68674	-0.35636	0.94350
H	2.76705	0.44071	1.67800
C	1.43370	-0.71769	0.43096
C	1.33899	-1.80790	-0.43727
H	0.37422	-2.14111	-0.80080
C	2.48519	-2.50031	-0.81063
H	2.40162	-3.35372	-1.47508
C	3.73014	-2.11181	-0.32940
H	4.62029	-2.65731	-0.62346
H	-2.31310	-0.53529	2.42630
C	-2.51082	-0.76671	1.38398
C	-1.53193	-0.51027	0.41718
C	-1.80372	-0.81510	-0.92354
H	-1.05845	-0.64345	-1.69243
C	-3.02746	-1.36261	-1.28292
H	-3.22825	-1.59806	-2.32274
C	-3.99232	-1.61647	-0.31170
H	-4.94576	-2.04826	-0.59658
C	-3.73290	-1.32120	1.02007
H	-4.48014	-1.52205	1.77997

(Hartree/Particle)
HF(PBE1PBE)= -803.786617
Zero-point correction= 0.182674
Thermal correction to Energy= 0.192788
Thermal correction to Enthalpy= 0.193733
Thermal correction to Gibbs Free Energy= 0.145085
Sum of electronic and zero-point Energies= -803.603943
Sum of electronic and thermal Energies= -803.593829
Sum of electronic and thermal Enthalpies= -803.592884
Sum of electronic and thermal Free Energies= -803.641532

$[(^iPr_2N)_2P]^+$

N	1.29006	0.44650	-0.22150
C	1.92411	0.23923	1.09827
C	1.95976	1.22563	-1.30905
C	2.52002	1.49758	1.72544
C	3.39530	0.80027	-1.58900
C	1.84010	2.74067	-1.15052
H	1.38442	0.96629	-2.20617
H	2.79470	1.28408	2.76254
H	1.82223	2.33489	1.72326
H	3.43187	1.80942	1.21175
H	4.08463	1.09605	-0.79498
H	3.73349	1.29441	-2.50386
H	3.47793	-0.27830	-1.74211
H	2.48552	3.13607	-0.36511
H	0.81031	3.03226	-0.92802
H	2.13037	3.22574	-2.08723
H	0.01414	1.57343	2.49097
H	-0.71407	-0.86034	2.46516
C	-1.00193	1.90430	2.27567
H	-2.78532	3.10498	0.44828
H	-1.46117	2.17881	3.22987
H	-0.94663	2.80055	1.65646
C	-1.74075	-0.48834	2.40246
N	-1.57333	0.62651	0.16173
H	-2.69358	-0.28573	-2.25166
C	-2.68175	2.74860	-0.57823
C	-1.82686	0.80649	1.61150
H	-2.07318	-0.31205	3.42893
H	-3.52501	3.14858	-1.14792
H	-2.38287	-1.26456	1.97694
C	-2.69032	0.79988	-2.13103
H	-1.76188	3.16629	-0.99929
C	-2.67100	1.22643	-0.67114
H	-1.84971	1.20434	-2.69969
H	-3.61016	1.18556	-2.57881
H	-3.59720	0.84604	-0.22033
P	-0.26632	-0.00981	-0.68760
H	-2.87400	1.12372	1.65422
C	2.97338	-0.87684	1.10832
H	2.62562	-1.77514	0.59428
H	3.20769	-1.14850	2.14246
H	3.90643	-0.56893	0.63568
H	1.10803	-0.08774	1.75078

(Hartree/Particle)
HF(PBE1PBE) = -924.0487036
Zero-point correction= 0.392658
Thermal correction to Energy= 0.412137
Thermal correction to Enthalpy= 0.413081
Thermal correction to Gibbs Free Energy= 0.347378
Sum of electronic and zero-point Energies= -923.656046
Sum of electronic and thermal Energies= -923.636567
Sum of electronic and thermal Enthalpies= -923.635622
Sum of electronic and thermal Free Energies= -923.701325

$[R_2P]^\cdot$

$[Me_2P]^\cdot$

P	-1.23634	0.00000	-0.70761
C	-1.88918	1.43061	0.26018
C	-1.88918	-1.43061	0.26018
H	-1.45310	2.36629	-0.09637
H	-1.75812	1.34161	1.34101
H	-2.96156	1.48195	0.05027
H	-1.45310	-2.36629	-0.09637
H	-2.96156	-1.48195	0.05027
H	-1.75812	-1.34161	1.34101

(Hartree/Particle)
HF(PBE1PBE)= -420.9399194
Zero-point correction= 0.073204
Thermal correction to Energy= 0.077846
Thermal correction to Enthalpy= 0.078790
Thermal correction to Gibbs Free Energy= 0.045338
Sum of electronic and zero-point Energies= -420.866716
Sum of electronic and thermal Energies= -420.862073
Sum of electronic and thermal Enthalpies= -420.861129
Sum of electronic and thermal Free Energies= -420.894582

[Et$_2$P]$^·$

P	0.81134	0.07359	-0.68763
C	1.69400	-1.28612	0.22983
C	1.24033	1.55788	0.34736
H	1.33069	-2.23668	-0.17257
H	1.46806	-1.26334	1.30149
C	3.20235	-1.18581	0.00201
C	0.51950	2.84134	-0.04640
H	2.31540	1.67877	0.17294
H	1.12571	1.33827	1.41420
H	3.44889	-1.18374	-1.06315
H	3.70126	-2.04469	0.45673
H	3.62795	-0.28696	0.45383
H	0.59394	3.03497	-1.11963
H	0.96225	3.69360	0.47420
H	-0.54103	2.82456	0.22238

(Hartree/Particle)

Zero-point correction= 0.131356
Thermal correction to Energy= 0.138313
Thermal correction to Enthalpy= 0.139257
Thermal correction to Gibbs Free Energy= 0.099725
Sum of electronic and zero-point Energies= -499.354057
Sum of electronic and thermal Energies= -499.347100
Sum of electronic and thermal Enthalpies= -499.346155
Sum of electronic and thermal Free Energies= -499.385687

[^{i}Pr$_2$P]$^·$

P	0.57938	0.05617	-0.61869
C	1.48498	-1.33404	0.28827
C	1.13027	1.53929	0.40782
C	0.67599	-2.56309	0.68068
H	1.87758	-0.87390	1.20224
C	2.65336	-1.74226	-0.61473
C	0.38514	2.82584	0.06805
C	2.63110	1.72028	0.16373
H	0.97729	1.29369	1.46590
H	2.29156	-2.25009	-1.51345
H	3.30820	-2.43361	-0.07568
H	3.26098	-0.89395	-0.93796
H	0.37811	3.01971	-1.00901
H	0.88750	3.67101	0.54724
H	-0.64550	2.83189	0.43161
H	-0.10151	-2.34453	1.41824
H	1.34736	-3.29523	1.13904
H	0.22355	-3.05011	-0.18766
H	2.84281	1.91601	-0.89211
H	3.22232	0.85934	0.48122
H	2.98267	2.58264	0.73744

(Hartree/Particle)
HF(PBE1PBE) = -578.0304584
Zero-point correction= 0.188054
Thermal correction to Energy= 0.198470
Thermal correction to Enthalpy= 0.199414
Thermal correction to Gibbs Free Energy= 0.152157
Sum of electronic and zero-point Energies= -577.842405
Sum of electronic and thermal Energies= -577.831989
Sum of electronic and thermal Enthalpies= -577.831045
Sum of electronic and thermal Free Energies= -577.878302

[^{t}Bu$_2$P]$^·$

P	0.36932	0.00201	-0.75905
C	1.12198	-1.57863	0.00626
C	1.12915	1.57872	0.00746
C	0.70192	2.73585	-0.90655
C	2.65273	1.44777	-0.12025
C	0.76095	1.88543	1.45834
C	0.16841	-2.74296	-0.28058
C	1.44023	-1.53849	1.49884
C	2.40295	-1.83760	-0.80463
H	-0.30361	2.10463	1.58154
H	1.30160	2.78163	1.78262
H	1.02961	1.07817	2.14121
H	0.99231	2.55549	-1.94473
H	1.20294	3.65106	-0.57355
H	-0.37051	2.93244	-0.88480
H	2.96197	1.19444	-1.13845
H	3.07795	0.71878	0.57063
H	3.09680	2.41971	0.11940
H	2.19520	-1.90566	-1.87603
H	2.82576	-2.79613	-0.48512
H	3.16856	-1.07758	-0.65106
H	-0.71527	-2.73353	0.36388
H	0.69256	-3.68159	-0.07364
H	-0.14687	-2.77365	-1.32796
H	0.55728	-1.34689	2.11337
H	2.19709	-0.79317	1.74689
H	1.84118	-2.51286	1.80021

(Hartree/Particle)
HF(PBE1PBE) = -656.5732492
Zero-point correction= 0.243653
Thermal correction to Energy= 0.256710
Thermal correction to Enthalpy= 0.257654
Thermal correction to Gibbs Free Energy= 0.205674
Sum of electronic and zero-point Energies= -656.329596
Sum of electronic and thermal Energies= -656.316539
Sum of electronic and thermal Enthalpies= -656.315595
Sum of electronic and thermal Free Energies= -656.367575

[Ph$_2$P]$^·$

P	0.00471	0.23269	1.07299
H	4.79469	-0.75438	0.95634
C	3.82977	-1.04084	0.55204
C	2.68674	-0.35636	0.94350
H	2.76705	0.44071	1.67800
C	1.43370	-0.71769	0.43096
C	1.33899	-1.80790	-0.43727
H	0.37422	-2.14111	-0.80080
C	2.48519	-2.50031	-0.81063
H	2.40162	-3.35372	-1.47508
C	3.73014	-2.11181	-0.32940
H	4.62029	-2.65731	-0.62346
H	-2.31310	-0.53529	2.42630
C	-2.51082	-0.76671	1.38398
C	-1.53193	-0.51027	0.41718
C	-1.80372	-0.81510	-0.92354
H	-1.05845	-0.64345	-1.69243
C	-3.02746	-1.36261	-1.28292
H	-3.22825	-1.59806	-2.32274
C	-3.99232	-1.61647	-0.31170

H	-4.94576	-2.04826	-0.59658
C	-3.73290	-1.32120	1.02007
H	-4.48014	-1.52205	1.77997

(Hartree/Particle)
HF(PBE1PBE) = -804.0485755
Zero-point correction= 0.182122
Thermal correction to Energy= 0.193051
Thermal correction to Enthalpy= 0.193996
Thermal correction to Gibbs Free Energy= 0.142235
Sum of electronic and zero-point Energies= -803.866454
Sum of electronic and thermal Energies= -803.855524
Sum of electronic and thermal Enthalpies= -803.854580
Sum of electronic and thermal Free Energies= -803.906340

[(^{i}Pr$_2$N)$_2$P]$^·$

N	1.29006	0.44650	-0.22150
C	1.92411	0.23923	1.09827
C	1.95976	1.22563	-1.30905
C	2.52002	1.49758	1.72544
C	3.39530	0.80027	-1.58900
C	1.84010	2.74067	-1.15052
H	1.38442	0.96629	-2.20617
H	2.79470	1.28408	2.76254
H	1.82223	2.33489	1.72326
H	3.43187	1.80942	1.21175
H	4.08463	1.09605	-0.79498
H	3.73349	1.29441	-2.50386
H	3.47793	-0.27830	-1.74211
H	2.48552	3.13607	-0.36511
H	0.81031	3.03226	-0.92802
H	2.13037	3.22574	-2.08723
H	0.01414	1.57343	2.49097
H	-0.71407	-0.86034	2.46516
C	-1.00193	1.90430	2.27567
H	-2.78532	3.10498	0.44828
H	-1.46117	2.17881	3.22987
H	-0.94663	2.80055	1.65646
C	-1.74075	-0.48834	2.40246
N	-1.57333	0.62651	0.16173
H	-2.69358	-0.28573	-2.25166
C	-2.68175	2.74860	-0.57823
C	-1.82686	0.80649	1.61150
H	-2.07318	-0.31205	3.42893
H	-3.52501	3.14858	-1.14792
H	-2.38287	-1.26456	1.97694
C	-2.69032	0.79988	-2.13103
H	-1.76188	3.16629	-0.99929
C	-2.67100	1.22643	-0.67114
H	-1.84971	1.20434	-2.69969
H	-3.61016	1.18556	-2.57881
H	-3.59720	0.84604	-0.22033
P	-0.26632	-0.00981	-0.68760
H	-2.87400	1.12372	1.65422
C	2.97338	-0.87684	1.10832
H	2.62562	-1.77514	0.59428
H	3.20769	-1.14850	2.14246
H	3.90643	-0.56893	0.63568
H	1.10803	-0.08774	1.75078

(Hartree/Particle)
HF(PBE1PBE) = -924.2653159
Zero-point correction= 0.391347
Thermal correction to Energy= 0.411315
Thermal correction to Enthalpy= 0.412260
Thermal correction to Gibbs Free Energy= 0.343401
Sum of electronic and zero-point Energies= -923.873969
Sum of electronic and thermal Energies= -923.854000
Sum of electronic and thermal Enthalpies= -923.853056
Sum of electronic and thermal Free Energies= -923.921915

[R]$^+$

[Me]$^+$

C	-1.88918	1.43061	0.26018
H	-1.45310	2.36629	-0.09637
H	-1.75812	1.34161	1.34101
H	-2.96156	1.48195	0.05027

(Hartree/Particle)
HF(PBE1PBE) = -39.3793856
Zero-point correction= 0.030510
Thermal correction to Energy= 0.033377
Thermal correction to Enthalpy= 0.034321
Thermal correction to Gibbs Free Energy= 0.011416
Sum of electronic and zero-point Energies= -39.348876
Sum of electronic and thermal Energies= -39.346009
Sum of electronic and thermal Enthalpies= -39.345065
Sum of electronic and thermal Free Energies= -39.367970

[Et]$^+$

C	1.24033	1.55788	0.34736
C	0.51950	2.84134	-0.04640
H	2.31540	1.67877	0.17294
H	1.12571	1.33827	1.41420
H	0.59394	3.03497	-1.11963
H	0.96225	3.69360	0.47420
H	-0.54103	2.82456	0.22238

(Hartree/Particle)
HF(PBE1PBE) = -78.7090096
Zero-point correction= 0.057625
Thermal correction to Energy= 0.061028
Thermal correction to Enthalpy= 0.061972
Thermal correction to Gibbs Free Energy= 0.034819
Sum of electronic and zero-point Energies= -78.651385
Sum of electronic and thermal Energies= -78.647981
Sum of electronic and thermal Enthalpies= -78.647037
Sum of electronic and thermal Free Energies= -78.674191

[^{i}Pr]$^+$

C	1.13027	1.53929	0.40782
C	0.38514	2.82584	0.06805
C	2.63110	1.72028	0.16373
H	0.97729	1.29369	1.46590
H	0.37811	3.01971	-1.00901
H	0.88750	3.67101	0.54724
H	-0.64550	2.83189	0.43161
H	2.84281	1.91601	-0.89211
H	3.22232	0.85934	0.48122
H	2.98267	2.58264	0.73744

(Hartree/Particle)
HF(PBE1PBE) = -118.016942
Zero-point correction= 0.086331
Thermal correction to Energy= 0.090056
Thermal correction to Enthalpy= 0.091000
Thermal correction to Gibbs Free Energy= 0.061448
Sum of electronic and zero-point Energies= -117.930611
Sum of electronic and thermal Energies= -117.926886
Sum of electronic and thermal Enthalpies= -117.925942
Sum of electronic and thermal Free Energies= -117.955494

[^{t}Bu]$^+$

C	1.12198	-1.57863	0.00626
C	0.16841	-2.74296	-0.28058
C	1.44023	-1.53849	1.49884
C	2.40295	-1.83760	-0.80463
H	2.19520	-1.90566	-1.87603
H	2.82576	-2.79613	-0.48512
H	3.16856	-1.07758	-0.65106
H	-0.71527	-2.73353	0.36388
H	0.69256	-3.68159	-0.07364
H	-0.14687	-2.77365	-1.32796
H	0.55728	-1.34689	2.11337
H	2.19709	-0.79317	1.74689
H	1.84118	-2.51286	1.80021

(Hartree/Particle)
HF(PBE1PBE) = -157.3124688
Zero-point correction= 0.115025
Thermal correction to Energy= 0.119236
Thermal correction to Enthalpy= 0.120180
Thermal correction to Gibbs Free Energy= 0.088609
Sum of electronic and zero-point Energies= -157.197444
Sum of electronic and thermal Energies= -157.193233
Sum of electronic and thermal Enthalpies= -157.192289
Sum of electronic and thermal Free Energies= -157.223860

[Ph]$^+$

H	4.79469	-0.75438	0.95634
C	3.82977	-1.04084	0.55204
C	2.68674	-0.35636	0.94350
H	2.76705	0.44071	1.67800
C	1.43370	-0.71769	0.43096
C	1.33899	-1.80790	-0.43727
H	0.37422	-2.14111	-0.80080
C	2.48519	-2.50031	-0.81063
H	2.40162	-3.35372	-1.47508
C	3.73014	-2.11181	-0.32940
H	4.62029	-2.65731	-0.62346

(Hartree/Particle)
HF(PBE1PBE)= -230.9743015
Zero-point correction= 0.081916
Thermal correction to Energy= 0.086215
Thermal correction to Enthalpy= 0.087159
Thermal correction to Gibbs Free Energy= 0.054538
Sum of electronic and zero-point Energies= -230.892385
Sum of electronic and thermal Energies= -230.888086
Sum of electronic and thermal Enthalpies= -230.887142
Sum of electronic and thermal Free Energies= -230.919764

[^{i}Pr$_2$N]$^+$

H	0.01414	1.57343	2.49097
H	-0.71407	-0.86034	2.46516
C	-1.00193	1.90430	2.27567
H	-2.78532	3.10498	0.44828
H	-1.46117	2.17881	3.22987
H	-0.94663	2.80055	1.65646
C	-1.74075	-0.48834	2.40246
N	-1.57333	0.62651	0.16173
H	-2.69358	-0.28573	-2.25166
C	-2.68175	2.74860	-0.57823
C	-1.82686	0.80649	1.61150
H	-2.07318	-0.31205	3.42893
H	-3.52501	3.14858	-1.14792
H	-2.38287	-1.26456	1.97694
C	-2.69032	0.79988	-2.13103
H	-1.76188	3.16629	-0.99929
C	-2.67100	1.22643	-0.67114
H	-1.84971	1.20434	-2.69969
H	-3.61016	1.18556	-2.57881
H	-3.59720	0.84604	-0.22033
H	-2.87400	1.12372	1.65422

(Hartree/Particle)
HF(PBE1PBE) = -291.1449448
Zero-point correction= 0.188033
Thermal correction to Energy= 0.196930
Thermal correction to Enthalpy= 0.197874
Thermal correction to Gibbs Free Energy= 0.155355
Sum of electronic and zero-point Energies= -290.956912
Sum of electronic and thermal Energies= -290.948015
Sum of electronic and thermal Enthalpies= -290.947071
Sum of electronic and thermal Free Energies= -290.989590

[R]·

[Me]·

C	-1.88918	1.43061	0.26018
H	-1.45310	2.36629	-0.09637
H	-1.75812	1.34161	1.34101
H	-2.96156	1.48195	0.05027

(Hartree/Particle)
HF(PBE1PBE) = -39.7824967
Zero-point correction= 0.030515
Thermal correction to Energy= 0.033381
Thermal correction to Enthalpy= 0.034325
Thermal correction to Gibbs Free Energy= 0.010767
Sum of electronic and zero-point Energies= -39.751981
Sum of electronic and thermal Energies= -39.749115
Sum of electronic and thermal Enthalpies= -39.748171
Sum of electronic and thermal Free Energies= -39.771730

[Et]·

C	1.24033	1.55788	0.34736
C	0.51950	2.84134	-0.04640
H	2.31540	1.67877	0.17294
H	1.12571	1.33827	1.41420
H	0.59394	3.03497	-1.11963
H	0.96225	3.69360	0.47420
H	-0.54103	2.82456	0.22238

(Hartree/Particle)
HF(PBE1PBE) = -79.0623107
Zero-point correction= 0.060451
Thermal correction to Energy= 0.063948
Thermal correction to Enthalpy= 0.064893
Thermal correction to Gibbs Free Energy= 0.036898
Sum of electronic and zero-point Energies= -79.001860
Sum of electronic and thermal Energies= -78.998362
Sum of electronic and thermal Enthalpies= -78.997418
Sum of electronic and thermal Free Energies= -79.025412

[^{i}Pr]·

C	1.13027	1.53929	0.40782
C	0.38514	2.82584	0.06805
C	2.63110	1.72028	0.16373
H	0.97729	1.29369	1.46590
H	0.37811	3.01971	-1.00901
H	0.88750	3.67101	0.54724
H	-0.64550	2.83189	0.43161
H	2.84281	1.91601	-0.89211
H	3.22232	0.85934	0.48122

H	2.98267	2.58264	0.73744

(Hartree/Particle)
HF(PBE1PBE) = -118.3416484\
Zero-point correction= 0.088980
Thermal correction to Energy= 0.093861
Thermal correction to Enthalpy= 0.094805
Thermal correction to Gibbs Free Energy= 0.061740
Sum of electronic and zero-point Energies= -118.252668
Sum of electronic and thermal Energies= -118.247788
Sum of electronic and thermal Enthalpies= -118.246843
Sum of electronic and thermal Free Energies= -118.279909

[^{t}Bu]·

C	1.12198	-1.57863	0.00626
C	0.16841	-2.74296	-0.28058
C	1.44023	-1.53849	1.49884
C	2.40295	-1.83760	-0.80463
H	2.19520	-1.90566	-1.87603
H	2.82576	-2.79613	-0.48512
H	3.16856	-1.07758	-0.65106
H	-0.71527	-2.73353	0.36388
H	0.69256	-3.68159	-0.07364
H	-0.14687	-2.77365	-1.32796
H	0.55728	-1.34689	2.11337
H	2.19709	-0.79317	1.74689
H	1.84118	-2.51286	1.80021

(Hartree/Particle)
HF(PBE1PBE) = -157.6200591
Zero-point correction= 0.117241
Thermal correction to Energy= 0.123414
Thermal correction to Enthalpy= 0.124358
Thermal correction to Gibbs Free Energy= 0.087851
Sum of electronic and zero-point Energies= -157.502818
Sum of electronic and thermal Energies= -157.496645
Sum of electronic and thermal Enthalpies= -157.495701
Sum of electronic and thermal Free Energies= -157.532209

[Ph]·

H	4.79469	-0.75438	0.95634
C	3.82977	-1.04084	0.55204
C	2.68674	-0.35636	0.94350
H	2.76705	0.44071	1.67800
C	1.43370	-0.71769	0.43096
C	1.33899	-1.80790	-0.43727
H	0.37422	-2.14111	-0.80080
C	2.48519	-2.50031	-0.81063
H	2.40162	-3.35372	-1.47508
C	3.73014	-2.11181	-0.32940
H	4.62029	-2.65731	-0.62346

(Hartree/Particle)
HF(PBE1PBE) = -231.3308918
Zero-point correction= 0.087546
Thermal correction to Energy= 0.091917
Thermal correction to Enthalpy= 0.092862
Thermal correction to Gibbs Free Energy= 0.059504
Sum of electronic and zero-point Energies= -231.243346
Sum of electronic and thermal Energies= -231.238974
Sum of electronic and thermal Enthalpies= -231.238030
Sum of electronic and thermal Free Energies= -231.271388

[^{i}Pr$_2$N]·

H	0.01414	1.57343	2.49097
H	-0.71407	-0.86034	2.46516
C	-1.00193	1.90430	2.27567
H	-2.78532	3.10498	0.44828
H	-1.46117	2.17881	3.22987
H	-0.94663	2.80055	1.65646
C	-1.74075	-0.48834	2.40246
N	-1.57333	0.62651	0.16173
H	-2.69358	-0.28573	-2.25166
C	-2.68175	2.74860	-0.57823
C	-1.82686	0.80649	1.61150
H	-2.07318	-0.31205	3.42893
H	-3.52501	3.14858	-1.14792
H	-2.38287	-1.26456	1.97694
C	-2.69032	0.79988	-2.13103
H	-1.76188	3.16629	-0.99929
C	-2.67100	1.22643	-0.67114
H	-1.84971	1.20434	-2.69969
H	-3.61016	1.18556	-2.57881
H	-3.59720	0.84604	-0.22033
H	-2.87400	1.12372	1.65422

(Hartree/Particle)
HF(PBE1PBE) = -291.4753153
Zero-point correction= 0.191278
Thermal correction to Energy= 0.201084
Thermal correction to Enthalpy= 0.202028
Thermal correction to Gibbs Free Energy= 0.155794
Sum of electronic and zero-point Energies= -291.284037
Sum of electronic and thermal Energies= -291.274231
Sum of electronic and thermal Enthalpies= -291.273287
Sum of electronic and thermal Free Energies= -291.319521

[Me]$^+$

[Me$_2$PPMe$_3$]$^+$ parent phosphinophosphonium

C	1.66568	1.46468	-0.66151
H	1.22779	2.37108	-0.23824
H	1.54566	1.48245	-1.74699
H	2.73056	1.43884	-0.41681

(Hartree/Particle)
HF(PBE1PBE) = -39.3829415
Zero-point correction= 0.030596
Thermal correction to Energy= 0.033462
Thermal correction to Enthalpy= 0.034406
Thermal correction to Gibbs Free Energy= 0.011503
Sum of electronic and zero-point Energies= -39.352345
Sum of electronic and thermal Energies= -39.349480
Sum of electronic and thermal Enthalpies= -39.348536
Sum of electronic and thermal Free Energies= -39.371439

[Et$_2$PPMe$_3$]$^+$ parent phosphinophosphonium

C	-1.60986	-2.08182	-0.28235
H	-1.01704	-2.71303	0.38192
H	-1.38120	-2.33544	-1.32009
H	-2.67090	-2.26749	-0.09641

Hartree/Particle)
HF(PBE1PBE) = -39.3829242
Zero-point correction= 0.030599 (
Thermal correction to Energy= 0.033465
Thermal correction to Enthalpy= 0.034409
Thermal correction to Gibbs Free Energy= 0.011506
Sum of electronic and zero-point Energies= -39.352325
Sum of electronic and thermal Energies= -39.349460
Sum of electronic and thermal Enthalpies= -39.348515

Sum of electronic and thermal Free Energies= -39.371418

$[^{i}Pr_2PPMe_3]^+$ parent phosphinophosphonium

C	-1.99494	0.21462	1.69868
H	-1.52821	-0.53845	2.33643
H	-3.08042	0.16638	1.82174
H	-1.64516	1.20294	2.00312

(Hartree/Particle)
HF(PBE1PBE) = -39.3827754
Zero-point correction= 0.030598
Thermal correction to Energy= 0.033464
Thermal correction to Enthalpy= 0.034408
Thermal correction to Gibbs Free Energy= 0.011505
Sum of electronic and zero-point Energies= -39.352177
Sum of electronic and thermal Energies= -39.349311
Sum of electronic and thermal Enthalpies= -39.348367
Sum of electronic and thermal Free Energies= -39.371270

$[^{t}Bu_2PPMe_3]^+$ parent phosphinophosphonium

C	-2.69309	-1.12256	-1.07493
H	-2.50399	-0.87215	-2.12085
H	-3.75552	-0.98500	-0.85634
H	-2.42403	-2.16346	-0.90460

(Hartree/Particle)
HF(PBE1PBE) = -39.3834049
Zero-point correction= 0.030658
Thermal correction to Energy= 0.033523
Thermal correction to Enthalpy= 0.034468
Thermal correction to Gibbs Free Energy= 0.011567
Sum of electronic and zero-point Energies= -39.352747
Sum of electronic and thermal Energies= -39.349881
Sum of electronic and thermal Enthalpies= -39.348937
Sum of electronic and thermal Free Energies= -39.371838

$[Ph_2PPMe_3]^+$ parent phosphinophosphonium

C	1.48376	3.05322	0.40551
H	1.32011	3.24878	1.46715
H	1.50949	4.00044	-0.13932
H	2.43987	2.54150	0.27620

(Hartree/Particle)
HF(PBE1PBE) = -39.3837578
Zero-point correction= 0.030613
Thermal correction to Energy= 0.033478
Thermal correction to Enthalpy= 0.034422
Thermal correction to Gibbs Free Energy= 0.011520
Sum of electronic and zero-point Energies= -39.353145
Sum of electronic and thermal Energies= -39.350280
Sum of electronic and thermal Enthalpies= -39.349335
Sum of electronic and thermal Free Energies= -39.372238

$[(^{i}Pr_2N)_2PPMe_3]^+$ parent phosphinophosphonium

C	0.85546	-2.88707	-1.54352
H	0.60094	-2.43530	-2.50433
H	1.88795	-2.63273	-1.29977
H	0.75971	-3.97323	-1.61648

(Hartree/Particle)
HF(PBE1PBE) = -39.3830425
Zero-point correction= 0.030624 Thermal
correction to Energy= 0.033490
Thermal correction to Enthalpy= 0.034434
Thermal correction to Gibbs Free Energy= 0.011532
Sum of electronic and zero-point Energies= -39.352419
Sum of electronic and thermal Energies= -39.349553
Sum of electronic and thermal Enthalpies= -39.348609
Sum of electronic and thermal Free Energies= -39.371510

$[Me]^{\cdot}$

$[Me_2PPMe_3]^+$ parent phosphinophosphonium

C	1.66568	1.46468	-0.66151
H	1.22779	2.37108	-0.23824
H	1.54566	1.48245	-1.74699
H	2.73056	1.43884	-0.41681

(Hartree/Particle)
HF(PBE1PBE) = -39.7838079
Zero-point correction= 0.030511
Thermal correction to Energy= 0.033378
Thermal correction to Enthalpy= 0.034322
Thermal correction to Gibbs Free Energy= 0.010763
Sum of electronic and zero-point Energies= -39.753297
Sum of electronic and thermal Energies= -39.750430
Sum of electronic and thermal Enthalpies= -39.749485
Sum of electronic and thermal Free Energies= -39.773045

$[Et_2PPMe_3]^+$ parent phosphinophosphonium

C	-1.60986	-2.08182	-0.28235
H	-1.01704	-2.71303	0.38192
H	-1.38120	-2.33544	-1.32009
H	-2.67090	-2.26749	-0.09641

(Hartree/Particle)
HF(PBE1PBE) = -39.7838032
Zero-point correction= 0.030514
Thermal correction to Energy= 0.033382
Thermal correction to Enthalpy= 0.034326
Thermal correction to Gibbs Free Energy= 0.010766
Sum of electronic and zero-point Energies= -39.753289
Sum of electronic and thermal Energies= -39.750422
Sum of electronic and thermal Enthalpies= -39.749477
Sum of electronic and thermal Free Energies= -39.773037

$[^{i}Pr_2PPMe_3]^+$ parent phosphinophosphonium

C	-1.99494	0.21462	1.69868
H	-1.52821	-0.53845	2.33643
H	-3.08042	0.16638	1.82174
H	-1.64516	1.20294	2.00312

(Hartree/Particle)
HF(PBE1PBE) = -39.7837531
Zero-point correction= 0.030518
Thermal correction to Energy= 0.033385
Thermal correction to Enthalpy= 0.034329
Thermal correction to Gibbs Free Energy= 0.010770
Sum of electronic and zero-point Energies= -39.753235
Sum of electronic and thermal Energies= -39.750368
Sum of electronic and thermal Enthalpies= -39.749424
Sum of electronic and thermal Free Energies= -39.772983

$[^{t}Bu_2PPMe_3]^+$ parent phosphinophosphonium

C	-2.69309	-1.12256	-1.07493
H	-2.50399	-0.87215	-2.12085
H	-3.75552	-0.98500	-0.85634
H	-2.42403	-2.16346	-0.90460

(Hartree/Particle)
HF(PBE1PBE) = -39.7839771
Zero-point correction= 0.030558
Thermal correction to Energy= 0.033426
Thermal correction to Enthalpy= 0.034370
Thermal correction to Gibbs Free Energy= 0.010813
Sum of electronic and zero-point Energies= -39.753419
Sum of electronic and thermal Energies= -39.750551
Sum of electronic and thermal Enthalpies= -39.749607
Sum of electronic and thermal Free Energies= -39.773164

$[Ph_2PPMe_3]^+$ parent phosphinophosphonium

C	1.48376	3.05322	0.40551
H	1.32011	3.24878	1.46715
H	1.50949	4.00044	-0.13932
H	2.43987	2.54150	0.27620

(Hartree/Particle)
HF(PBE1PBE) = -39.784085
Zero-point correction= 0.030507
Thermal correction to Energy= 0.033374
Thermal correction to Enthalpy= 0.034318
Thermal correction to Gibbs Free Energy= 0.010758
Sum of electronic and zero-point Energies= -39.753578
Sum of electronic and thermal Energies= -39.750711
Sum of electronic and thermal Enthalpies= -39.749767
Sum of electronic and thermal Free Energies= -39.773327

$[(^iPr_2N)_2PPMe_3]^+$ parent phosphinophosphonium

C	0.85546	-2.88707	-1.54352
H	0.60094	-2.43530	-2.50433
H	1.88795	-2.63273	-1.29977
H	0.75971	-3.97323	-1.61648

(Hartree/Particle)
HF(PBE1PBE) = -39.7838475
Zero-point correction= 0.030535
Thermal correction to Energy= 0.033403
Thermal correction to Enthalpy= 0.034347
Thermal correction to Gibbs Free Energy= 0.010788
Sum of electronic and zero-point Energies= -39.753312
Sum of electronic and thermal Energies= -39.750445
Sum of electronic and thermal Enthalpies= -39.749501
Sum of electronic and thermal Free Energies= -39.773059

PBE1PBE Optimizations and Frequency Analysis of β-Hydride Elimination Transition States and Products

Cartesian coordinates, enthalpies and Gibbs energies for PBE1PBE/6-311++G(d,p) optimized transition states and products for β-hydride elimination in $[R_2PPMe_3]^+$ cations in the gas phase (298 K) absence of a counterion or solvent. Fragments are listed in the order: TS, and $[R(H)PPMe^3]^+$/alkene. Within each subsection the structures are in the order: R = Et, ^{i}Pr, ^{t}Bu. The quoted HF energies come from the PBE1PBE /6-311++G(d,p) frequency analysis. Data for transition states are listed by the parent phosphinophosphonium from which they are derived. Optimized transition states were obtained using either opt=QST2 or opt=TS Gaussian09 keywords and were confirmed to have one negative (imaginary) frequency in subsequent frequency analysis. For corresponding reactant data see PBE1PBE Optimizations, Frequency Analysis and MP2 Single Point Calculations section $[R_2PPMe_3]^+$.

Transition States (TS)

$[Et_2PPMe_3]^+$ parent phosphinophosphonium

P	1.601890	-0.164515	-0.017724
P	-1.175177	-0.328341	0.257657
C	2.107143	-1.824067	-0.595454
C	2.255381	-0.082007	1.687606
C	-2.566336	-1.405058	-0.546194
C	-1.373043	1.257937	-0.675583
H	1.850742	-1.948693	-1.650208
H	1.585663	-2.593192	-0.020511
H	3.184710	-1.968423	-0.476616
H	2.105407	0.918913	2.099148
H	3.323734	-0.314987	1.708949
H	1.727050	-0.796501	2.323513
C	2.669565	0.982241	-0.965851
H	2.421242	0.929823	-2.028327
H	3.725720	0.726195	-0.837359
H	2.511459	2.008184	-0.626124
H	-2.386917	-2.460435	-0.366139
H	-2.614236	-1.143559	-1.601011
C	-3.424125	-0.701704	0.352885
C	-1.022399	2.469826	0.179009
H	-2.392586	1.336158	-1.060269
H	-0.705963	1.183548	-1.539418
H	-3.766925	-1.186606	1.261309
H	-3.953767	0.190536	0.042068
H	-2.094021	-0.039078	1.371653
H	-1.715876	2.585173	1.016327
H	-1.073170	3.380183	-0.422542
H	-0.010071	2.402589	0.588046

(Hartree/Particle)
HF(PBE1PBE) = -960.0884684
Zero-point correction= 0.242994
Thermal correction to Energy= 0.258287
Thermal correction to Enthalpy= 0.259232
Thermal correction to Gibbs Free Energy= 0.199679
Sum of electronic and zero-point Energies= -959.845474
Sum of electronic and thermal Energies= -959.830181
Sum of electronic and thermal Enthalpies= -959.829237
Sum of electronic and thermal Free Energies= -959.888789

$[^iPr_2PPMe_3]^+$ parent phosphinophosphonium

P	1.636766	-0.146945	-0.054434
P	-1.208881	-0.238662	0.141736
C	2.142716	-0.979781	-1.605764
C	2.192168	-1.315486	1.241895
C	-2.729763	-1.495311	0.125866
C	-1.419451	1.278142	-0.928923
H	1.959258	-0.323940	-2.460428
H	1.559360	-1.893210	-1.745206
H	3.204705	-1.240637	-1.587319
H	2.041487	-0.875834	2.231158
H	3.251752	-1.560822	1.127204
H	1.611930	-2.240066	1.185472
C	2.833214	1.235987	0.089654
H	2.667053	1.957787	-0.713417
H	3.863690	0.872920	0.029942
H	2.694394	1.749733	1.043663
H	-2.920246	-1.658559	-0.934599

C	-3.269272	-0.317303	0.688873
C	-1.821728	2.526694	-0.156711
H	-0.390633	1.407635	-1.283095
H	-3.366572	-0.231758	1.765690
H	-3.853409	0.365005	0.085325
H	-1.029543	0.074970	1.499240
H	-2.843942	2.462306	0.223694
H	-1.768192	3.392533	-0.823886
H	-1.153943	2.719963	0.686712
C	-2.467575	-2.736889	0.929067
H	-3.406194	-3.298824	0.986348
H	-1.716738	-3.374191	0.460257
H	-2.160753	-2.505573	1.951676
C	-2.299995	0.993879	-2.142405
H	-1.970305	0.114786	-2.702829
H	-3.354707	0.869829	-1.883061
H	-2.241749	1.850204	-2.819833

(Hartree/Particle)
HF(PBE1PBE) =-1038.6540042
Zero-point correction= 0.300395
Thermal correction to Energy= 0.318460
Thermal correction to Enthalpy= 0.319404
Thermal correction to Gibbs Free Energy= 0.253040
Sum of electronic and zero-point Energies= -1038.353609
Sum of electronic and thermal Energies= -1038.335545
Sum of electronic and thermal Enthalpies= -1038.334600
Sum of electronic and thermal Free Energies= -1038.400964

[^{t}Bu$_2$PPMe$_3$]$^+$ parent phosphinophosphonium

P	2.208305	-0.129294	-0.049316
P	-0.733232	-0.095937	0.656049
C	2.620260	-1.859549	-0.502147
C	2.975340	0.028238	1.609698
C	-1.838474	-1.513299	-0.065039
C	-1.105841	1.625605	-0.056806
H	2.297947	-2.067237	-1.525497
H	2.107948	-2.552561	0.169717
H	3.696942	-2.040172	-0.431184
H	2.928176	1.066232	1.948409
H	4.022527	-0.287445	1.594939
H	2.432586	-0.588220	2.330715
C	3.348057	0.842433	-1.112618
H	3.073238	0.719992	-2.163142
H	4.385002	0.519217	-0.979845
H	3.276128	1.904190	-0.864324
C	-2.759606	-0.939278	0.888888
C	-0.272027	2.529820	0.867120
H	-2.946969	-1.452178	1.829161
H	-3.510908	-0.228169	0.568839
H	-1.631530	-0.059980	1.827017
H	-0.626737	2.502384	1.901862
H	-0.358351	3.562893	0.515636
H	0.789147	2.266306	0.855454
C	-1.249366	-2.864307	0.300057
H	-2.004841	-3.638655	0.129156
H	-0.382161	-3.095876	-0.321478
H	-0.949318	-2.914003	1.349815
C	-2.566892	2.067442	0.013614
H	-3.212927	1.502828	-0.663512
H	-2.973871	2.012594	1.026785
H	-2.621403	3.114782	-0.301758
C	-2.168874	-1.369328	-1.539284
H	-2.842827	-2.184830	-1.817836
H	-2.672592	-0.430098	-1.770012
H	-1.274335	-1.453781	-2.159443
C	-0.599431	1.749697	-1.496723
H	-1.175441	1.143773	-2.198062
H	-0.703514	2.794500	-1.807553
H	0.453130	1.477128	-1.585410

(Hartree/Particle)
HF(PBE1PBE) = -1117.1858793
Zero-point correction= 0.354859
Thermal correction to Energy= 0.375701
Thermal correction to Enthalpy= 0.376645
Thermal correction to Gibbs Free Energy= 0.305573
Sum of electronic and zero-point Energies= -1116.831021
Sum of electronic and thermal Energies= -1116.810178
Sum of electronic and thermal Enthalpies= -1116.809234
Sum of electronic and thermal Free Energies= -1116.880306

[R(H)PPMe$_3$]$^+$ and alkene

[Et(H)PPMe$_3$]$^+$ and ethane

P	-1.642043	-0.311703	-0.057966
P	0.545148	-0.421250	0.271683
C	-2.215240	-2.025732	-0.114170
C	-2.225866	0.495509	-1.571778
C	0.983677	1.332686	0.692696
H	-1.936869	-2.536880	0.809627
H	-1.752908	-2.546062	-0.956359
H	-3.301462	-2.050947	-0.232984
H	-1.941939	1.548012	-1.581348
H	-3.314959	0.411985	-1.628548
H	-1.784855	0.001114	-2.440187
C	-2.409705	0.491230	1.368894
H	-2.094008	-0.008601	2.287525
H	-3.497336	0.425697	1.283525
H	-2.118799	1.542603	1.414198
C	0.641643	2.454540	-0.274449
H	2.069189	1.261633	0.825948
H	0.582363	1.521722	1.694330
H	1.002222	2.241944	-1.284295
H	1.111164	3.385744	0.053679
H	-0.433922	2.645421	-0.327207
H	4.561348	0.615931	-0.423257
C	4.156588	-0.367848	-0.643932
C	3.882801	-1.243287	0.318589
H	4.025963	-0.614412	-1.693831
H	4.053176	-1.012616	1.366451
H	3.520623	-2.242578	0.095404
H	0.821049	-0.418002	-1.123522

(Hartree/Particle)
HF(PBE1PBE) = -960.1337521
Zero-point correction= 0.243176
Thermal correction to Energy= 0.260142
Thermal correction to Enthalpy= 0.261087
Thermal correction to Gibbs Free Energy= 0.195602
Sum of electronic and zero-point Energies= -959.890576
Sum of electronic and thermal Energies= -959.873610
Sum of electronic and thermal Enthalpies= -959.872666
Sum of electronic and thermal Free Energies= -959.938150

[^{i}Pr(H)PPMe$_3$]$^+$ and propene

P	-0.714327	-1.385862	0.062751
P	-1.163375	0.425759	-1.108147
C	0.152255	-2.518422	-1.047277
C	-2.257481	-2.179060	0.570754
C	3.333275	0.813369	-0.179945
C	-1.220340	1.771395	0.197792
H	1.126084	-2.097301	-1.306566
H	-0.432651	-2.669695	-1.957162
H	0.294712	-3.480547	-0.548401
H	-2.815117	-1.532603	1.250747
H	-2.034789	-3.122552	1.075555

H	-2.870707	-2.376082	-0.311291
C	0.319620	-1.119041	1.521137
H	1.271371	-0.684875	1.204345
H	0.501588	-2.075813	2.018551
H	-0.177716	-0.443709	2.220213
C	3.919545	0.726525	1.189409
H	2.956963	1.791912	-0.479576
C	3.285719	-0.184954	-1.061235
C	-2.503736	1.685070	1.015931
C	-1.121206	3.096362	-0.560063
H	-0.349340	1.674132	0.853375
H	0.212353	0.545167	-1.457334
H	3.697780	-1.164647	-0.828332
H	2.906330	-0.035281	-2.067955
H	-3.390488	1.706840	0.375006
H	-2.566990	2.541052	1.693773
H	-2.553921	0.783556	1.634484
H	4.778531	1.400285	1.277823
H	4.260486	-0.284959	1.423941
H	3.205862	1.046069	1.957856
H	-1.958519	3.225866	-1.252688
H	-0.190559	3.182057	-1.127510
H	-1.151635	3.925204	0.153061

(Hartree/Particle)
HF(PBE1PBE) = -1038.6934496
Zero-point correction= 0.299797
Thermal correction to Energy= 0.319348
Thermal correction to Enthalpy= 0.320292
Thermal correction to Gibbs Free Energy= 0.248548
Sum of electronic and zero-point Energies= -1038.393653
Sum of electronic and thermal Energies= -1038.374102
Sum of electronic and thermal Enthalpies= -1038.373157
Sum of electronic and thermal Free Energies= -1038.444902

[^{t}Bu(H)PPMe$_3$]$^+$ and isobutene

P	-0.282687	-1.352759	0.038176
P	-1.278468	0.303268	-1.030308
C	-1.439207	-2.715212	0.317853
C	3.694907	0.925551	-0.045044
C	-2.672328	0.964731	0.057156
C	0.566876	-0.973888	1.590952
H	-1.958645	-2.958024	-0.611601
H	-2.171810	-2.443422	1.079873
H	-0.883112	-3.592863	0.657707
H	1.312434	-0.198656	1.395399
H	1.069241	-1.877317	1.948876
H	-0.136398	-0.626544	2.347336
C	0.984503	-1.922929	-1.118653
H	0.527493	-2.185580	-2.074554
H	1.492079	-2.797326	-0.702802
H	1.713664	-1.122822	-1.270487
C	-3.917118	0.115935	-0.212410
C	-2.884143	2.379939	-0.500598
C	-2.370456	1.031425	1.549993
C	4.495630	0.121919	-1.024590
C	4.169387	0.882953	1.376035
C	2.648176	1.666743	-0.422462
H	-2.301456	0.036054	2.000208
H	-3.187992	1.550835	2.061668
H	-1.449048	1.581593	1.760863
H	-4.159591	0.076744	-1.277887
H	-4.773932	0.556421	0.307376
H	-3.810531	-0.910286	0.151145
H	-3.063644	2.376994	-1.580391
H	-2.033341	3.034877	-0.291945
H	-3.767011	2.818626	-0.024331
H	-0.236938	1.195987	-0.648792
H	2.351707	1.737233	-1.465715
H	2.116564	2.294704	0.288069
H	4.541178	-0.933781	-0.730972
H	5.532582	0.474806	-1.046294
H	4.097218	0.190468	-2.039215
H	4.197095	-0.145549	1.755882
H	3.545954	1.487472	2.038441
H	5.196187	1.258708	1.444740

(Hartree/Particle)
HF(PBE1PBE) = -1117.2485112
Zero-point correction= 0.355680
Thermal correction to Energy= 0.377722
Thermal correction to Enthalpy= 0.378666
Thermal correction to Gibbs Free Energy= 0.302211
Sum of electronic and zero-point Energies= -1116.892831
Sum of electronic and thermal Energies= -1116.870789
Sum of electronic and thermal Enthalpies= -1116.869845
Sum of electronic and thermal Free Energies= -1116.946300

MP2 Optimizations

Cartesian coordinates for structures pre-optimized at the PBE1PBE/6-311++G(d,p) level (and previously at the HF/6-311++G(d,p) level) and subsequently optimized at the MP2/6-311++G(d,p) level in the gas phase (298 K) absence of a counterion or solvent, in the order: [R$_2$PPMe$_3$]$^+$, [R(H)PPMe$_3$]$^+$, [RPPMe$_3$]$^{+\cdot}$, [RPPMe$_3$], [RPPMe$_2$]$^+$, [R$_2$P]$^+$, [R$_2$P]$^{\cdot}$, [R]$^+$, [R]$^{\cdot}$, alkanes, olefins, [PMe$_3$] derivatives, miscellaneous fragments. Within each subsection the structures are in the order: R = Me, Et, ^{i}Pr, ^{t}Bu, Ph, N^{i}Pr$_2$.

[R$_2$PPMe$_3$]$^+$

[Me$_2$PPMe$_3$]$^+$

P	0.841294	-0.000005	0.013737
P	-1.233007	-0.000006	-0.724495
C	1.668384	1.461429	-0.651208
C	1.668445	-1.461350	-0.651320
C	-1.874880	1.423433	0.262280
C	-1.874883	-1.423439	0.262287
H	1.228788	2.370570	-0.233908
H	1.562882	1.478113	-1.738939
H	2.730393	1.430325	-0.392077
H	1.228878	-2.370546	-0.234109
H	2.730448	-1.430227	-0.392166
H	1.562964	-1.477942	-1.739055
C	1.044413	-0.000059	1.811849
H	0.584331	0.891266	2.244856
H	2.111957	-0.000034	2.052194
H	0.584384	-0.891440	2.244802
H	-1.440812	2.363692	-0.086966
H	-1.727558	1.319973	1.340438
H	-2.950730	1.479343	0.069739
H	-1.440787	-2.363697	-0.086924
H	-2.950726	-1.479377	0.069715
H	-1.727594	-1.319950	1.340447

(Hartree/Particle)
HF(MP2)=-879.4386487
MP2=-880.453661

[Et$_2$PPMe$_3$]$^+$

P	-1.224794	-0.338185	0.016928

P	0.807030	0.078860	-0.737303
C	-1.558675	-2.098051	-0.230722
C	-2.445217	0.568898	-0.960634
C	1.684604	-1.277602	0.184854
C	1.227871	1.551215	0.313268
H	-0.935873	-2.701730	0.432506
H	-1.351561	-2.371973	-1.268818
H	-2.611983	-2.298140	-0.013748
H	-2.307328	1.645880	-0.849066
H	-3.451416	0.297472	-0.628147
H	-2.328769	0.304485	-2.014787
C	-1.494032	0.036669	1.767700
H	-0.794764	-0.533798	2.384469
H	-2.516384	-0.240384	2.042855
H	-1.351041	1.103190	1.954411
H	1.392555	-2.230323	-0.268851
H	1.403906	-1.302472	1.244705
C	3.204709	-1.098946	0.049581
C	0.421106	2.808610	-0.027771
H	2.286314	1.738421	0.100692
H	1.157321	1.308330	1.379942
H	3.502221	-1.020641	-1.000348
H	3.715799	-1.962987	0.480683
H	3.556395	-0.209204	0.576611
H	0.439723	3.017163	-1.101355
H	0.842804	3.674076	0.489089
H	-0.623650	2.727754	0.287125

Convergence criteria reduced to opt=tight
(Hartree/Particle)
HF(MP2)=-957.5220982
MP2=-958.8469582

$[R(H)PPMe_3]^+$

$[Et(H)PPMe_3]^+$

P	1.073942	-0.044829	-0.010886
P	-0.887289	0.934171	0.132392
C	1.462745	-0.768604	1.596860
C	2.303010	1.225147	-0.377610
C	-1.994662	-0.559556	0.072645
H	0.744202	-1.554681	1.842141
H	1.423366	0.005812	2.366936
H	2.466500	-1.201568	1.569458
H	2.110765	1.662178	-1.360629
H	3.301101	0.777996	-0.374534
H	2.259863	2.012734	0.378724
C	1.203021	-1.337098	-1.267947
H	0.522133	-2.159863	-1.039212
H	2.227795	-1.720333	-1.287018
H	0.954920	-0.925467	-2.249551
H	-1.877738	-1.097722	1.019271
H	-1.730354	-1.235293	-0.744695
C	-3.450284	-0.092775	-0.080247
H	-3.745311	0.569092	0.738078
H	-4.116256	-0.958554	-0.072866
H	-3.600025	0.437634	-1.024606
H	-0.883730	1.245232	-1.246287

(Hartree/Particle)
HF(MP2)=-879.43061
MP2=-880.4440123

$[^iPr(H)PPMe_3]^+$

P	1.276081	0.009783	0.042778
P	-0.607810	-0.917338	-0.619575
C	1.762193	1.302133	-1.121600
C	2.553916	-1.267066	0.006543
C	-1.863709	0.085452	0.334115
H	1.018744	2.101605	-1.142021
H	1.847643	0.871988	-2.122760
H	2.728564	1.717214	-0.821705
H	2.341427	-2.029998	0.759134
H	3.526645	-0.813554	0.217300
H	2.585155	-1.735775	-0.980372
C	1.235288	0.713207	1.707167
H	0.495624	1.514752	1.765129
H	2.221396	1.120682	1.950538
H	0.980686	-0.064802	2.431497
C	-2.001610	1.486327	-0.267700
H	-1.566733	0.158621	1.385566
C	-3.188730	-0.686354	0.249975
H	-3.517719	-0.789599	-0.789242
H	-3.961112	-0.135381	0.794755
H	-3.113096	-1.684420	0.691566
H	-1.100469	2.095345	-0.139114
H	-2.822135	2.016376	0.225085
H	-2.230728	1.435214	-1.337555
H	-0.442047	-1.967129	0.313148

(Hartree/Particle)
HF(MP2)=-918.4731691
MP2=-919.6440232

$[^tBu(H)PPMe_3]^+$

P	-1.463621	0.012784	0.003870
P	0.405493	-1.159511	-0.097293
C	-1.576347	1.178243	-1.372861
C	1.817466	0.070945	0.010506
C	-1.837016	0.895108	1.540542
H	-1.349036	0.666782	-2.311702
H	-0.879949	2.007486	-1.233774
H	-2.595745	1.573011	-1.418847
H	-1.789069	0.198062	2.381249
H	-2.850395	1.303529	1.473045
H	-1.129426	1.707434	1.707163
C	-2.773415	-1.213536	-0.219250
H	-2.643885	-1.721880	-1.177515
H	-3.747401	-0.716249	-0.197987
H	-2.733972	-1.955105	0.582960
C	2.118406	0.566888	-1.410809
C	2.995200	-0.783662	0.515018
C	1.599235	1.251705	0.957709
H	0.840792	1.944290	0.576560
H	2.533553	1.818570	1.044484
H	1.314763	0.923331	1.963115
H	2.326228	-0.264990	-2.091207
H	3.004233	1.210839	-1.383846
H	1.295631	1.156069	-1.828958
H	3.159208	-1.668318	-0.110230
H	2.845792	-1.107944	1.550120
H	3.906698	-0.177010	0.477497
H	0.302709	-1.491153	1.274080

(Hartree/Particle)
HF(MP2)=-957.5129537
MP2=-958.8433295

$[^iPr_2N(H)PPMe_3]^+$

$[RPPMe_3]^{+\cdot}$

[MePPMe$_3$]$^{+\cdot}$

P	-0.614304	-0.026757	-0.000482
P	1.353813	0.965930	0.000664
C	-1.870097	1.264024	-0.100659
C	-0.890843	-0.977366	1.513338
C	2.388132	-0.562398	0.001468
H	-1.741938	1.837749	-1.021923
H	-1.778477	1.939159	0.753895
H	-2.865075	0.810602	-0.094985
H	-0.174867	-1.799519	1.581443
H	-1.904257	-1.389242	1.497049
H	-0.780754	-0.328672	2.385790
C	-0.816919	-1.136033	-1.414145
H	-0.651561	-0.588129	-2.345151
H	-1.834482	-1.537922	-1.410428
H	-0.108147	-1.964652	-1.350827
H	2.196291	-1.200883	0.869167
H	3.432550	-0.244628	0.045025
H	2.256449	-1.150828	-0.911802

(Hartree/Particle)
HF(MP2)=-839.7860993
MP2=-840.6188385

[EtPPMe$_3$]$^{+\cdot}$

P	1.052144	-0.033585	0.004716
P	-0.891061	1.003604	-0.039506
c	1.329964	-0.855885	1.592511
c	2.344734	1.204673	-0.222378
c	-1.969177	-0.504281	-0.071659
H	0.585276	-1.639641	1.748975
H	1.262369	-0.126997	2.403812
H	2.327205	-1.305810	1.594330
H	2.224083	1.696999	-1.190456
H	3.326136	0.723949	-0.182640
H	2.281010	1.956706	0.568152
C	1.204796	-1.267270	-1.308562
H	0.473386	-2.065520	-1.165159
H	2.210887	-1.696410	-1.281675
H	1.042060	-0.797840	-2.282063
H	-1.695868	-1.190537	0.739265
H	-1.813133	-1.037198	-1.017940
C	-3.442288	-0.091207	0.061218
H	-3.629191	0.412734	1.012645
H	-4.079340	-0.977276	0.016288
H	-3.739291	0.580371	-0.748467

(Hartree/Particle)
HF(MP2)=-878.8301768
MP2=-879.8158659

[^{i}PrPPMe$_3$]$^{+\cdot}$

P	1.272621	-0.001932	0.047698
P	-0.573608	-0.557993	-1.007245
C	1.284939	1.722126	0.594956
C	2.665810	-0.246748	-1.072999
C	-1.795344	0.037130	0.275248
H	0.463055	1.905396	1.290675
H	1.186669	2.387816	-0.266044
H	2.233586	1.927537	1.100515
H	2.697068	-1.287749	-1.404300
H	3.599709	-0.004827	-0.557884
H	2.558605	0.400459	-1.946930
C	1.525916	-1.052880	1.499135
H	0.698630	-0.933327	2.202715
H	2.460032	-0.767673	1.992279
H	1.586271	-2.099403	1.190472
C	-2.341448	1.412447	-0.147065
H	-1.321849	0.127511	1.260499
C	-2.925011	-1.000339	0.367245
H	-3.409821	-1.135722	-0.605307
H	-3.682514	-0.648014	1.073853
H	-2.563615	-1.973989	0.709312
H	-1.561844	2.177331	-0.207083
H	-3.085855	1.742222	0.584520
H	-2.832490	1.350873	-1.123211

(Hartree/Particle)
HF(MP2)=-917.8720167
MP2=-919.014782

[^{t}BuPPMe$_3$]$^{+\cdot}$

P	-1.439769	-0.001747	0.000441
P	0.418650	-1.207640	-0.016926
C	-1.546528	1.386374	-1.158262
C	1.803264	0.051405	0.002313
C	-1.861532	0.606131	1.654130
H	-1.330680	1.042964	-2.172776
H	-0.848716	2.179647	-0.886355
H	-2.566985	1.781624	-1.125839
H	-1.847630	-0.225265	2.363626
H	-2.867986	1.035037	1.624912
H	-1.151356	1.367860	1.978479
C	-2.730567	-1.176312	-0.472662
H	-2.548710	-1.540159	-1.486856
H	-3.706296	-0.683766	-0.434011
H	-2.729072	-2.027200	0.213155
C	2.010312	0.616660	-1.412986
C	3.039990	-0.776159	0.403804
C	1.599084	1.186078	1.012167
H	0.790439	1.861237	0.713959
H	2.516100	1.784456	1.061590
H	1.397292	0.808818	2.020495
H	2.179819	-0.178530	-2.145962
H	2.898820	1.258858	-1.405174
H	1.164819	1.223597	-1.748486
H	3.210688	-1.612065	-0.282935
H	2.949978	-1.173813	1.419802
H	3.922132	-0.127566	0.368617

(Hartree/Particle)
HF(MP2)=-956.9122116
MP2=-958.2142496

[RPPMe$_3$]

[MePPMe$_3$]

P	-0.520630	0.012092	0.000000
P	1.340959	0.957488	0.000002
C	-1.815250	1.284126	-0.000002
C	-0.933384	-1.057122	1.425662
C	2.362874	-0.628431	-0.000001
H	-1.709762	1.910422	-0.888350
H	-1.709763	1.910426	0.888343
H	-2.804576	0.818240	-0.000002
H	-0.207344	-1.871701	1.495219
H	-1.936928	-1.483940	1.327055

H	-0.869497	-0.458433	2.337059
C	-0.933380	-1.057121	-1.425662
H	-0.869486	-0.458434	-2.337059
H	-1.936925	-1.483937	-1.327060
H	-0.207341	-1.871702	-1.495215
H	2.226176	-1.248087	0.892075
H	3.409181	-0.307172	0.000002
H	2.226179	-1.248081	-0.892081

(Hartree/Particle)
HF(MP2)=-839.9690756
MP2=-840.8400712

[EtPPMe$_3$]

P	0.957421	0.003220	0.000000
P	-0.874417	1.005019	-0.000003
C	1.340207	-1.076280	1.426585
C	2.291231	1.234909	-0.000008
C	-1.952120	-0.549165	-0.000008
H	0.593072	-1.871426	1.496175
H	1.291577	-0.474979	2.337259
H	2.332510	-1.528936	1.328888
H	2.205039	1.864424	-0.888181
H	3.265754	0.738539	-0.000002
H	2.205036	1.864438	0.888156
C	1.340201	-1.076296	-1.426574
H	0.593067	-1.871443	-1.496151
H	2.332506	-1.528950	-1.328875
H	1.291568	-0.475006	-2.337254
H	-1.773491	-1.169066	0.886679
H	-1.773504	-1.169050	-0.886708
C	-3.422807	-0.115455	0.000007
H	-3.649542	0.487954	0.884471
H	-4.089368	-0.984337	0.000001
H	-3.649554	0.487975	-0.884439

(Hartree/Particle)
HF(MP2)=-879.010966
MP2=-880.0361162

[^{i}PrPPMe$_3$]

P	1.179264	-0.020699	0.017247
P	-0.540609	-0.764951	-0.900380
C	1.375662	1.791352	0.176569
C	2.622352	-0.545103	-0.952409
C	-1.783925	0.077281	0.263960
H	0.573917	2.190693	0.803999
H	1.299251	2.242148	-0.815626
H	2.340371	2.044742	0.629223
H	2.609773	-1.631899	-1.060758
H	3.546275	-0.241007	-0.452197
H	2.579095	-0.091562	-1.944669
C	1.548948	-0.597928	1.714836
H	0.710600	-0.354552	2.373412
H	2.460017	-0.134584	2.108262
H	1.662014	-1.684184	1.690064
C	-2.309511	1.398437	-0.308615
H	-1.323855	0.281862	1.241582
C	-2.946698	-0.893373	0.503776
H	-3.407000	-1.178509	-0.449927
H	-3.721634	-0.425061	1.123638
H	-2.605633	-1.808016	0.996162
H	-1.503519	2.118287	-0.476297
H	-3.047996	1.851780	0.367186
H	-2.792468	1.220604	-1.275773

(Hartree/Particle)
HF(MP2)=-918.0519254
MP2=-919.23552

[^{t}BuPPMe$_3$]

P	-1.349297	-0.038064	0.003343
P	0.374865	-1.233610	0.027406
C	-1.587678	1.231012	-1.295186
C	1.780546	0.047834	0.003958
C	-1.881864	0.852626	1.516449
H	-1.397958	0.777216	-2.270505
H	-0.889846	2.059254	-1.150239
H	-2.610387	1.621882	-1.260473
H	-1.943101	0.127986	2.332264
H	-2.858880	1.325372	1.367082
H	-1.149508	1.615655	1.786009
C	-2.718408	-1.201706	-0.280105
H	-2.642428	-1.609387	-1.289763
H	-3.678846	-0.692491	-0.157814
H	-2.649610	-2.023164	0.436648
C	2.051912	0.569917	-1.413561
C	3.011548	-0.744182	0.473170
C	1.585423	1.235433	0.951188
H	0.785731	1.900195	0.603982
H	2.506093	1.835371	0.994825
H	1.344905	0.898941	1.965072
H	2.243021	-0.259769	-2.101162
H	2.930770	1.231988	-1.412533
H	1.201326	1.136668	-1.804842
H	3.175222	-1.629964	-0.151506
H	2.894166	-1.074841	1.510277
H	3.906933	-0.111404	0.405969

(Hartree/Particle)
HF(MP2)=-957.0907963
MP2=-958.4349868

[RPPMe$_2$]$^+$

[MePPMe$_2$]$^+$

P	-0.639170	0.026388	0.004046
P	1.148406	-0.905677	-0.005745
C	-2.125811	-0.994273	0.001387
C	2.316772	0.525668	0.003878
H	-2.710186	-0.795272	0.903349
H	-1.838042	-2.047810	-0.021225
H	-2.727100	-0.761394	-0.881246
C	-1.003123	1.796663	-0.004878
H	-1.699729	2.009347	0.811160
H	-1.465697	2.069389	-0.957592
H	-0.091322	2.376939	0.141944
H	3.178022	0.224603	-0.598219
H	2.667577	0.688722	1.027182
H	1.920918	1.456479	-0.402201

(Hartree/Particle)
HF(MP2)=-800.1058478
MP2=-800.8011118

[EtPPMe$_2$]$^+$

P	0.970694	0.055465	-0.034024
P	-0.567822	-1.241866	-0.166312
C	0.945385	1.842079	-0.307589
C	-1.958454	-0.061502	-0.529636
H	1.166037	2.357406	0.631742

H	-0.034692	2.154716	-0.671193
H	1.707935	2.097443	-1.048984
C	2.610630	-0.567576	0.386135
H	2.964501	-0.076960	1.296754
H	3.303927	-0.359196	-0.432892
H	2.553299	-1.645780	0.551366
H	-2.732921	-0.692894	-0.976888
H	-1.675699	0.674236	-1.287171
C	-2.494741	0.610417	0.743734
H	-2.752892	-0.128457	1.506988
H	-3.397907	1.176993	0.504479
H	-1.761596	1.297999	1.174978

(Hartree/Particle)
HF(MP2)=-839.1499351
MP2=-840.0000572

[^{i}PrPPMe$_2$]$^+$

P	-1.364191	0.042122	-0.000001
P	0.346535	-1.022718	0.000003
C	-1.608835	1.835039	-0.000006
C	-2.933322	-0.851370	0.000003
C	1.647732	0.311175	-0.000002
H	-0.651627	2.357180	-0.000007
H	-2.175634	2.114684	0.892683
H	-2.175631	2.114678	-0.892699
H	-2.735851	-1.925577	0.000006
H	-3.506316	-0.585067	-0.892089
H	-3.506315	-0.585062	0.892093
C	2.490363	0.131341	1.272489
H	1.204413	1.310361	-0.000009
C	2.490369	0.131326	-1.272487
H	2.940406	-0.866192	-1.311202
H	3.302587	0.864756	-1.262264
H	1.903993	0.282754	-2.182983
H	1.903983	0.282780	2.182981
H	3.302581	0.864771	1.262261
H	2.940400	-0.866176	1.311218

(Hartree/Particle)
HF(MP2)=-878.1962581
MP2=-879.200885

[^{t}BuPPMe$_2$]$^+$

P	-1.509523	-0.028676	0.000000
P	0.167743	-1.155247	-0.000001
C	-1.799599	1.759630	0.000000
C	1.601888	0.057924	0.000000
C	-3.075952	-0.930355	0.000001
H	-0.860794	2.310756	-0.000004
H	-2.377307	2.019955	0.892316
H	-2.377314	2.019954	-0.892311
H	-2.875473	-2.003808	0.000001
H	-3.649793	-0.664782	-0.891966
H	-3.649791	-0.664781	0.891968
C	1.606547	0.916065	-1.272566
C	2.839203	-0.862207	0.000004
C	1.606544	0.916067	1.272564
H	0.768975	1.617861	1.310133
H	2.534232	1.499128	1.295540
H	1.577226	0.298698	2.176184
H	1.577233	0.298694	-2.176185
H	2.534235	1.499126	-1.295540
H	0.768978	1.617857	-1.310139
H	2.874199	-1.499443	-0.889706
H	2.874195	-1.499441	0.889715
H	3.736103	-0.233673	0.000005

(Hartree/Particle)
HF(MP2)= -917.2353769
MP2= -918.4004605

[PhPPMe$_2$]$^+$

P	0.826508	1.914522	-1.010987
P	0.945823	0.194642	-2.077835
C	0.021010	3.364151	-1.725736
C	1.516837	2.212093	0.632450
H	-0.391242	3.101181	-2.702591
H	0.750147	4.170725	-1.841717
H	-0.785156	3.696282	-1.066253
H	1.756830	1.252074	1.094814
H	0.766597	2.740337	1.228336
H	2.423198	2.819932	0.564879
H	5.110078	-0.780521	0.470376
C	4.097869	-1.097604	0.235822
C	3.298377	-0.303868	-0.592283
H	3.698411	0.620158	-1.005284
C	1.986401	-0.719276	-0.900712
C	1.510711	-1.962397	-0.437311
H	0.513375	-2.306386	-0.702588
C	2.312833	-2.735993	0.406804
H	1.938481	-3.683921	0.782212
C	3.606161	-2.309557	0.736925
H	4.231811	-2.924265	1.377538

Convergence required keywords symmetry=none and opt=tight,calcfc
(Hartree/Particle)
HF(MP2)= -990.6566099
MP2= -992.0274295

[^{i}Pr$_2$NPPMe$_2$]$^+$

P	2.267391	0.057252	0.538503
N	-0.761702	0.274207	-0.048988
C	2.613211	-1.477860	-0.433284
C	3.597170	1.188238	-0.062812
C	-0.750429	-1.151110	0.397108
C	-2.034559	1.032894	-0.360410
C	-0.756050	-2.089859	-0.814565
C	-2.762571	1.473464	0.909904
C	-2.922519	0.296747	-1.356940
H	-1.670535	1.941963	-0.853472
H	-0.508560	-3.099759	-0.472513
H	-0.019206	-1.785858	-1.562257
H	-1.739370	-2.127259	-1.284874
H	-3.306841	0.660822	1.388908
H	-3.488333	2.240883	0.623730
H	-2.067157	1.917834	1.627565
H	-3.379163	-0.596474	-0.927466
H	-2.371610	0.026040	-2.261120
H	-3.730845	0.975913	-1.643670
P	0.598258	1.177178	-0.275874
H	1.941601	-2.288721	-0.152191
H	3.630412	-1.772170	-0.157356
H	2.575643	-1.316661	-1.512970
H	4.529201	0.881019	0.418849
H	3.393383	2.216178	0.248033
H	3.730567	1.151732	-1.146358
C	-1.835386	-1.499053	1.411034
H	-1.798151	-0.843625	2.283264
H	-1.639275	-2.520942	1.749983
H	-2.836270	-1.481663	0.977969
H	0.208479	-1.255922	0.919215

(Hartree/Particle)

HF(MP2)=-1050.3798784
MP2=-1052.0373728

$[R_2P]^+$

$[Me_2P]^+$

P	0.000000	0.646703	0.000000
C	1.384545	-0.467056	0.022230
C	-1.384545	-0.467056	-0.022230
H	2.278227	-0.018138	0.460513
H	1.591500	-0.556115	-1.069251
H	1.183476	-1.473682	0.395350
H	-2.278227	-0.018138	-0.460513
H	-1.591500	-0.556115	1.069251
H	-1.183476	-1.473682	-0.395350

(Hartree/Particle)
HF(MP2)=-419.6905438
MP2=-420.0972657

$[Et_2P]^+$

P	0.251399	-0.630039	-0.541757
C	1.688367	-0.326857	0.505725
C	-1.122200	-0.259820	0.601043
H	2.610797	-0.790346	0.168739
H	1.529111	-0.387419	1.578830
C	1.446749	1.077165	-0.074950
C	-2.309015	0.427004	-0.088843
H	-0.775340	0.287978	1.483011
H	-1.417336	-1.263645	0.950338
H	0.583824	1.071458	-0.885149
H	2.254596	1.430077	-0.715876
H	1.062973	1.793396	0.649822
H	-2.047698	1.436472	-0.418240
H	-3.140826	0.509045	0.613122
H	-2.654493	-0.141374	-0.956090

(Hartree/Particle)
HF(MP2)=-497.7834703
MP2=-498.5070462

$[^iPr_2P]^+$

P	-0.102840	0.000429	-0.732855
C	-1.469149	-0.173369	0.442364
C	1.361358	-0.034932	0.365077
C	-1.293963	1.361625	0.400332
H	-1.161133	-0.573987	1.407464
C	-2.803180	-0.697541	-0.066569
C	2.436061	0.960937	-0.083162
C	1.849382	-1.499103	0.238297
H	1.059996	0.152726	1.401659
H	-3.033014	-0.299744	-1.059423
H	-3.602317	-0.402537	0.618617
H	-2.785403	-1.787719	-0.127154
H	2.722249	0.791867	-1.126216
H	3.328354	0.829078	0.535132
H	2.105529	1.998766	0.022102
H	-0.864891	1.785198	1.307259
H	-2.173371	1.872535	0.004226
H	-0.519273	1.703413	-0.421364
H	2.130013	-1.744830	-0.791947
H	1.108362	-2.225097	0.587956
H	2.744452	-1.611796	0.856490

(Hartree/Particle)
HF(MP2)=-575.8768902
MP2=-576.9100919

$[^tBu_2P]^+$

P	-0.070744	-0.881772	-0.388061
C	-1.572110	0.084403	0.006084
C	1.483734	0.060820	-0.035314
C	2.598477	-1.000768	-0.140265
C	1.634485	0.816294	1.286566
C	1.567169	1.010834	-1.253552
C	-2.799514	-0.519771	-0.682319
C	-1.626585	1.605439	0.038978
C	-1.269135	-0.591119	1.360090
H	1.385106	0.494616	-2.206740
H	2.588112	1.404960	-1.304078
H	0.873648	1.850584	-1.170767
H	2.549578	-1.721892	0.682564
H	3.567876	-0.494121	-0.089690
H	2.557379	-1.551246	-1.086381
H	1.612616	0.134201	2.142823
H	0.889380	1.599330	1.433775
H	2.617065	1.301088	1.283510
H	-0.399510	-1.391419	1.276231
H	-2.052641	-1.277049	1.690067
H	-0.890720	0.095822	2.115106
H	-2.881568	-0.146797	-1.706605
H	-3.699660	-0.223959	-0.133108
H	-2.759188	-1.613313	-0.714955
H	-1.688667	1.997228	-0.979599
H	-0.772396	2.060185	0.537835
H	-2.534385	1.911562	0.569327

(Hartree/Particle)
HF(MP2)=-653.9557263
MP2=-655.306216

$[Ph_2P]^+$

P	-0.000551	1.543758	0.007997
H	4.705812	0.727856	0.825170
C	3.784045	0.303964	0.438596
C	2.613452	1.055345	0.446012
H	2.613521	2.066252	0.849601
C	1.395287	0.474743	-0.015754
C	1.412587	-0.820665	-0.608584
H	0.514252	-1.224983	-1.065442
C	2.605910	-1.534927	-0.658040
H	2.632498	-2.512137	-1.131238
C	3.782549	-0.983826	-0.122888
H	4.707390	-1.553488	-0.161366
H	-0.513797	-1.234430	1.057527
C	-1.412328	-0.826743	0.604062
C	-1.395758	0.473751	0.022453
C	-2.614226	1.057570	-0.434443
H	-2.614879	2.071930	-0.829271
C	-3.784342	0.305407	-0.433703
H	-4.706324	0.732035	-0.816740
C	-3.782111	-0.987192	0.116618
H	-4.706595	-1.557753	0.150021
C	-2.605209	-1.542155	0.647176
H	-2.631244	-2.523438	1.111901

Convergence required keywords symmetry=none and opt=tight,calcfc
(Hartree/Particle)
HF(MP2)=-800.8291666
MP2=-802.5773586

$[(^iPr_2N)_2P]^+$

N	1.233987	0.240134	-0.079117
C	1.361409	-0.729588	1.050621
C	2.423460	0.863669	-0.763001
C	1.485250	0.018922	2.383035
C	3.049270	-0.077444	-1.793224
C	3.433446	1.466121	0.207301
H	1.989783	1.700181	-1.331687
H	1.388843	-0.706630	3.197248
H	0.688825	0.761491	2.489376
H	2.449689	0.516772	2.487864
H	3.613593	-0.886407	-1.328807
H	3.740775	0.500582	-2.414129
H	2.283278	-0.504785	-2.447119
H	3.958859	0.707338	0.789378
H	2.957658	2.177590	0.886602
H	4.181803	2.006401	-0.380437
H	-2.015949	2.914493	-0.137164
H	-2.180621	1.502534	1.972400
C	-2.724160	2.239418	-0.627032
H	-3.754464	-1.592752	0.416705
H	-3.723319	2.664228	-0.493626
H	-2.510501	2.211139	-1.699168
C	-2.976780	0.914338	1.502586
N	-1.499347	0.033057	-0.258059
H	0.340819	-1.870264	-1.048670
C	-3.045871	-1.956486	-0.328520
C	-2.745343	0.842814	-0.005676
H	-3.933722	1.404092	1.708289
H	-3.019181	-3.046949	-0.248507
H	-2.995257	-0.076796	1.963860
C	-0.708880	-2.152365	-1.128285
H	-3.404875	-1.704090	-1.330820
C	-1.615332	-1.458644	-0.109372
H	-1.054399	-1.913819	-2.139277
H	-0.776684	-3.234752	-0.988056
H	-1.326990	-1.725365	0.915271
P	-0.161863	0.950377	-0.609127
H	-3.554990	0.296552	-0.488043
C	2.456167	-1.777380	0.867281
H	2.338135	-2.338595	-0.062110
H	2.378952	-2.484662	1.698916
H	3.456699	-1.343506	0.898516
H	0.412891	-1.262260	1.076041

(Hartree/Particle)
HF(MP2)=-920.2763295
MP2=-922.6177063

$[R_2P]^{\cdot}$

$[Me_2P]^{\cdot}$

P	0.000000	0.712072	0.000000
C	0.000000	-0.508116	1.387409
C	0.000000	-0.508116	-1.387409
H	0.000000	0.009887	2.349152
H	0.886180	-1.150865	1.342961
H	-0.886180	-1.150865	1.342961
H	0.000000	0.009887	-2.349152
H	-0.886180	-1.150865	-1.342961
H	0.886180	-1.150865	-1.342961

(Hartree/Particle)
HF(MP2)=-419.9743533
MP2=-420.387269

$[Et_2P]^{\cdot}$

P	-0.061098	-0.924874	-0.218961
C	1.577779	-0.365123	0.448469
C	-1.063347	0.589246	0.166297
h	2.271768	-1.206650	0.349399
h	1.471887	-0.166351	1.523136
C	2.162429	0.868222	-0.254604
C	-2.565372	0.335959	-0.001301
H	-0.751592	1.405981	-0.496372
H	-0.842767	0.912198	1.192287
H	2.253568	0.699041	-1.332017
H	3.159218	1.098186	0.134619
H	1.534844	1.750153	-0.104265
H	-2.797099	0.015121	-1.021389
H	-3.141413	1.242717	0.206154
H	-2.910873	-0.447104	0.679698

(Hartree/Particle)
HF(MP2)=-498.0571819
MP2=-498.7762267

$[^iPr_2P]^{\cdot}$

P	0.000000	-0.000003	-0.916743
C	-1.414559	-0.124494	0.294225
C	1.414559	0.124496	0.294223
C	-1.808155	1.264705	0.818679
C	-1.078714	-0.736001	1.142478
C	-2.615271	-0.810661	-0.365644
C	2.615270	0.810659	-0.365651
C	1.808156	-1.264700	0.818685
H	1.078715	0.736008	1.142473
H	-2.947690	-0.245023	-1.243531
H	-3.456067	-0.869573	0.334932
H	-2.370401	-1.825393	-0.693447
H	2.947689	0.245016	-1.243535
H	3.456067	0.869576	0.334924
H	2.370400	1.825389	-0.693459
H	-0.971278	1.773118	1.307510
H	-2.622566	1.177007	1.547524
H	-2.156376	1.899469	-0.003442
H	2.156376	-1.899469	-0.003433
H	0.971280	-1.773111	1.307520
H	2.622567	-1.176998	1.547528

(Hartree/Particle)
HF(MP2)=-576.1412448
MP2=-577.1716305

$[^tBu_2P]^{\cdot}$

P	0.000000	1.068107	0.000001
C	-1.537878	-0.024320	-0.001558
C	1.537878	-0.024320	0.001558
C	2.676120	0.931509	0.403864
C	1.838941	-0.553358	-1.410619
C	1.510263	-1.185887	1.001459
C	-2.676120	0.931510	-0.403861
C	-1.838940	-0.553362	1.410618
C	-1.510264	-1.185884	-1.001462
H	1.294801	-0.836543	2.017025
H	2.496708	-1.669178	1.015631
H	0.773526	-1.946989	0.732927
H	2.737125	1.788948	-0.275457
H	3.633653	0.396171	0.365643
H	2.536332	1.313616	1.420829
H	1.890020	0.265014	-2.137275
H	1.089214	-1.269679	-1.753680
H	2.813813	-1.059817	-1.404744

H	-1.294803	-0.836538	-2.017028
H	-2.496709	-1.669176	-1.015634
H	-0.773526	-1.946986	-0.732932
H	-2.737125	1.788947	0.275463
H	-3.633653	0.396172	-0.365641
H	-2.536333	1.313620	-1.420825
H	-1.890018	0.265008	2.137276
H	-1.089214	-1.269684	1.753676
H	-2.813813	-1.059820	1.404742

(Hartree/Particle)
HF(MP2)=-654.2159522
MP2=-655.5651616

[(^{i}Pr$_2$N)$_2$P]$^{\cdot}$

N	1.245041	-0.003593	-0.258153
C	1.250068	0.781340	0.982702
C	2.462878	-0.507564	-0.918381
C	1.644086	2.254213	0.782482
C	2.958116	-1.828716	-0.315309
C	3.592213	0.516516	-1.041120
H	2.143982	-0.739930	-1.945801
H	1.341118	2.830671	1.665041
H	1.137594	2.672000	-0.090894
H	2.721761	2.381100	0.656233
H	3.381295	-1.687914	0.681609
H	3.736664	-2.257442	-0.957214
H	2.132987	-2.543388	-0.249075
H	4.032472	0.755505	-0.069545
H	3.237099	1.439394	-1.506537
H	4.385074	0.094843	-1.668483
H	-1.381515	2.704285	0.301355
H	-3.223167	1.376134	1.460974
C	-1.737561	2.360426	-0.675916
H	-3.606687	-1.484697	-0.973658
H	-2.443836	3.097557	-1.073318
H	-0.886366	2.302735	-1.358630
C	-3.598405	1.171377	0.451673
N	-1.449181	-0.008315	-0.052638
H	-0.457726	-1.465137	1.992548
C	-2.841066	-2.076183	-0.461270
C	-2.431047	1.006099	-0.523926
H	-4.201685	2.026959	0.131434
H	-3.340332	-2.907798	0.049406
H	-4.256419	0.300327	0.489407
C	-0.996967	-2.069484	1.258215
H	-2.165798	-2.492004	-1.216827
C	-2.053813	-1.228620	0.544966
H	-0.273862	-2.489132	0.552895
H	-1.485348	-2.898800	1.781841
H	-2.742295	-0.880134	1.321126
P	-0.162308	-0.315728	-1.198914
H	-2.846707	0.720660	-1.506936
C	2.069677	0.150580	2.111869
H	1.777110	-0.888334	2.284288
H	1.896007	0.717138	3.033921
H	3.143533	0.182021	1.904994
H	0.199584	0.772733	1.293178

Convergence failure.

Item	Value	Threshold	Converged?
Maximum Force	0.000001	0.000002	YES
RMS Force	0.000000	0.000001	YES
Maximum Displacement	0.000016	0.000006	NO
RMS Displacement	0.000004	0.000004	YES

Predicted change in Energy=-2.147399D-12

(Hartree/Particle)
(From single point calculation)
HF(MP2)=-920.4617148
MP2=-922.7983732

[R]$^+$

[Me]$^+$

C	-1.721505	-0.377270	-0.269197
H	-0.692409	-0.661111	-0.495189
H	-2.235574	0.354326	-0.894639
H	-2.236532	-0.825025	0.582236

(Hartree/Particle)
HF=-39.2436474
MP2=-39.3565317

[Et]$^+$

C	-0.064319	0.693385	0.000000
C	-0.064319	-0.693386	0.000000
H	-0.069513	1.244488	0.937971
H	-0.069513	1.244488	-0.937971
H	-0.069513	-1.244488	0.937971
H	1.049874	0.000000	0.000000
H	-0.069513	-1.244488	-0.937971

(Hartree/Particle)
HF(MP2)=-78.3347249
MP2=-78.61423

[^{i}Pr]$^+$

C	0.000000	0.443391	-0.000002
C	1.287619	-0.203105	-0.000002
C	-1.287619	-0.203105	0.000001
H	0.000000	1.537238	0.000000
H	1.262957	-1.289619	-0.000022
H	1.855659	0.204709	0.857329
H	1.855705	0.204747	-0.857282
H	-1.262957	-1.289619	0.000008
H	-1.855674	0.204721	-0.857314
H	-1.855691	0.204735	0.857297

(Hartree/Particle)
HF(MP2)=-117.4123631
MP2=-117.8345059

[^{t}Bu]$^+$

C	0.002492	-0.004383	-0.020146
C	1.329078	-0.618501	-0.015879
C	-1.203457	-0.833247	-0.014423
C	-0.123281	1.449831	0.018143
H	-0.060139	1.713082	-1.059440
H	0.725297	1.941221	0.498915
H	-1.089417	1.801011	0.382008
H	1.364675	-1.565814	-0.556568
H	1.480972	-0.864867	1.055933
H	2.126644	0.068416	-0.301305
H	-1.043455	-1.856099	-0.351637
H	-2.035794	-0.342953	-0.528008
H	-1.497771	-0.856197	1.053933

(Hartree/Particle)
HF(MP2)=-156.4841016
MP2=-157.0588011

[Ph]$^+$

H	-2.195645	1.403825	-0.000001
C	-1.278150	0.827960	0.000000
C	-0.000002	1.178731	0.000000
C	1.278147	0.827964	0.000001
H	2.195641	1.403830	0.000002
C	1.214845	-0.614107	0.000000
H	2.179977	-1.117122	0.000000
C	0.000002	-1.303867	-0.000001
H	0.000003	-2.388825	-0.000001
C	-1.214842	-0.614111	0.000000
H	-2.179973	-1.117127	-0.000001

(Hartree/Particle)
HF(MP2)=-229.8032427
MP2=-230.5981129

[^{i}Pr$_2$N]$^+$

N	-0.527047	0.549545	0.000002
C	0.970389	0.388085	0.000003
C	-1.399566	-0.404683	-0.000002
C	1.453246	-0.268217	-1.291373
C	-0.981113	1.956908	0.000005
C	-1.170680	-1.869804	-0.000006
H	-2.438488	-0.075248	-0.000002
H	2.543081	-0.190145	-1.324666
H	1.051543	0.248238	-2.167817
H	1.191116	-1.324955	-1.353099
H	-0.584539	2.443583	0.893102
H	-2.069742	1.996119	0.000002
H	-0.584534	2.443588	-0.893087
H	-0.129934	-2.172131	-0.000017
H	-1.677076	-2.287361	-0.878026
H	-1.677058	-2.287363	0.878023
C	1.453242	-0.268236	1.291372
H	1.051534	0.248205	2.167822
H	2.543076	-0.190163	1.324671
H	1.191114	-1.324975	1.353081
H	1.326133	1.421481	0.000012

(Hartree/Particle)
HF(MP2)=-289.6619902
MP2=-290.736737

[R]$^\cdot$

[Me]$^\cdot$

C	0.000000	0.000000	0.000000
H	0.000000	1.079451	0.000000
H	0.934832	-0.539726	0.000000
H	-0.934832	-0.539726	0.000000

(Hartree/Particle)
HF(MP2)=-39.5736132
MP2=-39.7086614

[Et]$^\cdot$

C	-0.796115	0.000000	-0.029981
C	0.696915	0.000000	0.000671
H	-1.348798	-0.926092	0.063768
H	-1.348798	0.926092	0.063768
H	1.106471	-0.886151	-0.491703
H	1.079855	0.000000	1.031724
H	1.106471	0.886151	-0.491702

(Hartree/Particle)
HF(MP2)=-78.620329
MP2=-78.9047924

[^{i}Pr]$^\cdot$

C	-0.014729	0.549853	0.000000
C	-0.014729	-0.201263	-1.292080
C	-0.014729	-0.201263	1.292080
H	0.322676	1.581448	0.000000
H	-0.781703	-0.984176	-1.290583
H	0.949937	-0.704863	-1.464301
H	-0.197008	0.456331	-2.145842
H	-0.781703	-0.984176	1.290583
H	-0.197008	0.456331	2.145842
H	0.949937	-0.704863	1.464301

(Hartree/Particle)
HF(MP2)=-117.6681068
MP2=-118.1037835

[^{t}Bu]$^\cdot$

C	0.000000	0.000000	-0.211208
C	0.000000	-1.479184	0.022225
C	-1.281011	0.739592	0.022225
C	1.281011	0.739592	0.022225
H	2.136179	0.207428	-0.406524
H	1.479662	0.854284	1.102115
H	1.247727	1.746271	-0.406524
H	-0.888451	-1.953699	-0.406524
H	0.000000	-1.708567	1.102115
H	0.888451	-1.953699	-0.406524
H	-2.136179	0.207428	-0.406524
H	-1.247727	1.746271	-0.406524
H	-1.479662	0.854284	1.102115

(Hartree/Particle)
HF(MP2)=-156.7160178
MP2=-157.3050757

[Ph]$^\cdot$

H	-2.141788	1.311371	0.000000
C	-1.208985	0.757744	0.000000
C	-0.000006	1.373619	0.000000
C	1.208979	0.757753	0.000001
H	2.141777	1.311386	0.000001
C	1.191152	-0.618561	0.000000
H	2.130511	-1.165343	0.000000
C	0.000006	-1.302686	0.000000
H	0.000009	-2.387852	-0.000001
C	-1.191146	-0.618570	-0.000001
H	-2.130502	-1.165358	-0.000001

(Hartree/Particle)
HF(MP2)=-230.1112797
MP2=-230.8657075

[^{i}Pr$_2$N]$^\cdot$

N	0.000000	0.000002	-1.140210
C	1.216122	0.366028	-0.425228
C	-1.216121	-0.366027	-0.425228
C	2.012535	-0.892552	-0.032141
C	-2.012536	0.892551	-0.032140
C	-1.084573	-1.332284	0.760156
H	-1.820724	-0.881544	-1.182562

H	3.035152	-0.599603	0.226504
H	2.056581	-1.594718	-0.869422
H	1.574217	-1.398905	0.829803
H	-1.574217	1.398904	0.829804
H	-3.035152	0.599600	0.226506
H	-2.056584	1.594717	-0.869421
H	-0.559129	-0.888752	1.608811
H	-0.567672	-2.249338	0.465381
H	-2.090540	-1.607264	1.095488
C	1.084574	1.332283	0.760157
H	0.567674	2.249338	0.465383
H	2.090541	1.607262	1.095490
H	0.559129	0.888751	1.608812
H	1.820725	0.881545	-1.182561

(Hartree/Particle)
HF(MP2)=-289.8324761
MP2=-290.9018367

Alkanes

Ethane, [CH_3CH_3]

H	0.000000	-1.019829	1.158906
C	0.000000	0.000000	0.764575
H	-0.883198	0.509915	1.158906
H	0.883198	0.509915	1.158906
C	0.000000	0.000000	-0.764575
H	0.883198	-0.509915	-1.158906
H	-0.883198	-0.509915	-1.158906
H	0.000000	1.019829	-1.158906

(Hartree/Particle)
HF(MP2)=-79.2517305
MP2=-79.5716716

Propane, [$CH_3CH_2CH_3$]

H	0.000000	2.170641	0.357326
C	0.000000	1.268443	-0.261405
H	-0.884563	1.300788	-0.906055
H	0.884563	1.300788	-0.906055
C	0.000000	0.000000	0.592016
H	0.878637	0.000000	1.247166
H	-0.878637	0.000000	1.247166
C	0.000000	-1.268443	-0.261405
H	-0.884563	-1.300788	-0.906055
H	0.884563	-1.300788	-0.906055
H	0.000000	-2.170641	0.357326

(Hartree/Particle)
HF(MP2)=-118.2958704
MP2=-118.7674331

Isobutane, [$(CH_3)_2CHCH_3$]

H	-0.814047	-1.989389	0.429615
C	-0.129525	-1.433713	-0.219798
H	0.896019	-1.684352	0.077149
H	-0.277346	-1.780714	-1.247817
C	-0.358733	0.074342	-0.105530
C	0.582260	0.836586	-1.040056
H	1.626377	0.645185	-0.764512
H	0.445627	0.525267	-2.080967
H	0.410524	1.916479	-0.981574
C	-0.179338	0.540469	1.340281
H	-0.864642	0.015821	2.014213
H	-0.363044	1.615708	1.436174
H	0.844907	0.341341	1.677934
H	-1.392359	0.288547	-0.409595

(Hartree/Particle)
HF(MP2)=-157.3405012
MP2=-157.9663207

Neopentane, [$(CH_3)_4C$]

H	-0.004391	-0.513217	-2.116481
C	-0.336837	0.302183	-1.463679
H	0.154562	1.224555	-1.794291
H	-1.417757	0.425837	-1.597351
C	0.000000	0.000000	0.000000
C	-0.477233	1.157242	0.883271
H	0.011640	2.094981	0.594831
H	-1.560677	1.296259	0.791772
H	-0.246890	0.963701	1.937325
C	1.515398	-0.166676	0.151377
H	1.881129	-0.990499	-0.472406
H	1.781546	-0.384010	1.192280
H	2.040079	0.747273	-0.150211
C	-0.701328	-1.292749	0.429031
H	-0.475015	-1.530315	1.474924
H	-0.375429	-2.136808	-0.189760
H	-1.788799	-1.197756	0.329367

(Hartree/Particle)
HF(MP2)=-196.3846498
MP2=-197.167549

Toluene, [$C_6H_5CH_3$]

H	-0.738196	-2.149334	0.018054
C	-0.197082	-1.205260	-0.000551
C	-0.916620	-0.000004	0.018814
C	-0.197090	1.205254	-0.000545
H	-0.738209	2.149325	0.018064
C	1.202625	1.209269	0.008180
H	1.740419	2.153830	-0.002430
C	1.907155	0.000004	-0.018460
H	2.993723	0.000009	-0.012269
C	1.202634	-1.209263	0.008187
H	1.740429	-2.153824	-0.002421
C	-2.425918	-0.000001	-0.009180
H	-2.826922	0.886166	0.491092
H	-2.826924	-0.886305	0.490848
H	-2.798555	0.000143	-1.039606

Convergence failure.

Item	Value	Threshold	Converged?
Maximum Force	0.000003	0.000002	NO
RMS Force	0.000001	0.000001	YES
Maximum Displacement	0.000134	0.000006	NO
RMS Displacement	0.000030	0.000004	NO

Predicted change in Energy=-5.827930D-11

(Hartree/Particle)
(From single point calculation)
HF(MP2)=-269.8006933
MP2=-270.7848281

Diisopropylmethylamine, [$^2Pr_2NMe$]

N	-0.023484	0.499817	-0.697636
C	-1.207609	-0.313340	-0.367230
C	1.254050	0.315600	0.016462
C	-1.957904	0.038112	0.933194
C	2.077129	-0.863644	-0.515526
C	1.187021	0.283723	1.552111

H	1.823969	1.214989	-0.250796
H	-2.929825	-0.468473	0.928170
H	-2.148226	1.112025	1.017280
H	-1.416314	-0.288542	1.823602
H	1.698032	-1.827889	-0.172359
H	3.110344	-0.770679	-0.161937
H	2.079332	-0.855761	-1.608731
H	0.754266	-0.653968	1.912857
H	0.598849	1.116934	1.948374
H	2.200239	0.359764	1.962709
C	-0.932691	-1.814641	-0.420482
H	-0.406466	-2.083580	-1.339660
H	-1.886341	-2.352216	-0.394038
H	-0.345024	-2.150268	0.438594
H	-1.904200	-0.094616	-1.189626
C	-0.366981	1.914473	-0.787866
H	0.412070	2.442858	-1.346473
H	-0.485878	2.417302	0.187856
H	-1.308529	2.021699	-1.336354

(Hartree/Particle)
HF(MP2)=-329.4790462
MP2=-330.750678

Olefins

Ethene, $[CH_2CH_2]$

H	-0.926413	1.235085	0.000000
C	0.000000	0.669575	0.000000
H	0.926413	1.235085	0.000000
C	0.000000	-0.669575	0.000000
H	-0.926413	-1.235085	0.000000
H	0.926413	-1.235085	0.000000

(Hartree/Particle)
HF(MP2)=-78.0553573
MP2=-78.3465275

Propene, $[CH_2CHCH_3]$

H	2.081676	0.878143	0.000000
C	1.294509	0.131313	0.000000
H	1.594920	-0.913293	0.000000
C	0.000000	0.481624	0.000000
H	-0.258117	1.540304	0.000000
C	-1.142470	-0.492876	0.000000
H	-0.777808	-1.523706	0.000000
H	-1.776453	-0.350908	0.881590
H	-1.776453	-0.350908	-0.881590

(Hartree/Particle)
HF(MP2)=-117.1039427
MP2=-117.5465223

Isobutene, $[CH_2C(CH_3)_2]$

H	0.000000	0.928691	2.026870
C	0.000000	0.000000	1.463542
H	0.000000	-0.928691	2.026870
C	0.000000	0.000000	0.119784
C	0.000000	-1.276010	-0.680477
H	0.000000	-2.156609	-0.033100
H	-0.881625	-1.321526	-1.330444
H	0.881625	-1.321526	-1.330444
C	0.000000	1.276010	-0.680477
H	0.000000	2.156609	-0.033100
H	-0.881625	1.321526	-1.330444
H	0.881625	1.321526	-1.330444

(Hartree/Particle)
HF(MP2)=-156.1522477
MP2=-156.7479784

Isopropyliminopropane, $[^iPrNC(CH_3)]$

N	0.160380	-0.780048	0.521858
C	-1.263841	-0.453164	0.361060
C	1.105399	-0.063016	0.027421
C	-1.753431	-0.711348	-1.067288
C	2.521692	-0.520305	0.288209
C	1.023542	1.221274	-0.780165
H	-2.844952	-0.631048	-1.106855
H	-1.470848	-1.720788	-1.380287
H	-1.334463	-0.002154	-1.785301
H	3.107917	0.292450	0.733354
H	3.010739	-0.793056	-0.654472
H	2.517901	-1.380868	0.957879
H	0.063712	1.376338	-1.266713
H	1.806522	1.215861	-1.545233
H	1.221037	2.076007	-0.121928
C	-1.655226	0.927139	0.900155
H	-1.227664	1.073692	1.897145
H	-2.745379	0.990000	0.985638
H	-1.321210	1.745065	0.259177
H	-1.774779	-1.184645	0.998233

(Hartree/Particle)
HF(MP2)=-289.2788437
MP2=-290.3668217

$[PMe_3]$ Derivatives

$[PMe_3]$

P	0.000000	-0.000001	-0.611118
C	1.490585	-0.623763	0.281725
C	-1.285488	-0.979002	0.281725
H	2.367521	-0.034553	-0.002009
H	1.686519	-1.661931	-0.002012
H	1.365275	-0.571328	1.369263
H	-2.282533	-0.629594	-0.002009
H	-1.177420	-0.896702	1.369262
H	-1.213692	-2.033055	-0.002013
C	-0.205097	1.602765	0.281725
H	0.596017	2.291531	-0.002012
H	-0.187854	1.468026	1.369262
H	-1.153835	2.067610	-0.002010

(Hartree/Particle)
HF(MP2)=-459.6237062
MP2=-460.2176363

$[PMe_3]^{+\cdot}$

P	-0.000014	0.000016	-0.340931
C	0.122745	1.724313	0.154031
C	1.431943	-0.968462	0.153853
H	-0.731663	2.283021	-0.233786
H	1.047662	2.156357	-0.233830
H	0.126974	1.783356	1.248816
H	1.343609	-1.985470	-0.234040
H	1.481068	-1.001690	1.248632
H	2.342963	-0.507859	-0.234035
C	-1.554669	-0.755873	0.153982
H	-2.391296	-0.170863	-0.233818

H	-1.607904	-0.781827	1.248766
H	-1.611319	-1.775132	-0.233924

(Hartree/Particle)
HF(MP2)=-459.3773485
MP2=-459.9374246

$[HPMe_3]^+$

P	0.000000	0.000000	0.338856
C	-1.291148	1.121129	-0.213971
C	-0.325353	-1.678731	-0.213971
H	0.452137	-2.349238	0.159144
H	-0.330575	-1.705751	-1.306497
H	-1.297054	-2.010222	0.159120
C	1.616501	0.557602	-0.213971
H	1.808429	1.566183	0.159139
H	1.642513	0.566584	-1.306498
H	2.389431	-0.118168	0.159124
H	-2.260569	0.783056	0.159143
H	-1.311936	1.139162	-1.306498
H	-1.092377	2.128392	0.159120
H	0.000000	0.000000	1.733345

(Hartree/Particle)
HF(MP2)=-460.0053187
MP2=-460.5897019

Miscellaneous fragments

$[H_2PPMe_3]^+$

P	-0.331984	0.000000	-0.003685
P	1.862709	0.000001	0.084339
C	-0.921603	1.466601	0.863871
C	-0.921601	-1.466592	0.863888
H	-0.580827	2.370731	0.353090
H	-0.543109	1.469606	1.888976
H	-2.014889	1.460156	0.884315
H	-0.580822	-2.370727	0.353116
H	-2.014886	-1.460149	0.884332
H	-0.543106	-1.469585	1.888993
C	-1.011834	-0.000010	-1.675252
H	-0.677096	0.890113	-2.213356
H	-2.104703	-0.000011	-1.621914
H	-0.677095	-0.890140	-2.213345
H	1.952947	1.040388	-0.864539
H	1.952946	-1.040398	-0.864524

(Hartree/Particle)
HF(MP2)=-801.3351204
MP2=-802.042413

$[HPPMe_3]^{+\bullet}$

P	0.297520	0.002666	0.000000
P	-1.900061	0.098437	0.000000
C	0.910768	-0.854956	1.466446
C	0.935451	1.688449	-0.000005
H	0.557068	-1.888697	1.470830
H	0.560884	-0.350835	2.370490
H	2.004764	-0.848268	1.452148
H	0.588355	2.219082	-0.890116
H	2.028564	1.665120	-0.000004
H	0.588353	2.219088	0.890101
C	0.910767	-0.854965	-1.466442
H	0.557069	-1.888706	-1.470817
H	2.004763	-0.848275	-1.452145
H	0.560880	-0.350851	-2.370488
H	-1.954501	-1.315371	0.000001

(Hartree/Particle)
HF(MP2)=-800.7334818
MP2=-801.412515

$[P(H){}^tBu_2]$

P	-0.000796	-1.015599	-0.152835
C	1.550967	0.041423	0.005812
C	-1.556322	0.040897	0.003520
C	-2.703755	-0.937221	-0.306369
C	-1.562794	1.150057	-1.054719
C	-1.804093	0.637002	1.394078
C	2.646812	-0.957775	0.415693
C	1.517962	1.173376	1.035508
C	1.909858	0.607501	-1.376175
H	-1.848653	-0.150180	2.154675
H	-2.768731	1.163213	1.398389
H	-1.032966	1.351280	1.686699
H	-2.623777	-1.337253	-1.322189
H	-3.664873	-0.415121	-0.211731
H	-2.709621	-1.782055	0.392679
H	-1.350039	0.753699	-2.054218
H	-0.831484	1.932125	-0.829857
H	-2.554798	1.619469	-1.083019
H	1.993476	-0.192745	-2.119354
H	2.876739	1.124221	-1.319365
H	1.166550	1.326671	-1.732114
H	2.478406	-1.341218	1.428055
H	3.621889	-0.454248	0.399166
H	2.693547	-1.809542	-0.272233
H	1.245786	0.803947	2.029796
H	0.817242	1.964083	0.753154
H	2.516158	1.627535	1.105252
H	-0.004716	-1.481451	1.184639

(Hartree/Particle)
HF(MP2)=-654.816101
MP2=-656.1939943

$[{}^tBu(H)P]^+$

P	0.729146	-0.591769	0.058297
C	-0.788677	0.069212	-0.015567
C	-1.026447	1.550080	0.014899
C	-1.992496	-0.830697	-0.033438
C	2.274924	0.322857	-0.051654
H	2.094147	1.362680	0.225332
H	2.666955	0.270780	-1.069640
H	2.993330	-0.113346	0.645828
H	-1.634368	1.797450	0.891547
H	-1.605313	1.825502	-0.873655
H	-0.114149	2.147810	0.035206
H	-2.618889	-0.608350	0.836529
H	-1.746399	-1.894719	-0.030515
H	-2.579634	-0.607658	-0.930948
H	0.803307	-1.972331	-0.089576

(Hartree/Particle)
HF(MP2)=-497.8105105
MP2=-498.5287987

$[{}^tBu(H)P]^\bullet$

P	1.559106	0.138560	0.000000
C	-0.308386	0.000747	0.000000
C	-0.894571	1.418462	0.000000

C	-0.778723	-0.751174	1.252668
C	-0.778723	-0.751173	-1.252669
H	-0.452979	-0.248515	-2.169689
H	-1.875777	-0.797000	-1.263195
H	-0.395014	-1.776728	-1.268843
H	-0.580608	1.979797	0.886774
H	-1.990352	1.368242	0.000000
H	-0.580608	1.979797	-0.886773
H	-0.452978	-0.248517	2.169689
H	-0.395014	-1.776729	1.268842
H	-1.875777	-0.797001	1.263194
H	1.774938	-1.262916	0.000000

(Hartree/Particle)
HF(MP2)=-498.0524409
MP2=-498.7782461

Dihydrogen, [H_2]

H	0.000000	0.000000	0.369147
H	0.000000	0.000000	-0.369147

(Hartree/Particle)
HF(MP2)=-1.1325014
MP2=-1.1603011

References

[1] Weigand, J. J.; Riegel, S. D.; Burford, N.; Decken, A. *J. Am. Chem. Soc.* **2007**, *129* (25), 7969−7976.
[2] Chitnis, S. S.; MacDonald, E.; Burford, N.; Werner-Zwanziger, U.; McDonald, R. *Chem. Commun.* **2012**, *48* (59), 7359−7361.
[3] Chitnis, S. S.; Robertson, A. P. M.; Burford, N.; Weigand, J. J.; Fischer, R. *Chem. Sci.* **2015**, *6*, 2559−2574.
[4] Burford, N.; Ragogna, P. J.; McDonald, R.; Ferguson, M. J. *J. Am. Chem. Soc.* **2003**, *125*, 14404−14410.
[5] Mallet, C.; et al. AutoHotkey (Version 1.1.19.03) [Software]. Available from http://www.autohotkey.com/.
[6] Pike, S. D.; Pernik, I.; Theron, R.; McIndoe, J. S.; Weller, A. S. *J. Organomet. Chem.* **2015**, *784*, 75−83.
[7] Rai, B., ed. *Molecular modeling for the design of novel performance chemicals and materials*; CRC Press, Taylor and Francis Group, LLC: Boca Raton, 2012; p. 17.
